# Fruit Morphology, Citrulline, and Arginine Levels in Diverse Watermelon (*Citrullus lanatus*) Germplasm Collections

**DOI:** 10.3390/plants9091054

**Published:** 2020-08-19

**Authors:** Awraris Derbie Assefa, On-Sook Hur, Na-Young Ro, Jae-Eun Lee, Ae-Jin Hwang, Bich-Saem Kim, Ju-Hee Rhee, Jung-Yoon Yi, Ji-Hyun Kim, Ho-Sun Lee, Jung-Sook Sung, Myung-Kon Kim, Jae-Jong Noh

**Affiliations:** 1 National Agrobiodiversity Center, National Institute of Agricultural Sciences, RDA, Jeonju 54874, Korea; awrarisderbie@gmail.com (A.D.A.); oshur09@korea.kr (O.-S.H.); nonanona@korea.kr (N.-Y.R.); jnlee88@korea.kr (J.-E.L.); hyj6138@korea.kr (A.-J.H.); bsam92@korea.kr (B.-S.K.); rheehk@korea.kr (J.-H.R.); naaeskr@korea.kr (J.-Y.Y.); wlsghfla5158@naver.com (J.-H.K.); 2 International Technology Cooperation Center, RDA, Jeonju 54875, Korea; hosun83@korea.kr; 3 Upland Crop Breeding Division, Department of Southern Area Crop Science, National Institute of Crop Science, RDA, Miryang 50424, Korea; sjs31@korea.kr; 4 Department of Food Science and Technology, Jeonbuk National University, Jeonju 54896, Korea; kmyuko@jbnu.ac.kr; 5 Jeonbuk Agricultural Research and Extension Services, Iksan 54591, Korea

**Keywords:** genetic resources, fruit morphological characterization, flesh color, principal component analysis, Citrulline Assay Kit, HPLC

## Abstract

Watermelon (*Citrullus lanatus*) is a non-seasonal, economically important, cucurbit cultivated throughout the world, with Asia as a continent contributing the most. As part of the effort to diversify watermelon genetic resources in the already cultivated group, this study was devoted to providing baseline data on morphological quality traits and health-beneficial phytonutrients of watermelon germplasm collections, thereby promoting watermelon research and cultivation programs. To this end, we reported morphological traits, citrulline, and arginine levels of watermelon genetic resources obtained from the gene bank of Agrobiodiversity Center, Republic of Korea, and discussed the relationships between each. Diverse characteristics were observed among many of the traits, but most of the genetic resources (>90%) were either red or pink-fleshed. Korean originated fruits contained intermediate levels of soluble solid content (SSC) while the USA, Russian, Tajikistan, Turkmenistan, Taiwan, and Uruguay originated fruits had generally the highest levels of soluble solids. The citrulline and arginine contents determined using the High Performance Liquid Chromatography (HPLC) method ranged from 6.9 to 52.1 mg/g (average, 27.3 mg/g) and 1.8 to 21.3 mg/g (average, 9.8 mg/g), respectively. The citrulline content determined using the Citrulline Assay Kit ranged from 6.5 to 42.8 mg/g (average, 27.0 mg/g). Resources with high citrulline and arginine levels contained low SSC, whereas red- and pink-colored flesh samples had less citrulline compared to yellow and orange.

## 1. Introduction 

Watermelon (*Citrullus lanatus*) is a cucurbit crop cultivated all over the world. In the year 2018 alone, a production area of 3.2 million ha was utilized, and 103 million tons of watermelons were produced throughout the world. Asia, as a continent, contributed to 81% of the total production, where Korea produced 534,401 tons (0.5%) in the same year (Food and Agricultural Organization of the United Nations (FAO). Watermelon is regarded to be among the top important horticultural crops in South Korea [1]. It is a non-seasonal fruit grown in areas with long frost-free warm periods [2]. Watermelon fruits are produced in different sizes, shapes, rind patterns, flesh colors, and types based on the cultivar. Watermelon fruit is 93% water with small amounts of protein, fat, minerals, and vitamins, and with main nutritional components including, carbohydrates, vitamin A, lycopene, and β-carotene [2,3]. The contents of the metabolites in watermelon fruit varies with cultivar, ploidy, genotype, flesh color, and fruit anatomy [4,5,6,7,8,9]. The flesh of watermelon fruits is consumed in many parts of the world and is proven to be beneficial for disease prevention partly due to the presence of vitamins, lycopene, and amino acids such as citrulline and arginine.

Citrulline and arginine are naturally occurring amino acids in watermelon. Several reports indicate that both exhibit beneficial effects on plant metabolism and human health [3,10]. Citrulline is unusually abundant in watermelon, more than any other plant taxon recorded to date [5,8]. It is also available in most cucurbits, including bitter melon, cucumber, muskmelon, pumpkin, bottle gourd, dishrag gourd, and wax gourd [8,11]. Drought-tolerant watermelon accumulates high levels of citrulline in response to oxidative stress caused by drought conditions [10]. Citrulline is implied to have an important role in nitrogen translocation during germination [12]. Citrulline is used to regenerate arginine—an aminoacid that displays remarkable metabolic regulatory functions [13,14]. Arginine reverses endothelial dysfunction, enhances wound healing, prevents the early stages of tumorigenesis, and improves cardiovascular, reproductive, pulmonary, renal, digestive, and immune functions [15]. In rats, watermelon extract and arginine were shown to improve cardiovascular disease risk factors [16]. Since oral administration of free arginine could result in side effects such as nausea, gastrointestinal discomfort, and diarrhea in humans [17,18], citrulline can be more efficient in diseases related to arginine deficiencies due to its increased bioavailability, better absorption into the bloodstream, and reduced side-effects [8]. Citrulline efficiently scavenges the hydroxyl radical and effectively protects DNA and an enzyme from oxidative injuries [10], and can also exert a positive effect on vasodilation [5]. In vivo studies have indicated that watermelon juice containing citrulline reduced muscle soreness and improved athlete performance [19].

Due to a constant increase in the human population, the demand for high yielding, disease-resistant, and high-nutritive food crops, including watermelon, is intensified. To meet this demand, numerous research and cultivation programs are undergoing research on the conservation and collection of genetically diverse watermelon germplasm, the characterization of quality traits and metabolites, and breeding programs. To diversify the narrow genetic base in terms of important quality traits, morphological characters, and health beneficial phytonutrients, identifying and incorporating new genetic resources available within the watermelon germplasm collections in gene banks into the cultivated crop is crucial. Several reports on the diversity of watermelon genetic resources with regard to their morpholgical traits are available in the literature [20,21,22]. Studies related to biochemical traitsm such as citrulline on germplasm collections, are elusive and and are mostly limited to cultivars [6,8,23]. This study could be of paramount importance to plan future germplasm related studies including trait profiling & characterization and breeding programs, which in turn improve the current level of production, marketability, and nutraceutical effect of watermelon fruits. The main objectives of our study are (a) to characterize 100 watermelon accessions and seven commercial varieties for their fruit morphological characteristics and determine the contents of two important phytonutrients, citrulline (2-amino-5-(carbamoylamino)pentanoic acid) and arginine (2-amino-5-guanidinopentanoic acid); (b) to evaluate the relationship between these metabolites with some selected fruit morphological characters.

## 2. Materials and Methods

### 2.1. Reagents and Chemicals 

A Citrulline Assay Kit containing citrulline standard (240 mM), SDS solution, Proteinase K solution, Assay reagent A, and Assay Reagent B was purchased from Cell Biolabs, Inc. (San Diego, CA, USA). Citrulline and arginine standards for High Performance Liquid Chromatography (HPLC) analysis were purchased from Sigma-Aldrich (St. Louis, MO, USA). All solvents and reagents used in extraction and analysis were HPLC grade.

### 2.2. Plant Material 

#### 2.2.1. Cultivation

Seeds of seven commercial cultivars and 100 watermelon germplasm collections, originating from 22+ countries (AZE, BRA, CHN, IND, IRQ, ITA, JPN, KAZ, KGZ, KOR, MNG, NPL, PHL, RUS, TJK, TKM, TUR, TWN, UKR, URY, USA, UZB, and unknown origin) were obtained from the gene bank of the National Agrobiodiversity Center (NAC), Rural Development Administration (RDA), Jeonju, South Korea. Accession numbers, origin, some selected morphological characters, and photos of the genetic resources investigated in this study are presented as Supplementary Material 1 (Table S1) and 2 (Figure S1). Seeds were sown at a research farm of the NAC in Gochang, Republic of Korea. RDA’s recommended cultural management practices for watermelon were followed in the experimental field. Fertilizers were applied (N–P_2_O_5_–K_2_O = 13.8–4.9–8.7 kg/10a) followed by RDA’s standard and drip irrigation tape, which was used for watering. Seeds were sown on 9 April 2019, and were grown in a nursery bed for 37 days. Seedlings (twelve plants from each accession) were transplanted at an area of 35 cm × 300 cm in a polyethylene film house equipped with an insect net to prevent insect pollination. Plants of the same accession were grown in a single plot (plot area 12.6 m^2^). They were pollinated by hand and harvested after 45 days (on average) of pollination. 

#### 2.2.2. Sampling 

Fruits of the watermelon were harvested at a fully mature stage when the commercial varieties are ready to eat after 45 days of pollination (accumulated temperature of 1000 °C), collected, stored in polyethylene bags, and immediately transferred to a −18 °C walk-in freezer until further processing. Flesh (mesocarp) of the watermelon fruit was carefully separated from the seeds and rind manually, and the edible part was juiced, frozen at −80 °C, and lyophilized using a vacuum freeze drier (Ilishibiobase, Rijssen, The Netherlands). Lyophilized powdered samples were sealed to prevent moisture absorption and stored at −20 °C until analysis. 

#### 2.2.3. Analysis of Morphological Properties

The morphological characters were evaluated at commercial maturity stage at the field and in the laboratory. Watermelon fruit samples were evaluated for 14 morphological characters based on modified descriptors of The International Union for the Protection of New Varieties of Plants (UPOV) for watermelon fruits [24]. Six quantitative characters (size of pistil scar (SPS), the width of stripes (WS), weight of fruit (WF), length of fruit (LF), width of fruit (WIF), the thickness of outer layer of the pericarp (TP) (measured at the equatorial cross-section), and soluble solids content (SSC)) were evaluated with the help of a meter, digital caliper, digital balance, and a handheld electronic refractometer, as required. Ten to 12 fruit samples were used to measure the quantitative morphological characters. 

### 2.3. Extraction and Analysis of Citrulline and Arginine 

Extracts were prepared by dissolving 0.05 g of the freeze-dried powdered samples of watermelon using 10 mL HPLC grade water in a 15 mL conical plastic tube. The solution was kept in an automatic shaker for 30 min, centrifuged, and then the supernatant was filtered using a 0.45 μm microsyringe filter. Extract solutions were stored at −20 °C for subsequent analysis. 

The analysis of the citrulline and arginine was conducted using a Waters HPLC system equipped with a 2690 separation module and waters 996 diode array detector (Milford, MA, USA). UV-Vis detection was set at 200 nm, and the column temperature was held at room temperature. Separation of the compounds was done using a Gemini C_18_ (3 µm partial size, 250 × 4.6 mm, Phenomenex, Torrance, CA, USA) column. The mobile phase was 0.1% phosphoric acid in an aqueous solution, and the elution mode was isocratic. Flow rate and total run time were set at 0.3 mL/min and 15 min, respectively. Extraction and analysis of all the samples were carried out in triplicates. The citrulline and arginine concentrations were calculated using linear calibration functions prepared using the serial dilutions (0.1, 1, 10, 25, 50, 100, 250, 500, and 1000 μg/mL) of commercial standards, and the final results were expressed as milligram per gram (mg/g) of freeze-dried watermelon sample.

### 2.4. Analysis of Citrulline Using Citrulline Assay Kit 

Citrulline was also estimated by using the Citrulline Assay Kit (CAK), a colorimetric assay that measures the amount of citrulline present in biological samples in a 96-well microliter plate format. Briefly, 50 μL of the watermelon sample extract (see extraction procedure in Section 2.3) or citrulline standard are treated with sodium dodecyl sulfate (SDS) (5 μL) and proteinase K (5 μL) in 2 mL screwcap tubes. The mixture was mixed thoroughly by pipetting up and down, and was incubated for 2 h at 37 °C. Assay reagents (250 µL Assay Reagent A, 50 µL Assay Reagent B) were added to each tube, and then the tubes were closed tightly, mixed well, and incubated for 30 min at 95 °C. The tubes were transferred to 4 °C and held for five minutes, followed by centrifugation at 18,000× *g* for 10 min at room temperature. A measure of 200 µL of the supernatant was pipetted to a 96-well plate, and absorbance was read at 550 nm using a microplate reader spectrophotometer (Molecular Devices, VERSAmax tunable, San Jose, CA, USA). Citrulline concentration in unknown samples was determined by comparison with a predetermined standard curve obtained from the absorbance of serial dilutions (0, 37.5, 75, 150, 300, 600, 1200, 2400 μM) of the citrulline standard prepared using 0.1 M sodium phosphate buffer (PBS) (pH 7.5). 

### 2.5. Statistical Analysis

All experiments were conducted in triplicates. Results were reported as mean ± standard deviation and expressed in mg/g of flesh dry weight (DW). Analysis of variance (ANOVA), followed by the Duncan multiple range tests (*p* < 0.05), conducted using SPSS V 25 statistical program (SPSS Inc., Chicago, IL, USA), was employed to determine the significance of the variation between the mean concentration of each compound between the samples. Correlation analysis was also done using SPSS V 25 statistical program. Principal Component Analysis (PCA) was conducted using R-program (Version 3.6.1, RStudio, Inc., Boston, MA, USA).

## 3. Results and Discussion 

### 3.1. Fruit Morphological Characteristics

The variation in qualitative and quantitative morphological characters in 105 watermelon fruit samples is summarized in Table 1. Watermelon fruit samples have portrayed quite a wide range of diversity of fruit size, shape, skin color, flesh color, outer skin patterns, weight, and SSC. Most samples (94.3%) had a green ground skin color, and the remaining exhibited white skin. The intensity of the green color of the skin showed quite a range of diversity, which was described in nine levels from very light to very dark. Most of the resources were circular (60.6%) and broad elliptic (28.8%) in the longitudinal section. Fruits shapes that were truncated and moderately rounded at the apical part dominated the entire resources studied, comprising 90.5%. Grooving is absent in 85% of the accessions, and if present, it was mostly on the whole fruit. Only a single material (S/No 41, IT 203049) showed grooving at the basal half. Stripes were conspicuous in 66.7% of the resources. Considerable variation was observed in the main color of the flesh, where most resources exhibted pink, red, or a mixture of both. Yellow flesh color was observed in five resources, while white-colored flesh watermelon fruits were not observed. In concordance with this study, yellow and white flesh color were quite scarce in previous reports [20,21,25]. 

Other morphological characters evaluated quantitatively had also shown a wide range of variation. The average, maximum, and minimum values of seven quantitative measurements of watermelon fruit samples are presented in Table 1. The highest diversity was observed on the width of the stripes (0.0 to 29.8 mm), size of the pistil scar (2.0 to 32.6 mm), and thickness of the outer layer of pericarp (4.6 to 22.6 mm). The WF, SSC, length, and width of the fruit had also shown quite a wide diversity, but the medium character was common in most of the accessions. The average values of SSC, thickness of the outer layer of the pericarp, and length and width of fruits are comparable with previous reports; however, the fruit weight and size of the pistil scar were higher in the resources investigated in our study [8,20,21,22,26].

Some morphological characters showed diversity based on the resources country of origin. Analysis of the average quantitative morphological characters showed that Korean origin watermelon fruits were characterized by a smaller size of the pistil scar, conspicuous longer width of stripes, intermediate SSC, red/pink colored flesh, absence of grooves, smaller rounded shape at the apical, and a varied shape at the longitudinal section. On the other hand, stripes are absent in watermelon fruits originating from Iraq, Kyrgyzstan, Mongolia, and Uruguay, while fruits from Italy and Brazil exhibited a longer width of stripes. The USA, Russian, Tajikistan, Turkmenistan, Taiwan, and Uruguay originated fruits had generally higher SSC compared to other origins. Indian, Brazilian, USA, and Chinese originated fruits were larger in weight and length (Supplementary Material Table S1). 

**Figure 1:**
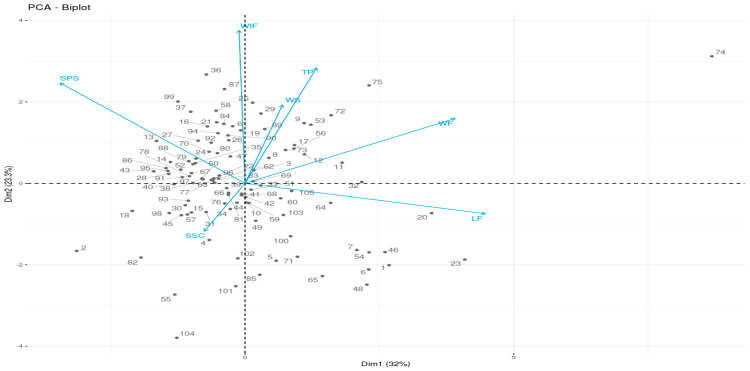
Principal Component Analysis (PCA)-Biplot of watermelon fruit samples with morphological characters measured quantitatively. SPS = Size of pistil scar (mm); WS = Width of stripes (mm); WF = Weight of fruit (kg); LF = Length of fruit (cm); WIF = Width of fruit (cm); TP = Thickness of outer layer of pericarp (mm); SSC = Soluble solids content (Brix).

Principal Component Analysis (PCA) for the seven quantitative morphological traits was conducted (Figure 1). Results of the Principal Component Analyses (for the first three PC’s) of the seven quantitative morphological traits are presented in the Supplementary Material 1 (See Table S2). The first three principal components, with eigenvalues greater than 1, explained 32.0, 23.3, and 19.2% of the variations, respectively, making a total of 74.5%. Weight and length of fruit and size of the pistil scar were important variables composing PC1, while PC2 was mostly constructed from the width of the fruit, the thickness of the outer layer of the pericarp, size of the pistil scar, and the width of the stripes. The SSC was highly correlated with PC3. Some of the characters showed a significant correlation to each other; for example, the weight of the fruit was highly correlated with the length and width of fruit. Other important significant correlations (*p* < 0.01) worth noting are the size of pistil scar vs. the weight, length, and width of fruit. The size of the pistil scar was negatively correlated with the former two characters, whereas it showed a strong positive correlation with the latter. Thicker pericarp watermelon fruits were also found to possess less SSC, as observed from the PCA and Pearson correlation data (Table 2, Figure 1; Supplementary Material 1, Table S2).

### 3.2. Analysis of Citrulline and Arginine Using HPLC and 

#### 3.2.1. Method Development and Validation 

**Figure 2:**
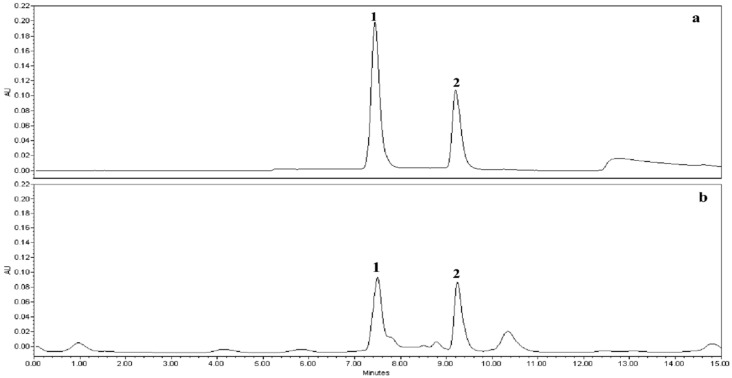
A representative HPLC chromatogram showing the isocratic RP-HPLC separation of arginine (peak 1) and citrulline (peak 2): Mixed standard (**a**); watermelon fruit sample (**b**).

The separation and analysis of citrulline and arginine in mixed standard and watermelon extracts were performed using selected mobile phases and a chromatographic column according to earlier reports, but with slight modifications [8,27,28]. Citrulline and arginine in watermelon flesh samples were efficiently separated in the Gemini C_18_ column using 0.1% phosphoric acid in an aqueous solution as a mobile phase and isocratic elution mode. The Gemini column, a first-generation stationary phase obtained from Phenomenex, is end-capped with porous silica used as a base core, and a layer of embedded silica is coated on the top of the silica core [28,29]. In this column and the specified mobile phase condition, arginine and citrulline were eluted at 7.5 and 9.3 min, respectively (Figure 2).

Calibration curves were prepared from an average of three independent standard solutions of serial dilutions in a concentration range of 5–500 µg/mL for citrulline and 0.1–1000 µg/mL for arginine versus the peak areas in the HPLC analysis. The linearity of the method for the quantification of citrulline using a Citrulline Assay Kit (CAK) was constructed in the concentration range of 0.3285 to 10.512 µg/mL. Regression analysis of the standard experimental data points in the HPLC method demonstrated a linear relationship with correlation coefficients (*R*^2^) of 0.999 and 0.997, and the regression equations of Y = 15716X − 11226 and Y = 36706X + 383305 (X stands for the concentration and Y for peak area), for citrulline and arginine, respectively. The regression equation and correlation coefficients (*R*^2^) for citrulline analysis using CAK were Y = 0.0414X + 0.0785 (X stands for concentration and Y stands for absorbance) and 0.994, respectively. 

Recovery test, inter- and intra-day precision, and the effect of the sample size were performed using two watermelon fruit samples. The recovery test verified the efficiency of the method for the extraction and analysis of citrulline and arginine. The data obtained by spiking 2 to 10 mg standards showed a mean recovery of 100.70 to 107.71% for citrulline, 96.47 to 102.28% for arginine, and 95.68 to 101.75% for citrulline using CAK, suggesting the reliability and accuracy of the method. Recovery test results are tabulated in Supplementary Material 1 (Table S3). The precision of the method was determined as the percentage of the ratio of the standard deviation to the mean value (relative standard deviation, RSD) of the inter-day (*n* = 5) and intra-day (*n* = 5) analyses. The RSD (%) of the intra-day and inter-day precisions were in the range between 0.63–5.49 and 0.95–4.48, respectively. The precision results for each compound using the HPLC and CAK methods are presented in Supplementary Material 1 (Table S4). The effect of sample size (sample to solvent ratio) on the responses of the instruments was evaluated in two watermelon fruit samples for citrulline (see Supplementary Material 1, Figure S2). For HPLC analysis, a linear response was exhibited in the range of 25 to 500 mg dry weight of watermelon fruit samples. The linearity of the responses using the Citrulline Assay Kit method was observed for a sample concentration of 10 to 75 mg dry weight of watermelon fruit. Alcohols are commonly used for the extraction of citrulline and arginine instead of water. Ridwan et al. (2018) [28] and this study ensure the use of water with two different extraction and measurement methods. Other authors either simply thawed, diluted, centrifuged, or used the supernatant for analysis [11,23]. On the other hand, Hartman et al. (2019) [8] and Soteriou et al. (2017) [30] extracted the frozen samples in acidified solvent.

#### 3.2.2. Determination of Citrulline and Arginine Content Using HPLC 

**Figure 3:**
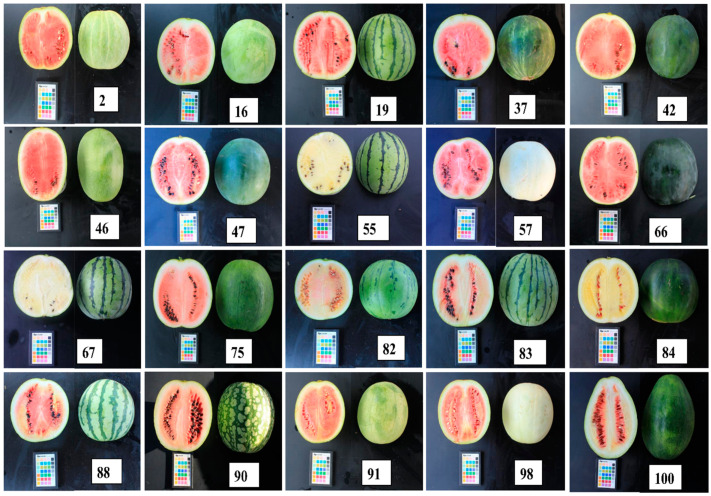
Representative photos of watermelon fruit samples exhibiting, relatively, the highest level of citrulline and arginine. The numbers in each photo correspond to S/No in Table 3.

A wide range of diversity in citrulline and arginine levels was exhibited by the investigated accessions and commercial varieties. The results are tabulated in Table 3. The average citrulline and arginine content in the entire genetic resources were 27.3 and 9.8 mg/g, with ranges of 6.9 (S/No 12) to 52.1 (S/No 82) mg/g and 1.8 (S/No 12) to 21.3 (S/No 100) mg/g, respectively. Previous reports indicated that citrulline levels ranged from 2.37 to 2.85 g/kg fresh weight (~33.8 to 40.7 mg/g, adjusted assuming 93% water content in the flesh of watermelon fruit [2]) [8], 1.26 to 7.21 mg/g fresh weight (~18.0 to 103 mg/g dry weight) [6,23], 11.25 to 14.74 mg/g [27], 5.7 to 28.8 mg/g [5], and 15.7 to 43.81 mg/g [28], while arginine concentrations ranged between 8.23 to 11.10 mg/g [8] and 1.32 to 1.47g/kg fresh weight (~18.8 to 21.0 mg/g dry weight) [28]. These results are fairly in agreement with our report. All the genetic resources contained a higher level of citrulline compared to arginine, where citrulline was 1.27- to 7.68-fold higher. The highest combined concentrations of citrulline and arginine were detected in K192365 (S/No 82) and K192264 (S/No 75), which originated from Turkey and India, respectively, while the lowest concentration was detected in IT 190058 (S/No 12). Watermelon fruits that originated from Japan contained the highest average citrulline content followed by those from India. Indian originated fruits were superior in terms of the arginine content (see Figure 3 and Supplementary Material 2 (Figure S1) for photos). 

**Figure 4:**
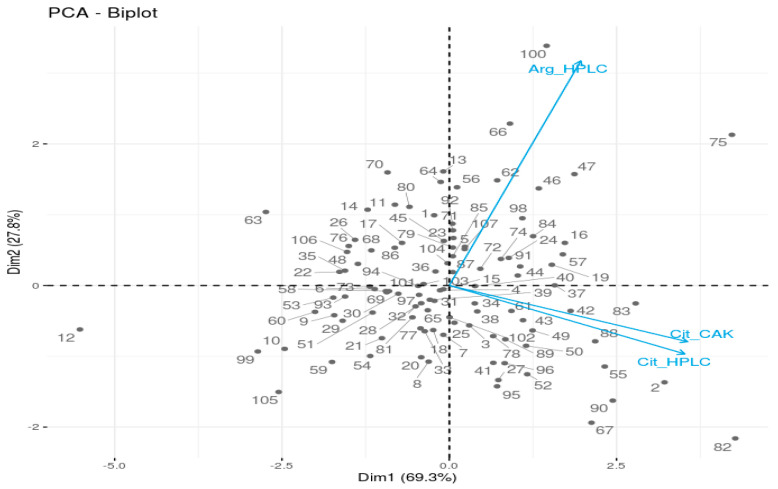
PCA-Biplot of watermelon fruit samples with citrulline and arginine as individual parameters (Arg_HPLC and Cit_HPLC represents arginine and citrulline contents determined using HPLC method); Cit_CAK is the citrulline content measured using the Citrulline Assay Kit method.

**Figure 5:**
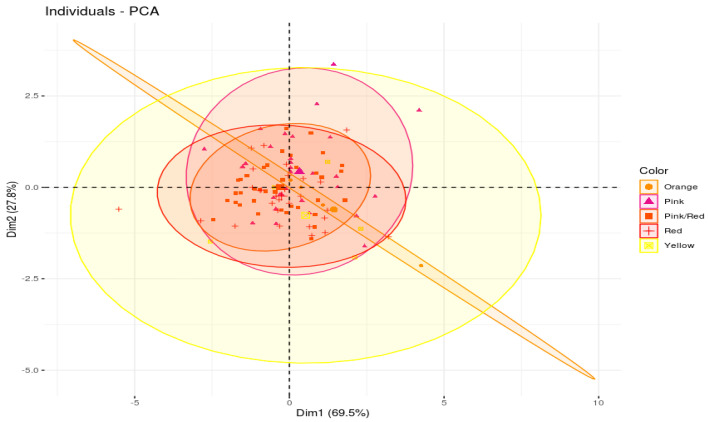
PCA-individual plot of watermelon fruit samples generated according to fruit flesh color.

In an attempt to group the genetic resources based on the levels of citrulline and arginine, and to examine the effect of flesh color on those phytonutrients, Principal Component Analysis (PCA) was conducted (Figure 4 and Figure 5; Supplementary Material 1, Table S5). The first two principal components contributed to 69.3 and 27.8% of the variation, with citrulline and arginine contributing the most for the first and second principal components, respectively. As can be seen in the scatter plots, the genetic resources were distributed homogeneously throughout the four quadrants with no significant groupings; however, some of the samples (S/No 12, 82, 100, 75) were seen to be well-separated from each other and from the entire population. This could be due to the exceptionally low levels of arginine and citrulline in S/No 12, high level of citrulline in S/No 82, high level of arginine in S/No 100, and high levels of arginine and citrulline in S/No 75. 

Most of the fruit morphological characteristics did not have an effect on the level of citrulline and arginine. The fruit width and length had a moderate negative effect on the citrulline level, with shorter and narrower accessions exhibiting higher citrulline levels. However, there was no significant correlation between arginine and fruit length and width. Fruit samples with shorter widths of stripes had a higher arginine level(Table 2). When averaged for all colors, orange (35.6 mg/g) and yellow (31.5 mg/g) colored flesh watermelon fruit samples contained slightly higher citrulline levels compared to pink (27.9 mg/g), pink/red (25.9 mg/g), and red (27.0 mg/g). However, arginine levels were not associated with the flesh color. Conflicting results were reported previously. Davis et al. (2011) [23] and Wehner et al. (2017) [26] reported the absence of association between citrulline levels and flesh color, whereas less citrulline content was detected in red-fleshed melons compared to yellow according to Rimando and Perkins-Veazie (2005) [5]. The SSC had a negative significant effect (*p* < 0.01) on both citrulline and arginine concentrations (Table 2). The negative correlations of citrulline, arginine, and citrulline + arginine with SSC observed in this study are concurrent with Hartman et al. (2019) [8], but contradict Wehner et al. (2017) [26]. A positive significant correlation (*p* < 0.01) was observed between citrulline and arginine, which could be because the former is the precursor of the latter.

The levels of citrulline and arginine in watermelon were reported to be affected by genotypes, cultivar, ploidy, flesh color, fruit anatomy, fruit ripeness, sample treatment methods, nitrogen status, salt tolerance, and environmental factors such as drought and light stress [5,7,8,10,31,32,33,34]. However, Davis et al. (2011) [23] ruled out the effect of some properties such as flesh color, fruit size and shape, skin color or pattern, fruit earliness, or soluble solids content on citrulline content in a study involving 56 watermelon cultivars, breeding lines, and PI accessions. These conflicting reports aside, the citrulline level in watermelon fruit is regulated by others factors such as genotypes, plant part, fruit development, nitrogen level, and stress. Previous reports indicated that watermelon fruit peel, an underutilized agricultural waste, is a rich source of citrulline compared to the edible flesh tissue, unlike arginine [7,28]. Citrulline is involved in maintaining the osmolarity of cells during abiotic stress, allowing watermelon to accumulate high levels of citrulline in response to oxidative stress caused by drastic conditions [10,33]. The accumulation of a higher level of citrulline in the peel could be due to the more stress it could experience relative to the central portion of the flesh and inner rinds. This could provide an insight into the source of variations of citrulline and arginine between different tissues. Transcriptomic analysis could help to understand the regulation of the genes associated with citrulline and arginine metabolism. To the best of our knowledge, no studies have been done to find out how genetic variability impacts the citrulline metabolism. In this study, the extraction and analysis of citrulline and arginine were conducted based on previously optimized methods for watermelon fruit [8,27,28], with some modifications such as extraction solvent. Citrulline and arginine could be better extracted using more environmentally benign solvents like water instead of organic solvents [28]. The nitrogen status of the plant regulates the citrulline level, where nitrogen deficiency causes a decrease in citrulline level [33]. The growth of watermelon in this study was maintained with an optimum addition of fertilizer as per the RDA’s agricultural management practices. Other factors that could impact the recovery of citrulline and arginine, such as sample size and particle size, were also optimized using citrulline to obtain the highest yield. As maturity impacts the levels of citrulline and arginine, watermelon fruits were harvested at full ripeness when their concentration peaks.

One way to improve watermelon fruit quality is through knowledge of the commulative effects of genotypic, physiological, and agroenvironmental factors. So far, signifcant advancements have been made on characterizing several factors of fruit quality. A study on a diverse genetic resources across the word could help to advance our current knowlege of watermelon fruit and its quality traits. Citrulline and arginine are important phytonutrients not only for the wellbeing of human health, but also for various metabolic processes taking place in plants, including watermelon. Since citrulline and arginine accumulation is affected by various factors, as discussed above, these compounds might be considered for assessing and screening watermelon genotypes as well as novel biochemical indicators in breeding programs. An increased citrulline and arginine content in watermelon could enhance tolerance of watermelon plants towards drought. Breeding could improve the levels of arginine in watermelon fruit, as both are heritable traits [26,35]. Because of the intensification of the demand for high yielding, diseases resistant, and highly nutritive food crops, including watermelon, the conservation and collection of genetically diverse genetic resources are essential activities for the maintenance and improvement of the current level of production. 

#### 3.2.3. Quantification of Citrulline Using Citrulline Assay Kit

The Citrulline Assay Kit (CAK) is a simple colorimetric assay that measures the amount of total citrulline present in biological samples such as cells, tissue, plasma serum, or urine samples [36,37,38]. Watermelon tissue sample extracts were treated with sodium dodecyl sulfate (SDS) and proteinase K to release free citrulline residues, followed by the addition of assay reagents (assay reagents A and B). The reaction of assay reagents with the released free citrulline at 95 °C produces chromophore that absorbs UV light at 540–560 nm. Studies on the citrulline levels of watermelon and other plant materials using a Citrulline Assay Kit are elusive. To the best of our knowledge, this is the first report on watermelon flesh samples. The citrulline content determined with this method ranged from 6.5 (S/No 12) to 42.8 (S/No 82) mg/g with a population mean of 27.0 mg/g (Table 3). The concentrations of citrulline measured using HPLC and the Citrulline Assay Kit showed a significant correlation with each other (*r*^2^ = 0.919; *p* < 0.01) and a 0.71- to 1.22-fold difference between each sample measurements among the two methods. The population means in the two methods (Citrulline Assay Kit and HPLC) are almost similar (27.0 and 27.3 mg/g, respectively). Colorimetric methods for the quantification of citrulline that is developed earlier [39] could overestimate the actual citrulline levels, and in thin layer chromatography and amino acid analyzers, the presence of glutamine could complicate quantification [6,23]. Other colorimetric citrulline analysis methods that have been mainly developed for animal and human tissues use strong oxidizers to hydrolyze tissues completely [40,41,42]. These strong sample treatments could cause the oxidation of citrulline in watermelon tissues and result in erroneously low values. The Citrulline Assay Kit method used in our study gave citrulline concentrations in the same general range as HPLC, which was adequate for estimating citrulline in order to compare a set of watermelon samples with another set of watermelon samples. The CAK is simple, efficient, and convenient to use for a large number of samples alternatively in the absence of other modern instruments such as HPLC.

## 4. Conclusions

Diverse characteristics were observed in terms of fruit morphological traits, citrulline, and arginine levels in watermelon germplasm collections. More than 90% of the genetic resources were either red- or pink-fleshed. Korean originated fruits contained intermediate levels of SSC while the USA, Russian, Tajikistan, Turkmenistan, Taiwan, and Uruguay originated fruits generally had the highest levels of soluble solids. Our results indicated that samples with high citrulline and arginine levels contained low soluble solid content, which was mainly comprised of sugar, making them less likable in terms of sweetness. Besides, red and pink colored flesh samples had less citrulline compared to yellow and orange. On the other hand, some of the primary quality traits consumers look for in a watermelon fruit include dark red flesh, high sugar content, and excellent flavor. This begs a breeding program that compromises these different quality traits with the health-beneficial compounds. In addition to the profiling of morphological characters and phytonutrients, molecular marker characterization and the identification of sources of resistance to diseases and pests are recommended for a more complete diversity analysis of watermelon genetic resources. As part of the effort in diversifying watermelon genetic resources, this study was devoted to providing baseline data on morphological quality traits and health beneficial phytonutrients of available watermelon germplasm collections in our gene bank, thereby identifying and incorporating new and quality resources into the already cultivated group.

## Figures and Tables

**Table 1:** Modified International Union for the Protection of New Varieties of Plants (UPOV) descriptors used for morphological characterization of watermelon fruits.

No	Trait	Description	Class	*n*	%	Min/Max/Ave *
1	Fruit shape in longitudinal section	Circular	1	63	60.6	-
		Broad elliptic	2	30	28.8	-
		Medium elliptic	3	9	8.7	-
		Narrow elliptic	4	2	1.9	-
2	Ground color of skin	White	1	6	5.7	-
		Yellow	2	0	0.0	-
		Green	3	99	94.3	-
3	Intensity of green color of skin	Very light	1	2	1.9	-
		Very light to light	2	5	4.8	-
		Light	3	3	2.9	-
		Light to medium	4	6	5.7	-
		Medium	5	18	17.1	-
		Medium to dark	6	21	20.0	-
		Dark	7	27	25.7	-
		Dark to very dark	8	13	12.4	-
		Very dark	9	10	9.5	-
4	Fruit shape of apical part	Truncate	1	52	49.5	-
		Little rounded	2	43	41.0	-
		Rounded	3	9	8.6	-
		Little acute	4	1	1.0	-
		Acute	5	0	0.0	-
5	Grooving distribution of fruit	Absent	1	85	81.0	-
		At basal half	2	1	1.0	-
		At apical half	3	0	0.0	-
		On whole fruit	4	19	18.1	-
6	Conspicuousness of stripes	Inconspicuous	0	35	33.3	-
		Conspicuous	1	70	66.7	-
7	Main color of flesh	White	1	0	0.0	-
		Yellow	2	5	4.8	-
		Orange	3	4	3.8	-
		Pink	4	30	28.6	-
		Pink/red	5	37	35.2	-
		Red	6	29	27.6	-
8	Size of pistil scar (mm)					2.0/32.6/14.1
9	Width of stripes (mm)					0.0/29.8/8.4
10	Weight of fruit (kg)					3.2/10.4/5.2
11	Length of fruit (cm)					17.0/48.4/23.3
12	Width of fruit(cm)					17.3/23.2/20.7
13	Thickness of outer layer of the pericarp (mm)					4.6/22.6/10.6
14	Soluble solids content (Brix)					5.1/12.3/8.5

* Average of 10 to 12 fruits; *n* = number of accessions/varieties; Min = minimum, Max = maximum, Ave = Average.

**Table 2:** Pearson’s correlation coefficient of traits for watermelon genetic resources.

Trait	SPS	WS	WF	LF	WIF	TP	SSC	Citrulline	Arginine	Citrulline ^#^
WS	0.020	-								
WF	−0.362 **	0.144	-							
LF	−0.662 **	0.052	0.737 **	-						
WIF	0.351 **	0.156	0.439 **	-0.204 *	-					
TP	0.094	0.197 *	0.174	0.191	0.146	-				
SSC	−0.013	0.142	−0.026	−0.094	0.046	−0.419 **	-			
Citrulline	0.191	0.014	−0.235 *	−0.203 *	−0.161	−0.083	−0.375 **	-		
Arginine	0.043	−0.289 **	0.072	0.084	-0.052	0.120	−0.375 **	0.283 **	-	
Citrulline ^#^	0.116	0.004	-0.223 *	−0.150	−0.218 *	−0.041	−0.351 **	0.919 **	0.318 **	-
Citrulline + Arginine	0.172	−0.103	−0.166	−0.133	−0.152	−0.021	−0.452	0.926	0.624	0.874

** Correlation is significant at the 0.01 level (2-tailed); * Correlation is significant at the 0.05 level (2-tailed); SPS = Size of pistil scar (mm); WS = Width of stripes (mm); WF = Weight of fruit (kg); LF = Length of fruit (cm); WIF = Width of fruit (cm); TP = Thickness of outer layer of pericarp (mm); SSC = Soluble solids content (Brix); ^#^ Citrulline content are determined using Citrulline Assay Kit (CAK).

**Table 3:** Citrulline and arginine contents (mg/g DW) in extracts of watermelon flesh samples using HPLC and the Citrulline Assay Kit.

S/No	Origin	IT NO/ Variety Name	Citrulline (HPLC)	Arginine (HPLC)	Citrulline (CAK)
1	KOR	IT104713	22.85 ± 1.67 i-s	12.37 ± 0.48 c-m	26.39 ± 0.69 e-r
2	TUR	IT119709	44.30 ± 0.49 ab	9.51 ± 0.27 e-q	39.54 ± 0.54 a-c
3	ITA	IT119743	30.03 ± 0.06 c-r	8.51 ± 0.16 f-r	28.46 ± 0.70 c-q
4	TWN	IT120006	28.52 ± 2.01 c-r	9.48 ± 0.26 e-q	25.23 ± 0.35 f-r
5	TWN	IT120008	27.16 ± 0.48 d-s	11.64 ± 0.18 c-n	27.29 ± 0.30 e-q
6	Unknown	IT138188	22.26 ± 0.39 i-s	8.37 ± 0.18 f-r	23.46 ± 0.45 h-r
7	CHN	IT160392	26.04 ± 0.59 e-s	7.62 ± 0.32 h-s	29.42 ± 0.24 c-q
8	Unknown	IT174803	28.62 ± 0.51 c-r	6.34 ± 0.09 m-s	26.75 ± 0.20 e-r
9	Unknown	IT185447	20.39 ± 0.42 l-s	6.61 ± 0.36 m-s	21.76 ± 0.15 j-r
10	Unknown	IT185456	18.10 ± 0.17 p-t	4.39 ± 0.34 p-s	19.72 ± 0.20 o-r
11	USA	IT190057	21.92 ± 0.63 j-s	12.18 ± 0.12 c-m	22.57 ± 0.25 h-r
12	USA	IT190058	6.90 ± 0.12 t	1.76 ± 0.19 s	6.54 ± 0.28 s
13	UKR	IT190059	25.22 ± 0.53 g-s	14.39 ± 0.33 c-g	23.64 ± 0.30 h-r
14	TKM	IT190077	21.52 ± 0.26 j-s	11.54 ± 0.31 c-n	20.19 ± 0.11 n-r
15	KGZ	IT190084	29.17 ± 1.30 c-r	9.59 ± 0.3 e-q	24.99 ± 0.59 g-r
16	TJK	IT190110	33.45 ± 0.98 b-n	13.50 ± 0.43 c-j	32.51 ± 0.16 a-j
17	TJK	IT190116	23.51 ± 0.17 i-s	10.74 ± 0.18 d-p	23.50 ± 0.51 h-r
18	KAZ	IT190123	25.96 ± 0.43 e-s	7.66 ± 0.22 h-s	28.22 ± 0.06 d-q
19	TKM	IT190135	33.46 ± 1.29 b-n	12.39 ± 0.18 c-m	31.98 ± 0.72 b-l
20	BRA	IT190141	26.63 ± 0.73 e-s	6.36 ± 0.1 m-s	27.45 ± 0.20 d-q
21	RUS	IT190146	23.54 ± 0.66 i-s	6.47 ± 0.19 l-s	25.10 ± 0.69 g-r
22	RUS	IT190148	19.31 ± 0.51o-s	8.47 ± 0.08 f-r	21.51 ± 0.47 j-r
23	Unknown	IT190151	26.54 ± 0.76 e-s	11.81 ± 0.14 c-n	26.24 ± 0.39 e-r
24	RUS	IT199769	31.47 ± 0.24 b-p	11.96 ± 0.04 c-n	28.79 ± 0.50 c-q
25	RUS	IT199772	29.22 ± 0.58 c-r	8.37 ± 0.18 f-r	27.45 ± 0.32 d-q
26	RUS	IT199773	20.57 ± 0.57 l-s	10.07 ± 0.19 d-q	20.84 ± 0.10 l-r
27	RUS	IT199788	33.00 ± 0.38 b-o	6.76 ± 0.18 k-s	31.23 ± 0.51 c-n
28	RUS	IT199796	24.75 ± 0.75 g-s	8.56 ± 0.2 f-r	26.89 ± 0.45 e-r
29	UKR	IT199805	20.76 ± 0.41 k-s	6.52 ± 0.31 l-s	22.56 ± 0.38 h-r
30	UKR	IT199806	21.80 ± 0.80 j-s	8.53 ± 0.33 f-r	26.55 ± 0.36 e-r
31	KAZ	IT199814	25.83 ± 0.43 f-s	8.89 ± 0.18 e-r	27.30 ± 0.17e-q
32	UZB	IT199823	24.65 ± 0.48 g-s	8.34 ± 0.05 f-r	26.50 ± 0.32 e-r
33	NPL	IT200493	28.08 ± 0.15 c-r	7.53 ± 0.24 h-s	25.58 ± 0.52 e-r
34	PHL	IT201722	29.63 ± 0.51 c-r	9.50 ± 0.36 e-q	28.51 ± 0.39 c-q
35	UZB	IT202998	19.65 ± 0.20 n-s	8.61 ± 0.12 f-r	21.76 ± 0.48 j-r
36	RUS	IT203017	26.38 ± 0.30 e-s	10.14 ± 0.12 d-q	25.79 ± 0.08 e-r
37	RUS	IT203019	34.73 ± 0.62 b-k	11.61 ± 0.41 c-n	32.04 ± 0.63 b-l
38	KAZ	IT203029	24.01 ± 0.52 i-s	9.07 ± 0.05 e-q	33.81 ± 0.08 a-h
39	UZB	IT203034	26.09 ± 1.55 e-s	8.87 ± 0.03 e-r	26.55 ± 0.91 e-r
40	RUS	IT203037	30.32 ± 0.49 c-q	10.24 ± 0.11 d-q	27.28 ± 0.20 e-q
41	UZB	IT203049	31.44 ± 2.27 b-p	7.36 ± 0.37 i-s	31.32 ± 0.29 c-n
42	UZB	IT203067	34.11 ± 2.50 b-l	10.78 ± 0.11 d-p	35.26 ± 0.26 a-g
43	AZE	IT203072	31.82 ± 3.32 b-p	9.60 ± 0.07 e-q	32.48 ± 0.38 a-k
44	Unknown	IT203627	32.79 ± 0.45 b-o	11.40 ± 0.35 c-n	29.34 ± 0.19 c-q
45	MNG	IT204167	25.98 ± 1.18 e-s	11.53 ± 0.11 c-n	25.81 ± 0.58 e-r
46	Unknown	IT208441	30.10 ± 1.30 c-r	15.28 ± 0.3bcde	30.39 ± 0.51 c-p
47	UZB	IT213903	33.22 ± 1.88 b-o	16.50 ± 0.18abcd	31.01 ± 0.24 c-o
48	Unknown	IT216860	21.20 ± 2.83 j-s	9.13 ± 0.36 e-q	21.57 ± 0.24 j-r
49	Unknown	IT251845	33.38 ± 1.62 b-n	9.36 ± 0.48 e-q	32.59 ± 0.39 a-j
50	Unknown	IT251849	34.91 ± 1.48 b-j	8.66 ± 0.31 f-r	31.23 ± 0.52 c-n
51	Unknown	IT251851	23.08 ± 0.93 i-s	7.38 ± 0.14 i-s	23.48 ± 0.34 h-r
52	RUS	IT251860	35.95 ± 2.25 b-i	7.52 ± 0.12 h-s	31.60 ± 0.36 b-m
53	USA	IT271064	20.89 ± 1.09 k-s	7.34 ± 0.18 j-s	20.54 ± 0.09 m-r
54	CHN	IT294452	24.85 ± 1.61 g-s	5.58 ± 0.36 n-s	23.46 ± 0.56 h-r
55	JPN	IT302244	39.30 ± 0.84 b-f	9.12 ± 0.22 e-q	36.74 ± 0.54 a-e
56	UZB	IT305108	26.65 ± 1.63 e-s	13.98 ± 0.05 c-h	24.53 ± 0.19 g-r
57	RUS	IT321060	32.52 ± 1.86 b-o	12.97 ± 0.15 c-l	33.53 ± 0.39 a-i
58	UZB	IT321075	24.79 ± 1.53 g-s	8.52 ± 0.10 f-r	22.61 ± 0.49 h-r
59	Unknown	IT32839	23.09 ± 0.45 i-s	4.70 ± 0.21 o-s	21.11 ± 0.62 k-r
60	URY	IT119741	16.94 ± 0.42 q-t	6.37 ± 0.23 m-s	22.47 ± 0.71 h-r
61	RUS	IT199776	31.57 ± 2.04 b-p	9.80 ± 0.03 e-q	31.09 ± 0.37 c-o
62	RUS	IT199783	27.11 ± 3.47 d-s	14.90 ± 0.08 c-f	28.15 ± 0.32 d-q
63	UKR	IT199804	14.00 ± 0.22 st	9.69 ± 0.19 e-q	15.75 ± 0.20 r
64	USA	IT199834	25.91 ± 0.98 f-s	13.93 ± 0.26 c-i	23.2 ± 0.52 h-r
65	CHN	803617	26.82 ± 0.70 d-s	8.42 ± 0.18 f-r	26.13 ± 0.54 e-r
66	RUS	805656	28.20 ± 0.60 c-r	17.48 ± 0.21 a-c	26.35 ± 0.34 e-r
67	JPN	807364	41.38 ± 2.12 a-c	6.63 ± 0.39 l-s	35.81 ± 1.00 a-g
68	KOR	906976	22.90 ± 0.90 i-s	9.94 ± 0.02 e-q	21.04 ± 0.44 l-r
69	Unknown	908581	24.79 ± 0.93 g-s	8.48 ± 0.11 f-r	22.75 ± 0.58 h-r
70	UZB	908835	22.02 ± 1.38 i-s	13.41 ± 0.11 c-j	20.42 ± 0.29 m-r
71	Unknown	K004668	26.78 ± 1.18 d-s	12.13 ± 0.08 c-n	25.69 ± 0.40 e-r
72	USA	K012424	28.67 ± 1.14 c-r	11.00 ± 0.29 d-o	28.54 ± 0.26 c-q
73	KOR	K038117	24.05 ± 0.67 i-s	8.44 ± 0.12 f-r	21.33 ± 0.46 j-r
74	IND	K192260	29.26 ± 1.62 c-r	11.74 ± 0.14 c-n	29.85 ± 0.36 c-q
75	IND	K192264	38.19 ± 0.67 b-h	20.69 ± 0.23 ab	42.06 ± 0.29 ab
76	TUR	K192296	19.91 ± 0.54 m-s	9.70 ± 0.15 e-q	20.97 ± 0.31 l-r
77	TUR	K192319	26.98 ± 0.64 d-s	7.56 ± 0.21 h-s	25.94 ± 0.52 e-r
78	TUR	K192321	30.68 ± 0.70 c-q	8.45 ± 0.35 f-r	30.89 ± 0.74 c-p
79	TUR	K192324	25.02 ± 0.68 g-s	11.37 ± 0.23c-n	27.83 ± 0.82 d-q
80	TUR	K192338	22.53 ± 0.54 i-s	12.33 ± 0.15 c-m	23.69 ± 0.62 h-r
81	TUR	K192352	26.08 ± 0.38 e-s	7.87 ± 0.19 g-s	25.40 ± 0.25 f-r
82	TUR	K192365	52.06 ± 0.59 a	8.46 ± 0.20 f-r	42.79 ± 0.48 a
83	TUR	K192370	40.53 ± 0.51 b-d	12.25 ± 0.33 c-m	36.51 ± 0.46 a-f
84	TUR	K192373	32.36 ± 0.34 b-o	13.26 ± 0.30 c-k	29.78 ± 0.29 c-q
85	TUR	K192378	28.84 ± 0.30 c-r	11.10 ± 0.07 c-o	24.97 ± 0.66 g-r
86	TUR	K192379	24.51 ± 1.04 h-s	10.45 ± 0.15 d-p	22.08 ± 0.23 j-r
87	TUR	K192381	28.33 ± 0.47 c-r	10.43 ± 0.18 d-p	26.02 ± 0.18 e-r
88	TUR	K192386	38.42 ± 0.86 b-g	10.00 ± 0.18 e-q	35.47 ± 0.30 a-g
89	TUR	K192390	33.35 ± 0.58 b-n	8.57 ± 0.20 f-r	30.07 ± 0.61 c-p
90	TUR	K192394	39.76 ± 0.87 b-e	7.84 ± 0.13 g-s	38.56 ± 0.28 a-d
91	TUR	K192397	33.05 ± 0.09 b-o	11.82 ± 0.04 c-n	29.02 ± 0.09 c-q
92	TUR	K192403	26.51 ± 0.84 e-s	12.39 ± 0.36 c-m	25.60 ± 0.52 e-r
93	TUR	K192432	19.80 ± 0.53 n-s	7.54 ± 0.24 h-s	22.67 ± 0.45 h-r
94	TUR	K192444	25.04 ± 0.54 g-s	9.24 ± 0.06 e-q	25.64 ± 0.27 e-r
95	TUR	K192446	33.79 ± 0.36 b-m	6.50 ± 0.35 l-s	30.68 ± 0.36 c-p
96	TUR	K192467	33.03 ± 0.42 b-o	7.56 ± 0.33 h-s	31.21 ± 0.60 c-n
97	TUR	K192469	25.41 ± 0.45 g-s	8.89 ± 0.22 e-r	25.78 ± 0.22 e-r
98	IRQ	K192471	31.14 ± 0.46 b-p	13.81 ± 0.26 c-j	28.95 ± 0.94 c-q
99	TUR	K192502	16.19 ± 0.61 r-t	3.82 ± 0.25 q-s	18.57 ± 0.38 qr
100	TUR	K192504	26.91 ± 0.34 d-s	21.25 ± 0.11 a	28.26 ± 0.51 d-q
101	KOR	Speedggul	22.07 ± 0.78 i-s	8.50 ± 0.19 f-r	25.23 ± 0.40 f-r
102	KOR	Sambokggul	25.70 ± 0.23 f-s	8.44 ± 0.15 f-r	29.67 ± 0.22 c-q
103	KOR	Seo Tae Ja	24.58 ± 0.86 g-s	9.39 ± 0.31 e-q	26.53 ± 0.33 e-r
104	KOR	Uriggul	23.88 ± 0.47 i-s	10.61 ± 0.35 d-p	28.85 ± 0.52 c-q
105	KOR	Newkkokkoma	19.50 ± 0.43 n-s	2.54 ± 0.03 rs	19.62 ± 0.34 p-r
106	KOR	Lycofreshi	18.33 ± 0.31 p-t	9.39 ± 0.13 e-q	22.35 ± 0.35 i-r
107	KOR	Norangsambokggul	23.85 ± 0.95 i-s	11.46 ± 0.2 c-n	30.13 ± 0.56 c-p

S/No = Sample identification number; Different letters between rows indicate statistically significant differences between watermelon genetic resources at *p* < 0.05.

## Supplementary Materials

The following are available online at https://www.mdpi.com/2223-7747/9/9/1054/s1, Figure S1: Photos of watermelon fruit germplasm collections with wide phenotypic diversity, Table S1: Accession numbers, origin, and some selected morphological characters of the genetic resources, Figure S2: The effect of sample size (sample to solvent ratio) on instrument response of citrulline levels evaluated in two watermelon fruit samples: HPLC (a and b); CAK (c and d), Table S2: Results of the principal component analyses (for the first three PC’s) of seven quantitative morphological traits for the investigated watermelon fruit samples, Table S3: Recovery test results of HPLC and Citrulline Assay Kit methods using two watermelon fruit samples, Table S4: Inter-and intra-day precision results of HPLC and Citrulline Assay kit methods using two representative watermelon fruit samples, Table S5: Results of the first three principle components of citrulline and arginine for investigated watermelon fruit samples.
