# Supplementary material for: Fruit Morphology, Citrulline, and Arginine Levels in Diverse Watermelon (Citrullus lanatus) Germplasm Collections
Source: Plants (Basel). 2020 Aug 19;9(9):1054. doi: 10.3390/plants9091054 (PMC7569901; doi:10.3390/plants9091054)
Supplement: Supplementary file 1 [file plants-09-01054-s001.zip › Supplementary Material 1.pdf]

Fruit morphology, citrulline, and arginine levels in diverse watermelon (*Citrullus lanatus*) germplasm collections

Awraris Derbie Assefa[1], On-Sook Hur[1], Na-Young Ro[1], Jae-Eun Lee[1], Ae-Jin Hwang[1], Bich-Saem Kim[1], Ju-Hee Rhee[1], Jung Yoon Yi[1], Ji Hyun Kim[1], Ho-Sun Lee[2], Jung-Sook Sung[3], Myung-Kon Kim[4], Jae-Jong Noh[5]*

[1]National Agrobiodiversity Center, National Institute of Agricultural Sciences, RDA, Jeonju 54874, Rep. of Korea

[2]International Technology Cooperation Center, RDA, Jeonju 54875, Rep. of Korea

[3]Upland Crop Breeding Division, Department of Southern Area Crop Science, National Institute of Crop Science, RDA, Miryang 50424, Rep. of Korea

[4]Department of Food Science and Technology, Jeonbuk National University, Jeonju 54896, Rep. of Korea

[5]Jeonbuk Agricultural Research and Extension Services, Iksan 54591, Rep. of Korea

*Corresponding author: Email nohjj@korea.kr; Tel. +82-63-290-6121

Figure S1 Photos of watermelon fruit germplasm collections with wide phenotypic diversity [Photos are submitted in a separate pdf file, Supplementary material 2]

Table S1: Accession numbers, origin, and some selected morphological characters of the genetic resources

S/No	IT NO/Variety name	Origin	Traits*													
			A	B	C	D	E	F	G	H	I	J	K	L	M	N
1	IT104713	KOR	3	3	5	2	1	0	5	2.0	0.0	6.3	33.0	19.1	9.8	8.8
2	IT119709	TUR	1	3	4	1	4	0	6	25.6	0.0	3.2	17.0	18.6	7.4	8.8
3	IT119743	ITA	2	3	6	3	1	1	5	11.4	26.0	5.5	22.5	21.3	8.2	9.5
4	IT120006	TWN	1	3	9	2	1	1	6	10.4	9.4	4.3	21.0	19.0	10.8	10.2
5	IT120008	TWN	2	3	5	2	1	1	5	4.6	9.8	5.1	25.0	19.3	8.6	10.3
6	IT138188	Unknown	3	3	5	2	1	0	5	3.0	0.0	6.2	31.8	19.1	9.2	9.2
7	IT160392	CHN	2	3	3	2	1	0	5	2.6	0.0	6.3	29.9	19.9	10.2	10.2
8	IT174803	Unknown	1	3	6	3	1	1	6	10.0	11.2	5.7	22.3	21.7	11.8	9.2
9	IT185447	Unknown	2	3	5	2	1	1	5	10.6	26.4	6.6	24.0	22.7	10.4	10.4
10	IT185456	Unknown	1	3	5	3	1	1	5	10.6	8.2	5.1	22.9	20.7	9.4	8.6
11	IT190057	USA	2	3	8	3	1	0	6	5.4	0.0	7.0	26.0	22.7	11.0	9.0
12	IT190058	USA	2	3	4	1	1	0	6	11.8	0.0	6.8	25.3	22.6	11.2	9.8
13	IT190059	UKR	2	1	1	1	1	0	5	32.6	0.0	5.6	21.9	22.3	7.6	8.8
14	IT190077	TKM	2	3	8	1	1	0	6	26.0	0.0	5.7	20.8	22.1	8.8	10.9
15	IT190084	KGZ	2	3	2	1	4	0	4	17.6	0.0	5.1	21.3	20.8	8.2	9.9
16	IT190110	TJK	1	3	5	1	1	1	5	24.0	5.6	5.7	21.7	22.3	10.0	7.8
17	IT190116	TJK	1	3	6	2	1	1	5	8.8	7.8	5.3	23.8	21.2	14.6	6.7
18	IT190123	KAZ	1	3	7	1	1	1	5	20.0	15.0	3.6	17.8	19.2	9.0	8.5
19	IT190135	TKM	2	3	6	1	1	1	4	19.8	11.2	5.7	22.6	21.6	11.4	7.6
20	IT190141	BRA	3	3	5	2	1	1	4	2.4	23.6	6.8	34.3	19.2	10.8	8.8
21	IT190146	RUS	1	3	7	1	1	1	5	23.4	19.4	5.9	22.2	22.2	10.0	10.4
22	IT190148	RUS	1	1	2	1	4	0	5	19.0	0.0	5.4	22.4	21.3	10.4	9.5
23	IT190151	Unknown	4	3	5	2	1	0	4	2.2	0.0	7.6	38.0	19.1	9.0	8.2
24	IT199769	RUS	1	3	9	1	4	0	5	19.4	0.0	5.2	20.5	21.8	11.4	9.3
25	IT199772	RUS	1	3	6	1	4	1	5	17.8	27.2	5.7	22.6	22.2	12.6	9.9
26	IT199773	RUS	1	3	8	1	4	1	4	17.2	12.4	5.0	21.8	21.1	14.0	9.1
27	IT199788	RUS	1	3	7	1	1	1	6	19.6	24.0	4.4	20.6	20.3	14.2	10.0
28	IT199796	RUS	1	3	9	1	1	0	6	23.0	0.0	4.8	20.8	21.4	9.4	8.7
29	IT199805	UKR	1	3	6	1	1	1	5	15.6	21.0	5.8	22.5	22.3	11.6	8.5

30	IT199806	UKR	1	1	2	1	4	0	5	20.6	0.0	5.1	21.7	20.6	9.4	10.6
31	IT199814	KAZ	2	3	6	1	4	1	4	17.0	4.8	4.5	20.8	18.3	12.8	7.6
32	IT199823	UZB	2	3	9	2	1	0	4	4.2	0.0	7.0	27.1	21.5	11.4	8.1
33	IT200493	NPL	1	3	9	2	1	1	6	10.6	6.4	5.5	23.1	21.1	11.2	9.5
34	IT201722	PHL	1	3	9	1	1	1	6	11.0	9.4	4.9	22.0	20.5	8.8	8.6
35	IT202998	UZB	1	3	7	1	1	1	5	20.8	14.0	4.8	20.7	20.3	10.4	7.5
36	IT203017	RUS	1	3	7	1	1	1	5	27.4	29.8	5.1	21.3	21.3	14.6	8.5
37	IT203019	RUS	1	3	7	2	1	1	4	26.4	19.8	5.1	21.4	21.7	10.6	7.9
38	IT203029	KAZ	2	3	6	1	1	1	4	17.4	8.6	4.4	21.2	20.6	10.8	8.7
39	IT203034	UZB	2	3	6	2	4	1	4	13.6	6.4	4.8	22.1	20.6	11.2	8.5
40	IT203037	RUS	1	3	5	1	1	1	3	21.4	5.2	4.5	20.7	20.4	10.2	8.4
41	IT203049	UZB	1	3	6	2	2	1	6	11.0	11.2	4.7	22.2	20.0	11.4	7.8
42	IT203067	UZB	1	3	7	2	1	1	5	8.0	10.6	4.7	21.8	20.6	9.8	7.1
43	IT203072	AZE	1	3	7	1	1	1	3	22.4	7.2	3.9	18.9	19.7	13.6	8.5
44	IT203627	Unknown	1	3	9	1	1	1	6	11.4	3.4	5.3	22.2	21.2	7.8	7.8
45	IT204167	MNG	1	3	7	2	1	0	6	16.4	0.0	4.2	20.6	19.8	11.2	9.0
46	IT208441	Unknown	3	3	5	3	1	0	4	2.6	0.0	6.4	32.0	19.4	9.2	7.5
47	IT213903	UZB	1	3	9	1	1	0	6	20.0	0.0	6.1	22.7	22.2	9.0	8.6
48	IT216860	Unknown	3	3	5	2	1	0	5	2.2	0.0	5.6	31.6	17.8	9.4	6.5
49	IT251845	Unknown	2	3	7	2	1	1	6	7.4	11.8	4.5	23.2	19.4	10.8	7.8
50	IT251849	Unknown	1	3	6	2	1	1	6	15.4	10.2	5.0	20.5	21.6	8.0	8.5
51	IT251851	Unknown	1	3	7	2	1	1	5	9.0	13.2	5.4	22.3	21.6	8.0	9.0
52	IT251860	RUS	2	3	7	1	1	1	6	20.4	6.0	4.6	20.4	21.0	9.6	7.3
53	IT271064	USA	1	3	6	2	1	1	5	11.2	21.8	6.9	24.8	23.2	8.4	9.2
54	IT294452	CHN	3	3	5	2	1	0	4	5.6	0.0	6.5	32.4	19.9	7.6	8.4
55	IT302244	JPN	1	3	7	3	1	1	2	8.8	9.8	3.8	20.1	18.6	4.6	9.0
56	IT305108	UZB	2	3	3	2	1	0	4	10.0	0.0	5.7	24.5	22.0	13.4	7.3
57	IT321060	RUS	1	1	1	1	1	0	5	16.3	0.0	4.9	20.4	20.6	8.5	9.3
58	IT321075	UZB	1	3	6	1	1	1	5	23.4	13.4	5.5	21.5	21.8	12.4	8.1
59	IT32839	Unknown	1	3	6	3	1	1	6	6.6	13.2	5.0	22.0	21.1	9.2	9.5
60	IT119741	URY	2	3	8	2	1	0	5	8.0	0.0	5.9	24.3	21.7	10.0	9.8
61	IT199776	RUS	1	3	7	1	1	1	5	20.0	14.4	6.0	22.0	22.3	10.6	9.7
62	IT199783	RUS	1	3	8	1	1	0	5	17.2	0.0	5.2	22.2	21.2	10.8	8.7
63	IT199804	UKR	2	3	6	1	1	1	4	17.4	11.4	5.1	21.7	20.4	10.4	9.3
64	IT199834	USA	2	3	7	3	1	0	4	4.0	0.0	6.3	26.4	21.4	9.8	8.1

65	803617	CHN	3	3	7	4	1	1	6	2.0	24.4	4.4	29.4	17.3	10.8	9.9
66	805656	RUS	2	3	9	2	1	0	4	12.2	0.0	4.9	21.6	20.9	10.4	7.3
67	807364	JPN	1	3	7	1	1	1	2	20.8	12.6	5.0	21.8	21.2	9.0	10.0
68	906976	KOR	1	3	7	2	1	1	6	10.0	16.2	5.1	22.2	21.0	8.2	8.9
69	908581	Unknown	1	3	8	2	1	1	6	11.0	10.2	5.4	22.4	21.1	10.2	8.7
70	908835	UZB	1	3	7	1	1	1	4	21.0	23.8	4.9	22.1	20.5	10.4	8.8
71	K004668	Unknown	2	3	8	3	1	0	4	4.4	0.0	5.4	26.4	19.9	6.8	7.0
72	K012424	USA	2	3	7	2	1	1	6	11.6	19.4	7.2	25.4	22.8	10.6	9.0
73	K038117	KOR	1	3	8	2	1	1	6	11.2	17.2	6.3	23.6	22.2	9.2	9.1
74	K192260	IND	4	3	7	2	1	1	4	2.2	28.0	10.4	48.4	20.2	22.6	5.5
75	K192264	IND	1	3	6	2	4	1	4	9.0	7.6	7.3	25.3	22.7	15.0	5.4
76	K192296	TUR	2	3	8	1	1	0	4	13.0	0.0	4.6	22.6	20.4	11.6	8.5
77	K192319	TUR	1	3	8	2	1	1	4	12.4	10.4	4.3	20.7	20.8	11.2	7.8
78	K192321	TUR	2	3	5	2	1	1	4	15.6	3.6	4.0	20.3	20.8	11.8	7.0
79	K192324	TUR	1	3	9	1	1	1	4	20.8	0.0	4.7	20.6	21.0	11.6	7.1
80	K192338	TUR	2	3	7	1	4	1	4	15.6	13.6	4.3	21.2	20.5	13.4	7.2
81	K192352	TUR	1	3	6	1	4	1	6	15.6	3.6	5.3	22.8	21.0	7.8	7.4
82	K192365	TUR	1	3	7	1	4	0	3	18.4	0.0	3.7	19.1	18.7	5.6	5.7
83	K192370	TUR	1	3	4	1	1	1	4	15.4	9.0	4.3	21.0	19.8	11.4	5.7
84	K192373	TUR	1	3	8	2	4	0	2	23.4	0.0	5.8	21.9	21.9	11.0	5.8
85	K192378	TUR	3	3	7	1	1	0	4	6.4	0.0	4.0	25.4	17.8	11.6	7.7
86	K192379	TUR	1	3	5	1	1	1	5	23.8	6.4	4.3	21.0	20.6	11.2	8.5
87	K192381	TUR	1	3	5	1	1	1	3	24.8	13.2	5.9	21.9	22.7	11.2	7.1
88	K192386	TUR	1	3	6	1	1	1	4	20.8	12.4	4.7	21.3	20.7	10.2	8.0
89	K192390	TUR	1	3	6	2	1	1	5	13.4	13.0	5.8	23.1	22.8	10.8	8.6
90	K192394	TUR	2	3	4	1	1	1	4	12.8	14.0	4.1	19.6	20.2	15.6	5.1
91	K192397	TUR	2	3	5	1	4	1	5	19.0	4.8	4.0	21.0	20.3	12.6	8.0
92	K192403	TUR	1	3	5	2	1	1	5	18.6	5.0	5.2	20.7	21.8	11.4	7.7
93	K192432	TUR	1	3	7	1	4	1	5	15.4	10.6	3.7	20.6	19.2	12.6	8.0
94	K192444	TUR	1	3	8	1	1	0	2	17.2	0.0	4.9	20.4	21.6	14.2	6.7
95	K192446	TUR	1	3	4	1	1	1	5	21.2	13.2	3.9	20.9	20.4	11.4	8.7
96	K192467	TUR	1	3	4	1	1	1	5	17.0	8.8	4.9	22.1	20.5	11.6	8.7
97	K192469	TUR	1	1	2	2	4	0	5	16.6	0.0	4.7	21.1	20.9	12.4	9.0
98	K192471	IRQ	1	1	2	1	4	0	5	19.6	0.0	4.3	20.5	19.7	10.6	9.0
99	K192502	TUR	1	3	5	1	4	1	6	23.0	3.8	3.8	21.2	22.2	17.4	8.6

100	K192504	TUR	3	3	8	2	1	0	4	6.6	0.0	3.9	27.1	18.0	15.6	6.6
101	Speedggul	KOR	1	3	7	2	1	1	6	2.4	11.4	4.5	22.6	19.5	6.0	10.9
102	Sambokggul	KOR	2	3	6	2	1	1	6	5.2	12.2	4.5	22.7	19.6	7.4	9.7
103	Seo Tae Ja	KOR	2	3	7	2	1	1	6	5.8	14.4	5.2	25.6	21.0	8.0	9.3
104	Uriggul	KOR	-	3	3	2	1	1	6	11.4	5.6	3.8	23.7	17.7	4.6	12.3
105	Newkkokkoma	KOR	1	3	6	2	1	1	2	8.0	12.6	5.9	25.2	21.5	9.2	10.1
106	Lycofresh	KOR	-	-	-	-	-	-	-	-	-	-	-	-	-	-
107	Norangsambokggul	KOR	-	-	-	-	-	-	-	-	-	-	-	-	-	-

*The ranking of the traits (A-G) are described in Table 1; A = Fruit shape in longitudinal section; B = Ground color of skin; C =Intensity of green color of skin; D = Fruit shape of apical part; E = Grooving distribution of fruit; F = Conspicuousness of stripes; G = Main color of flesh; H = Size of pistil scar (mm); I = Width of stripes (mm); J = Weight of fruit (kg); K = Length of fruit (cm); L = Width of fruit(cm); M = Thickness of the outer layer of the pericarp (mm); N =Soluble solids content (Brix)

Figure S2: The effect of sample size (sample to solvent ratio) on instrument response of citrulline levels evaluated in two watermelon fruit samples: HPLC (a and b); CAK (c and d)

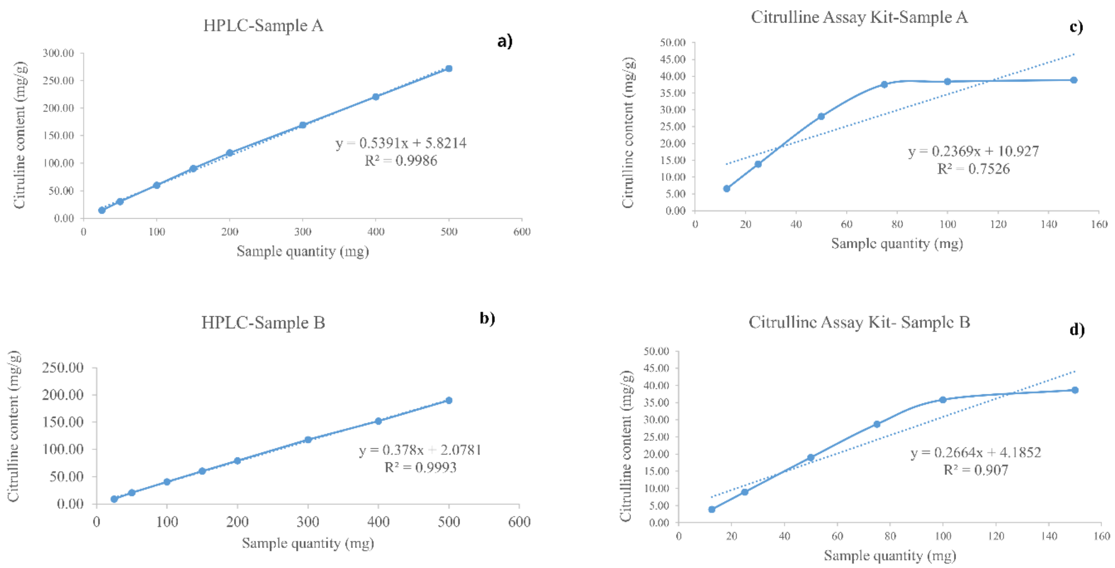

Table S2: Results of the principal component analyses (for the first three PC's) of seven quantitative morphological traits for the investigated watermelon fruit samples

Trait	PC1	PC2	PC3
Size of pistil scar (mm)	-0.487	0.4081	-0.02721
Width of stripes (mm)	0.09858	0.3191	0.2888
Weight of fruit (kg)	0.5529	0.2643	0.2527
Length of fruit (cm)	0.6326	-0.1238	-0.01731
Width of fruit(cm)	-0.01581	0.6228	0.3364
Thickness of the outer layer of the pericarp (mm)	0.1882	0.4694	-0.4651
Soluble solid content (Brix)	-0.1075	-0.1954	0.7226
Eigen value	2.23957	1.63202	1.34156
%Variance	31.9938921	23.3146356	19.1650975

Table S3: Recovery test results of HPLC and Citrulline Assay Kit methods using two watermelon fruit samples

Method	Compound	Sample	Original content (mg/g DW)	Spiked amount (mg)	Quantity after spiking (mg/g DW)	Recovery (%)
HPLC	Citrulline	Sample A	30.07±0.65	2	32.29±0.21	100.70±1.42
				5	35.96±0.50	102.55±0.74
				10	42.12±0.53	105.12±1.76
		Sample B	20.09±0.45	2	22.69±0.44	102.69±1.88
				5	26.35±0.35	104.98±1.12
				10	33.02±0.72	109.71±1.32
	Arginine	Sample A	6.84±0.08	2	9.04±0.09	102.28±1.06
				5	11.42±0.08	96.47±0.73
				10	16.81±0.14	99.82±0.84
		Sample B	9.78±0.34	2	11.62±0.20	98.60±1.77
				5	14.85±0.14	100.45±1.01
				10	19.66±0.45	99.38±2.36
CAK	Citrulline	Sample A	26.86±0.89	2	28.51±0.95	98.79±0.80
				5	32.39±1.37	101.62±1.02
				10	35.42±0.72	96.10±0.83
		Sample B	20.39±1.56	2	22.19±1.24	99.13±1.67
				5	25.83±1.95	101.75±2.03
				10	29.08±0.49	95.68±1.02

Table S4: Inter-and intra-day precision results of HPLC and Citrulline Assay kit methods using two representative watermelon fruit samples

Method	Compound	Sample	Intra-day precision (n = 5)		Inter-day precision (n = 5)	
			Content (mg/g DW)	RSD (%)	Content (mg/g DW)	RSD (%)
HPLC	Citrulline	Sample A	29.85	0.90	30.04	1.93
		Sample B	20.84	0.63	20.88	4.29
	Arginine	Sample A	6.78	1.22	6.71	0.95
		Sample B	9.42	1.85	9.30	1.08
CAK	Citrulline	Sample A	26.78	5.49	27.22	4.48
		Sample B	18.95	4.09	19.80	3.58

Table S5: Results of the first three principle components of citrulline and arginine for investigated watermelon fruit samples

Compound	PC1	PC2	PC3
Citrulline (HPLC)	0.6545	-0.2837	0.7008
Arginine (HPLC)	0.3653	0.9302	0.03538
Citrulline (CAK)	0.662	-0.2329	-0.7124
Eigen value	2.0795146	0.8348078	0.0856776
%Variance	69.31715	27.82693	2.85592
