# Supplementary material for: Fruit Morphology, Citrulline, and Arginine Levels in Diverse Watermelon (Citrullus lanatus) Germplasm Collections
Source: Plants (Basel). 2020 Aug 19;9(9):1054. doi: 10.3390/plants9091054 (PMC7569901; doi:10.3390/plants9091054)
Supplement: Supplementary file 1 [file plants-09-01054-s001.zip › Supplementary Material 2.pdf]

Fruit morphology, citrulline, and arginine levels in diverse watermelon (*Citrullus lanatus*) germplasm collections

Awraris Derbie Assefa[1], On-Sook Hur[1], Na-Young Ro[1], Jae-Eun Lee[1], Ae-Jin Hwang[1], Bich-Saem Kim[1], Ju-Hee Rhee[1], Jung Yoon Yi[1], Ji Hyun Kim[1], Ho-Sun Lee[2], Jung-Sook Sung[3], Myung-Kon Kim[4], Jae-Jong Noh[5]*

[1]National Agrobiodiversity Center, National Institute of Agricultural Sciences, RDA, Jeonju 54874, Rep. of Korea

[2]International Technology Cooperation Center, RDA, Jeonju 54875, Rep. of Korea

[3]Upland Crop Breeding Division, Department of Southern Area Crop Science, National Institute of Crop Science, RDA, Miryang 50424, Rep. of Korea

[4]Department of Food Science and Technology, Jeonbuk National University, Jeonju 54896, Rep. of Korea

[5]Jeonbuk Agricultural Research and Extension Services, Iksan 54591, Rep. of Korea

*Corresponding author: Email nohjj@korea.kr; Tel. +82-63-238-4940

Figure S1 Photos of watermelon fruit germplasm collections with wide phenotypic diversity

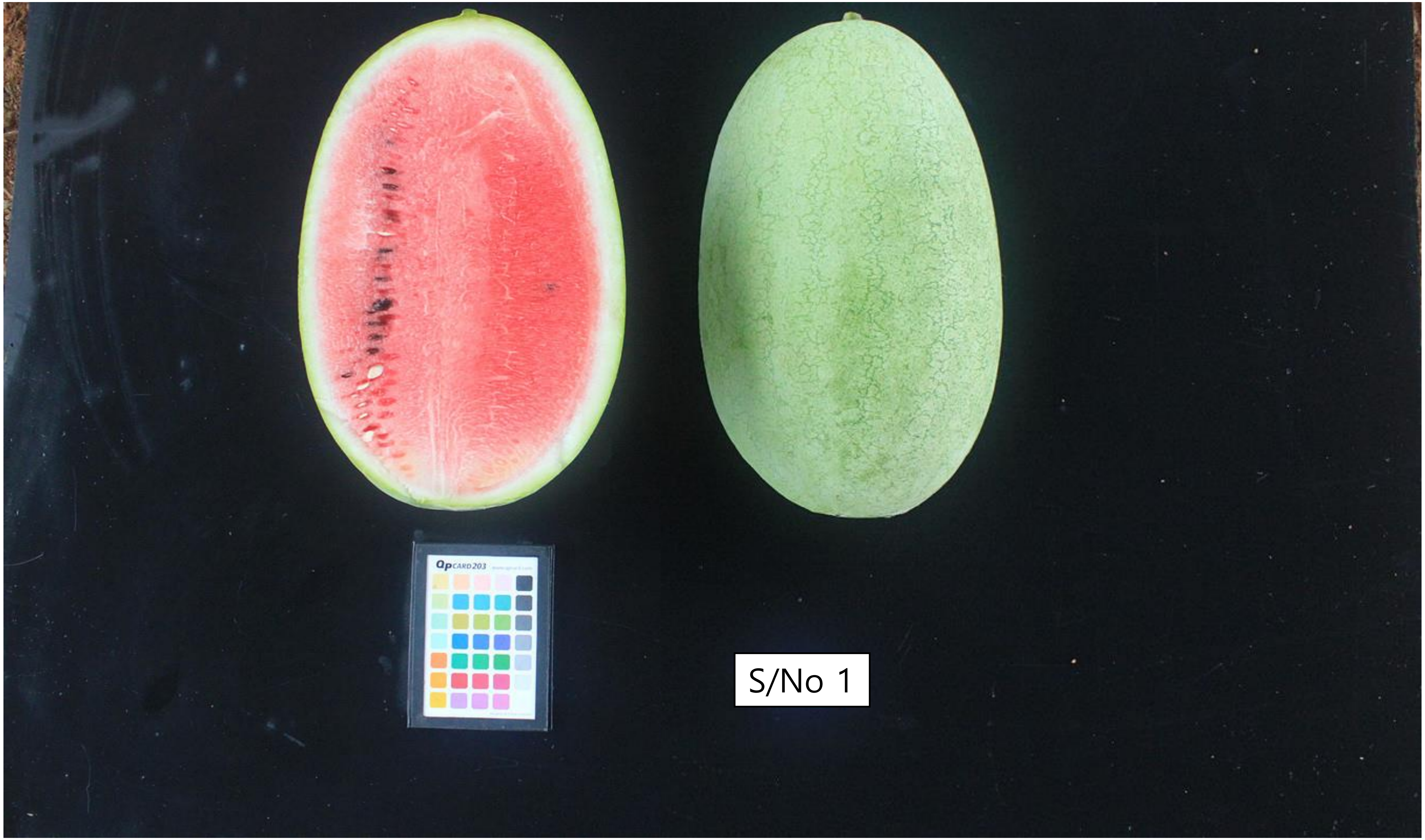
QpCARD203
S/No 1

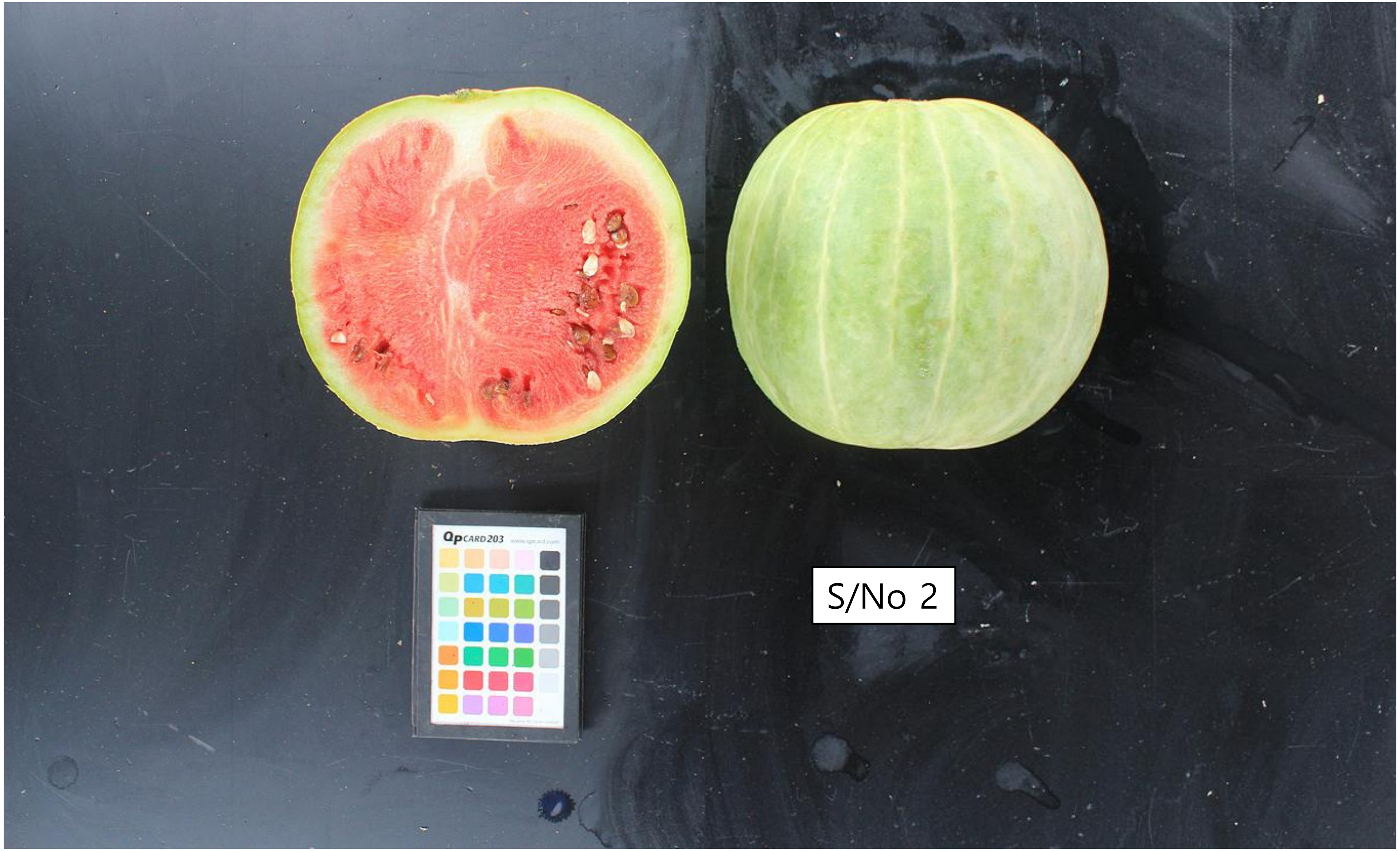
Qp CARD 203
S/No 2

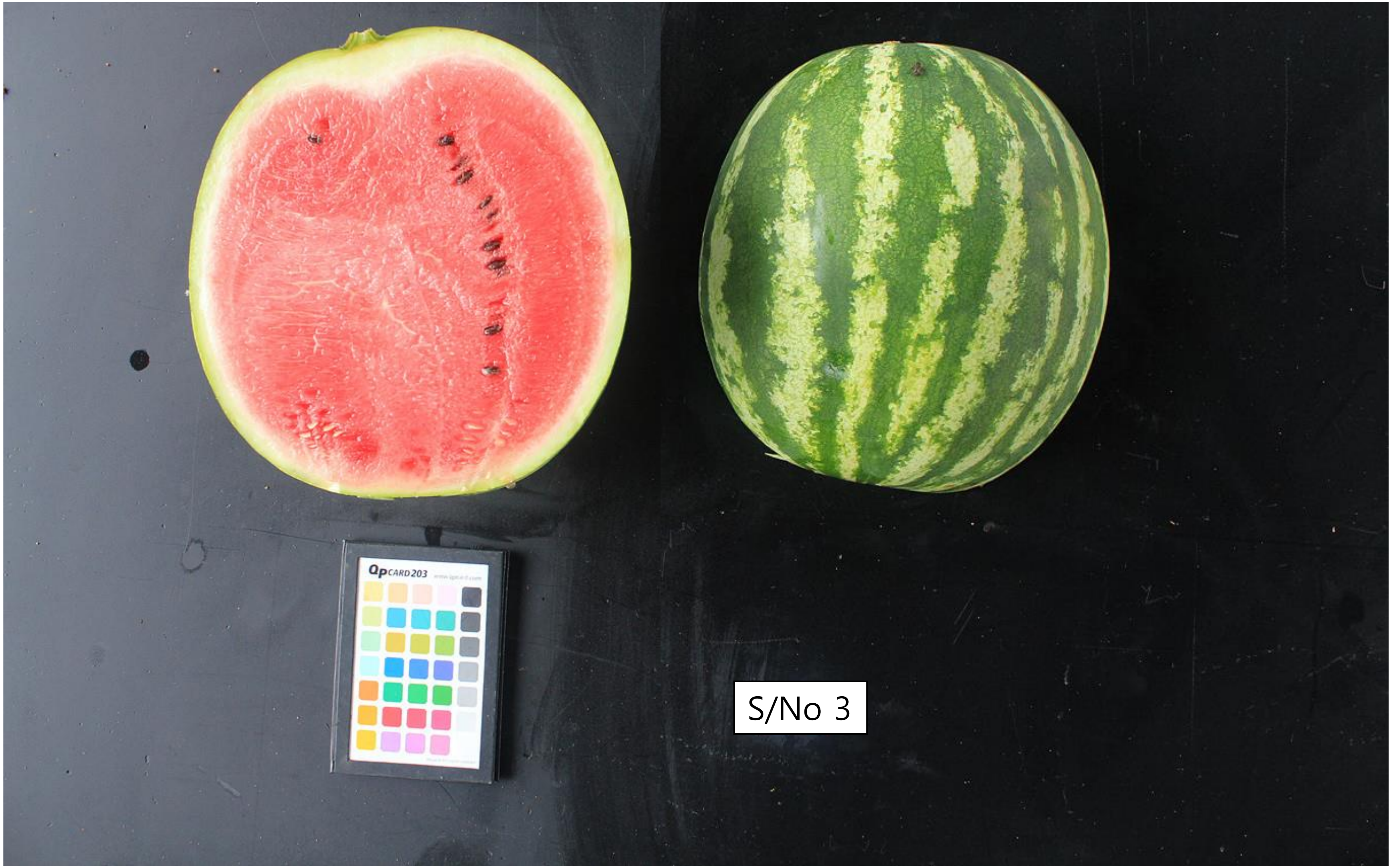
QpCARD 203
S/No 3

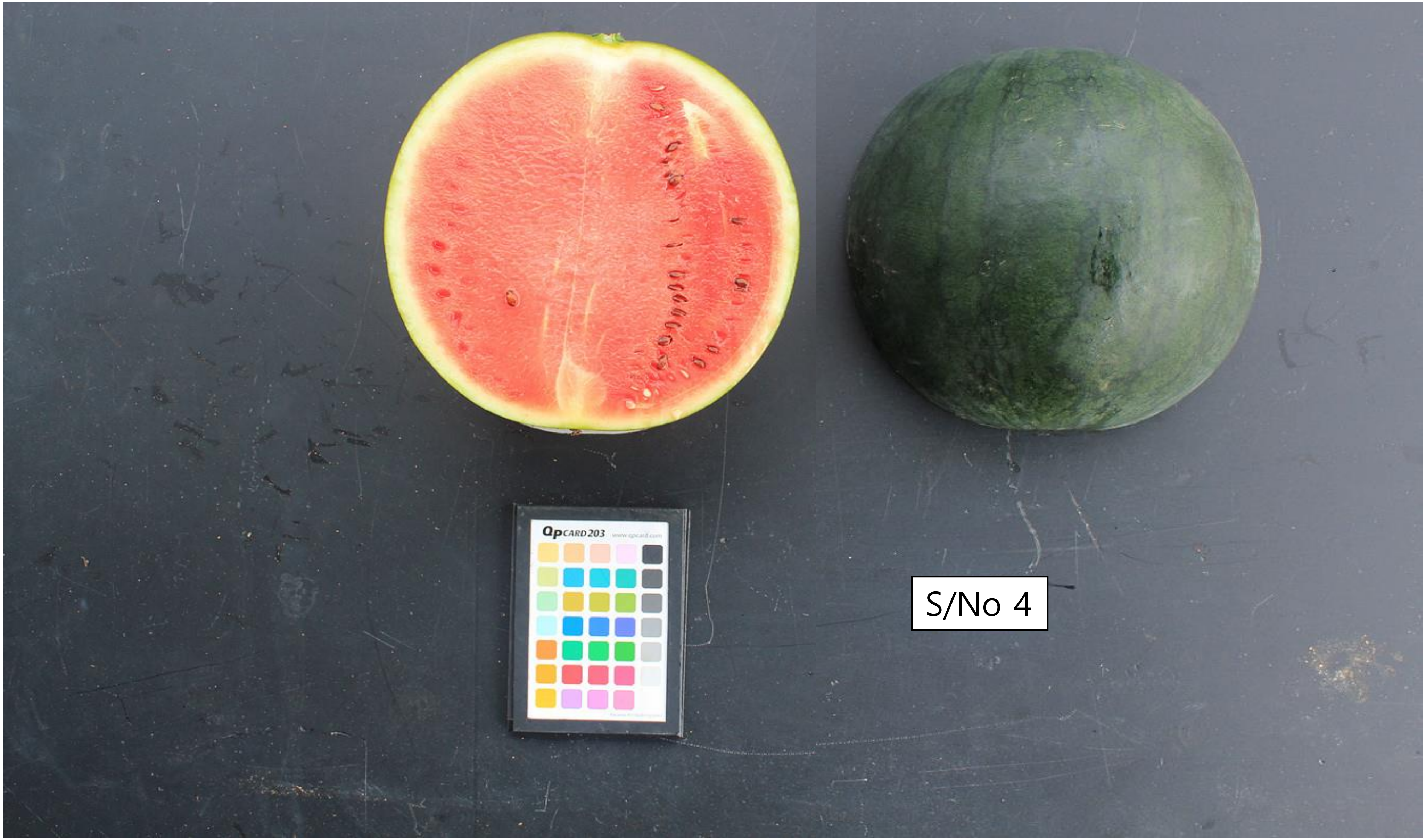
QpCARD 203
S/No 4

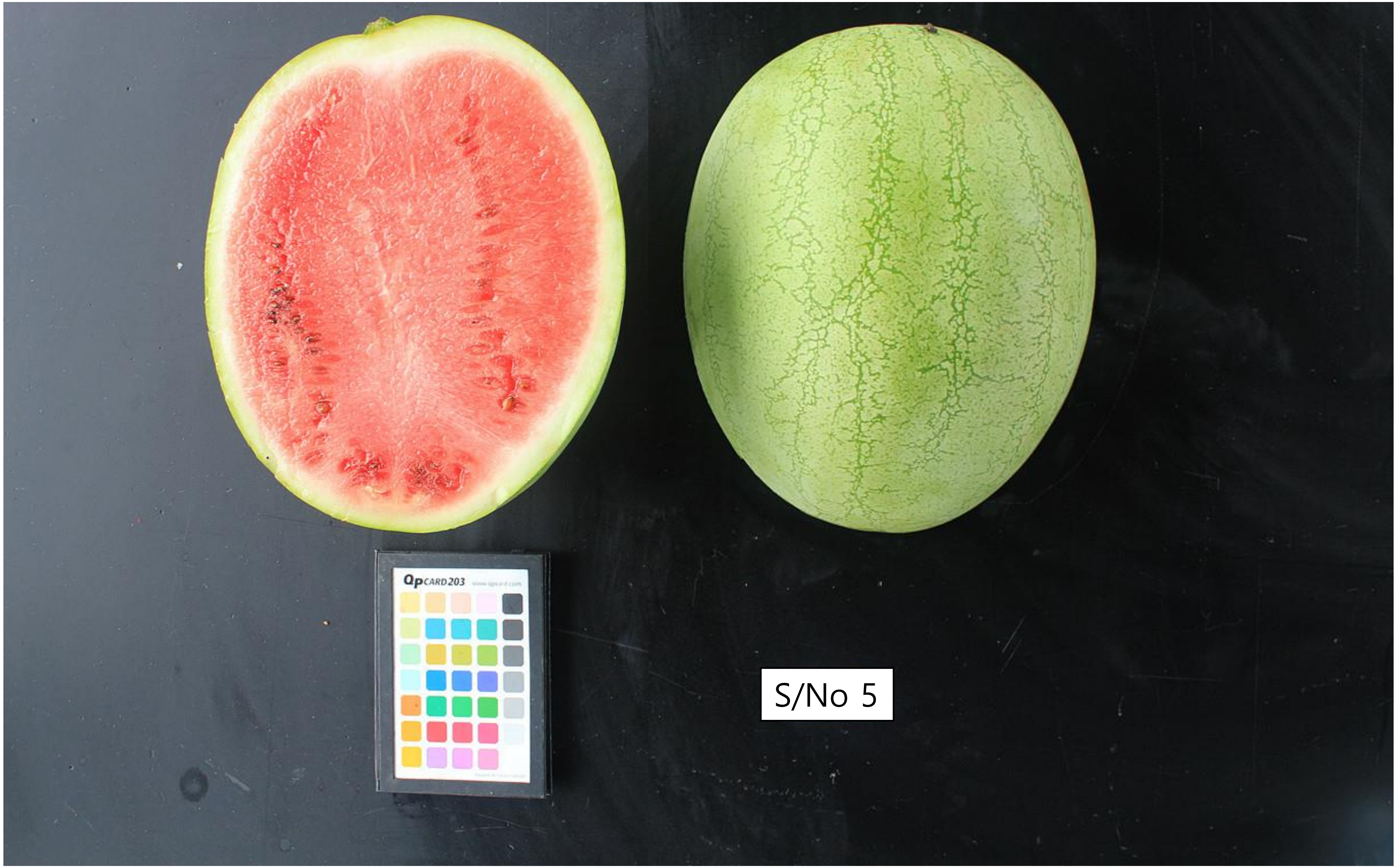
QpCARD 203
S/No 5

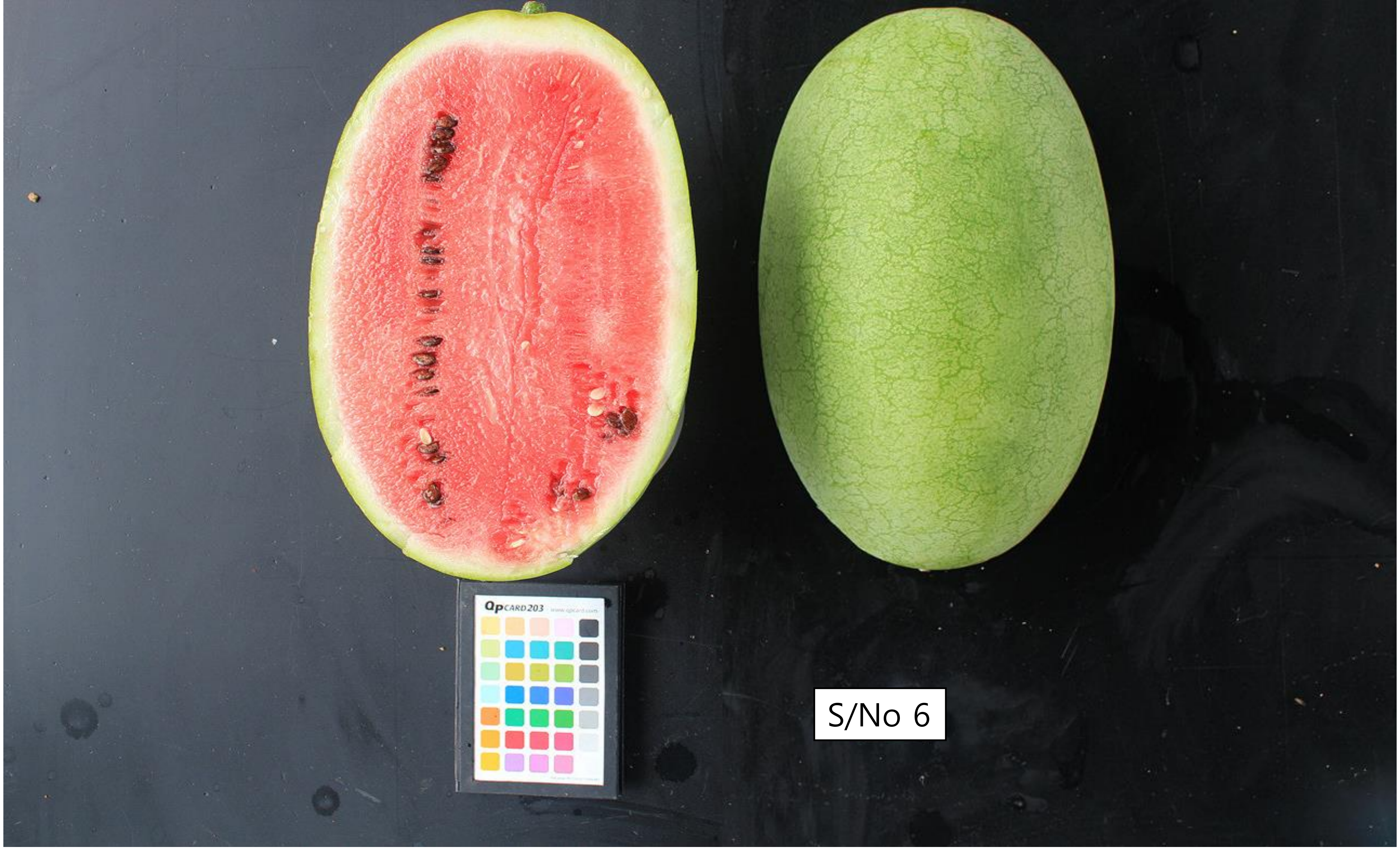
QpCARD 203
S/No 6

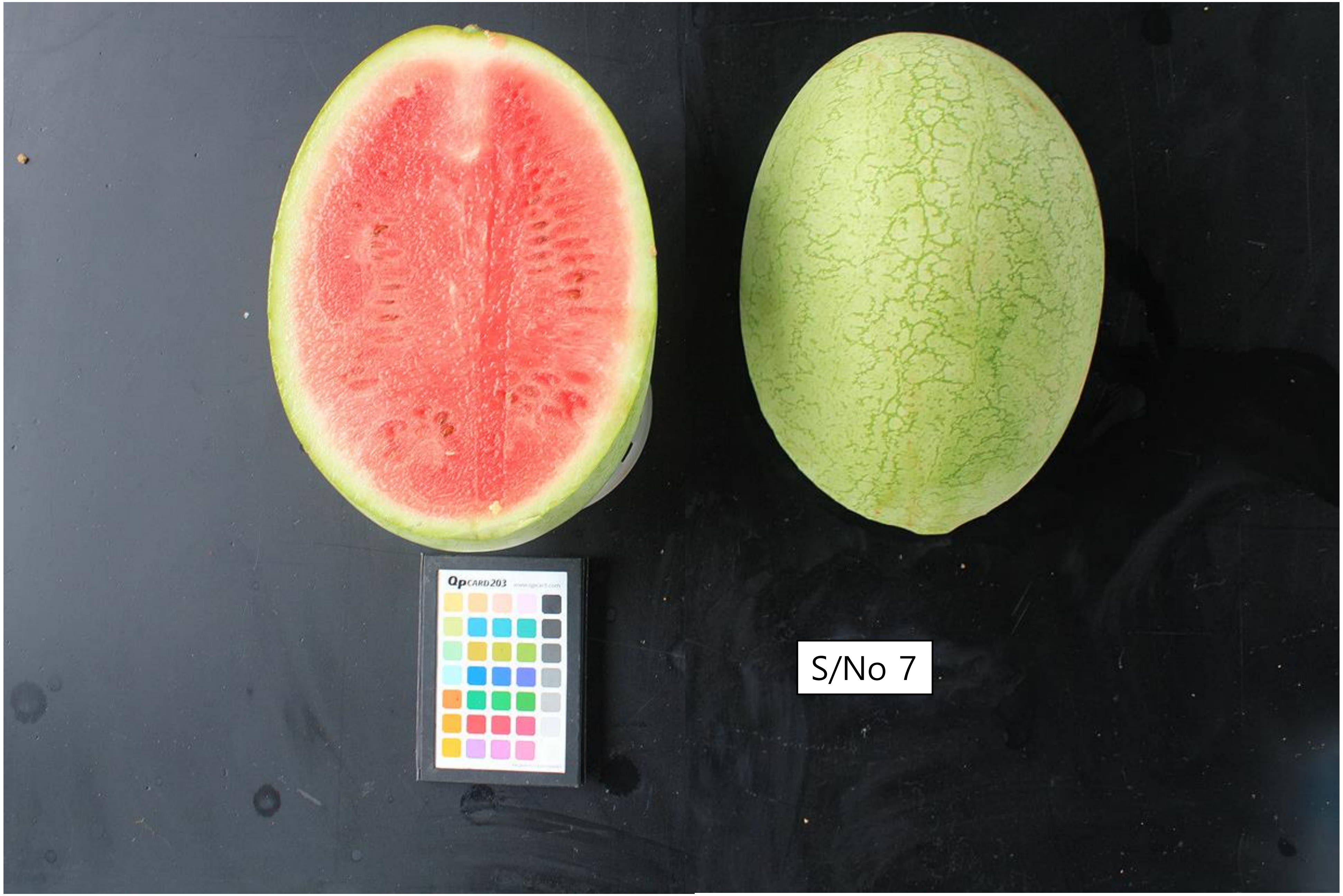
QpCARD 203
S/No 7

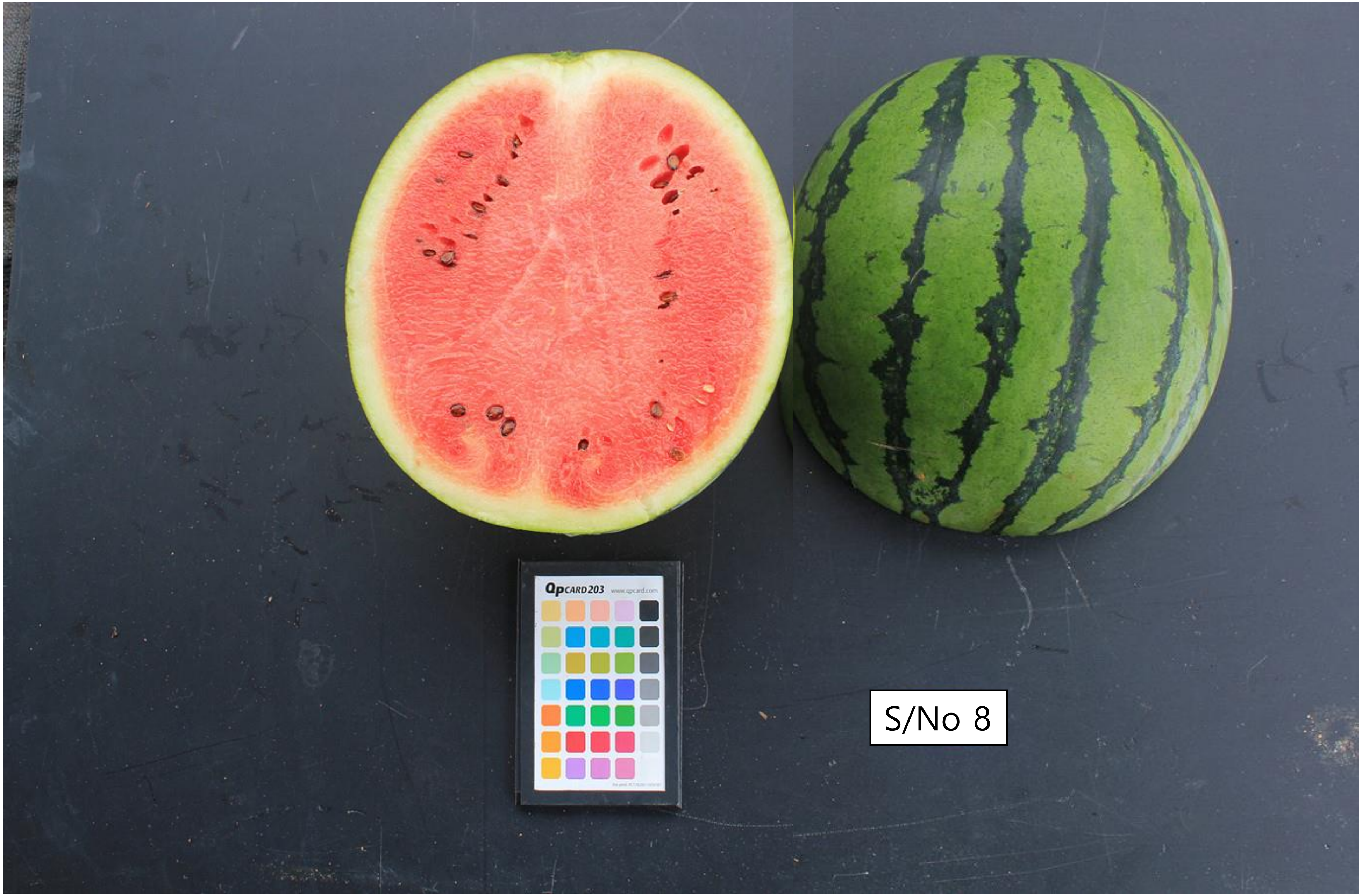
QpCARD203 www.qpcard.com
S/No 8

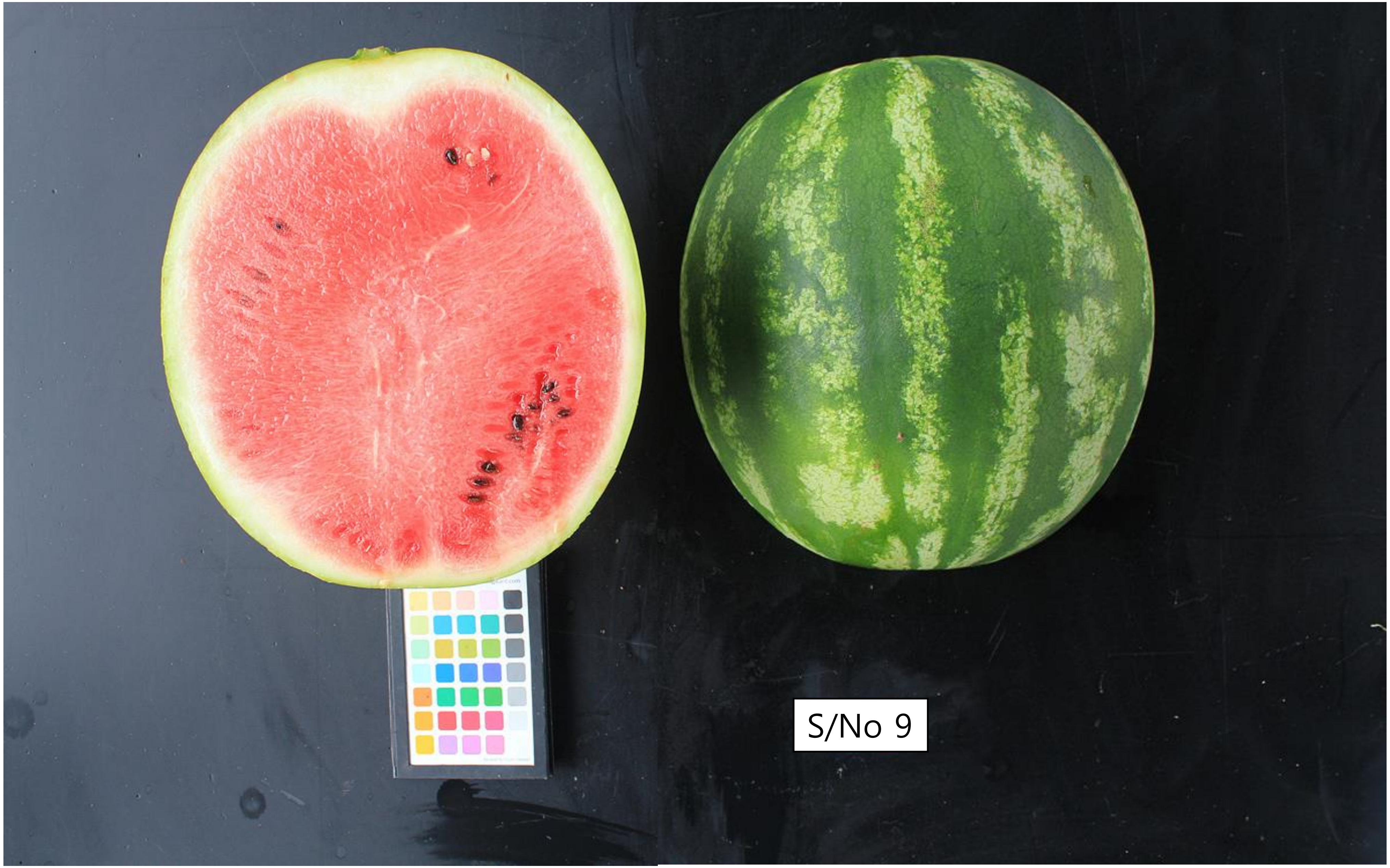
S/No 9

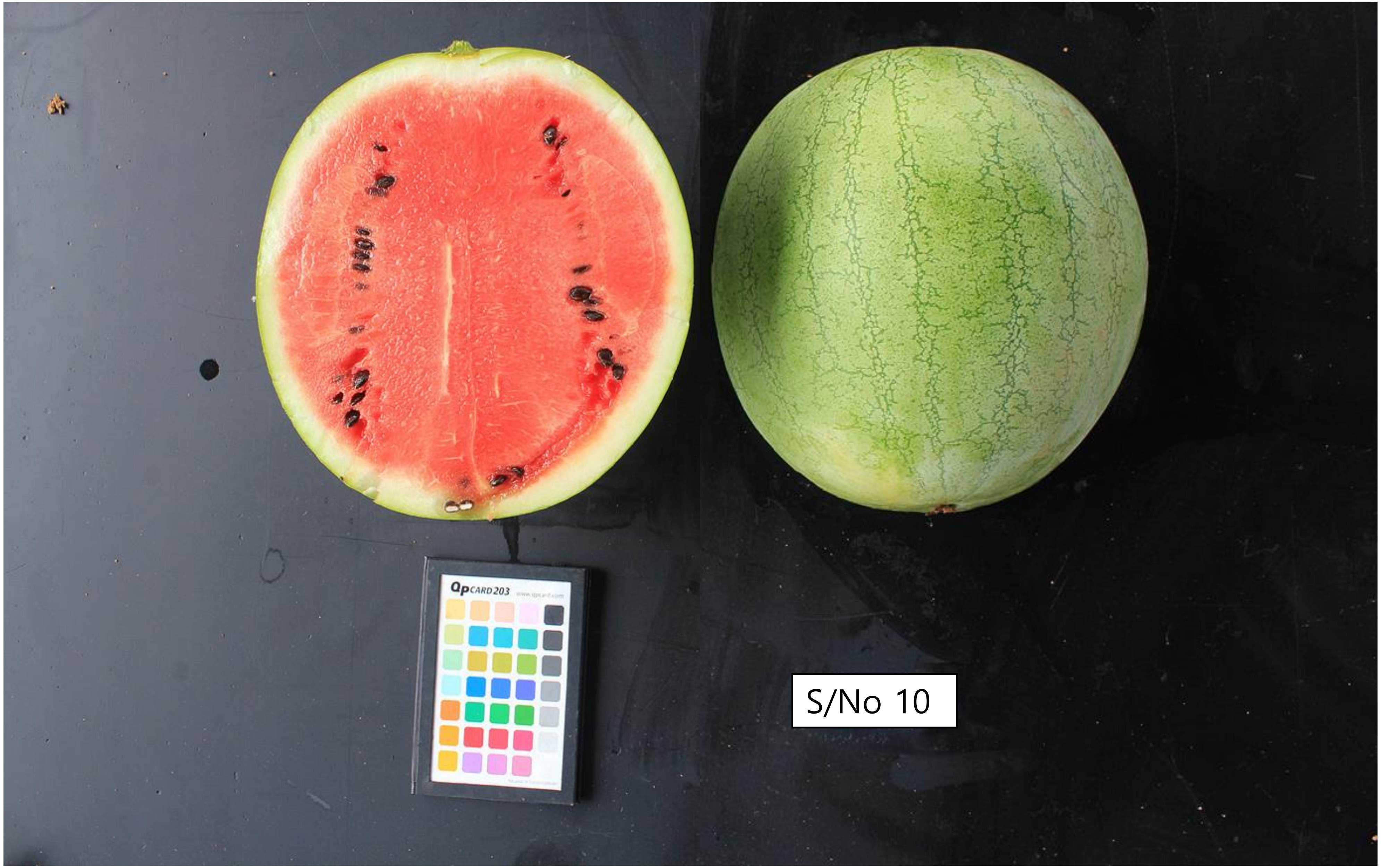
QpCARD 203
S/No 10

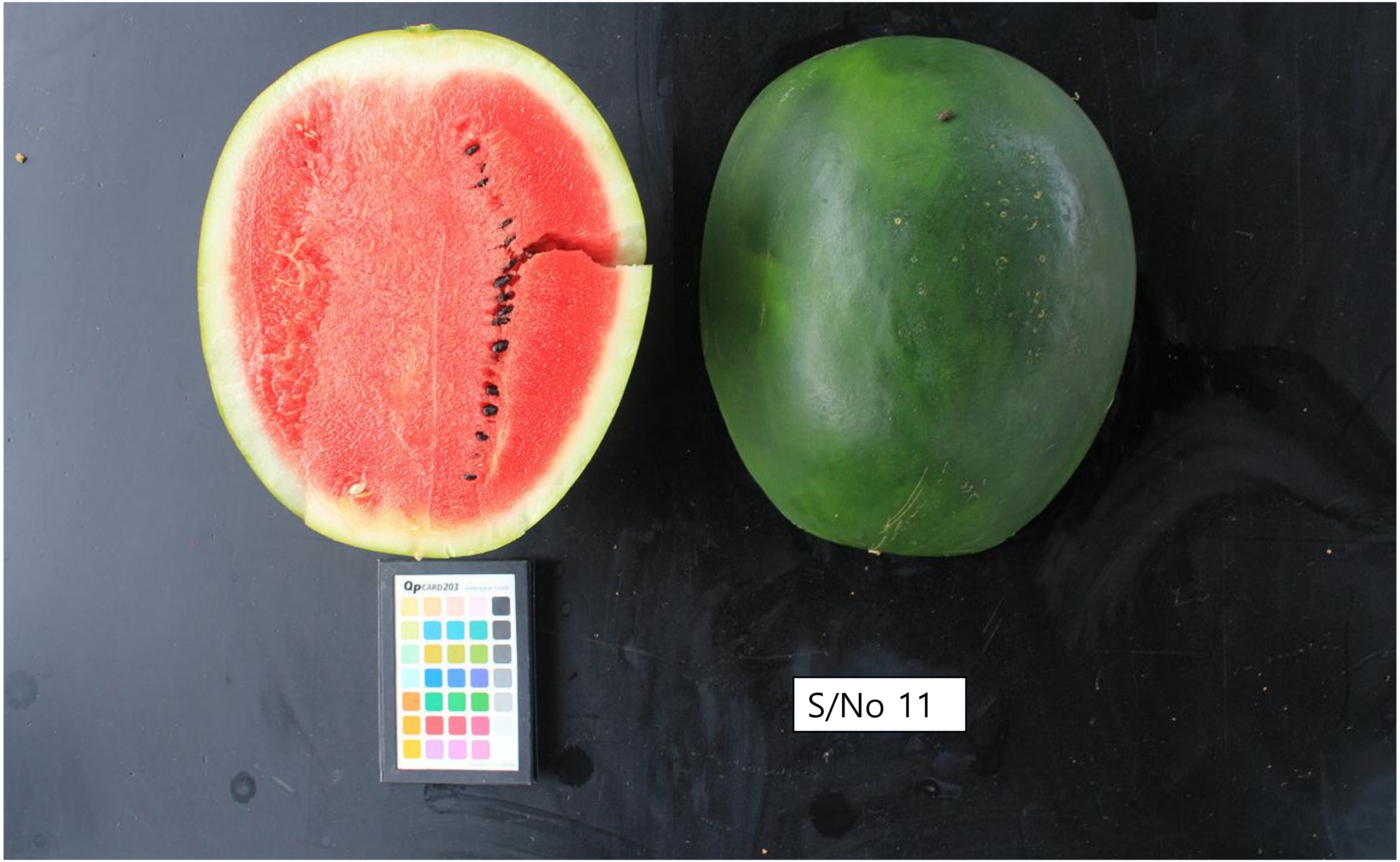
QpCARD203
S/No 11

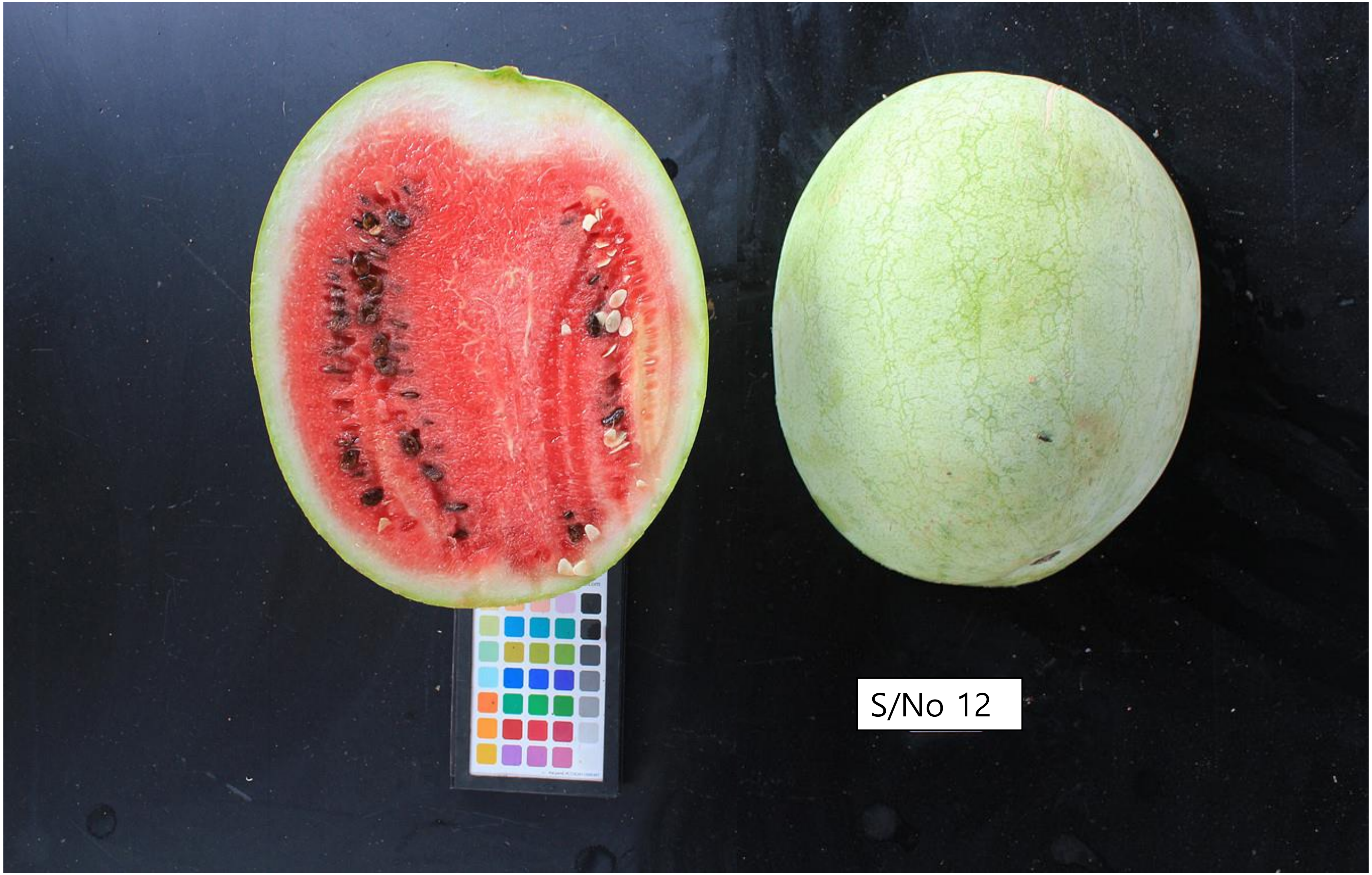
S/No 12

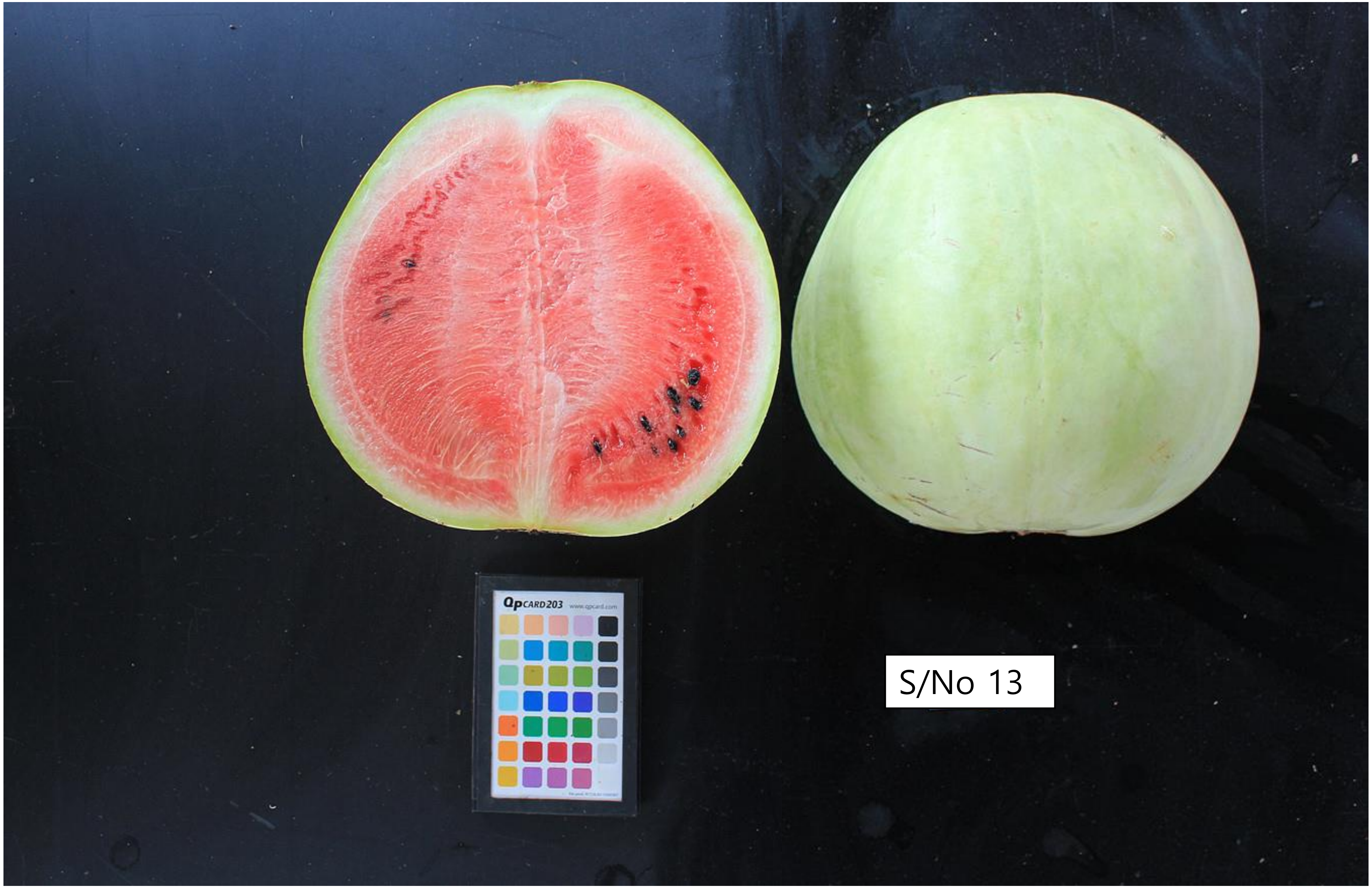
QpCARD 203
www.qpcard.com
S/No 13

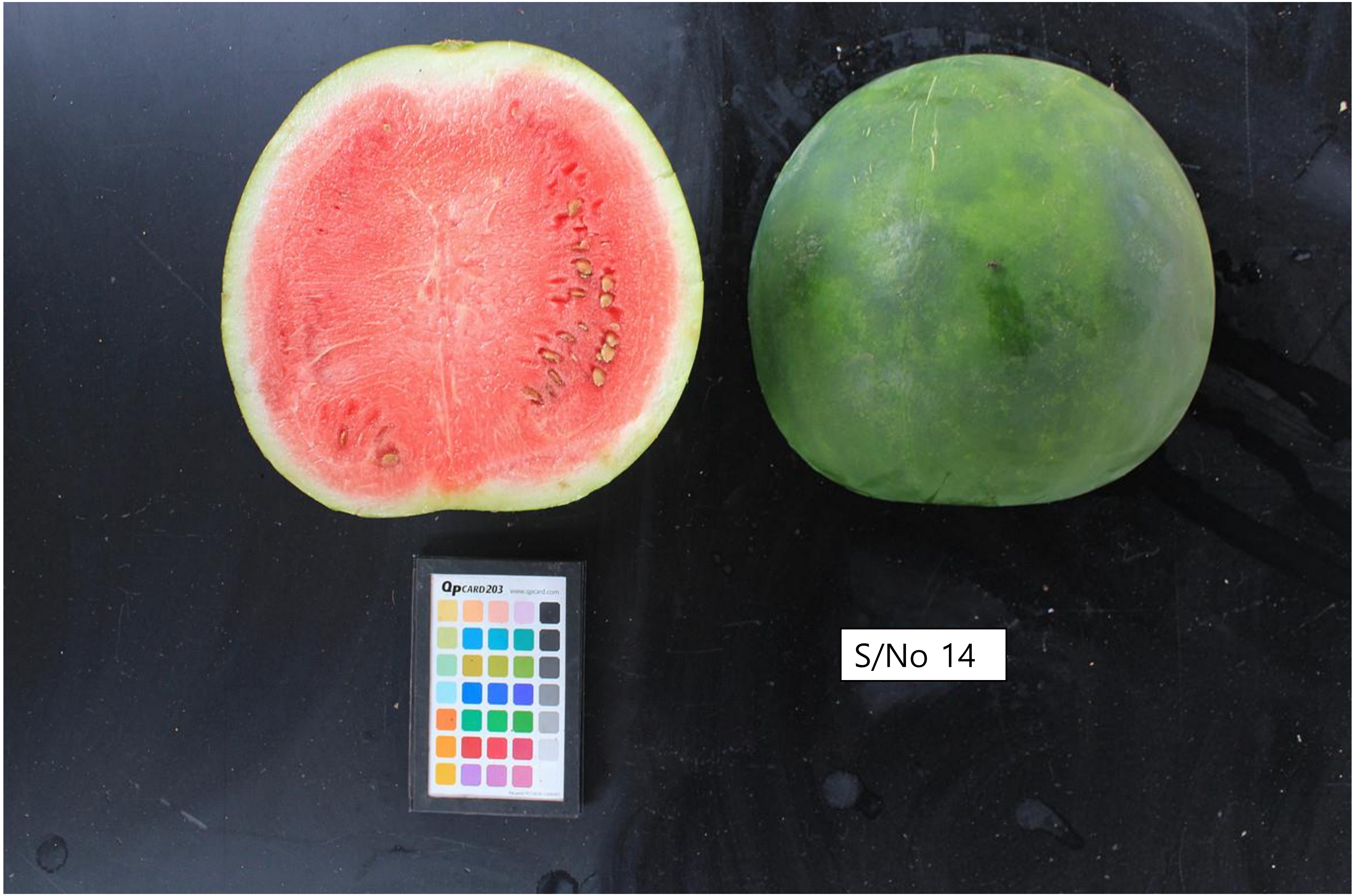
QpCARD203
www.qpcard.com
S/No 14

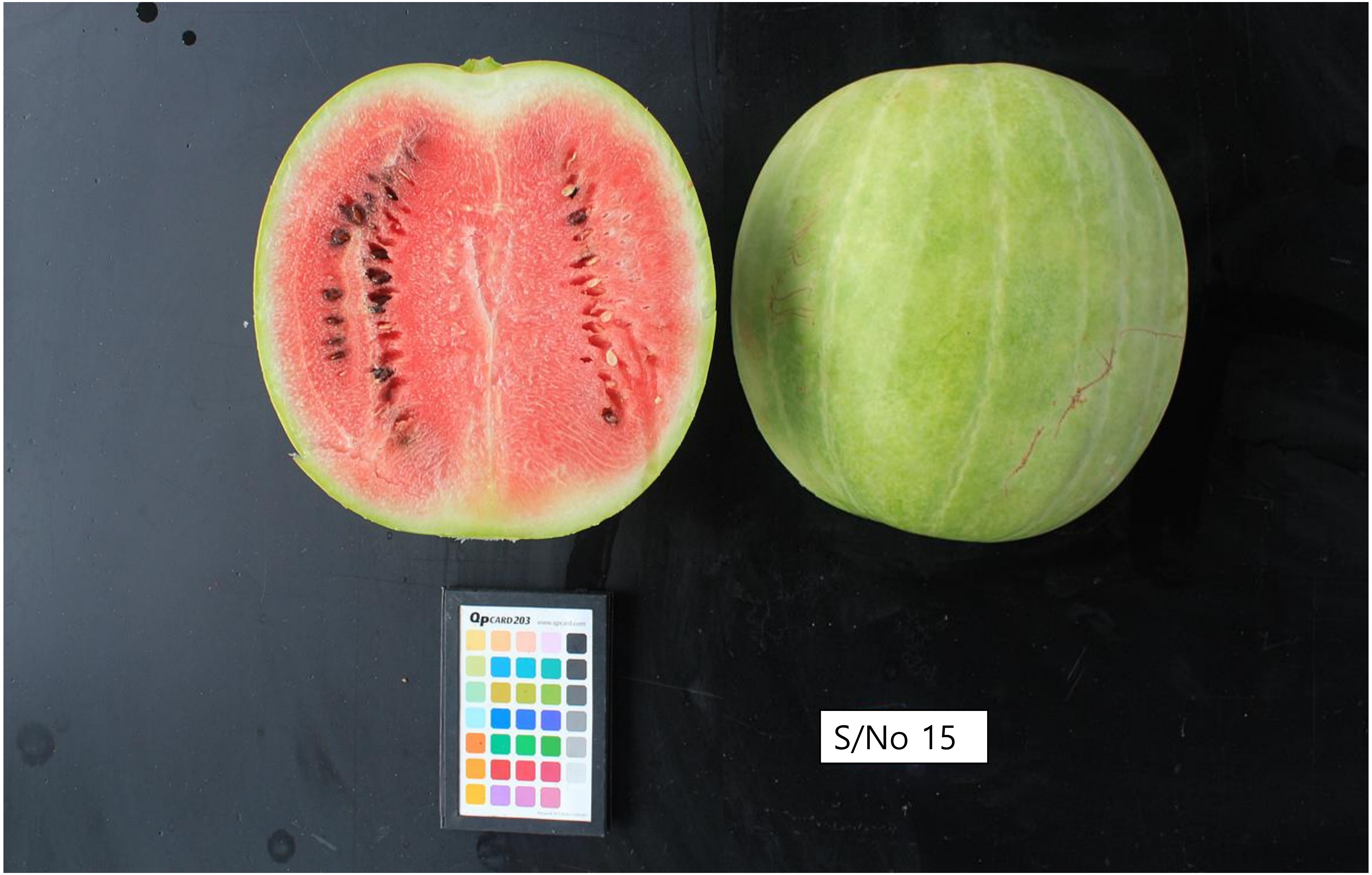
QpCARD 203
S/No 15

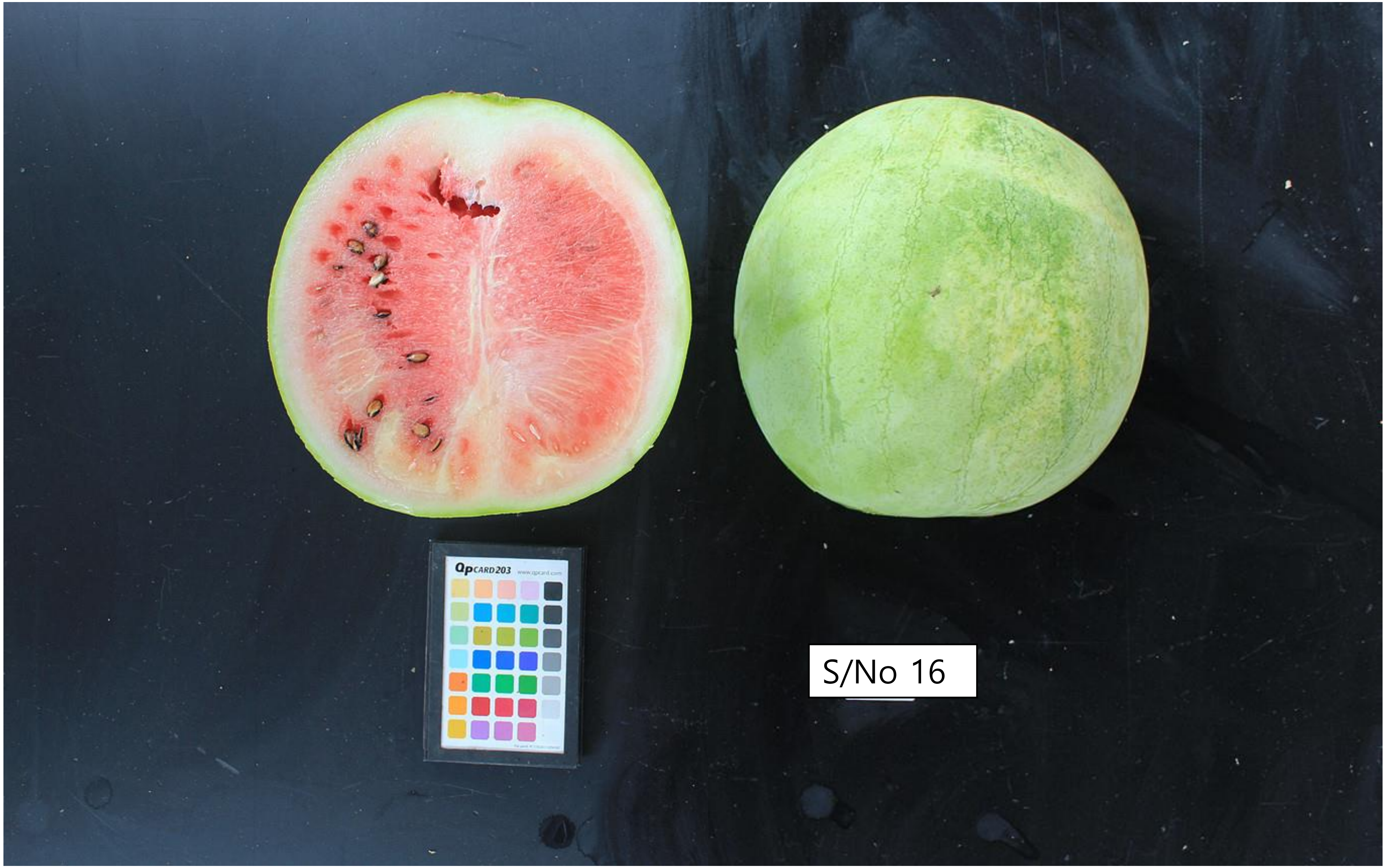
QpCARD 203
www.qpcard.com
S/No 16

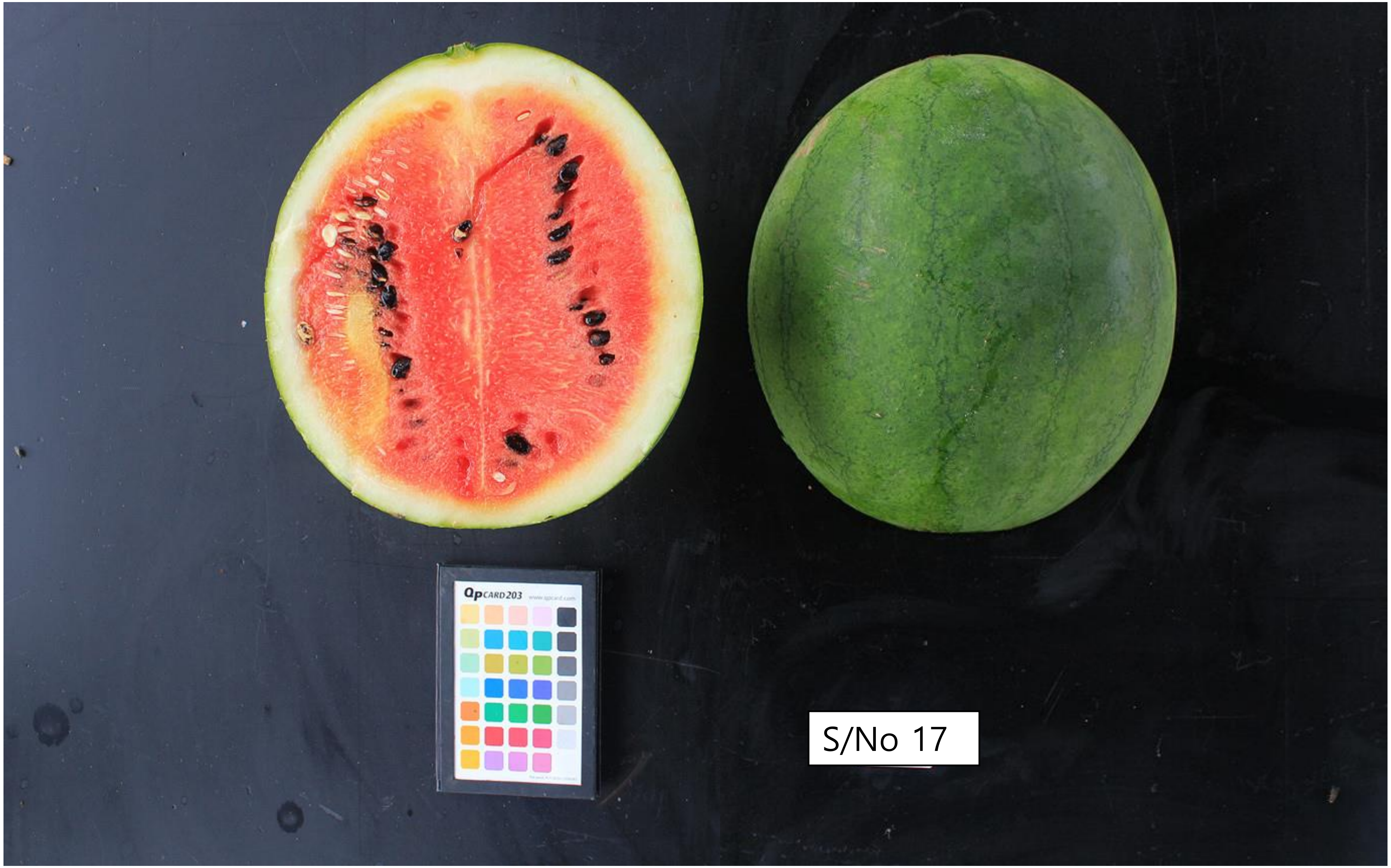
QpCARD 203
S/No 17

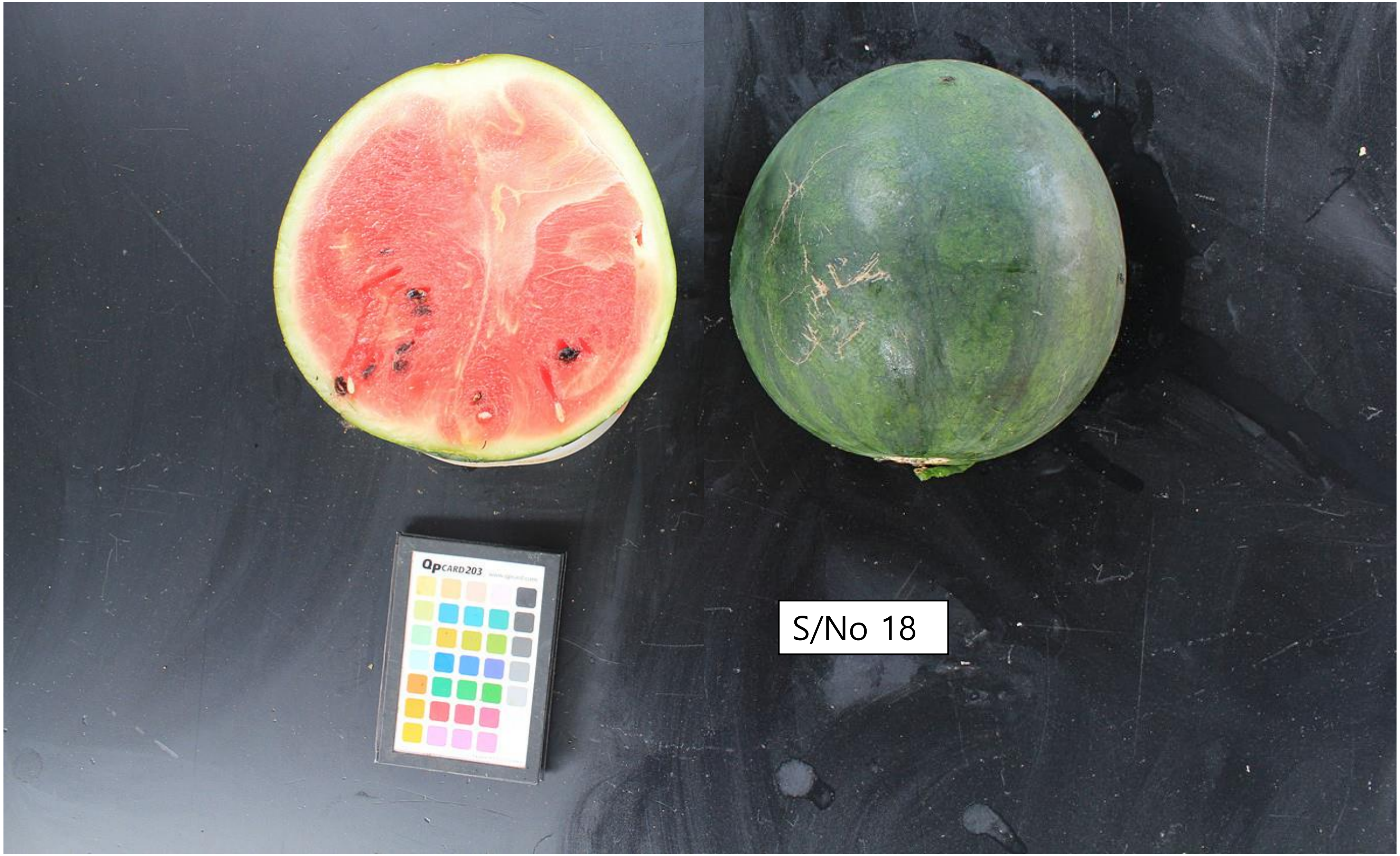
QpCARD 203
S/No 18

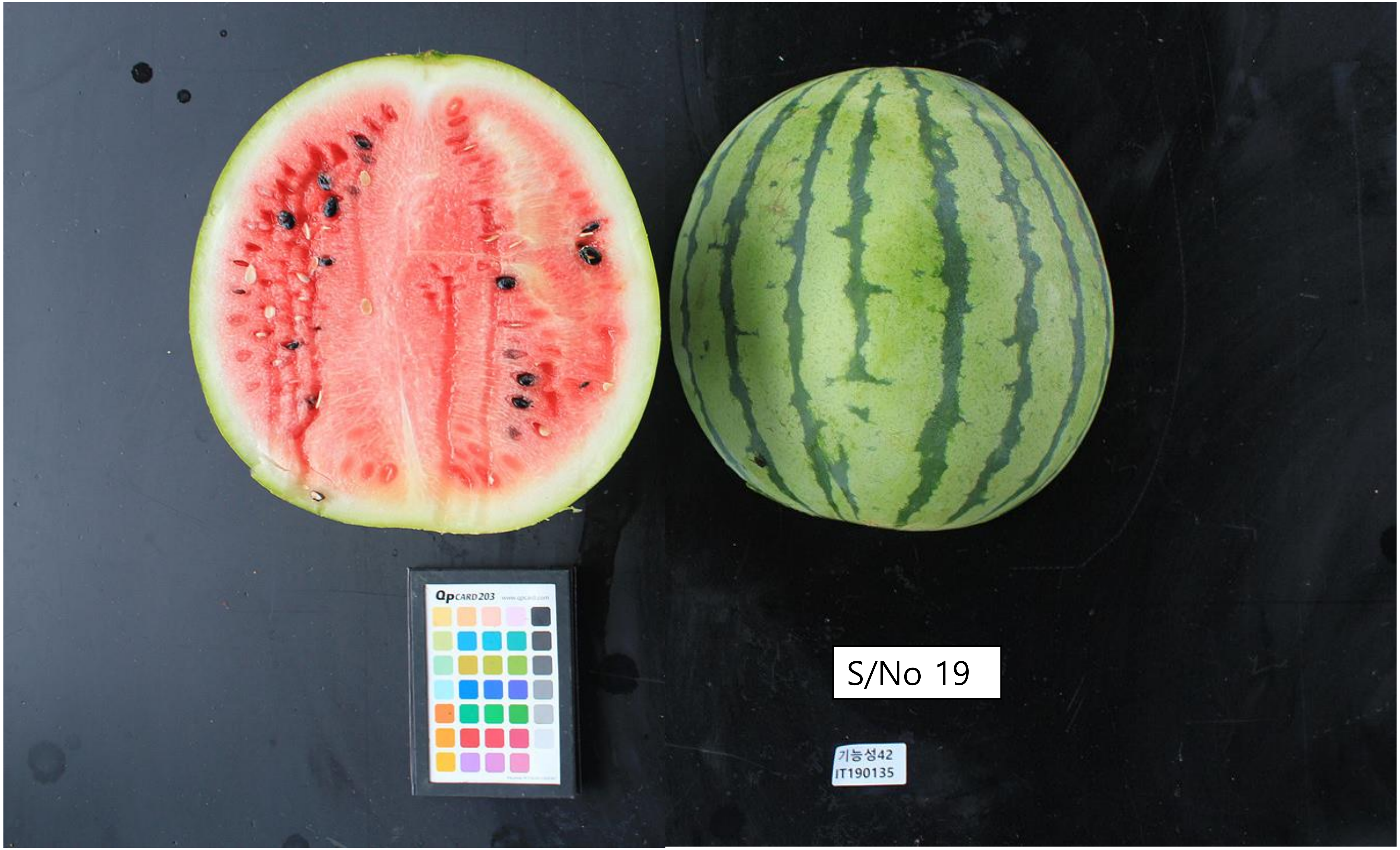
QpCARD203
S/No 19
기능성42
IT190135

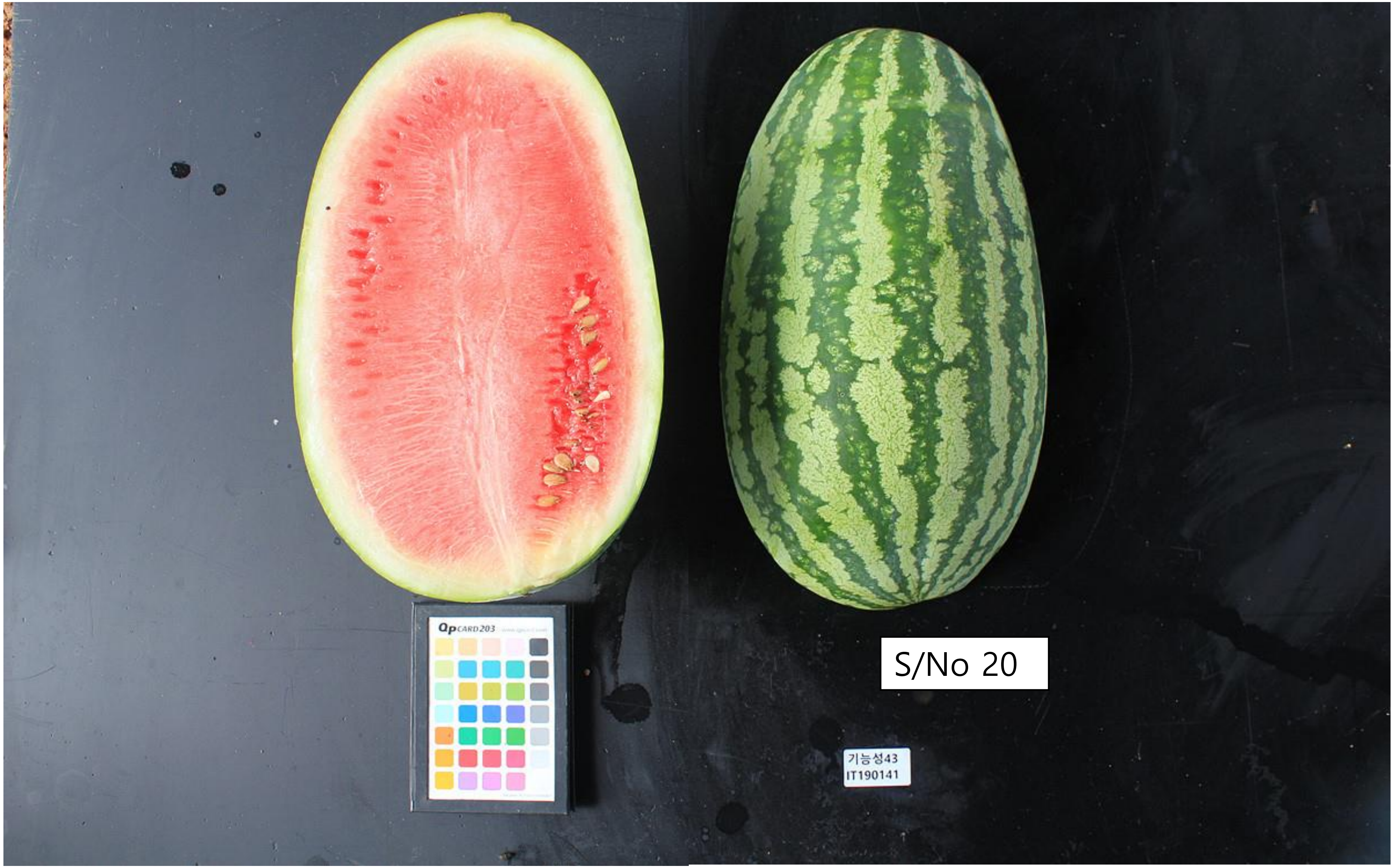
Qp CARD 203
S/No 20
기능성43
IT190141

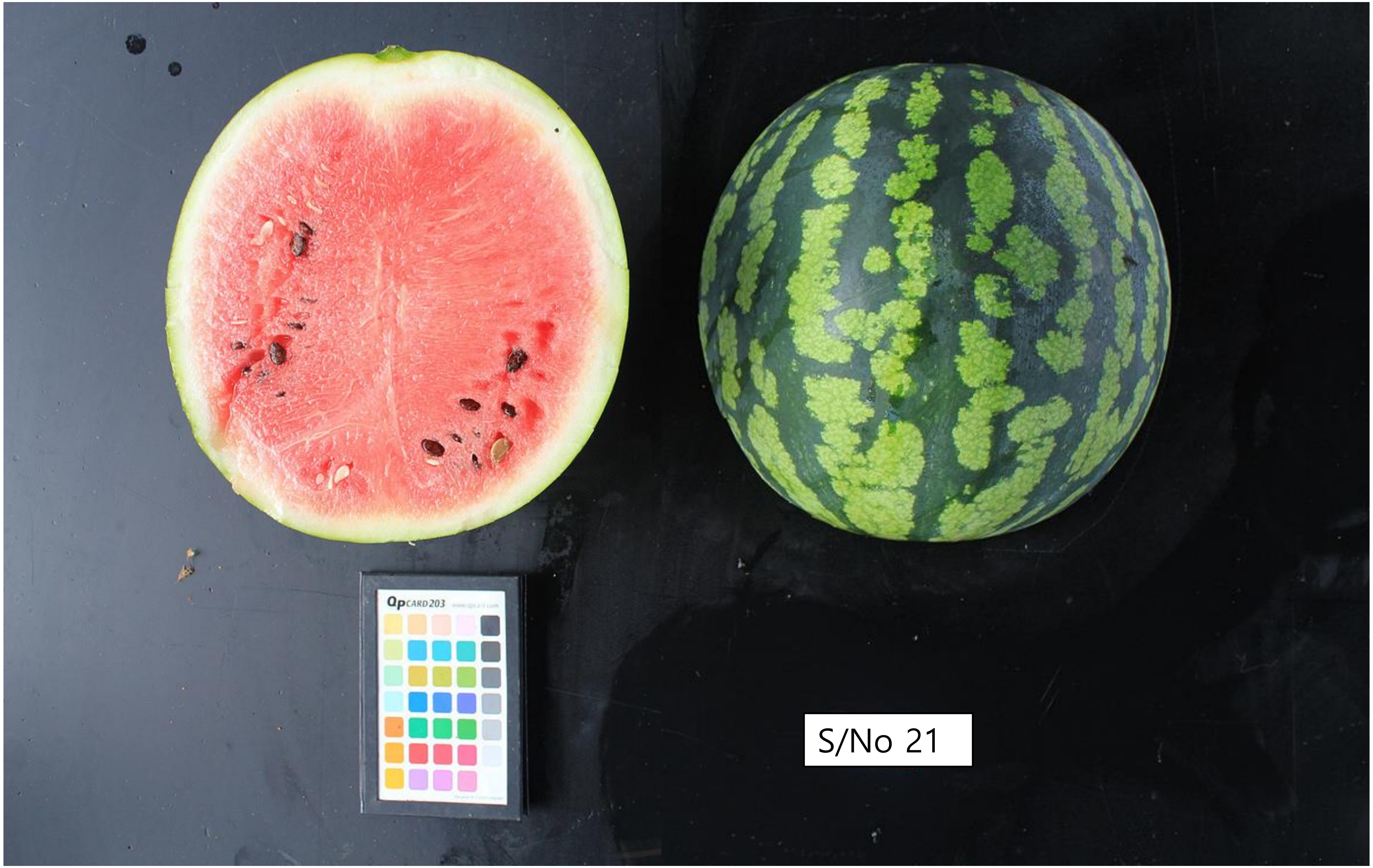
QpCARD203
S/No 21

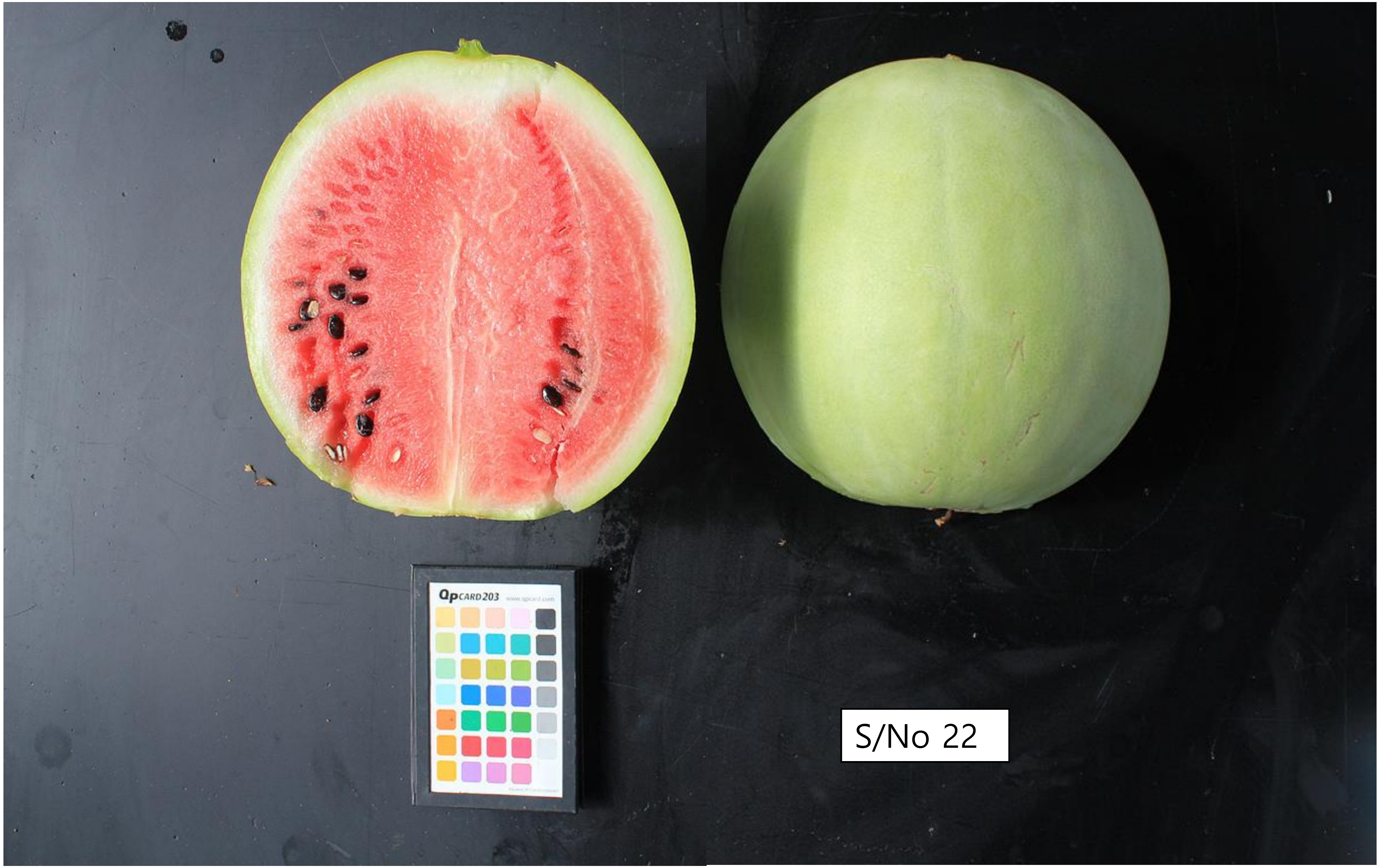
QpCARD 203
S/No 22

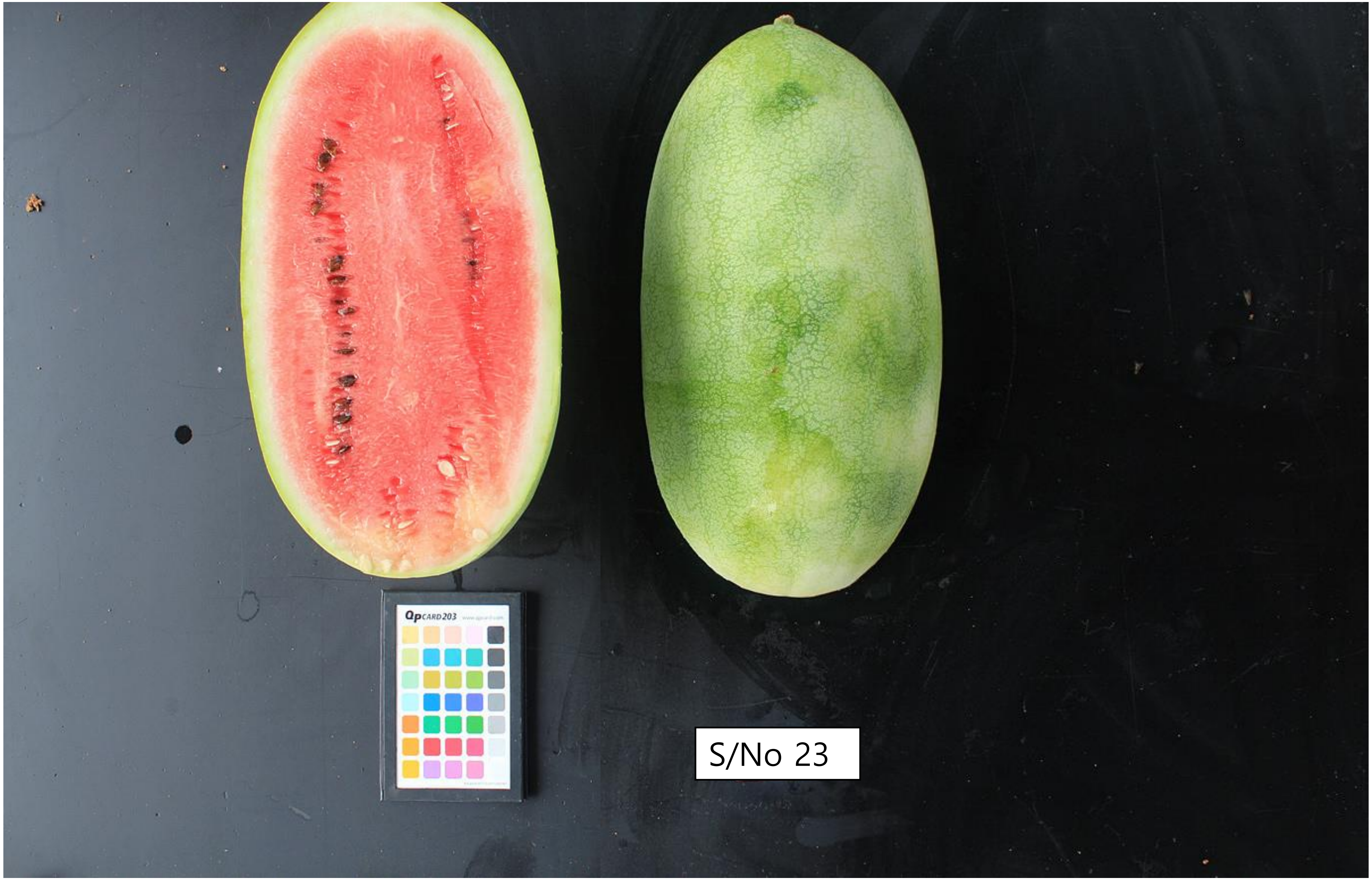
QpCARD 203
S/No 23

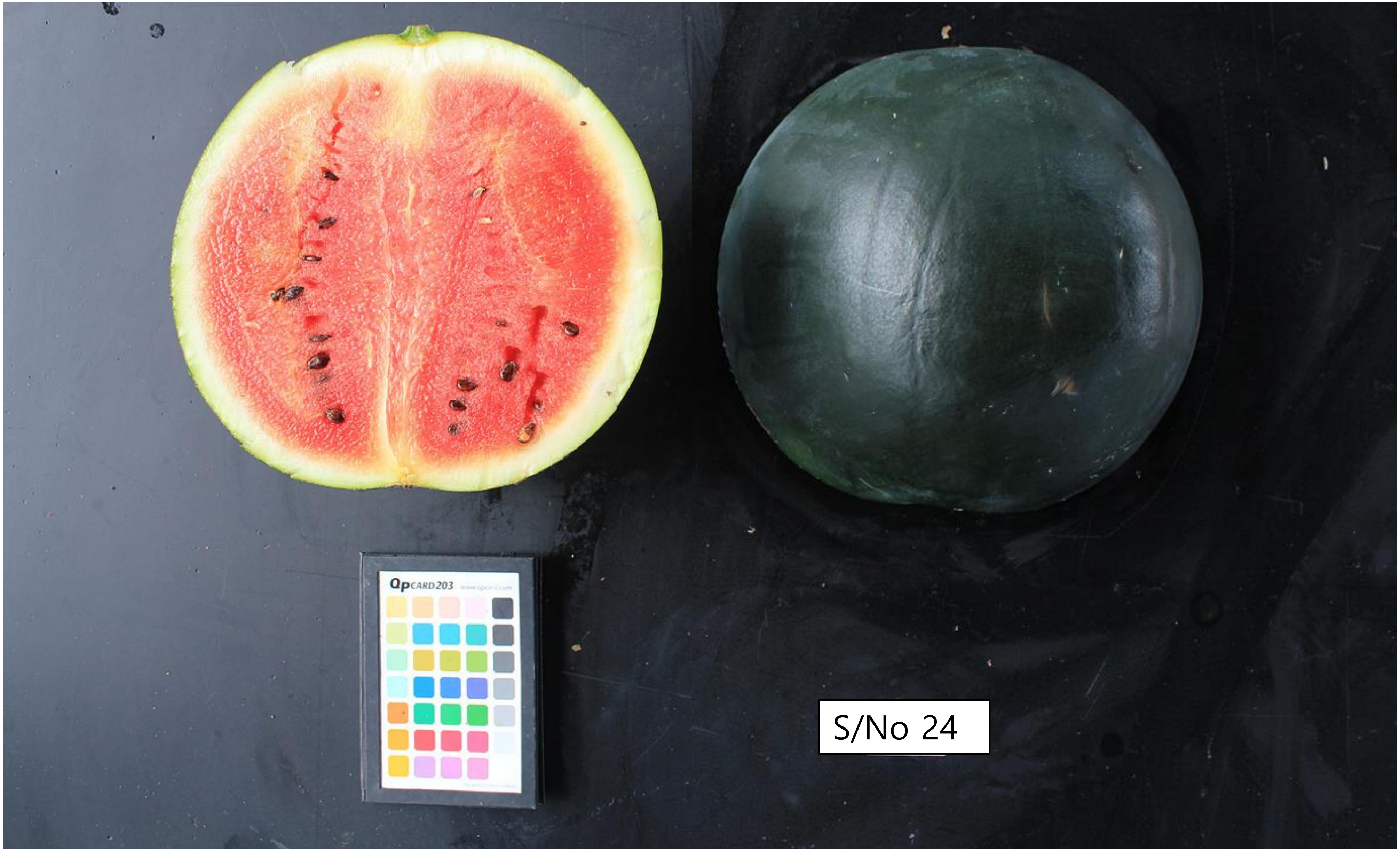
QpCARD 203
S/No 24

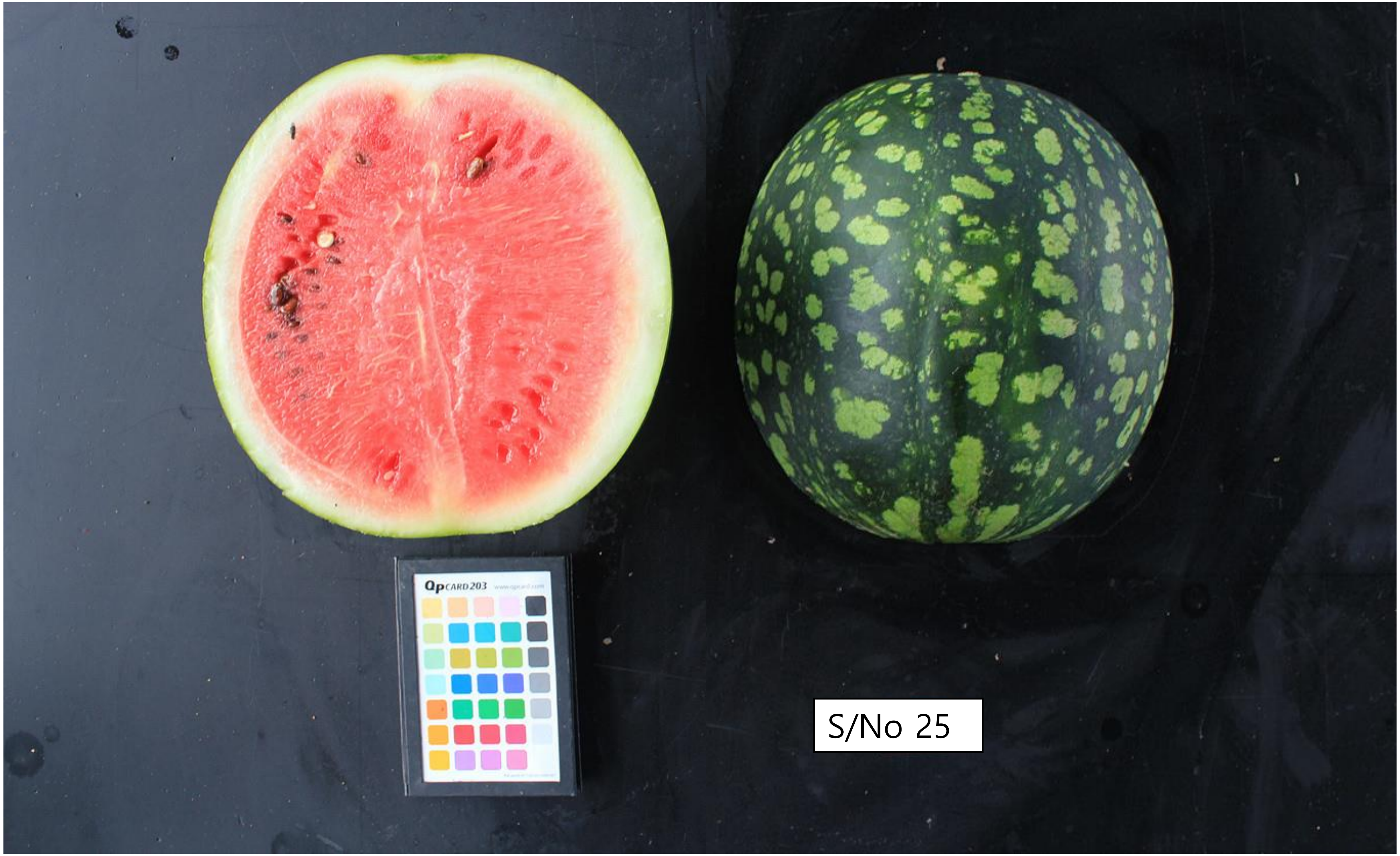
QpCARD 203
S/No 25

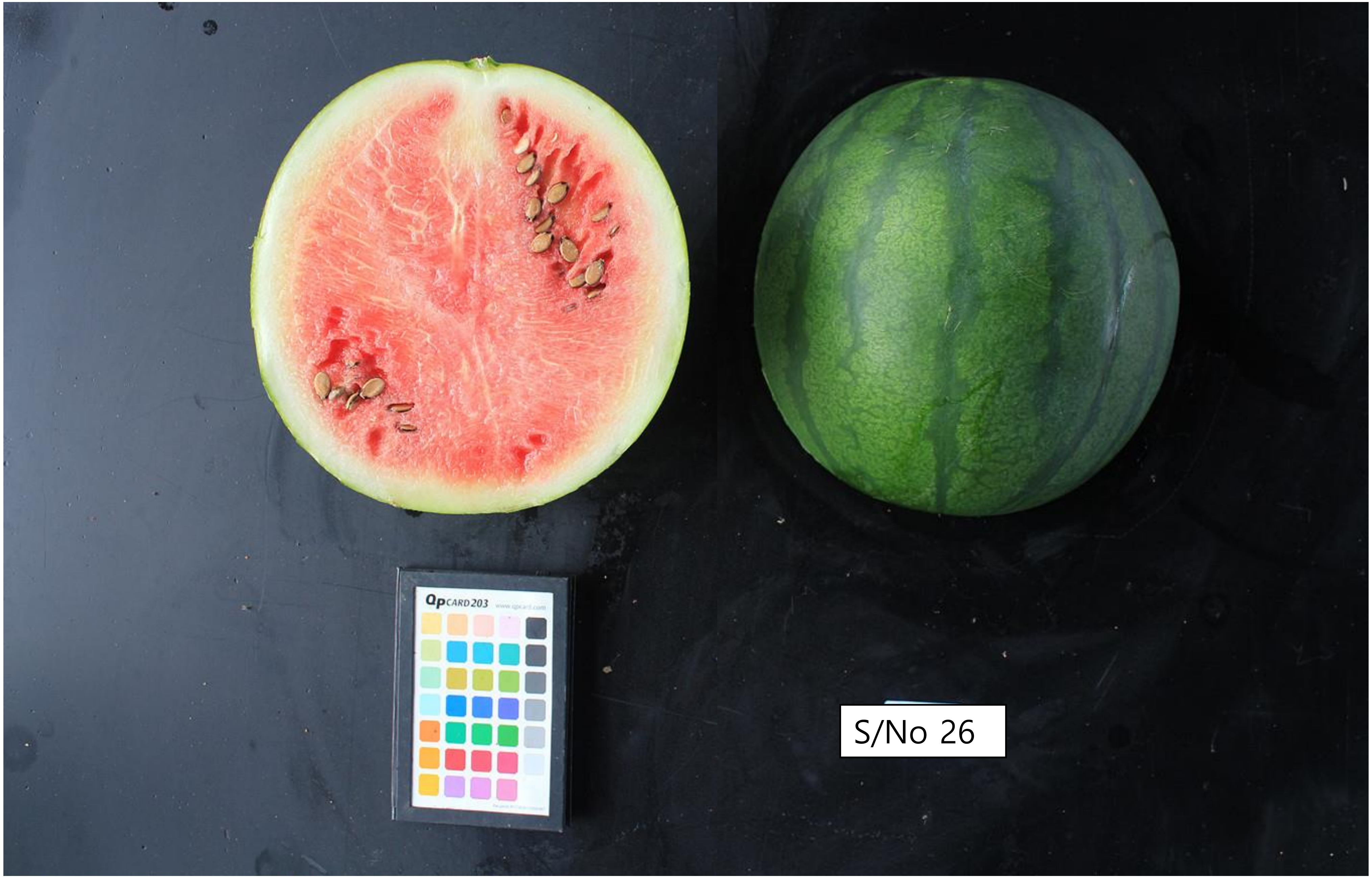
QpCARD 203
S/No 26

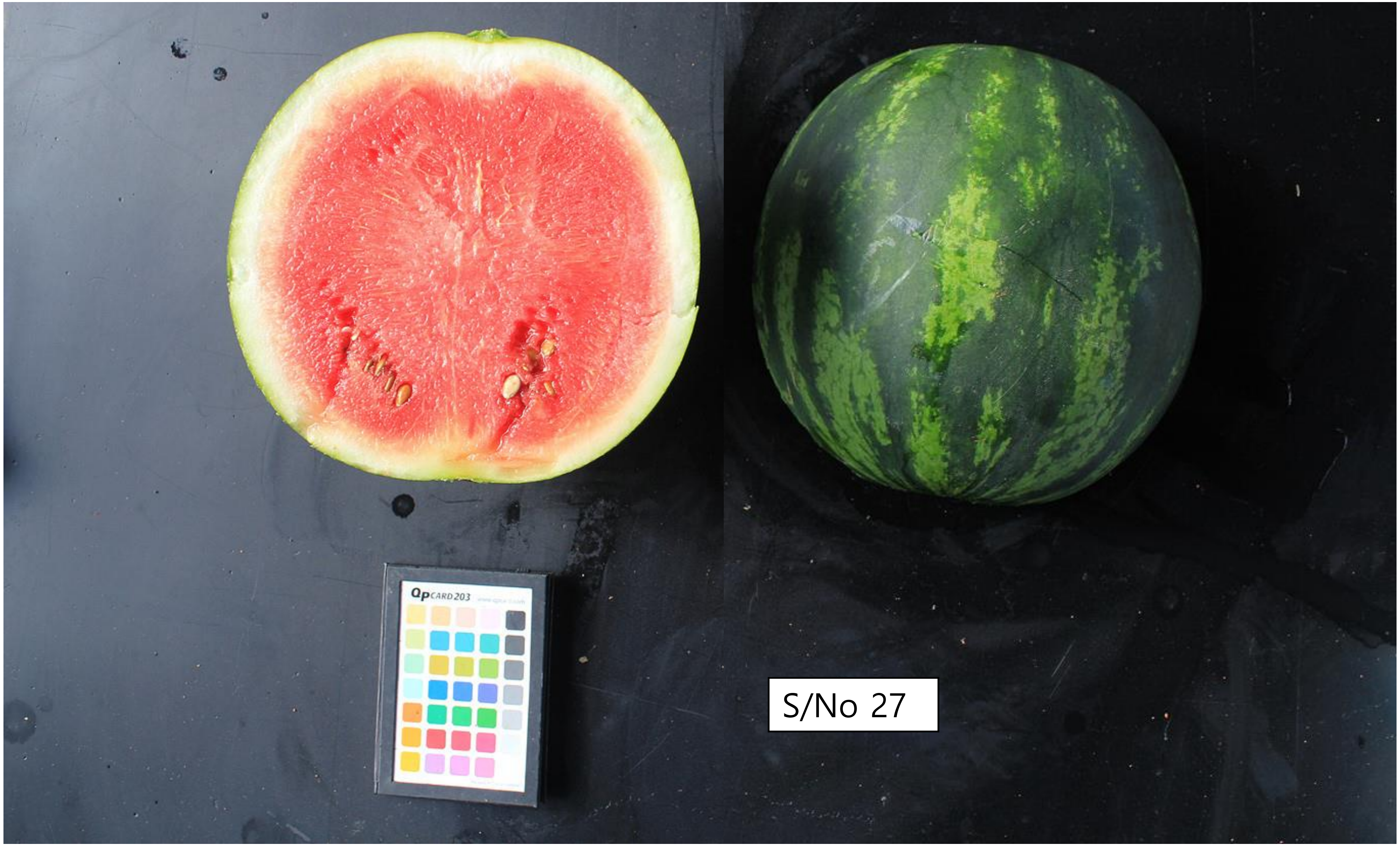

S/No 27

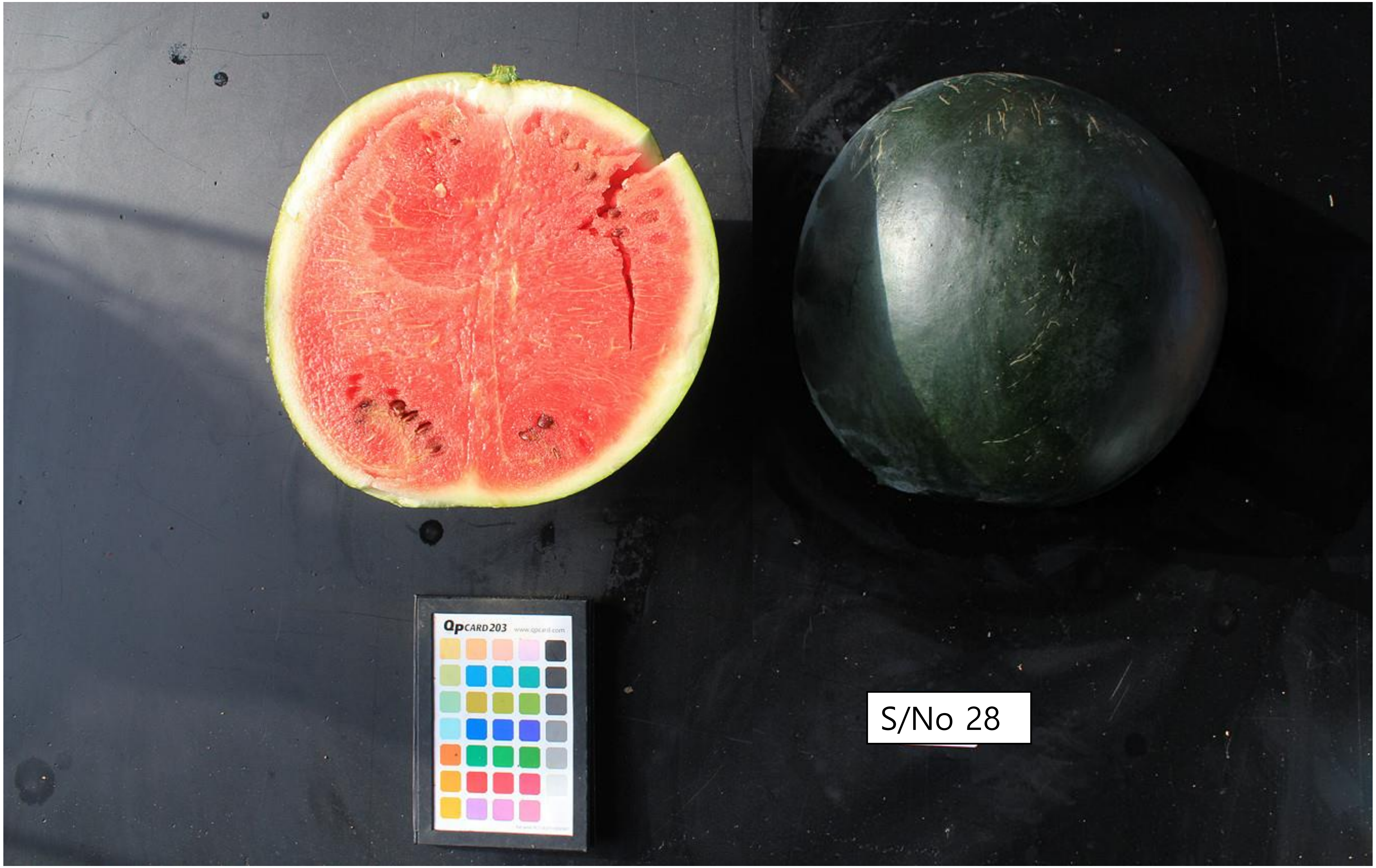
QpCARD 203
www.qpcard.com
S/No 28

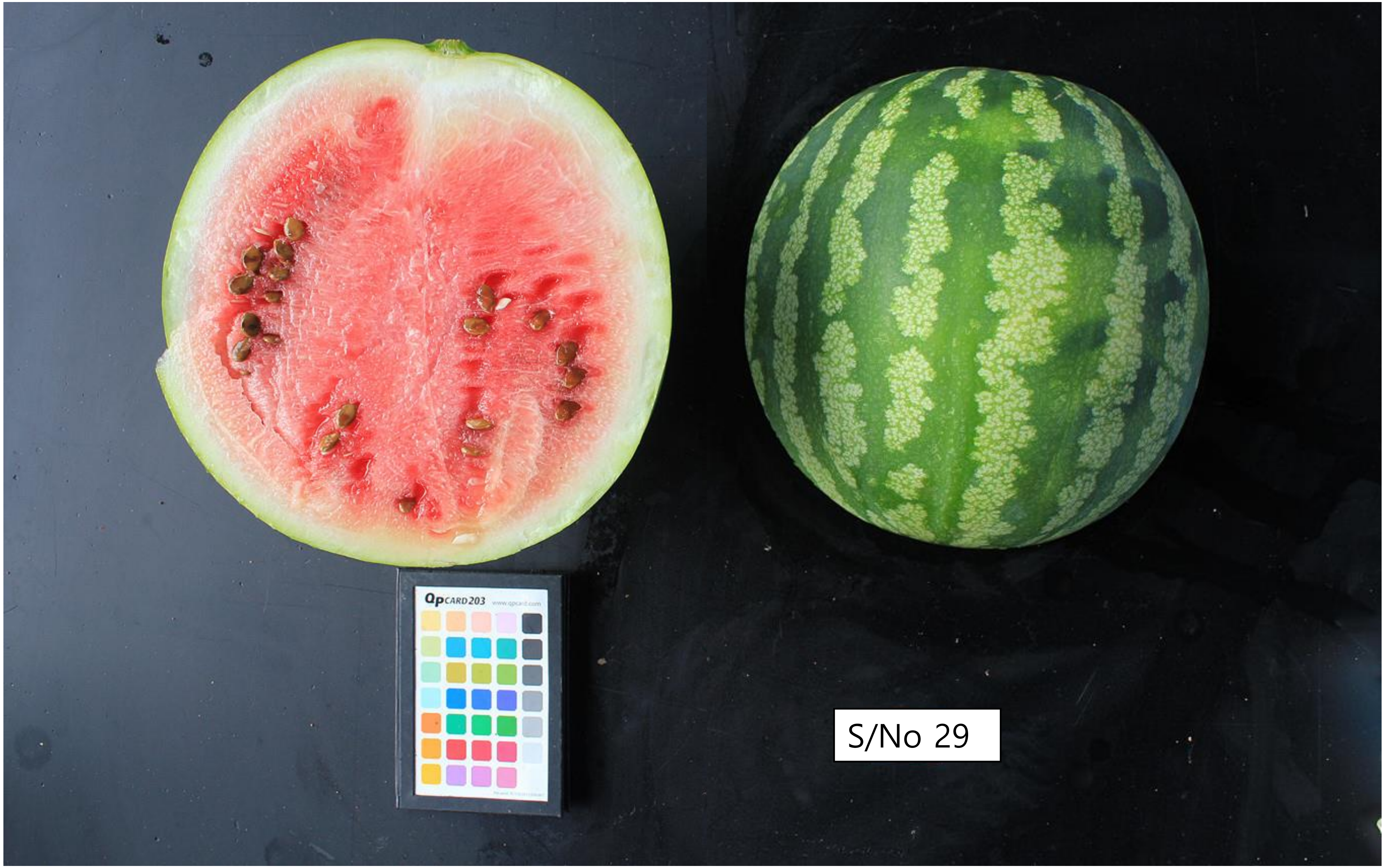
QpCARD 203
S/No 29

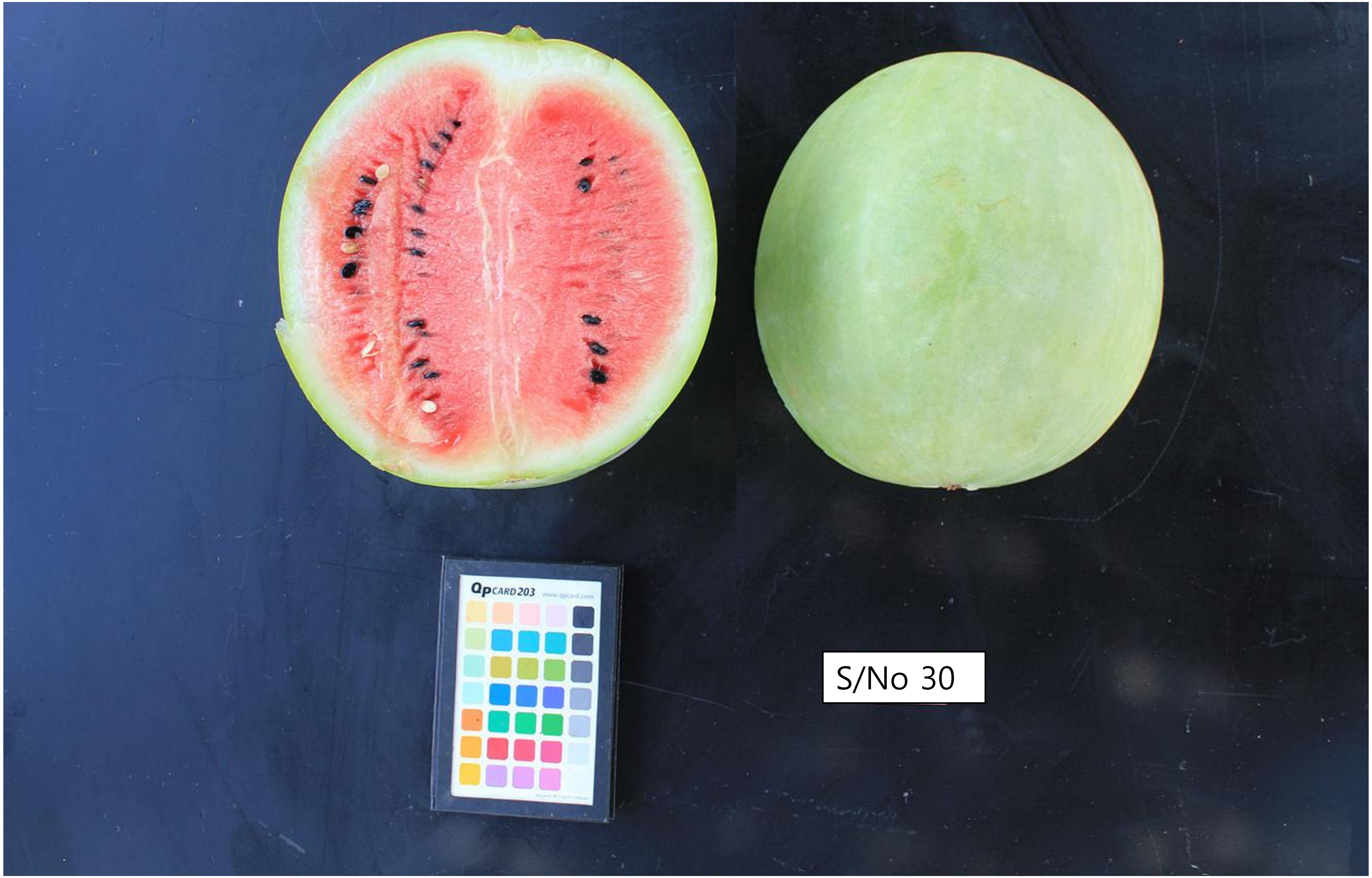
QpCARD 203
S/No 30

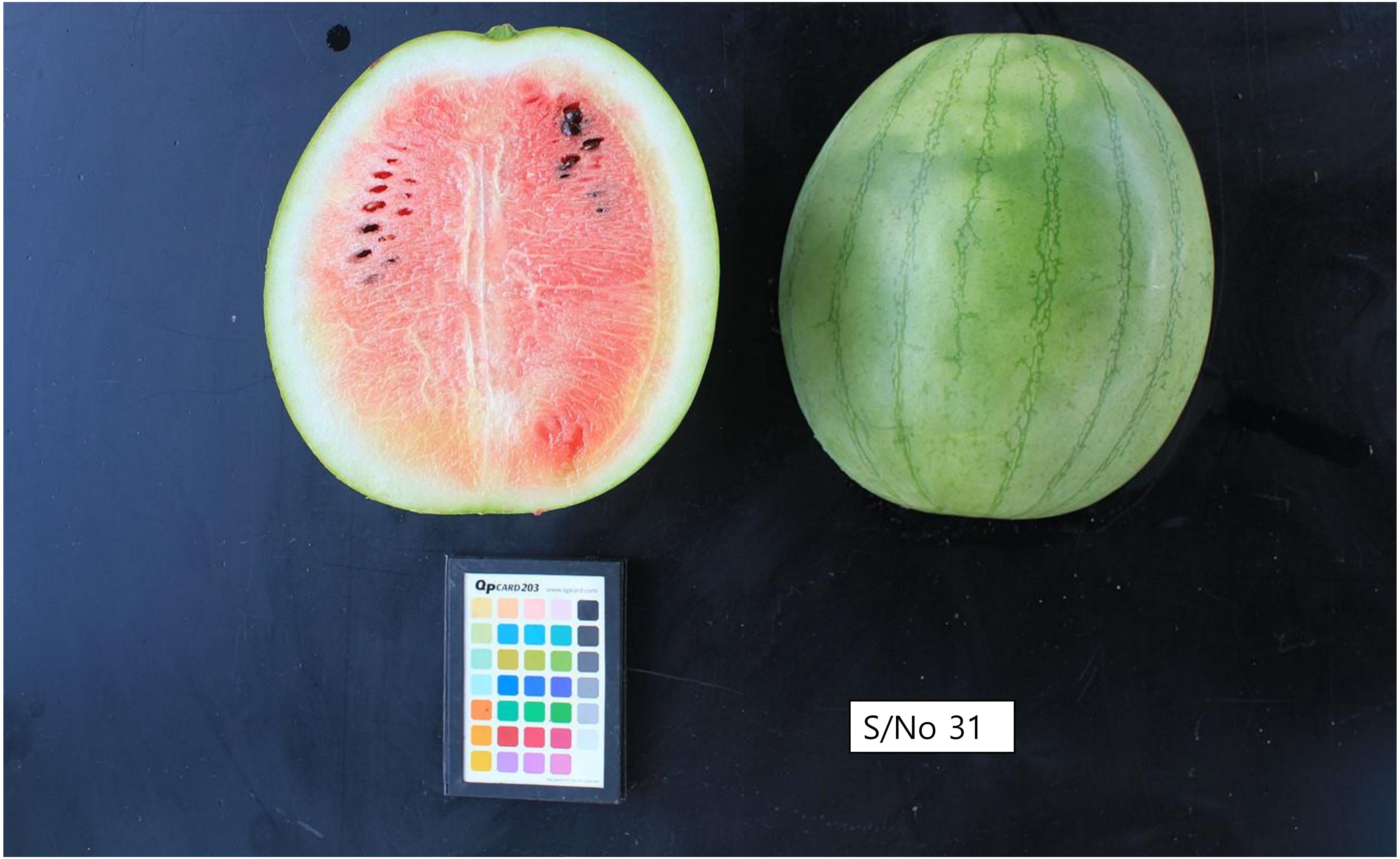
QpCARD 203
S/No 31

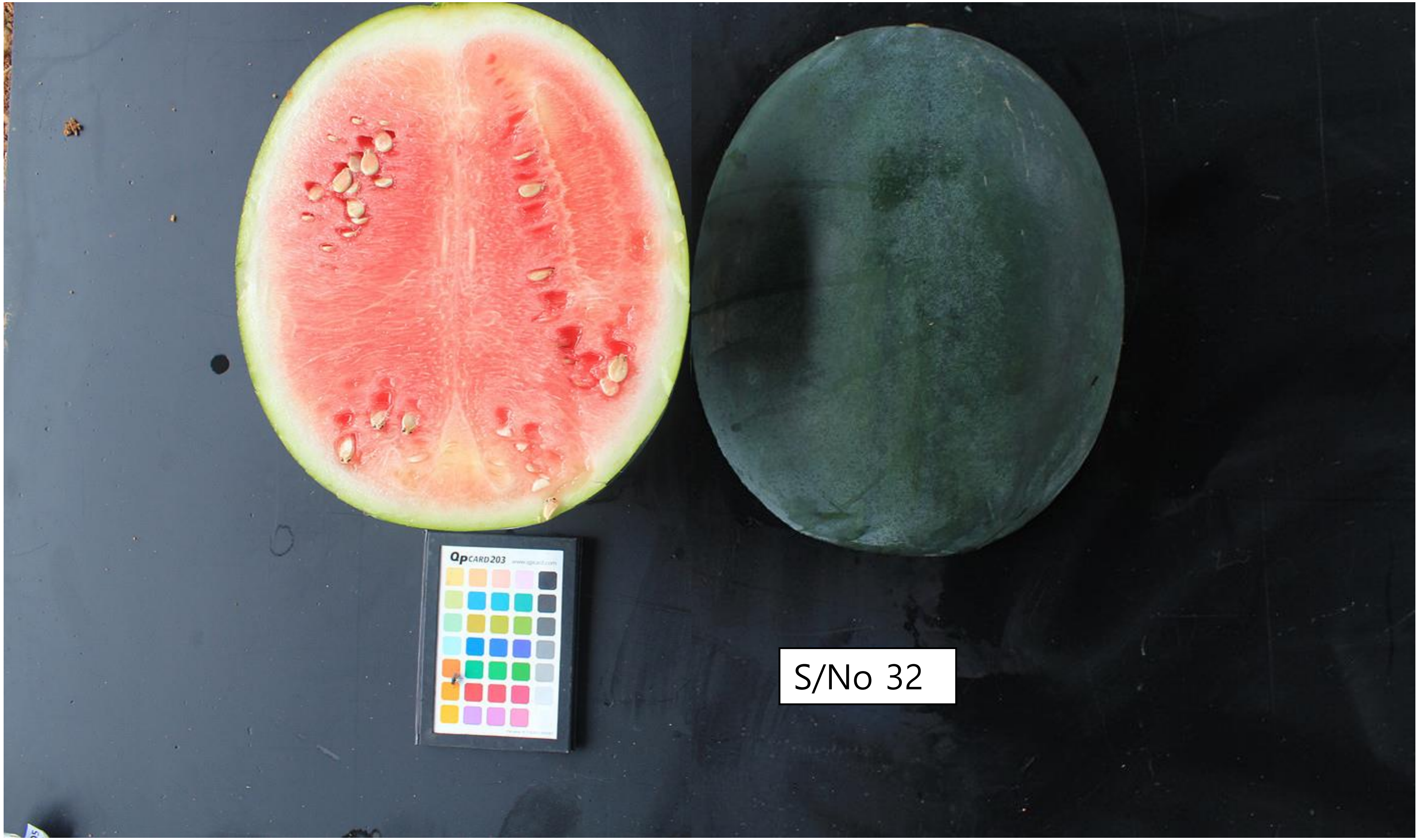
QpCARD203
S/No 32

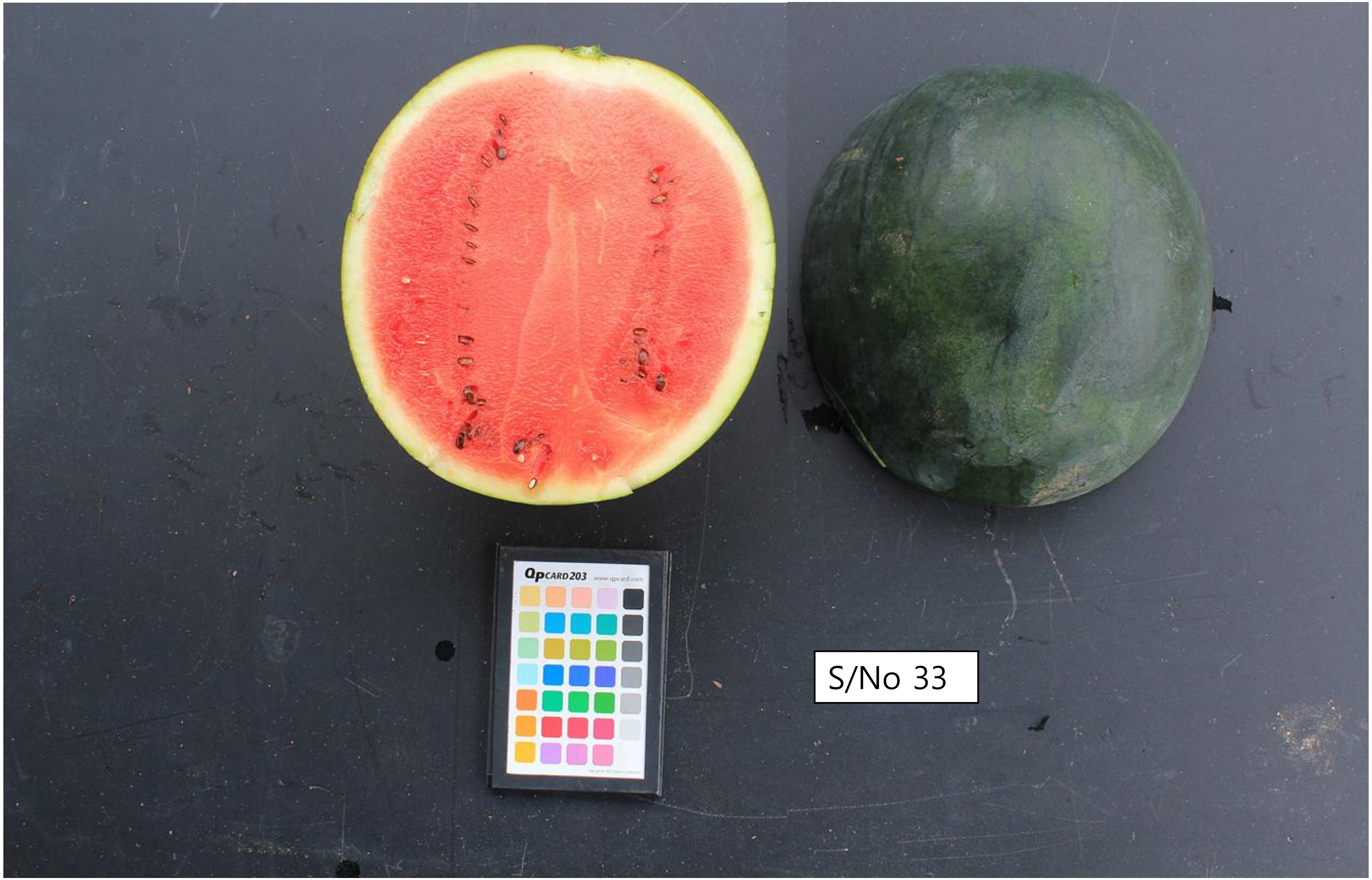
QpCARD 203
www.qpcard.com
S/No 33

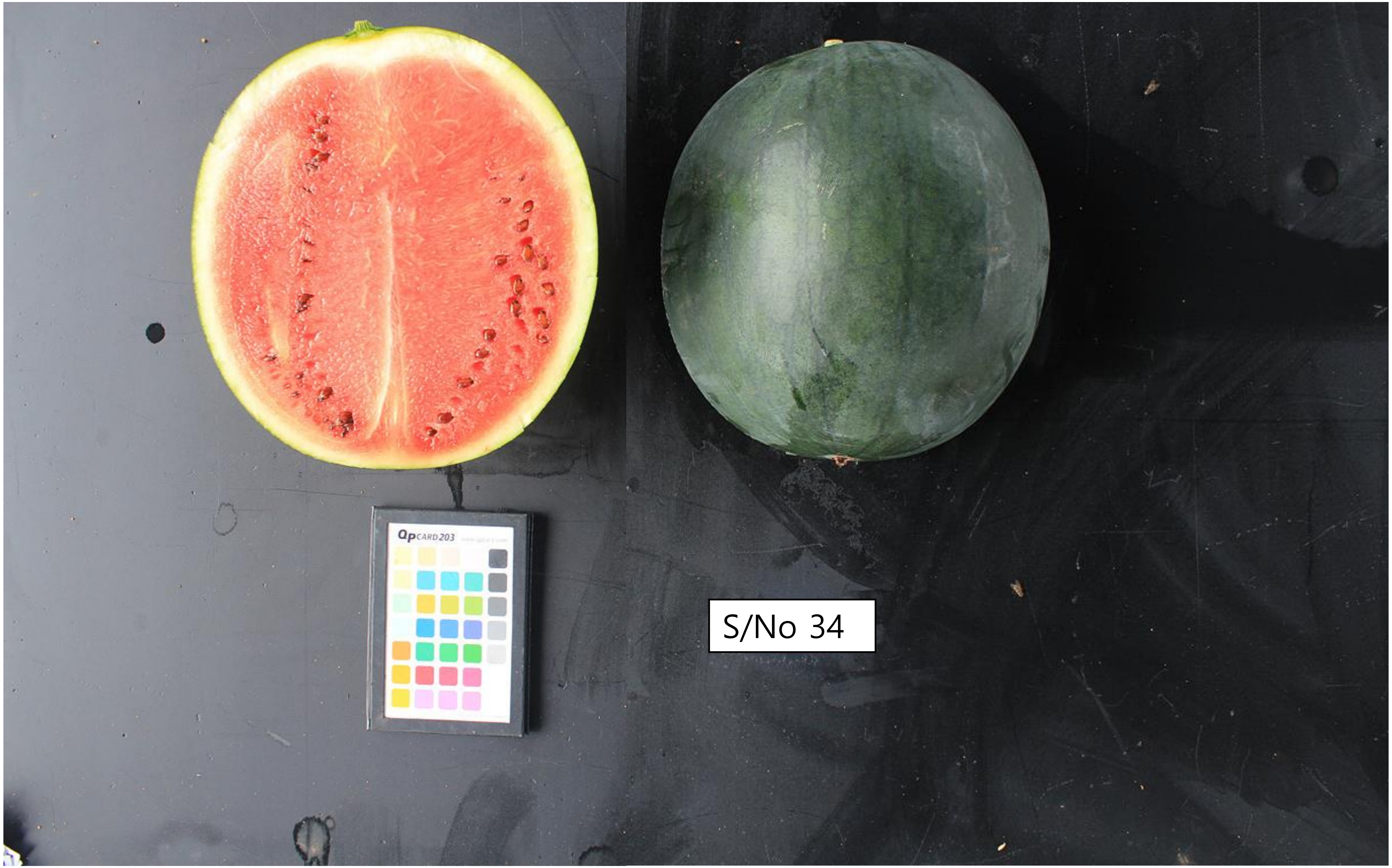
QpCARD 203
S/No 34

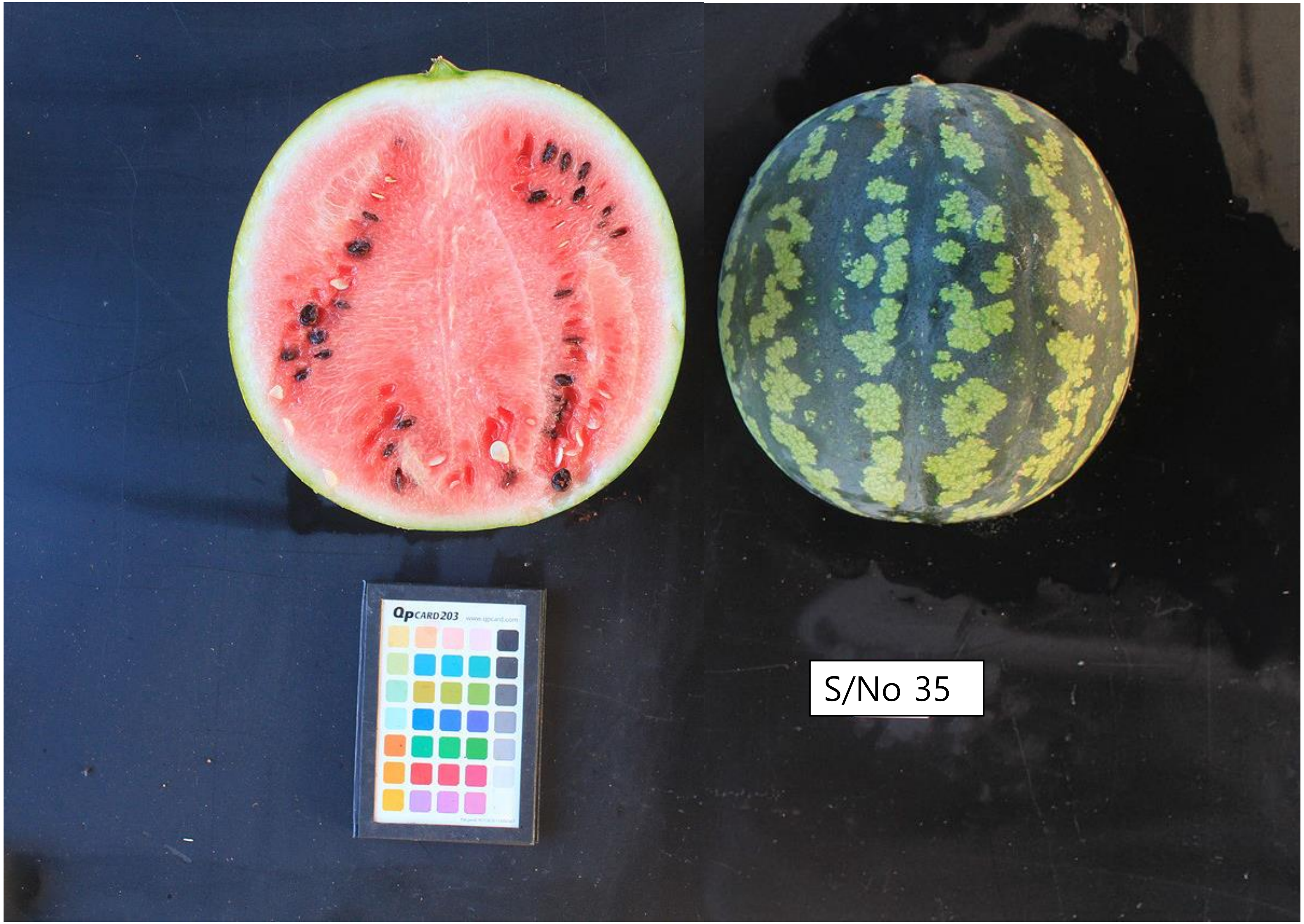

S/No 35

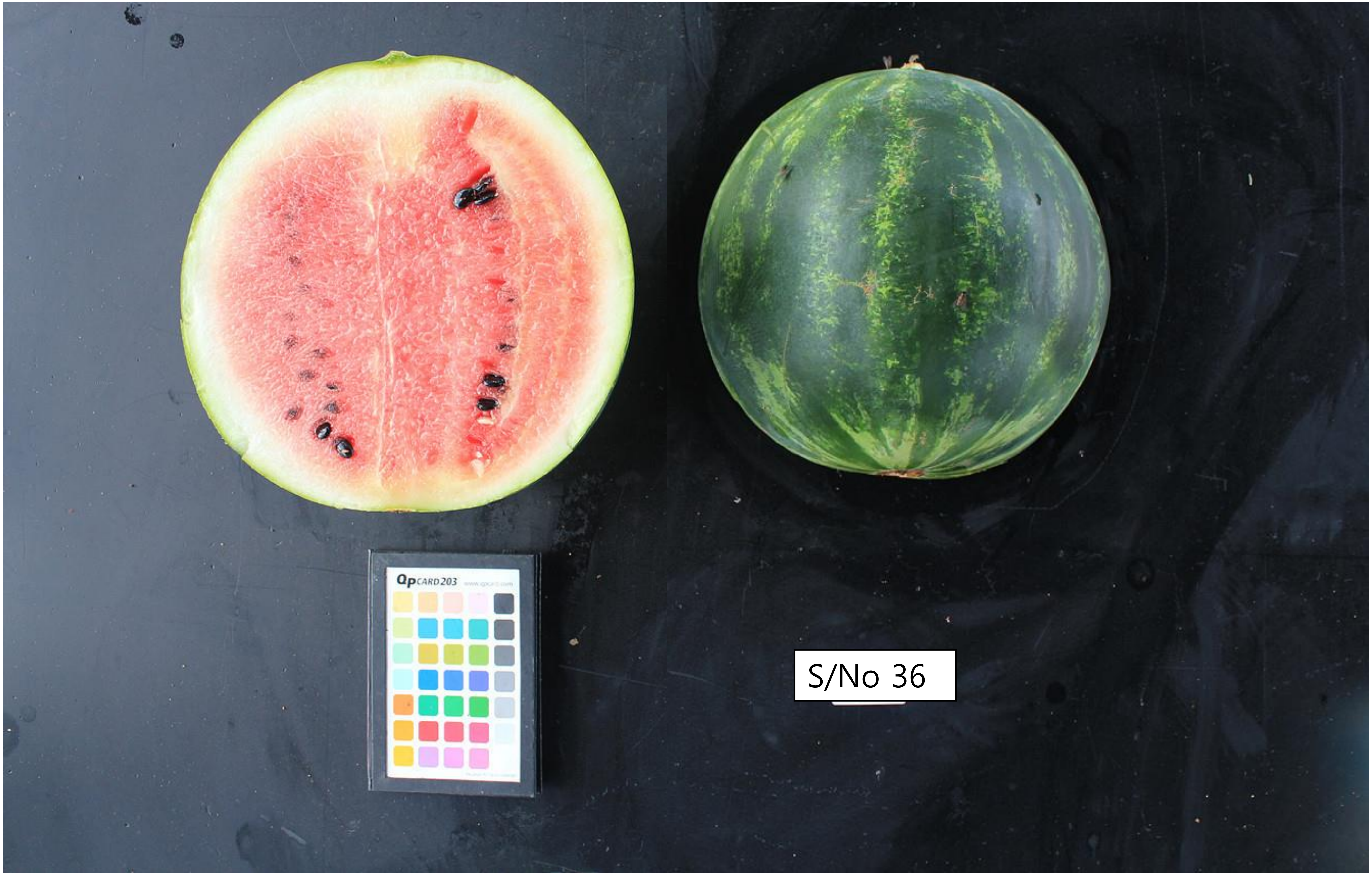
QpCARD 203
S/No 36

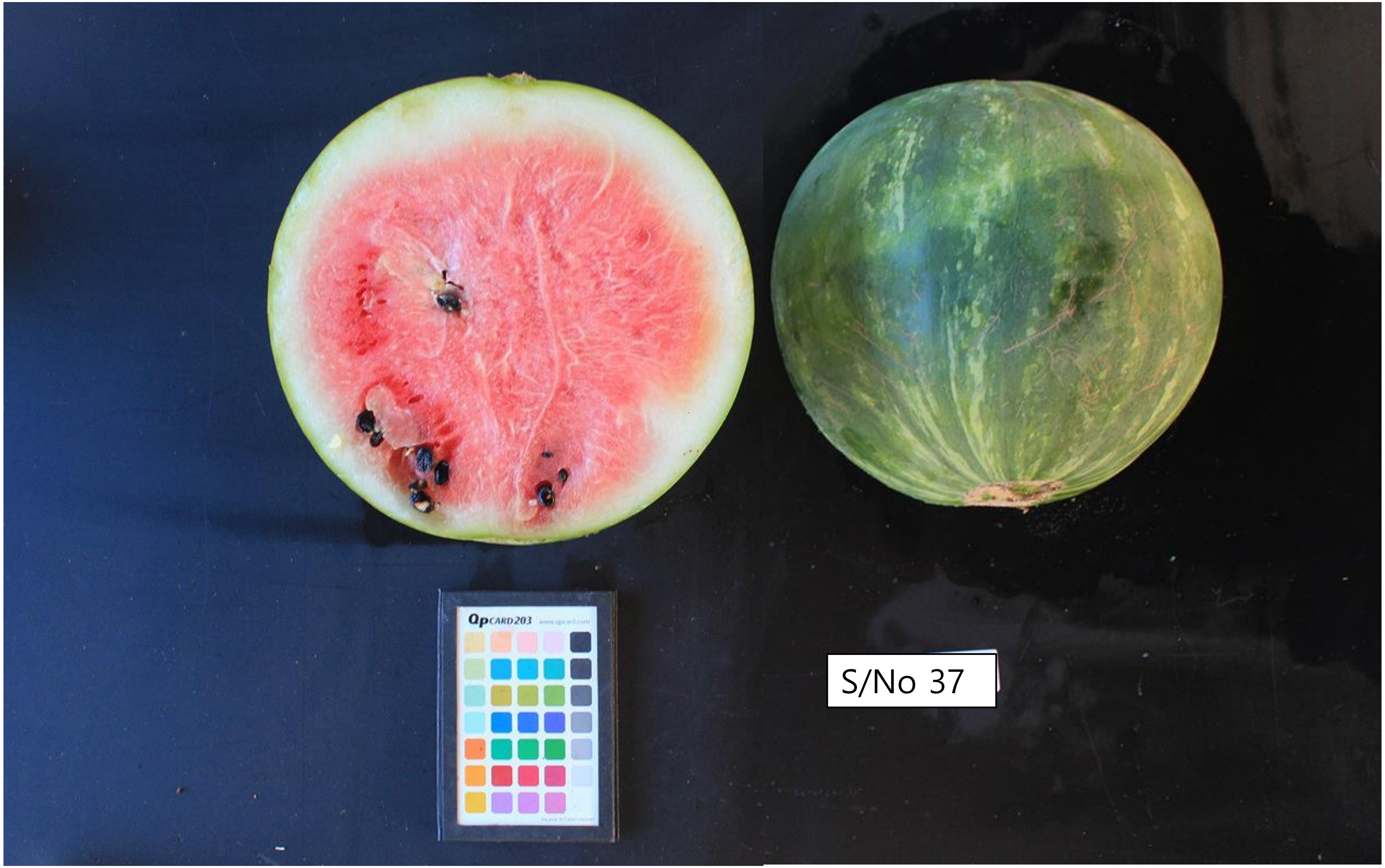
QpCARD 203
S/No 37

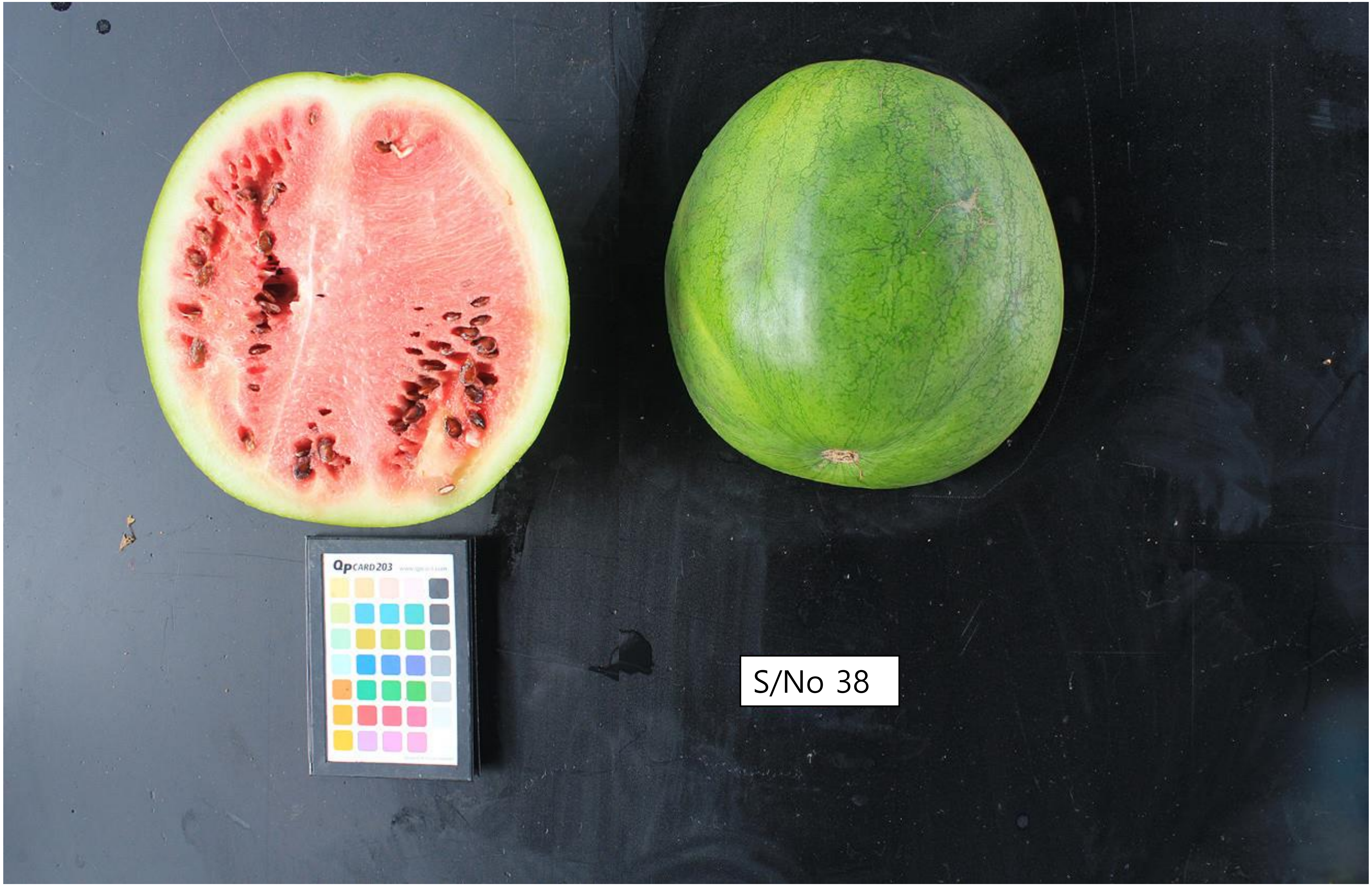
QpCARD203
S/No 38

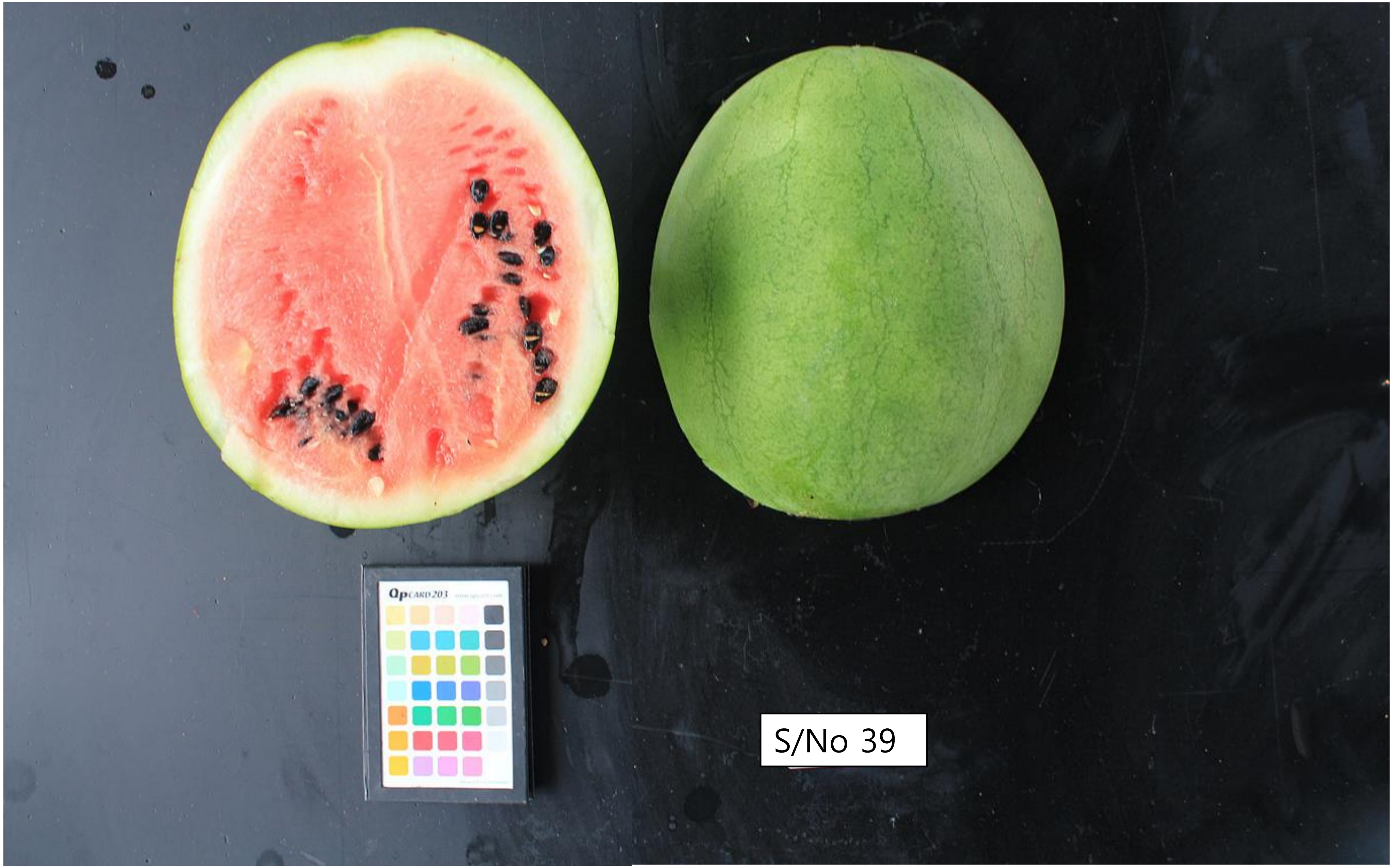
QpCARD203
S/No 39

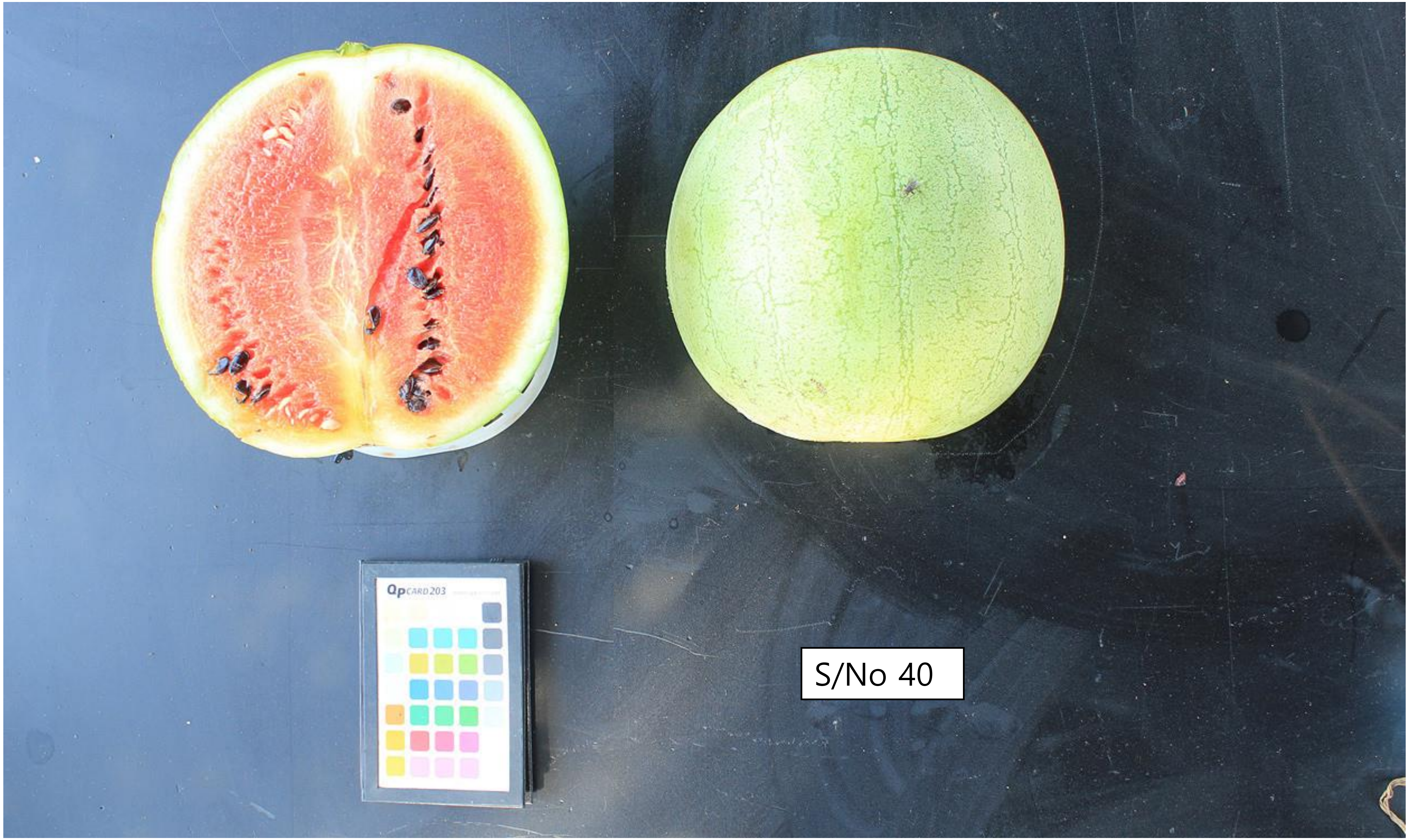
QpCARD 203
S/No 40

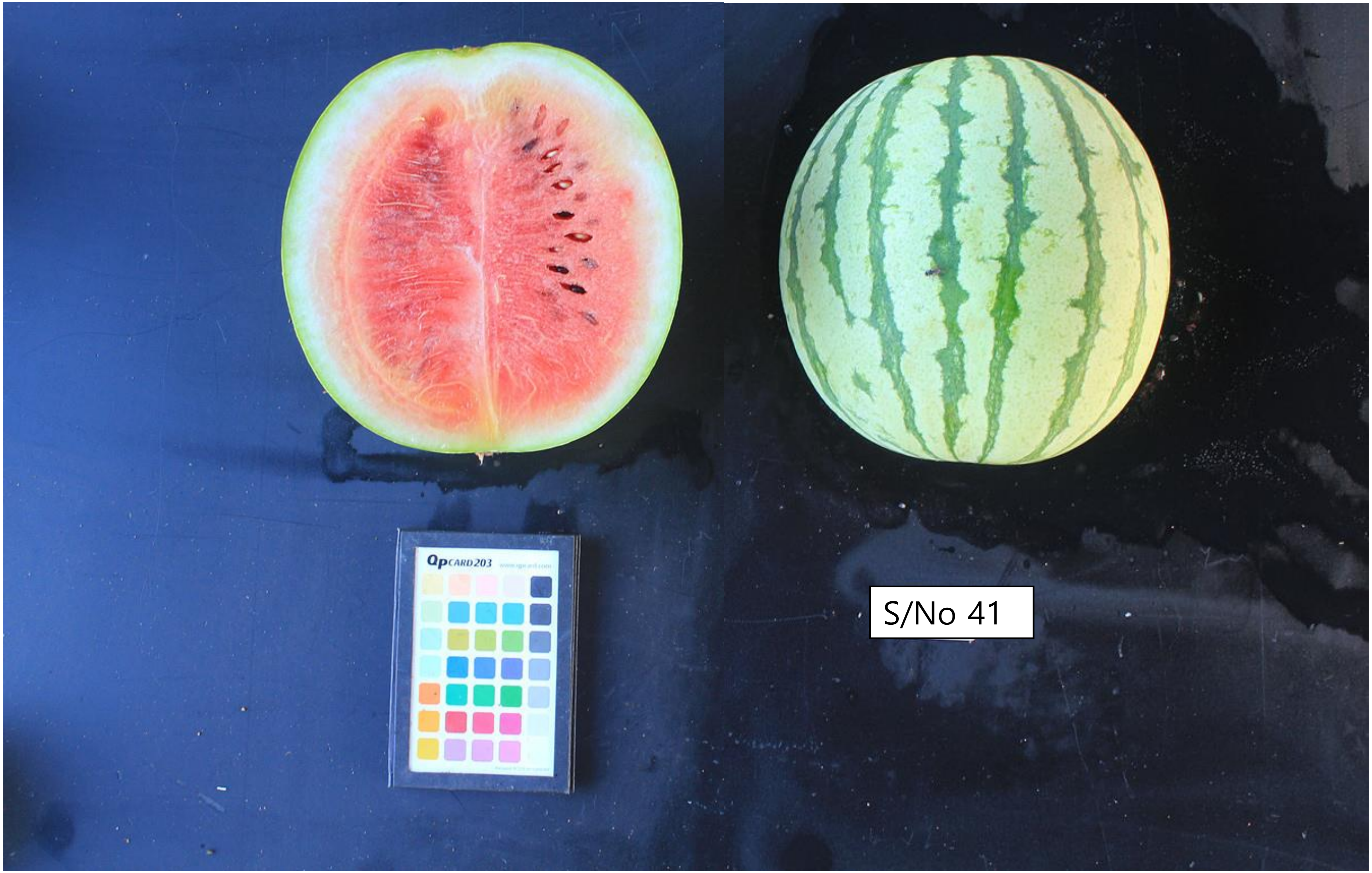
QpCARD203
S/No 41

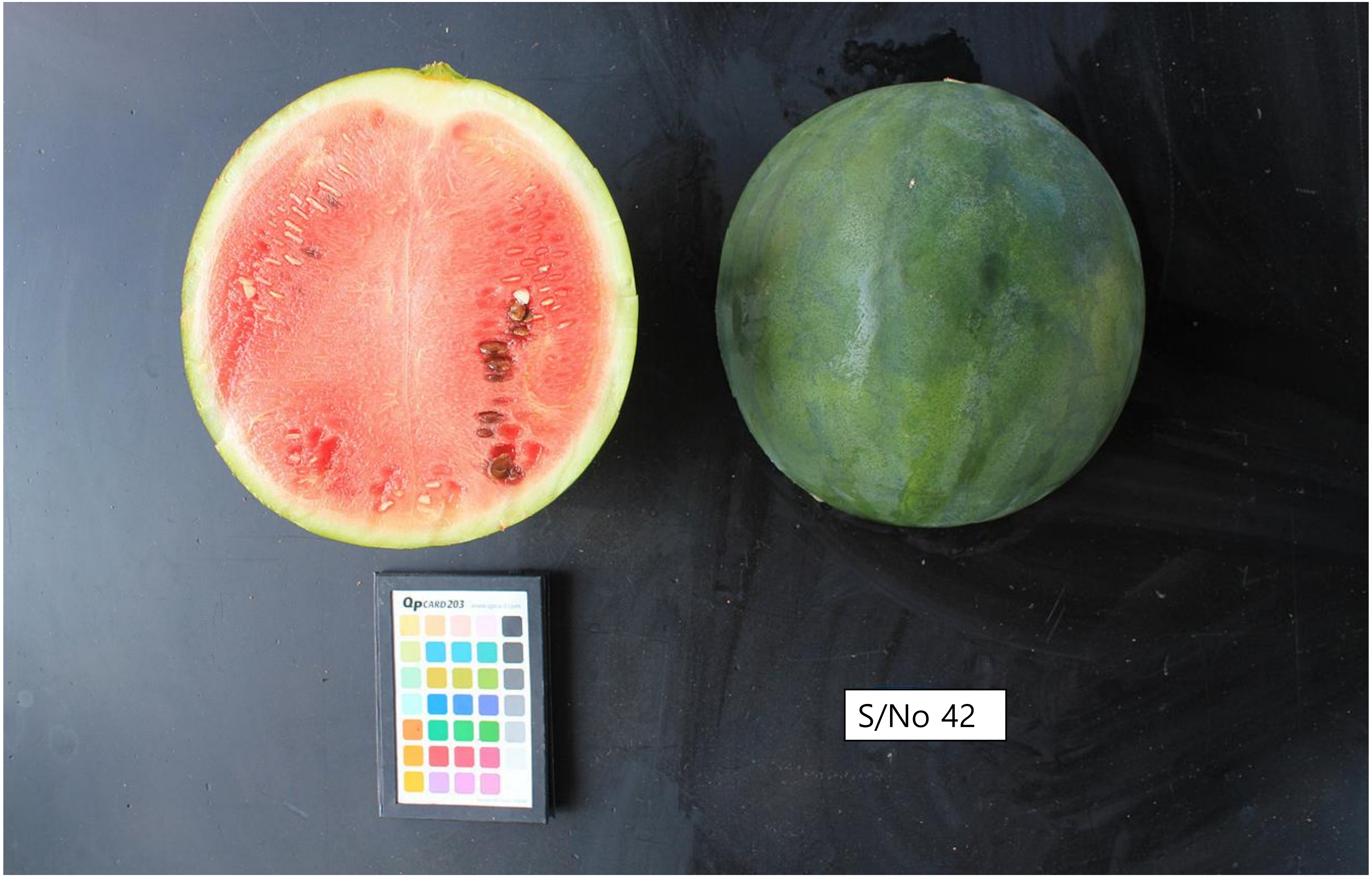
QpCARD 203
S/No 42

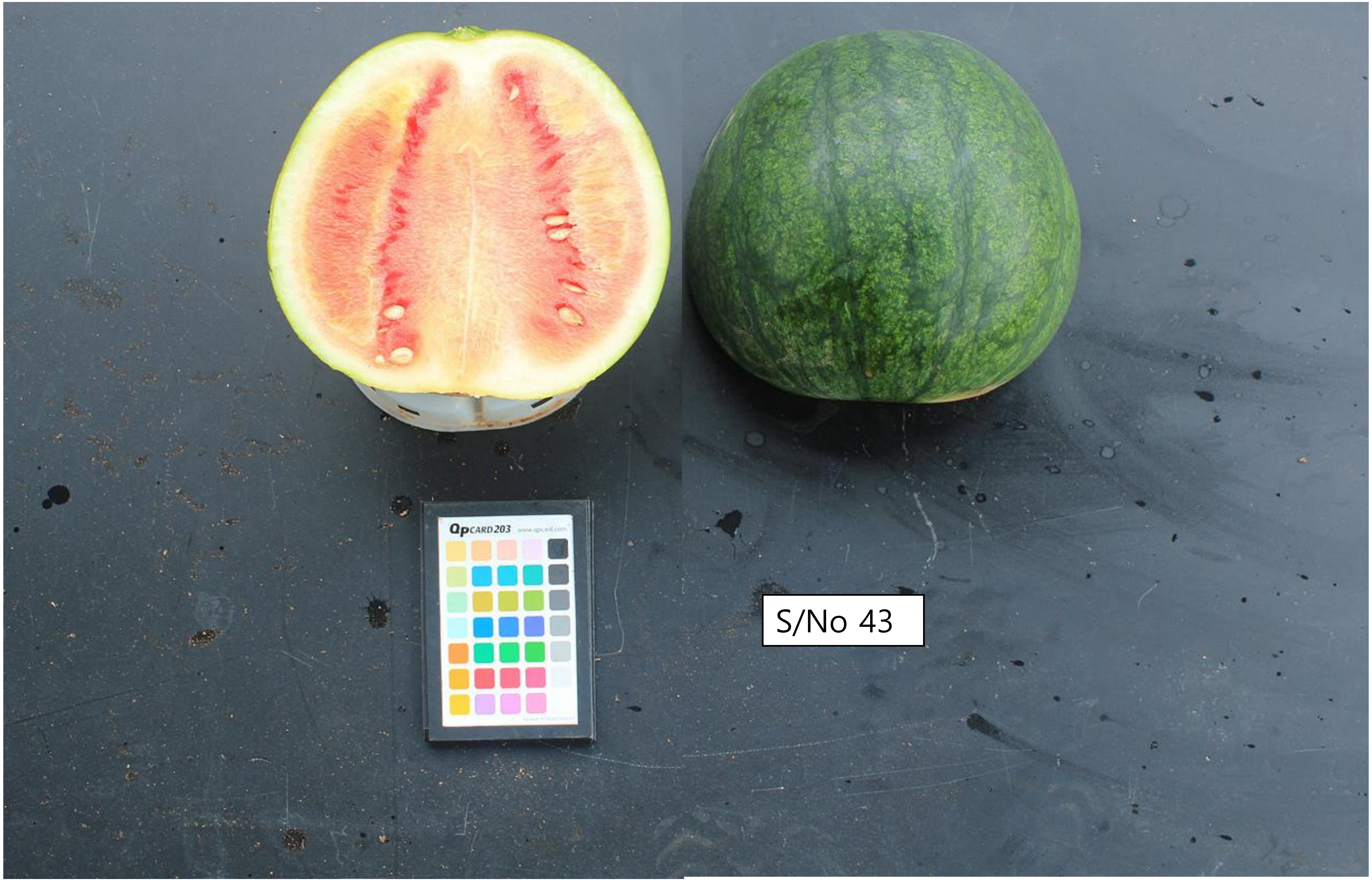
S/No 43

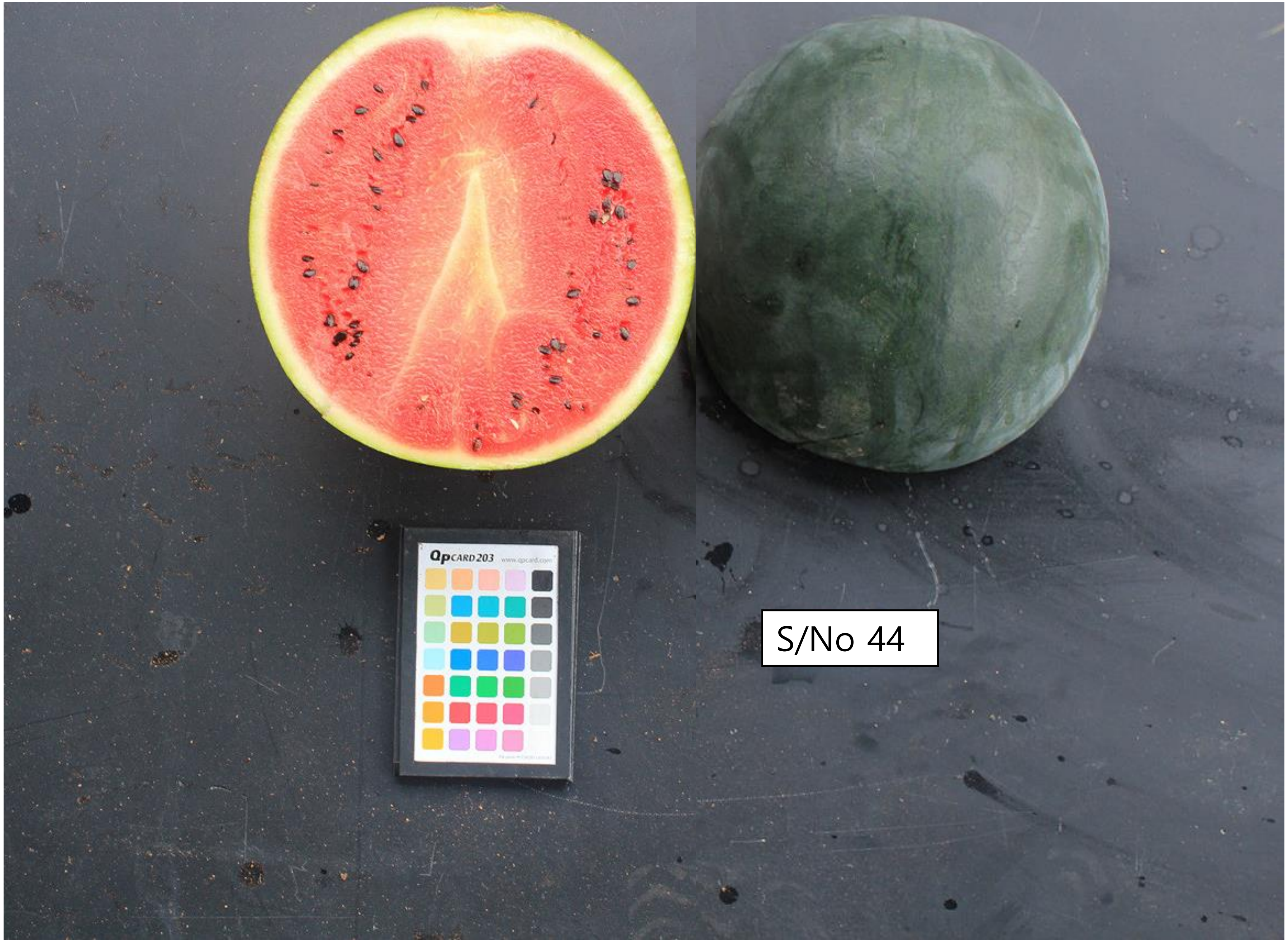
QpCARD 203
S/No 44

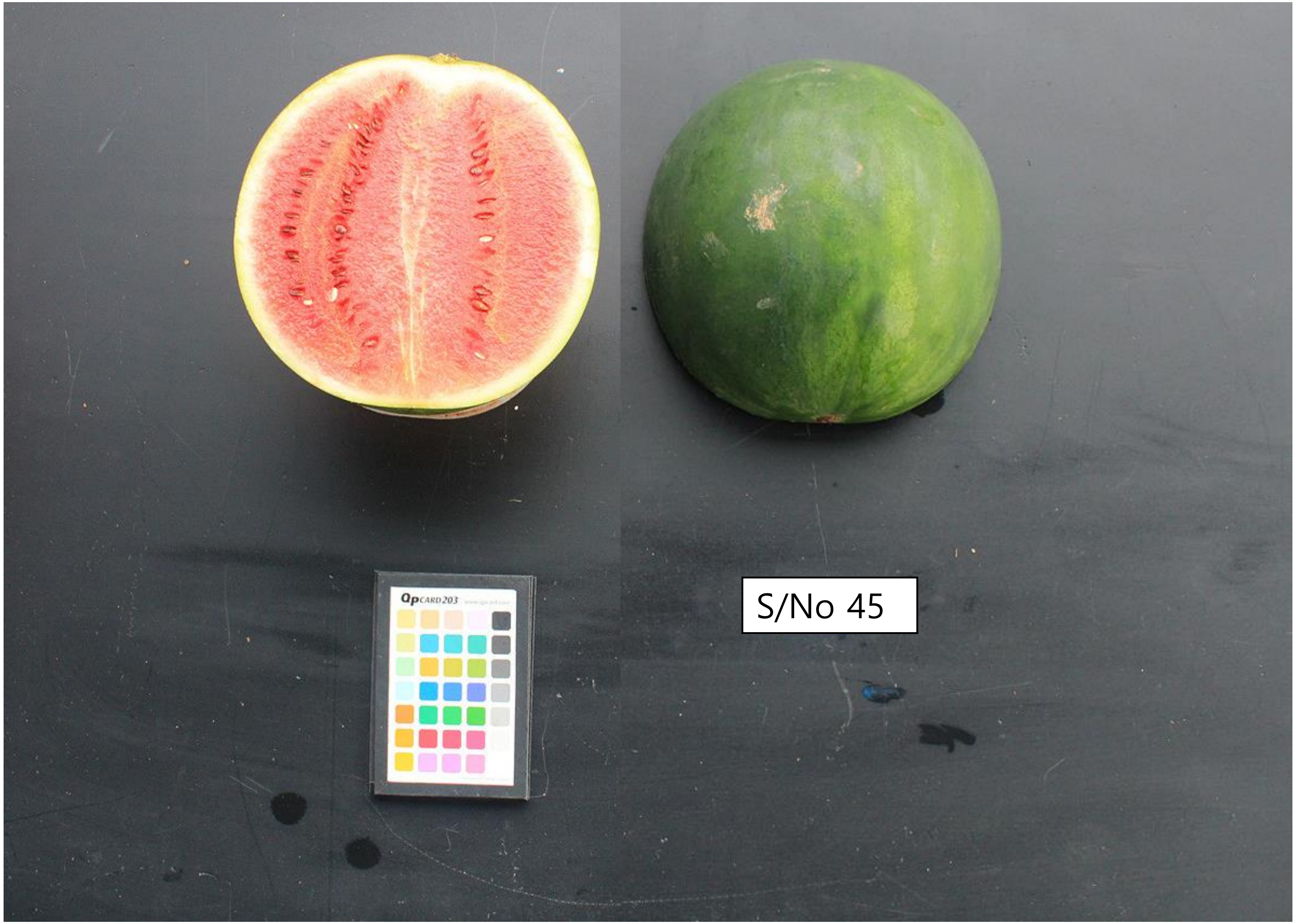
QpCARD 203
S/No 45

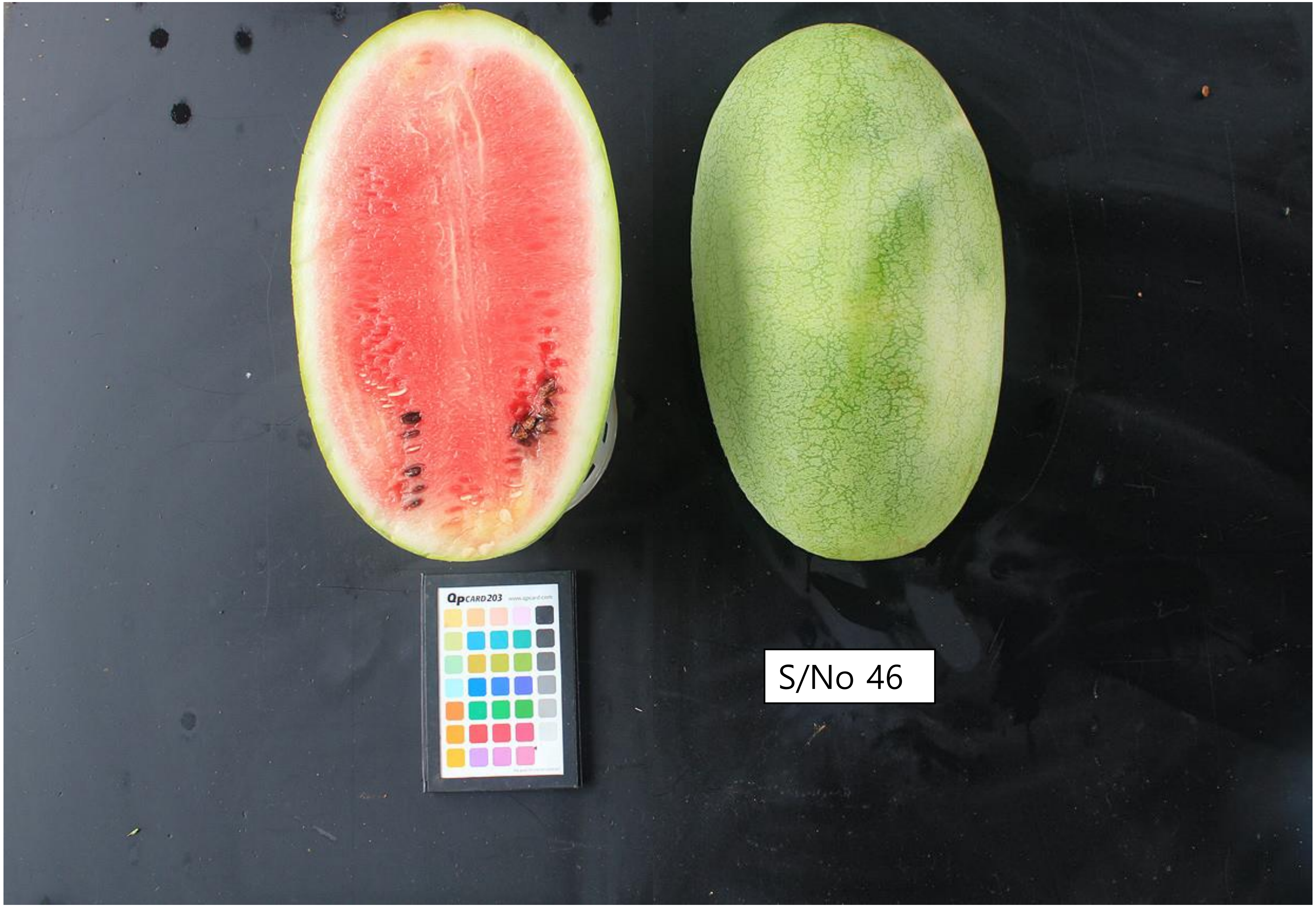
QpCARD203
S/No 46

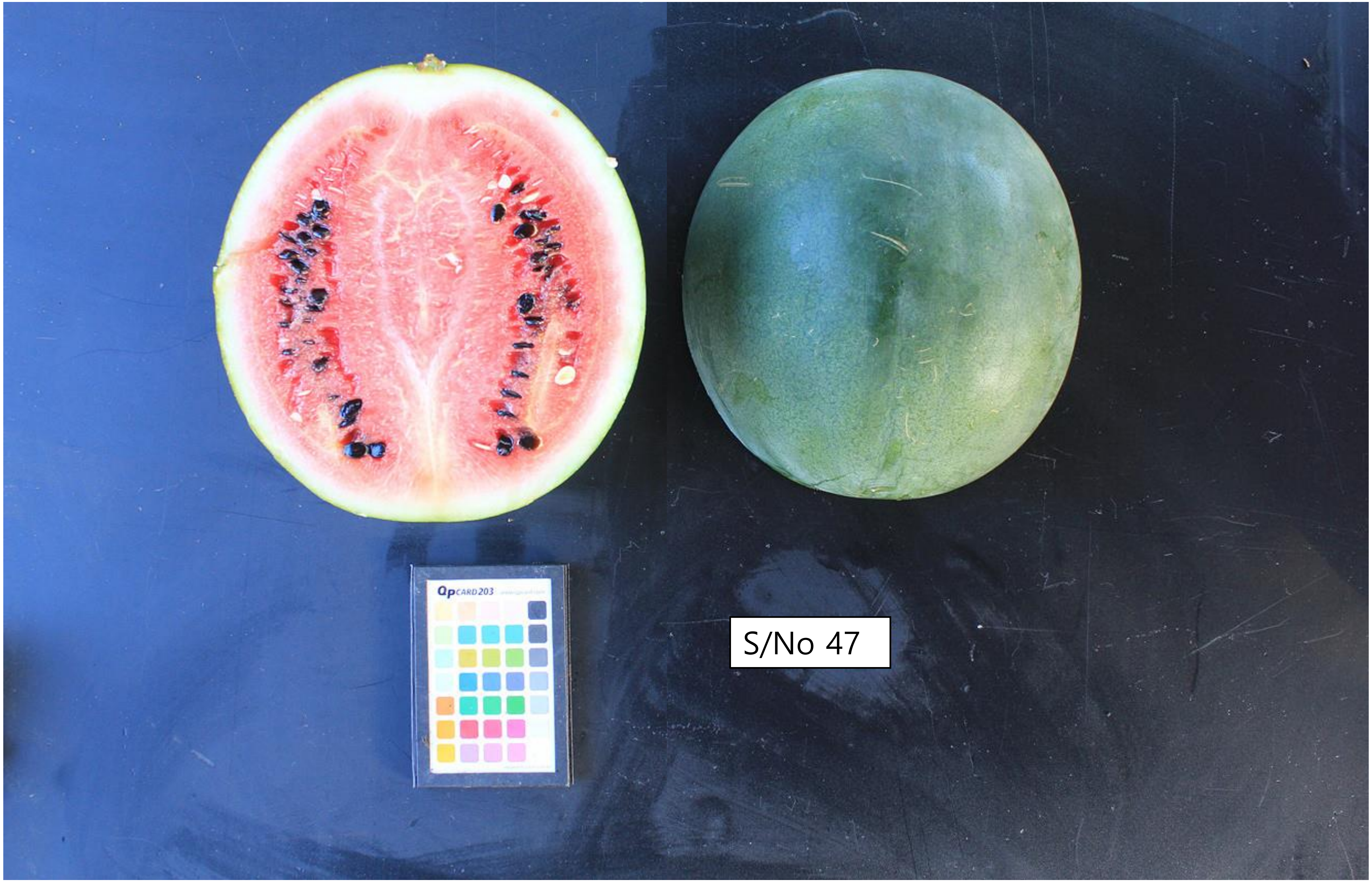
Qp CARD 203
S/No 47

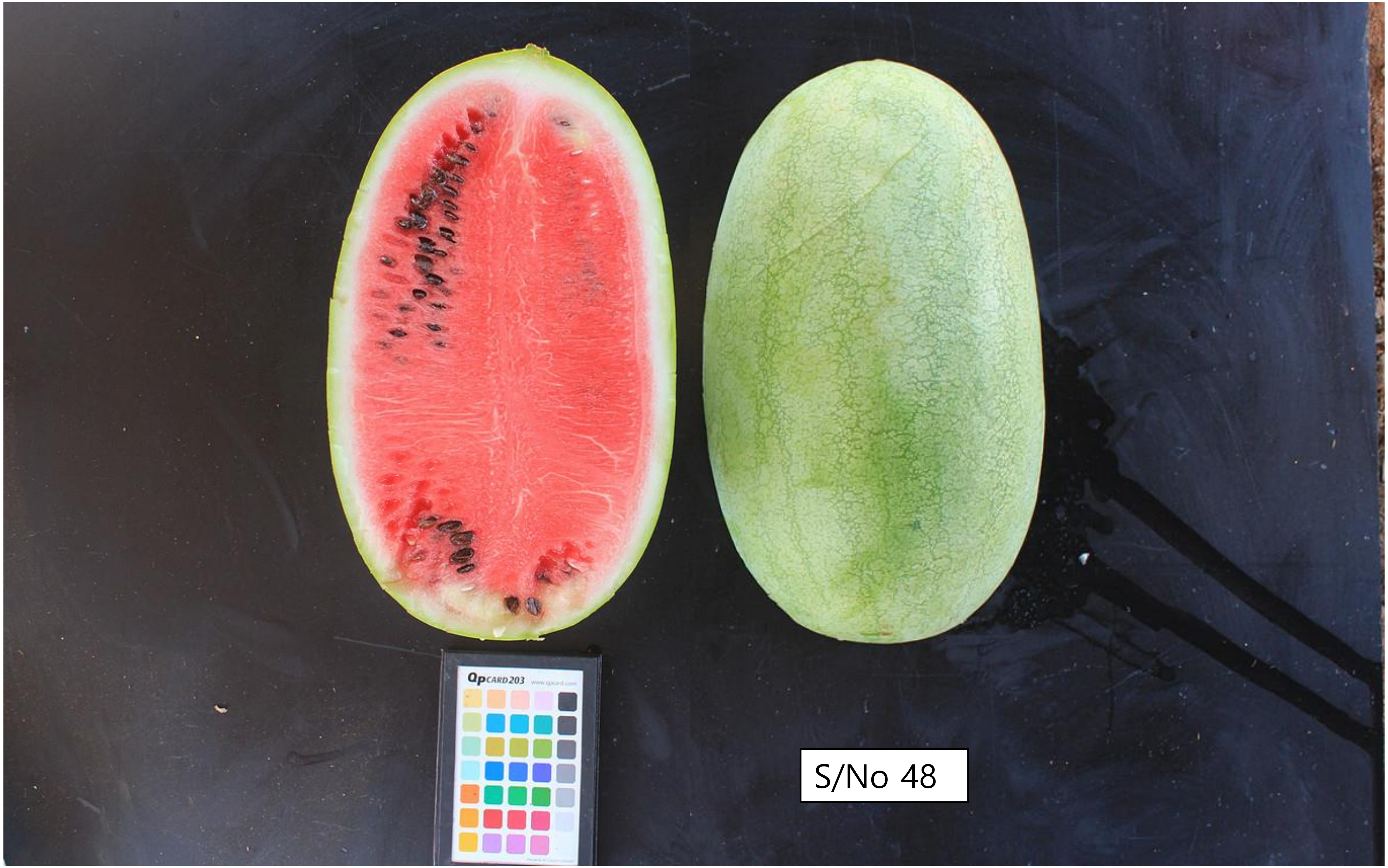
QpCARD 203
S/No 48

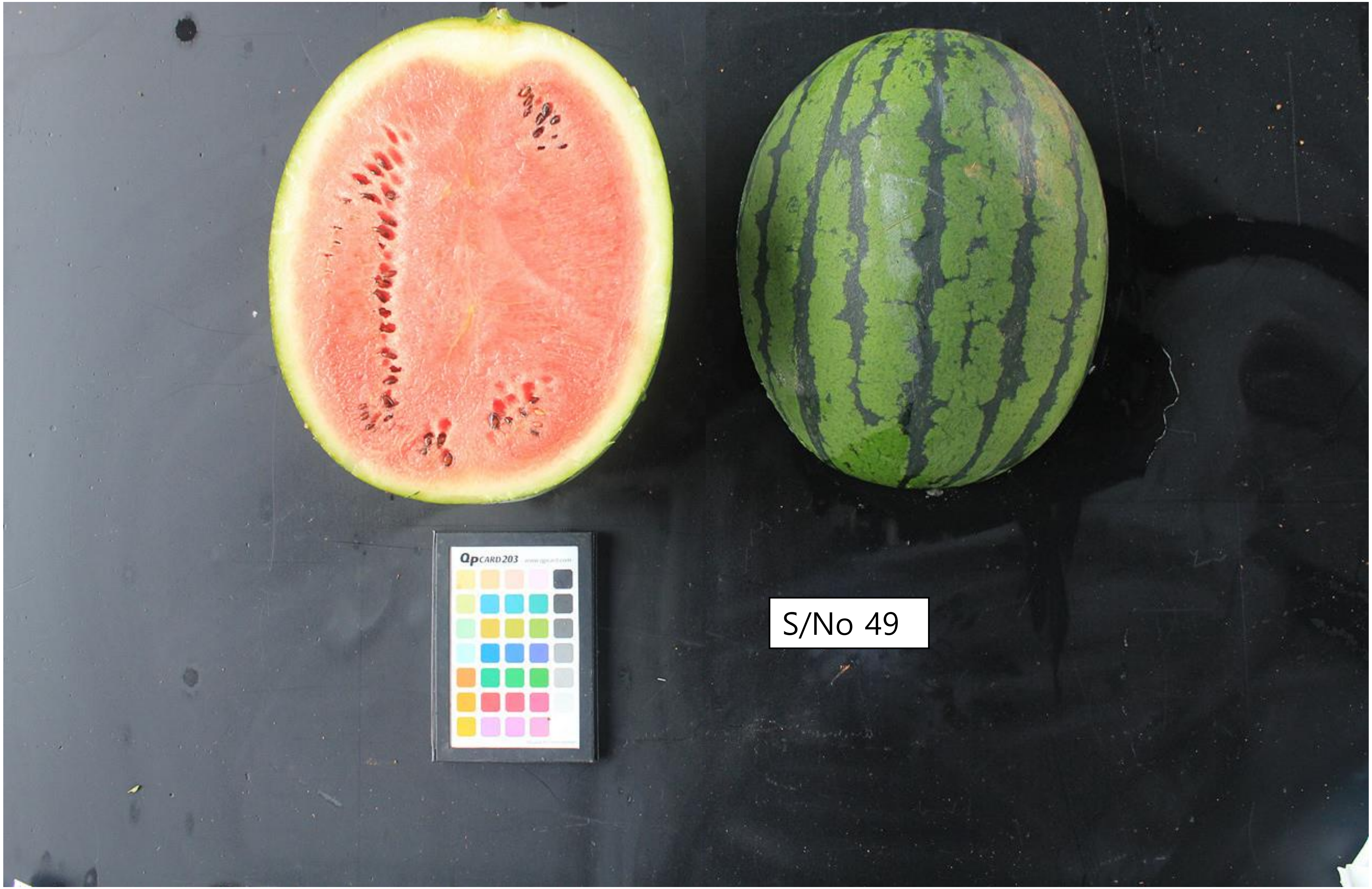
QpCARD203
S/No 49

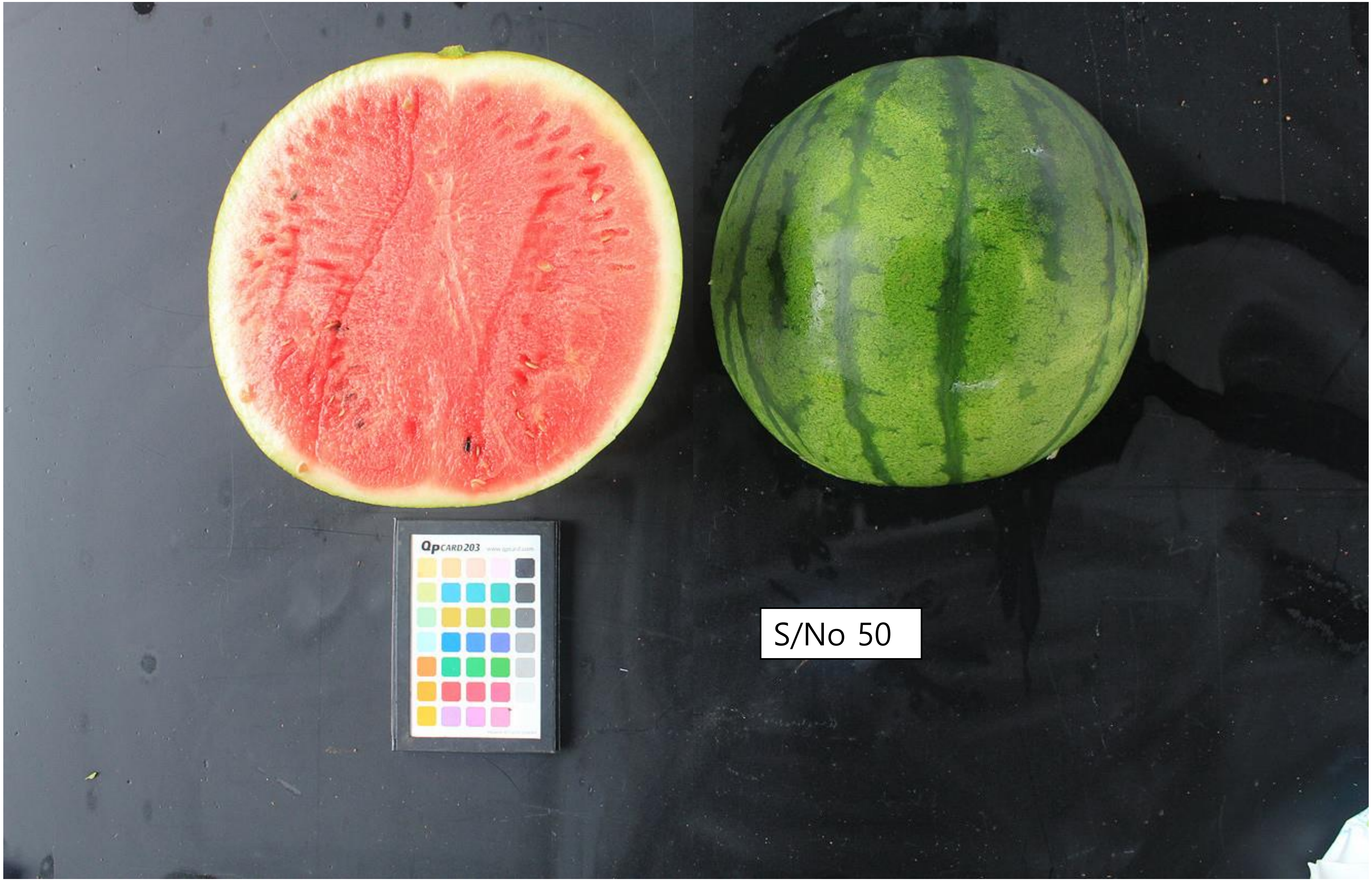
QpCARD203
S/No 50

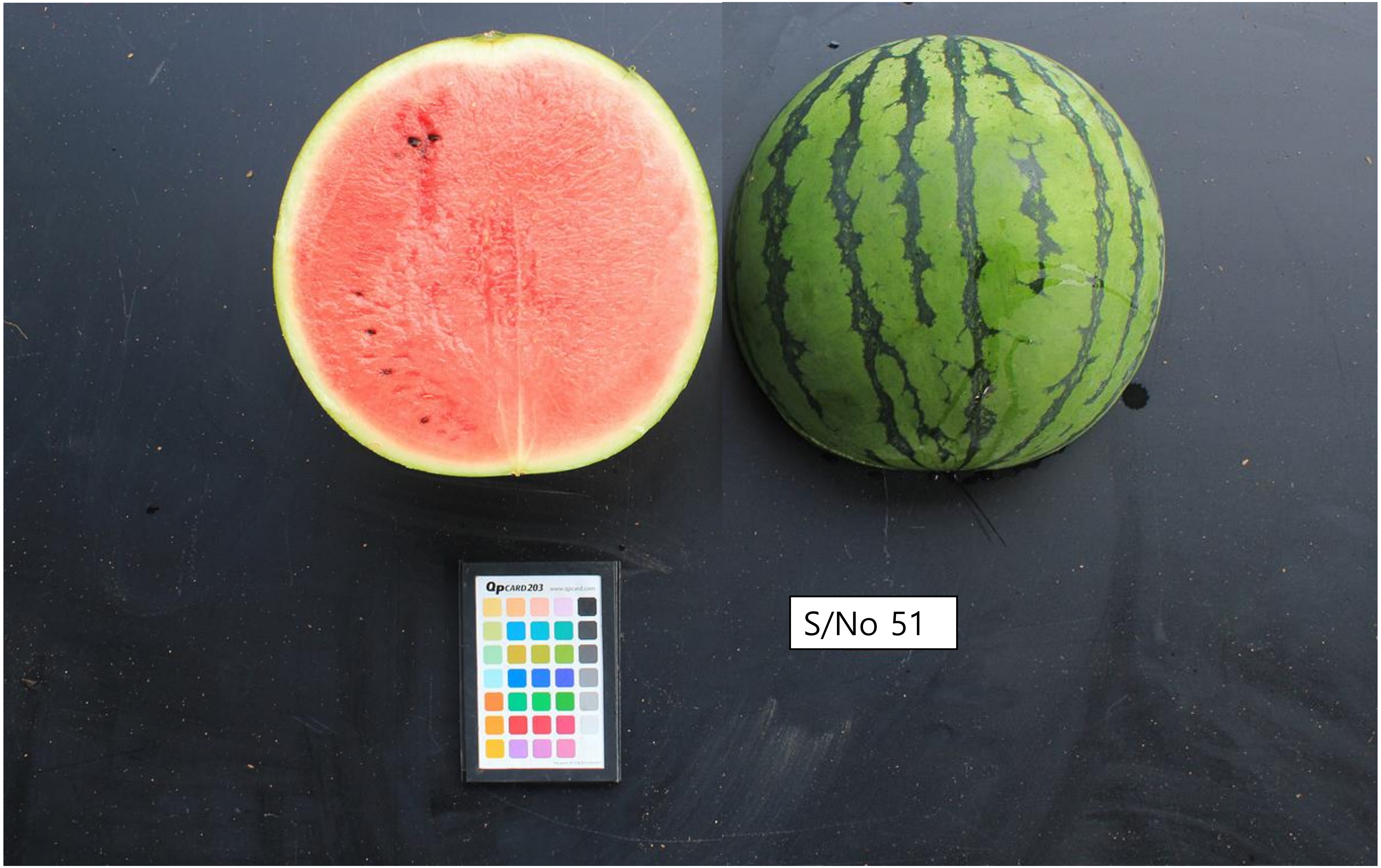
QpCARD203
S/No 51

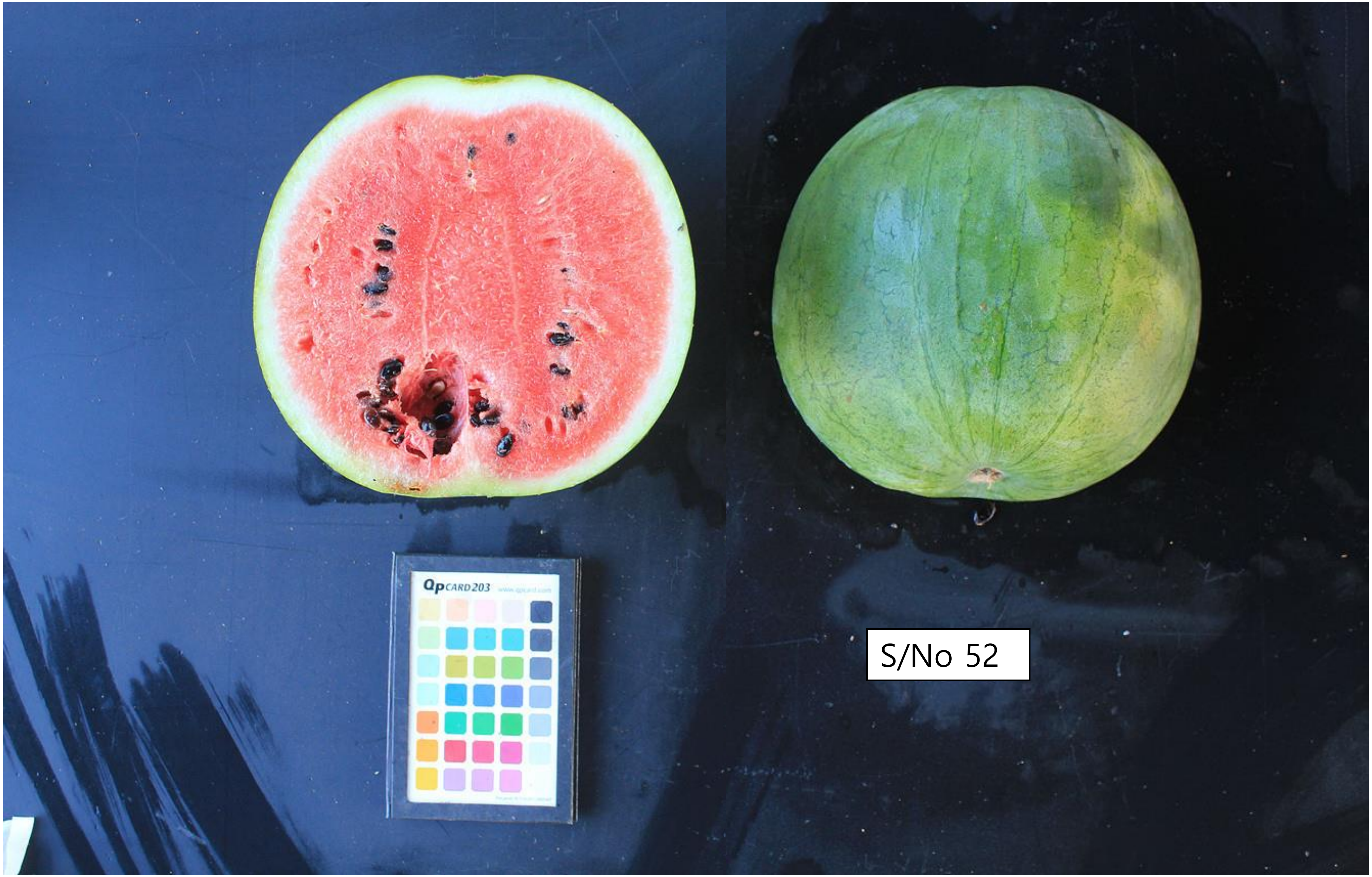
Qp CARD 203
S/No 52

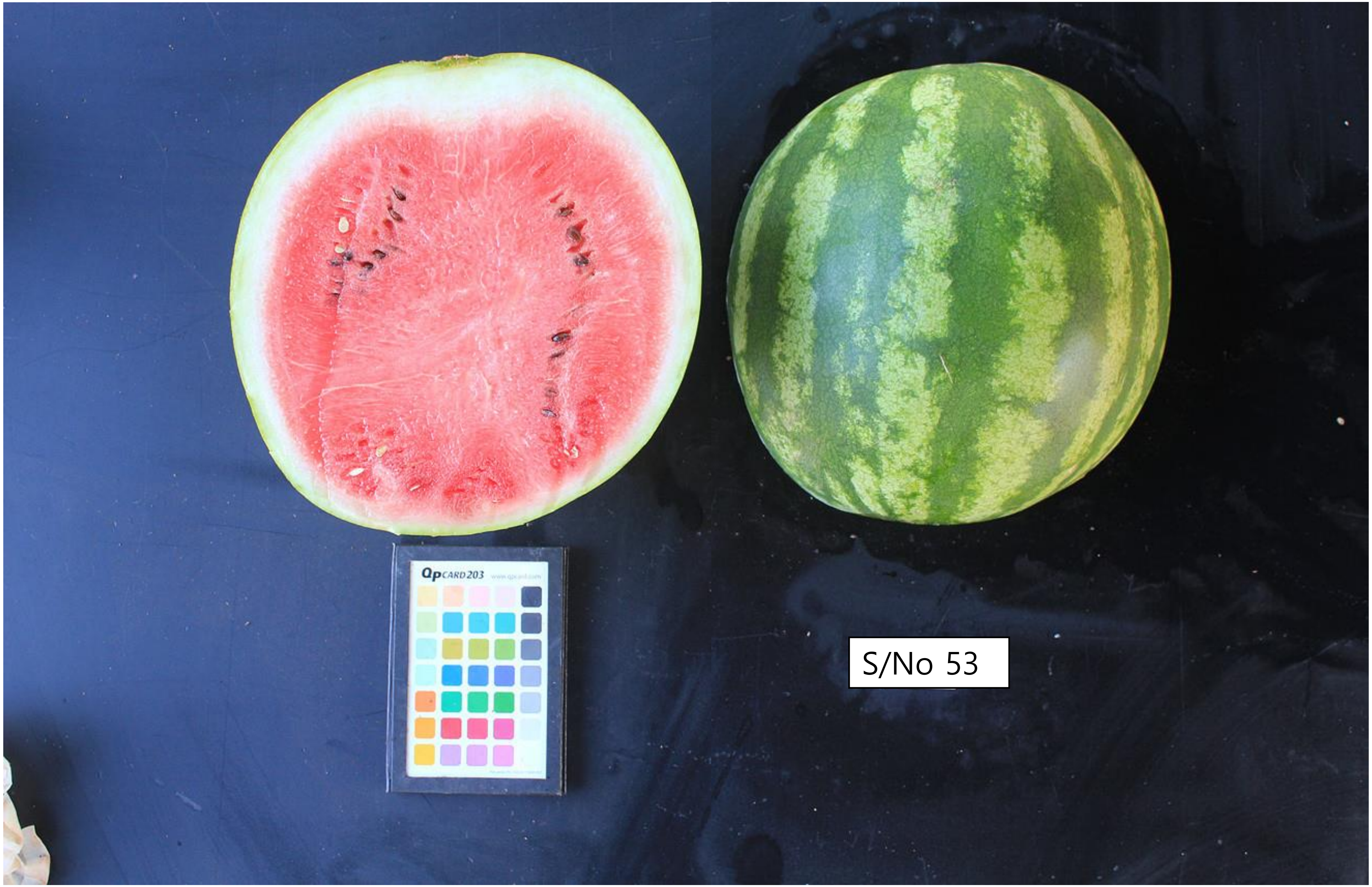
QpCARD 203
S/No 53

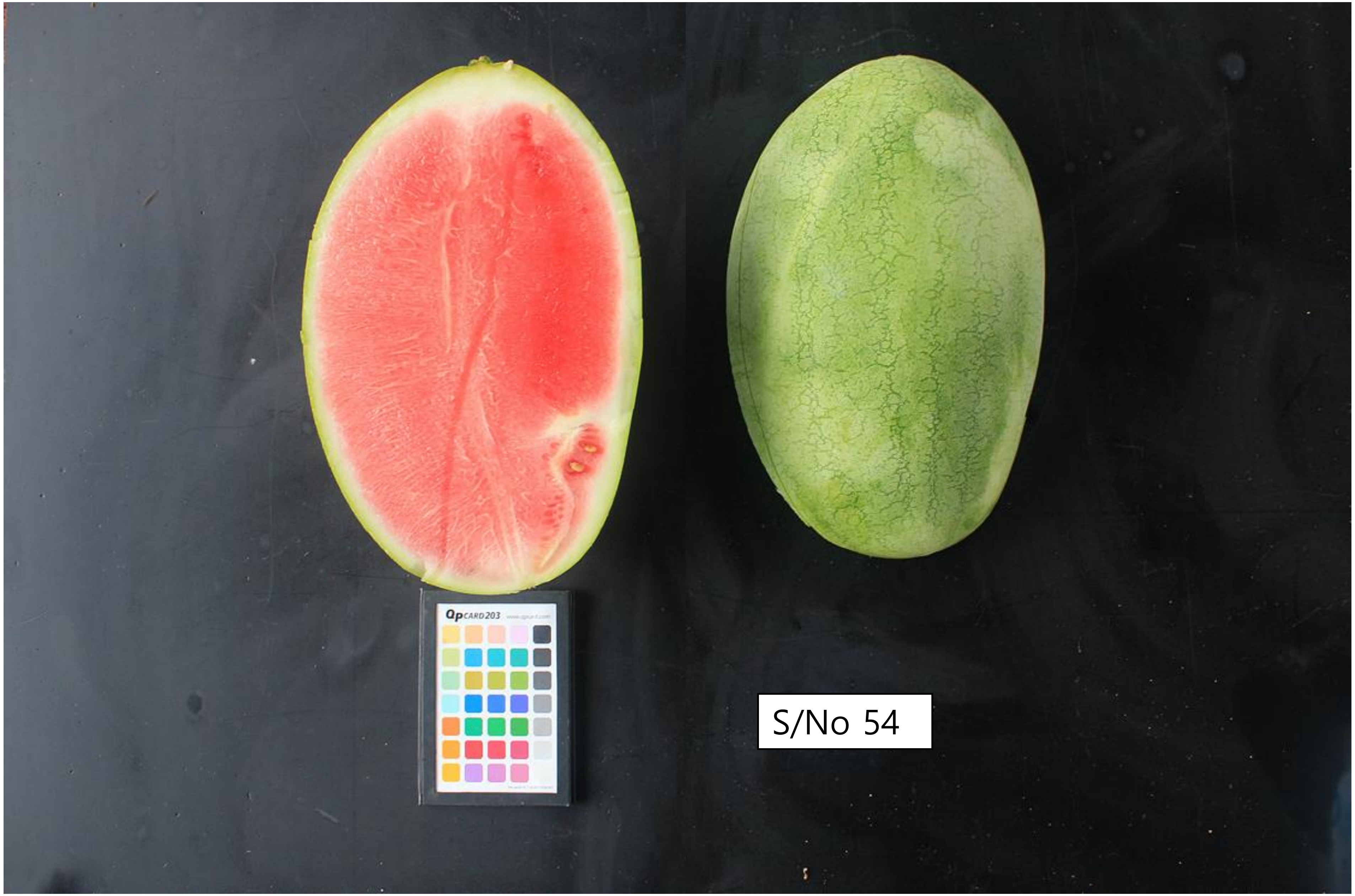
S/No 54

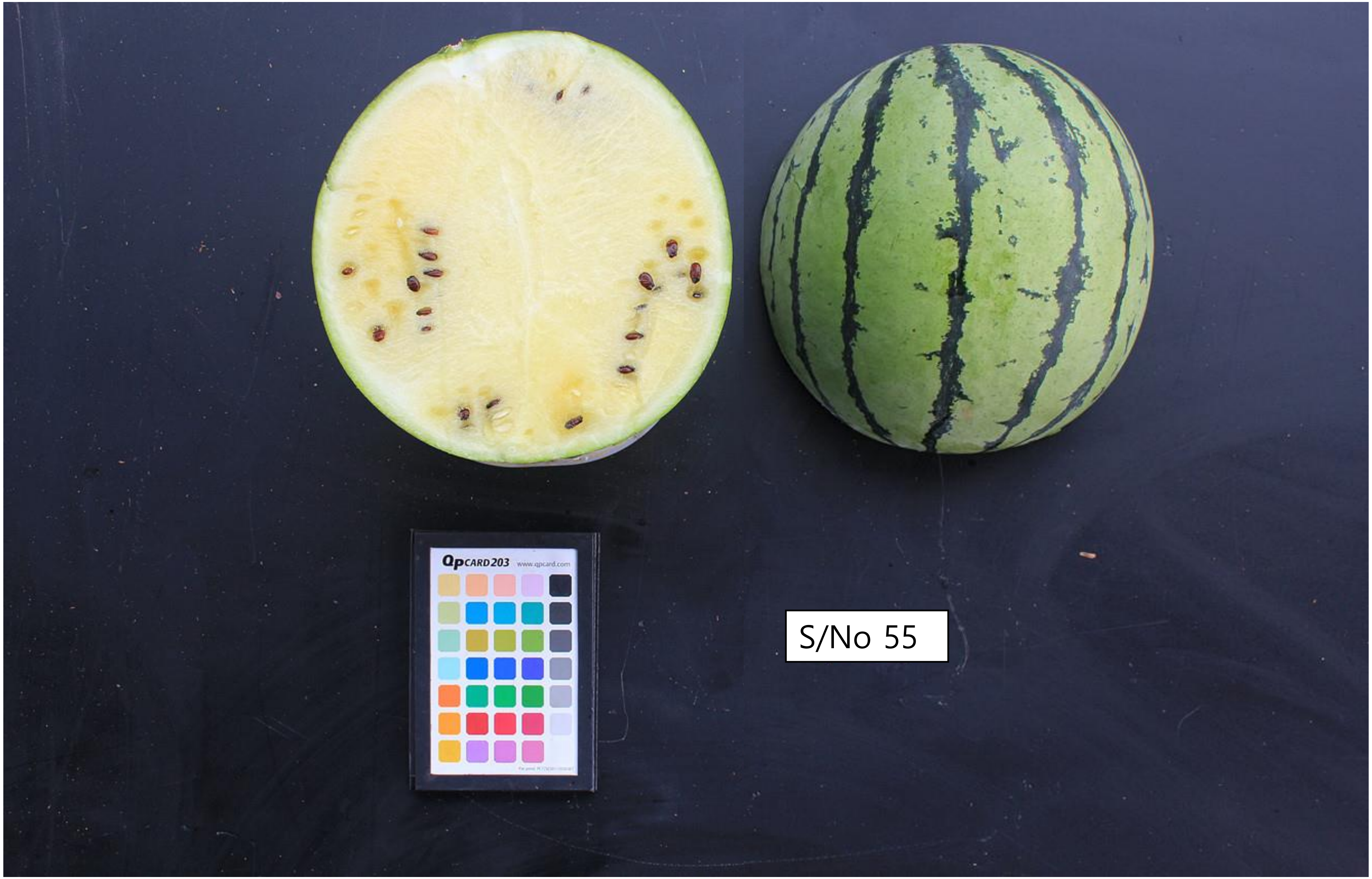
QpCARD 203
www.qpcard.com
S/No 55

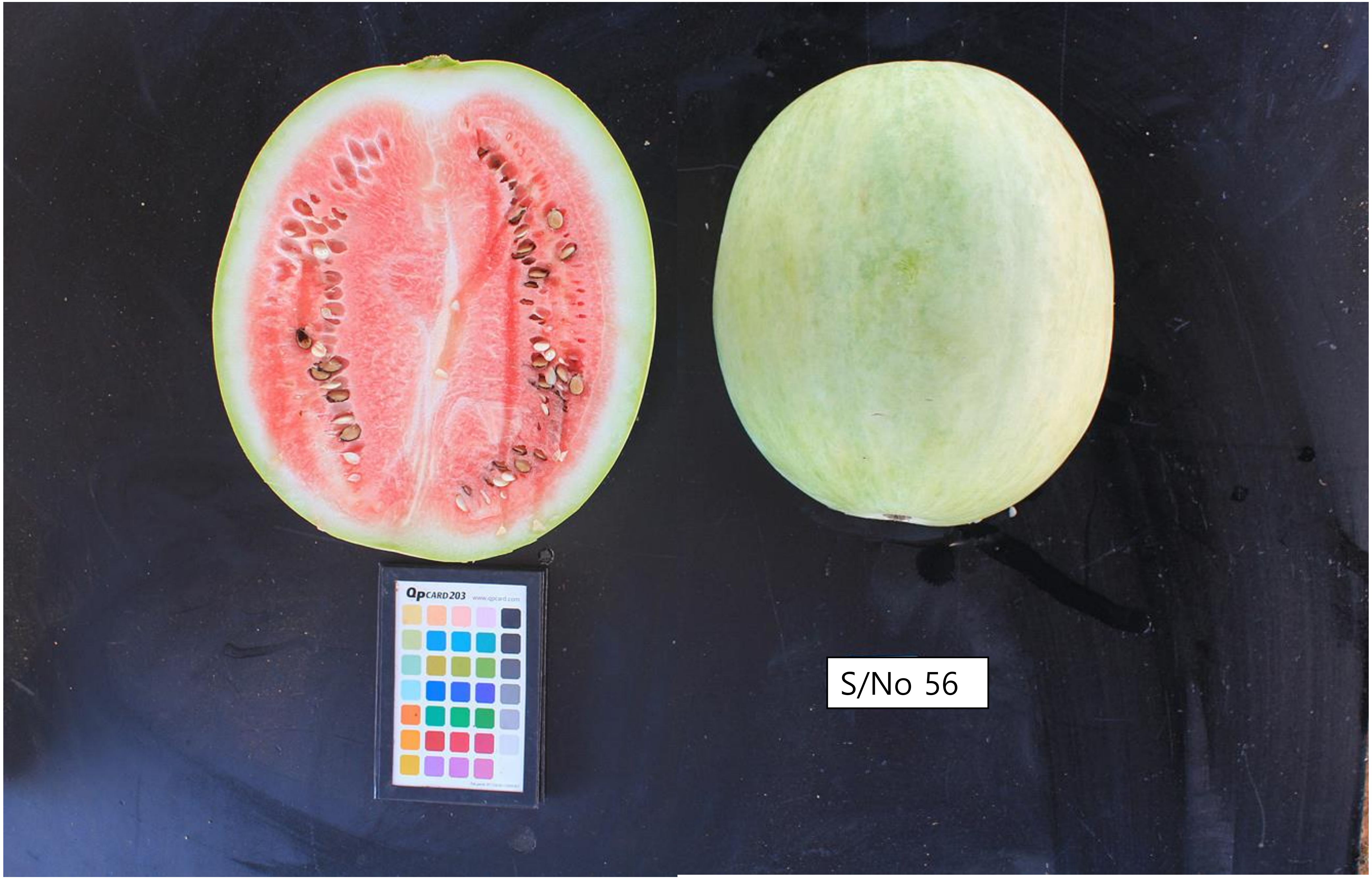
QpCARD 203
www.qpcard.com
S/No 56

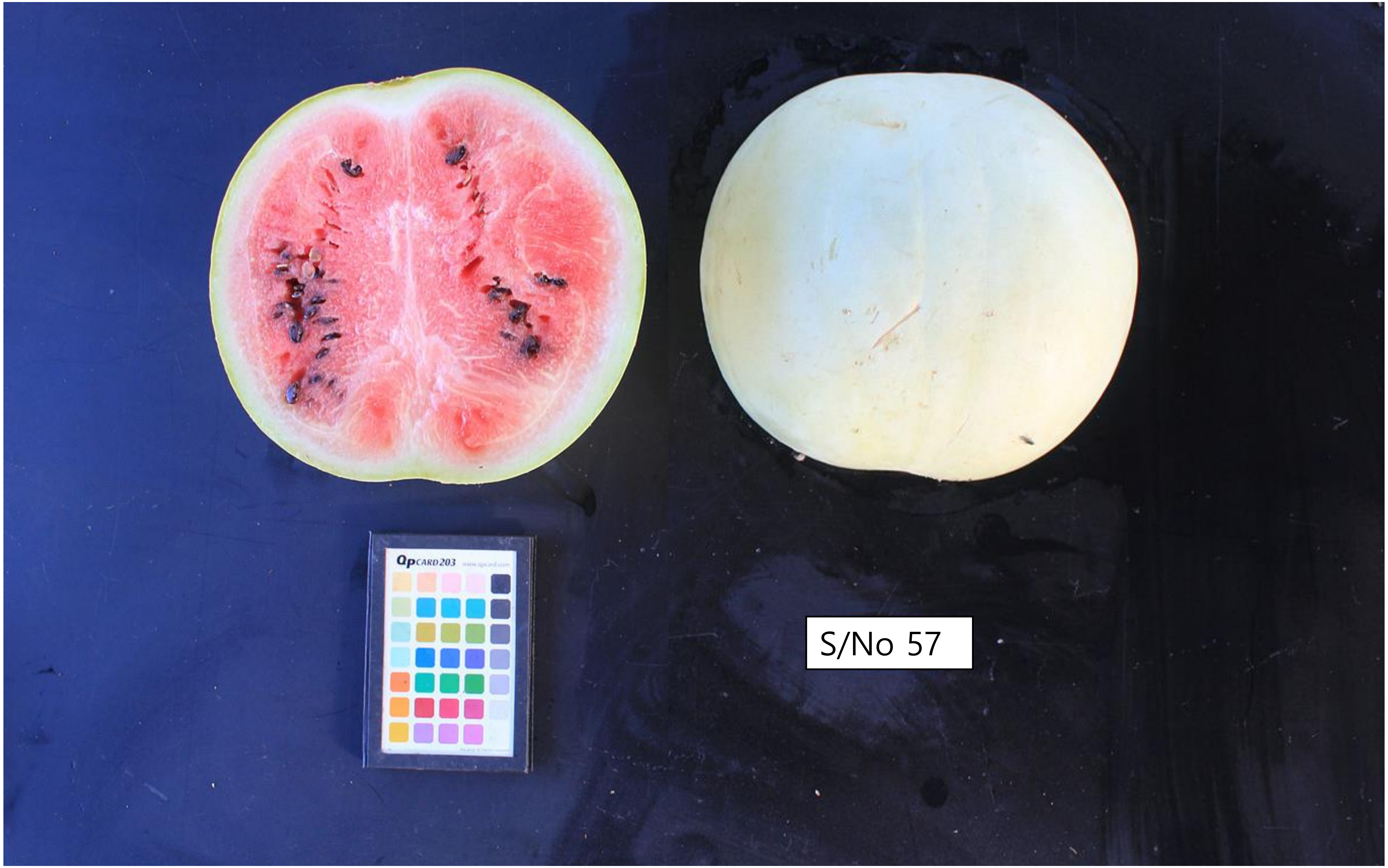
QpCARD 203
S/No 57

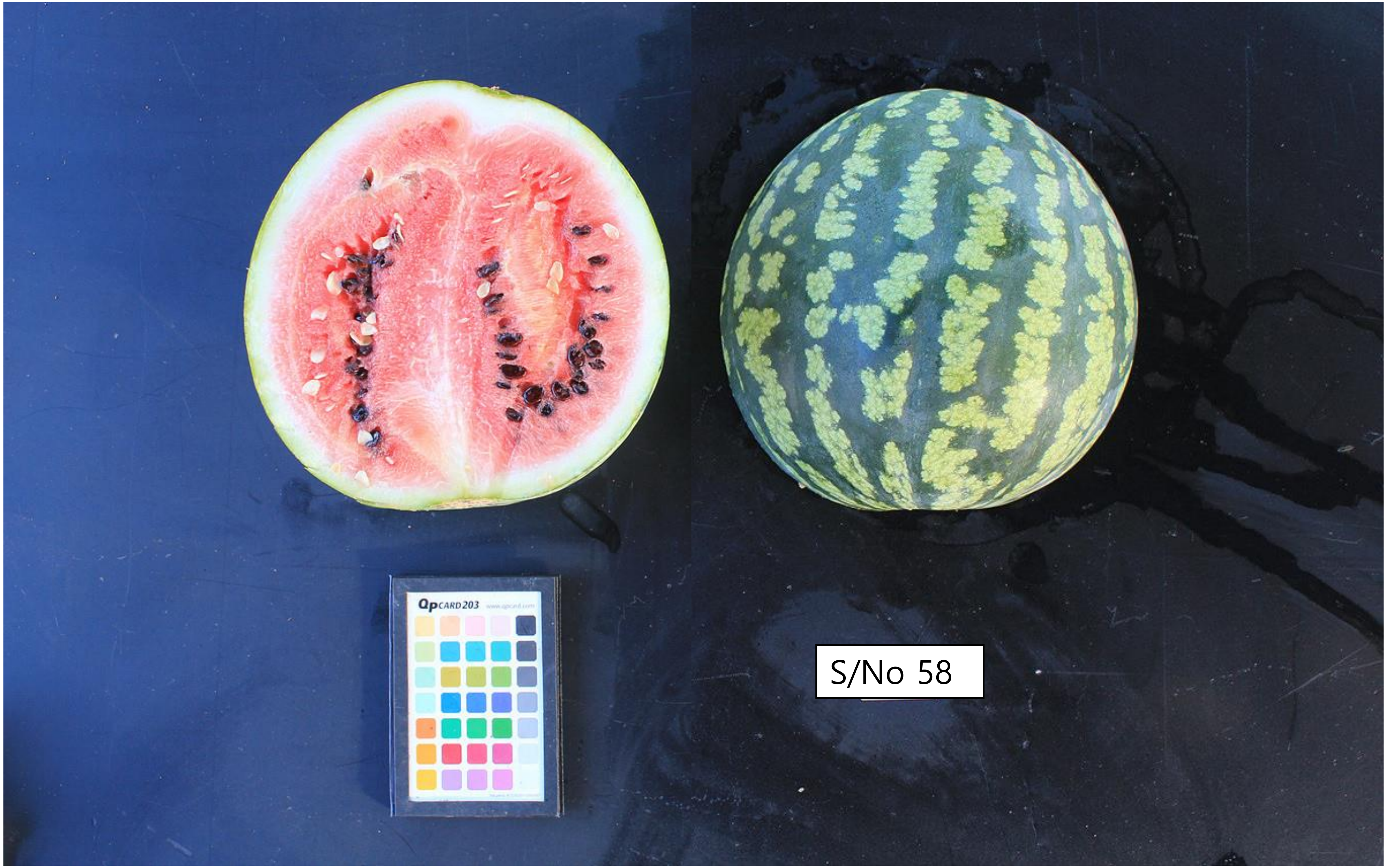
QpCARD203
S/No 58

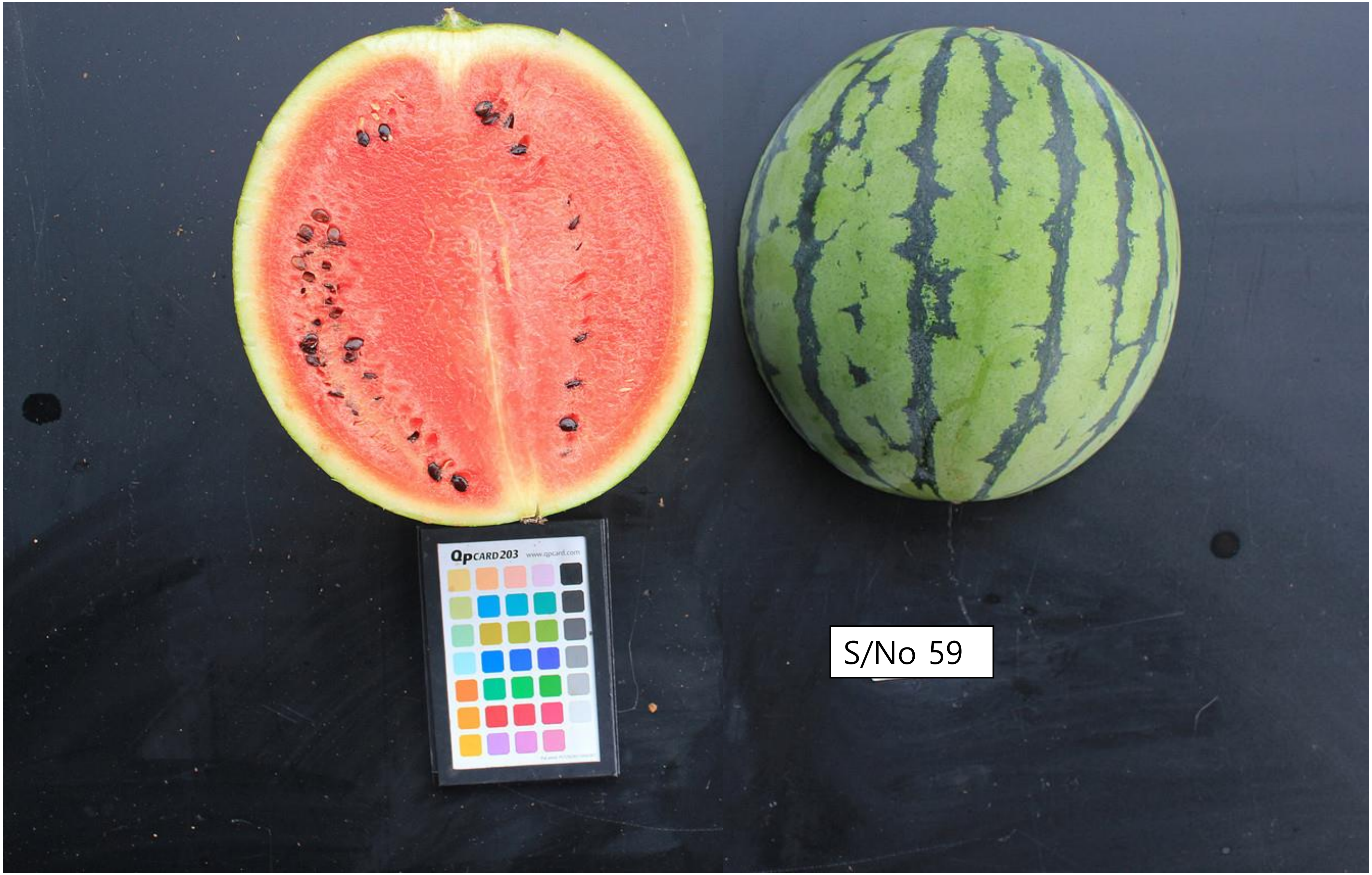
QpCARD203
www.qpcard.com
S/No 59

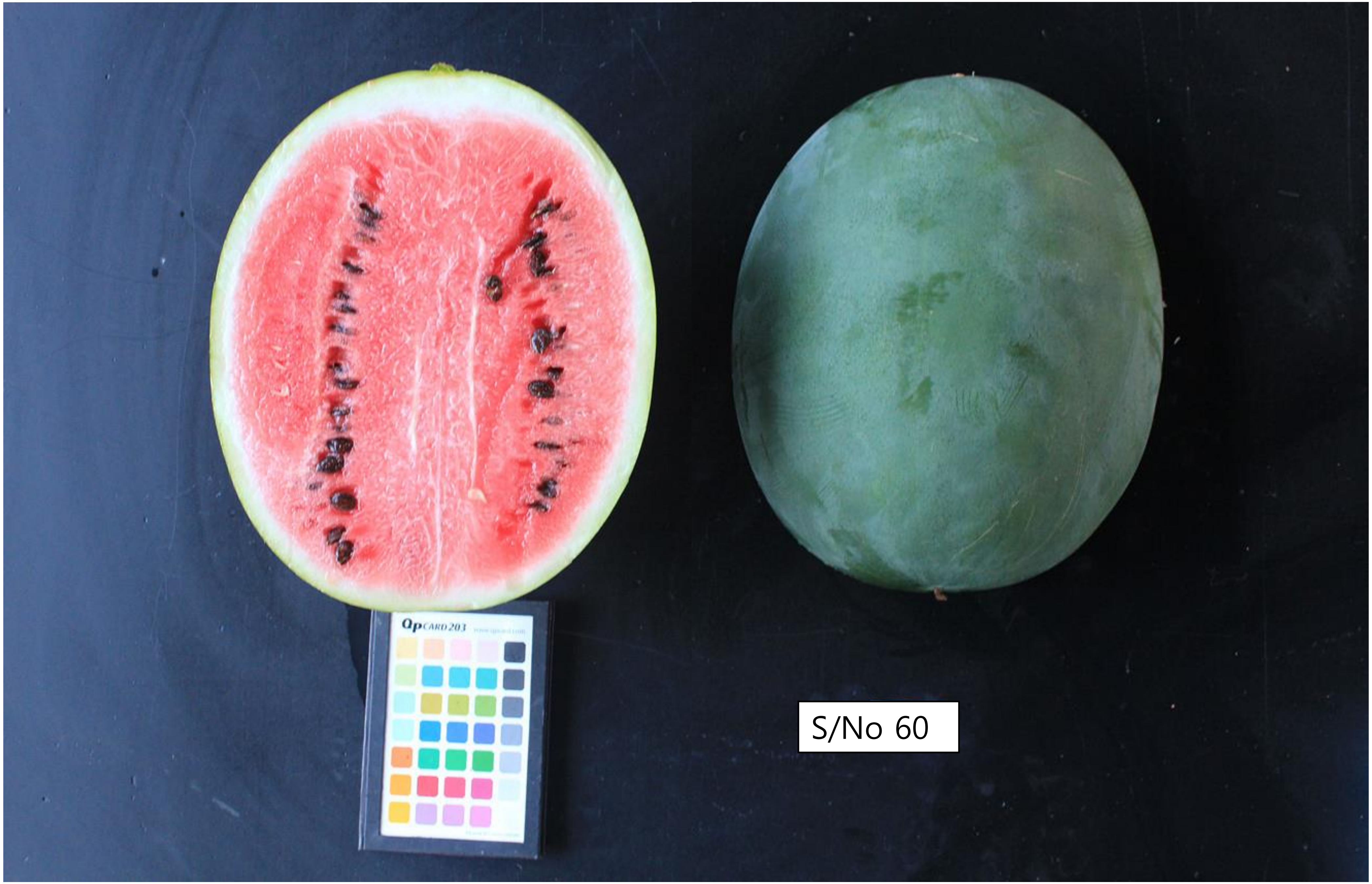
QpCARD 203
S/No 60

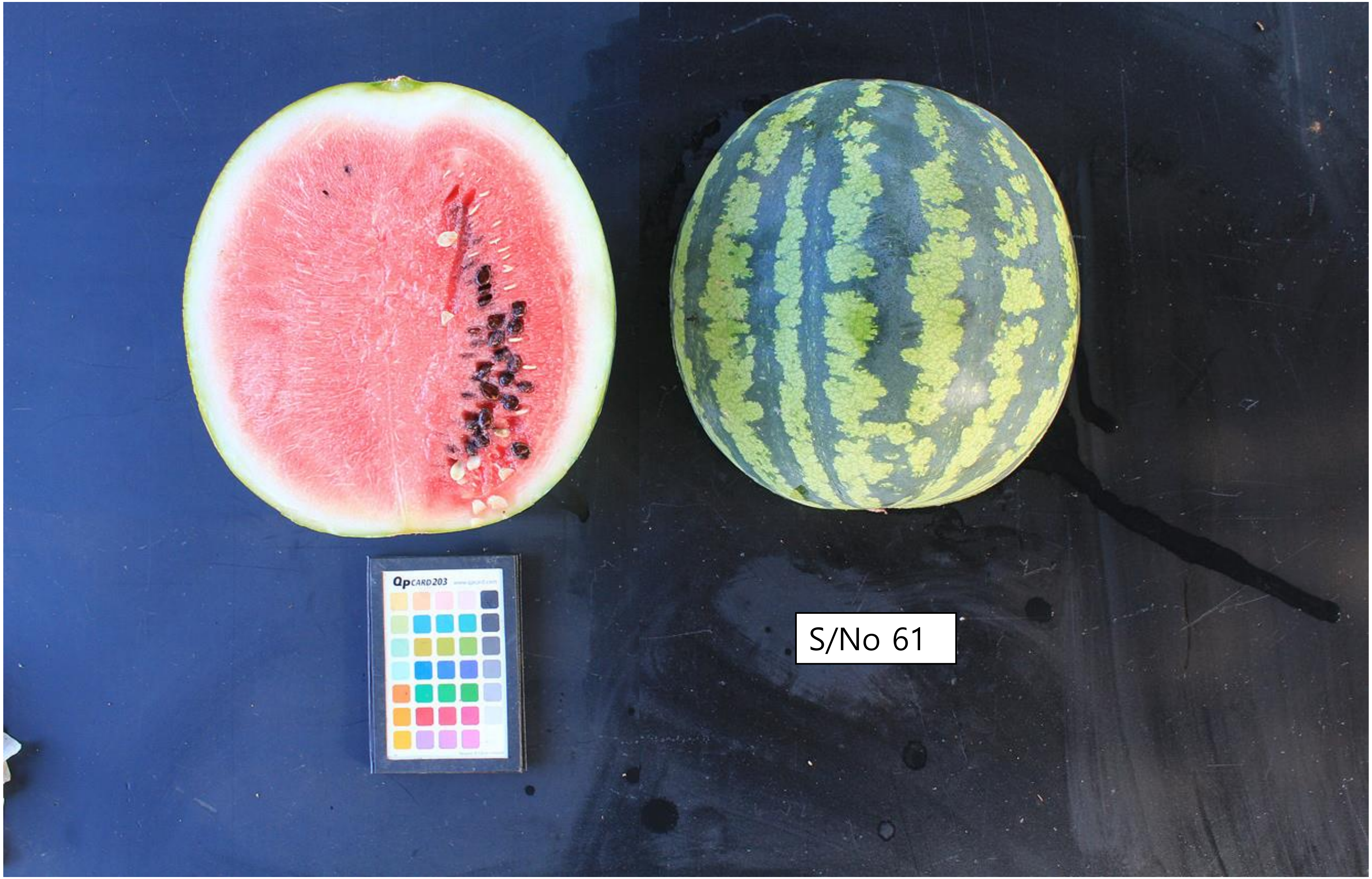
QpCARD203
S/No 61

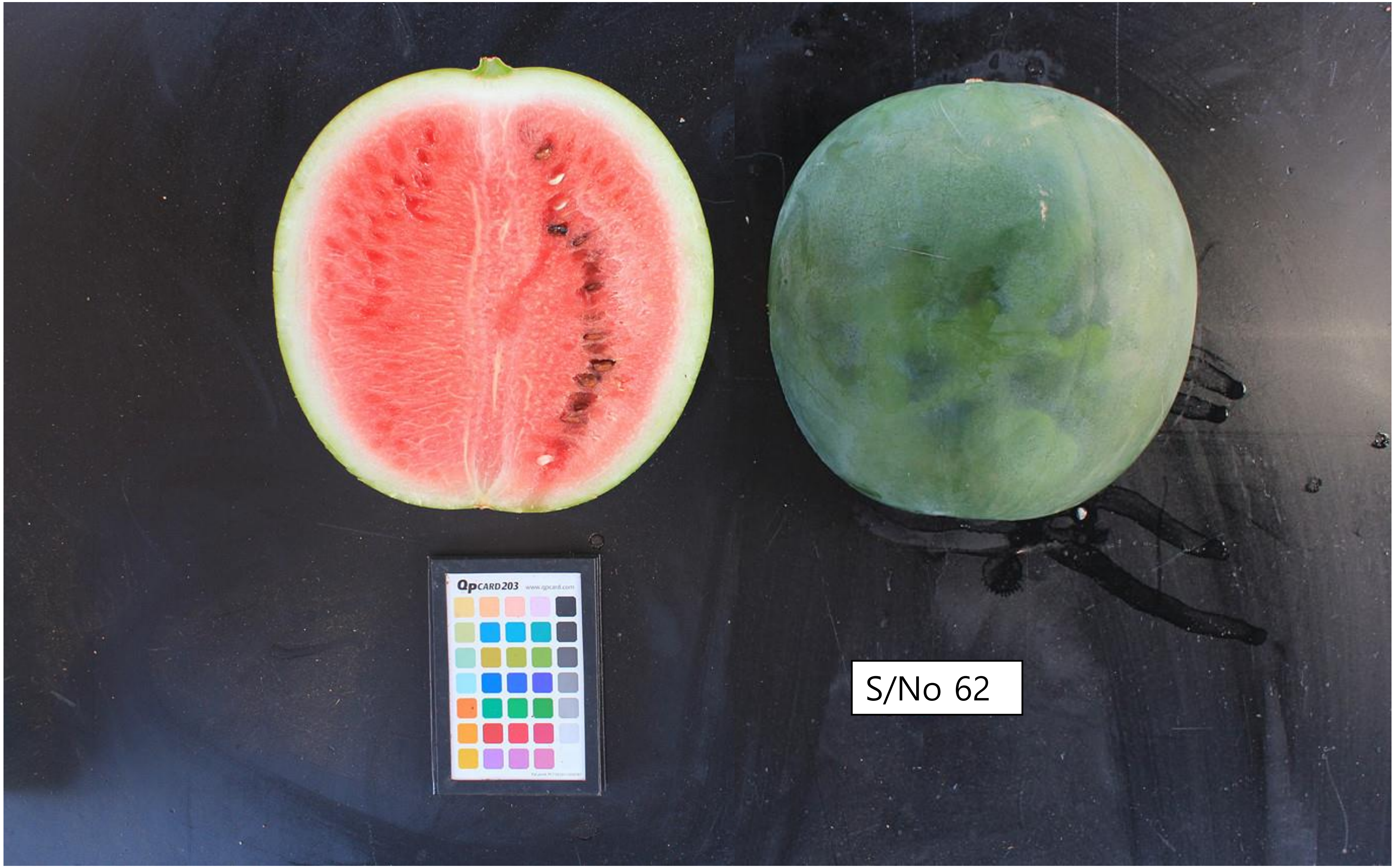
QpCARD203
S/No 62

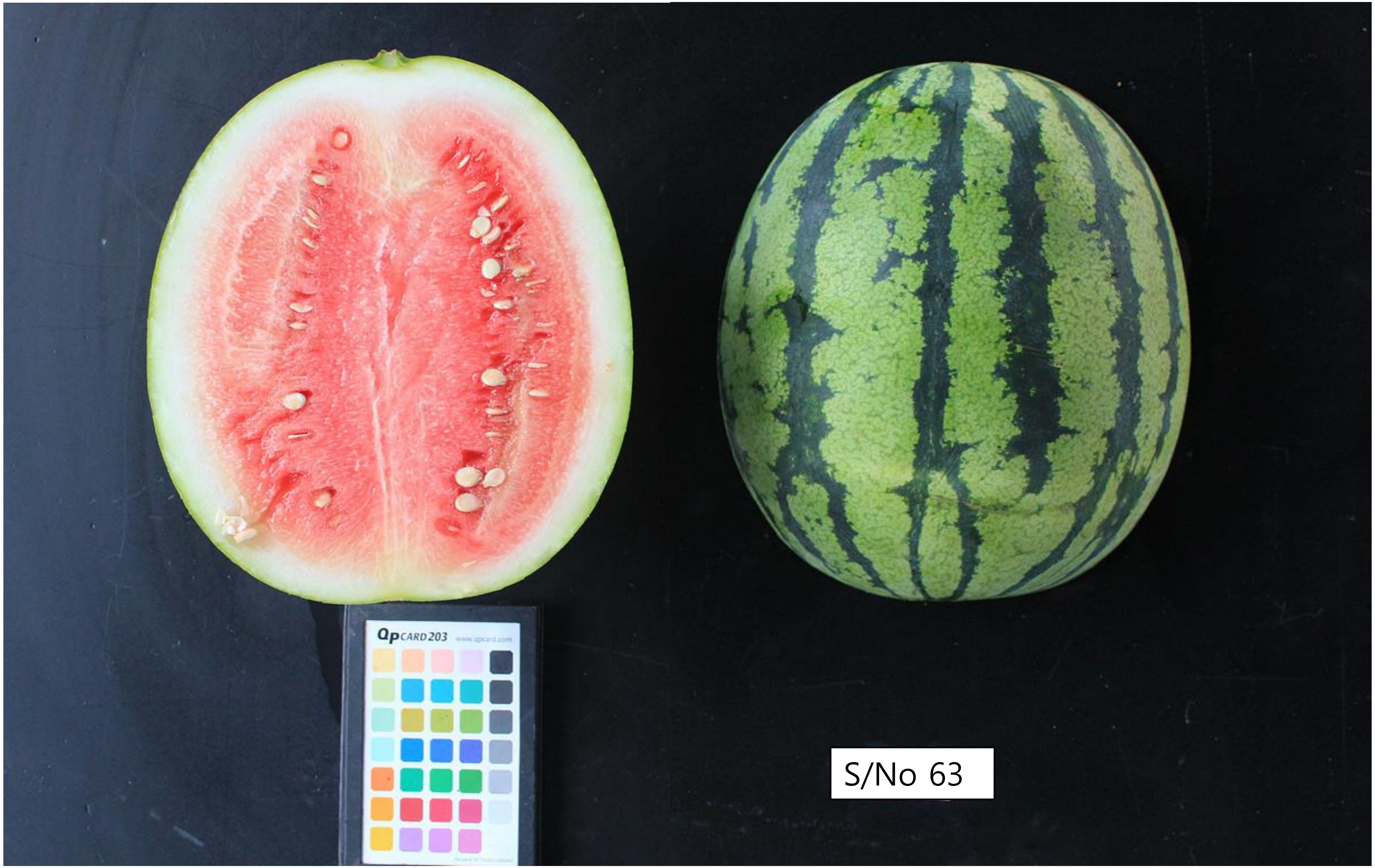

S/No 63

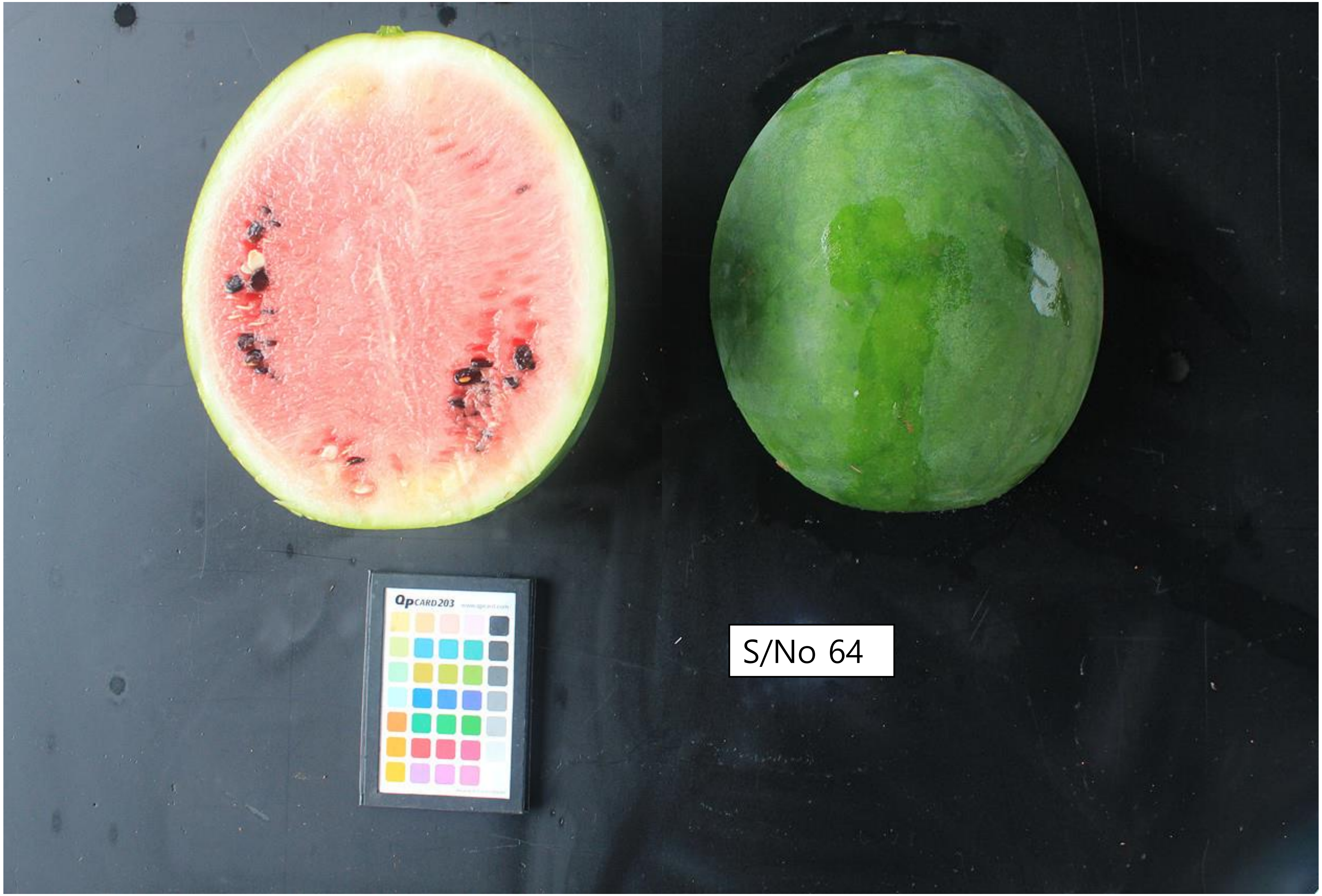
QpCARD 203
S/No 64

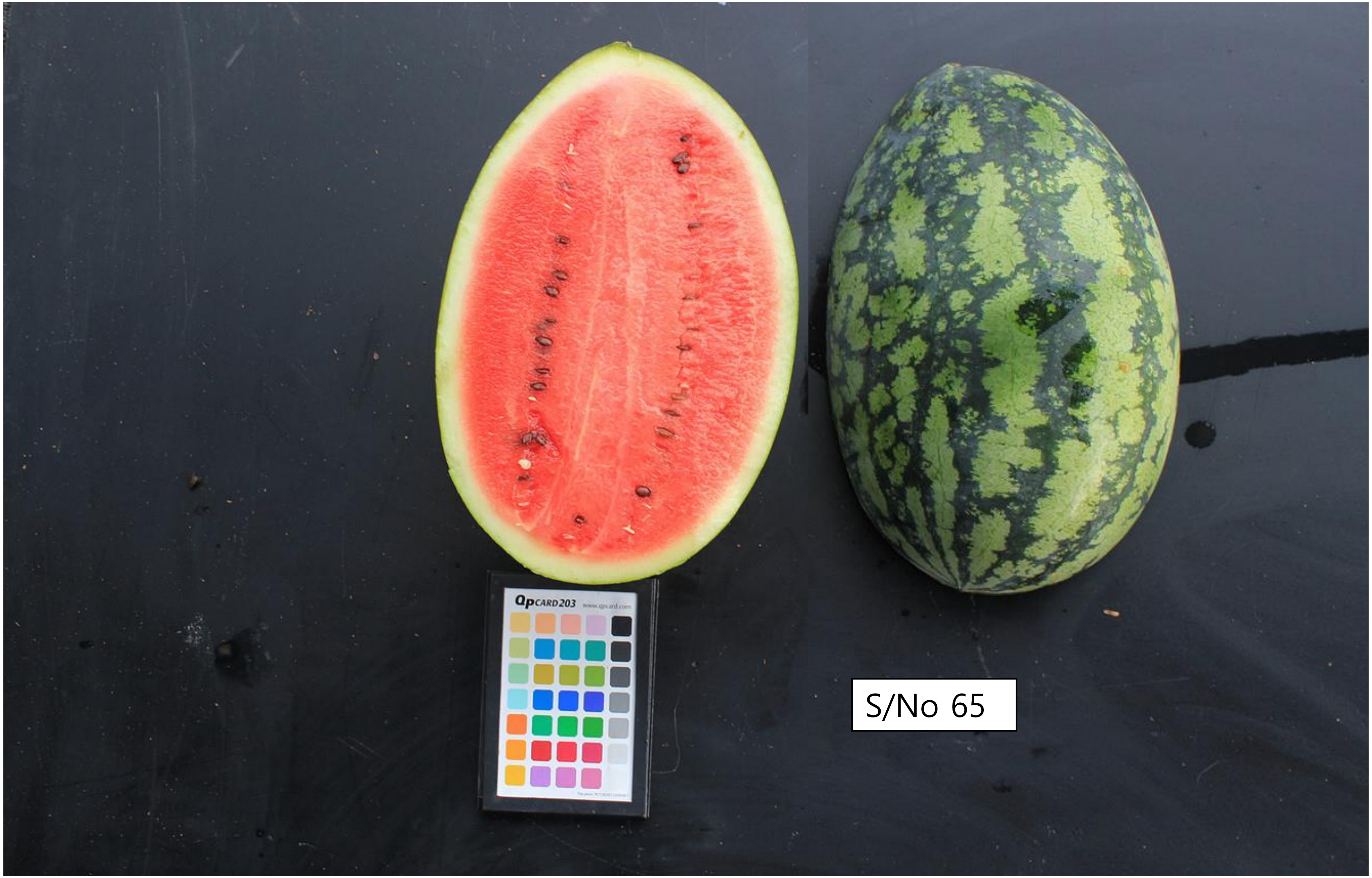
QpCARD203
www.qpcard.com
S/No 65

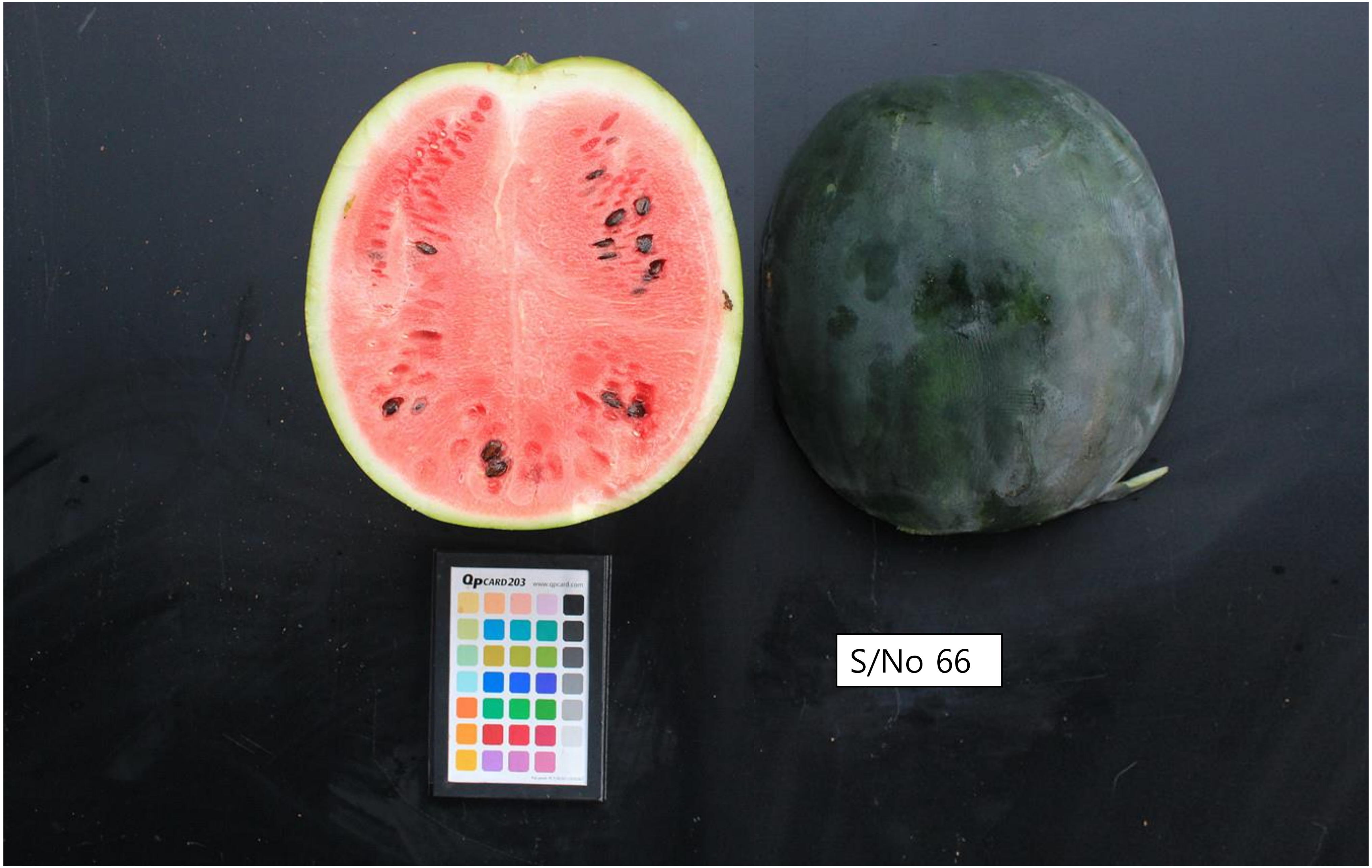
QpCARD 203
www.qpcard.com
S/No 66

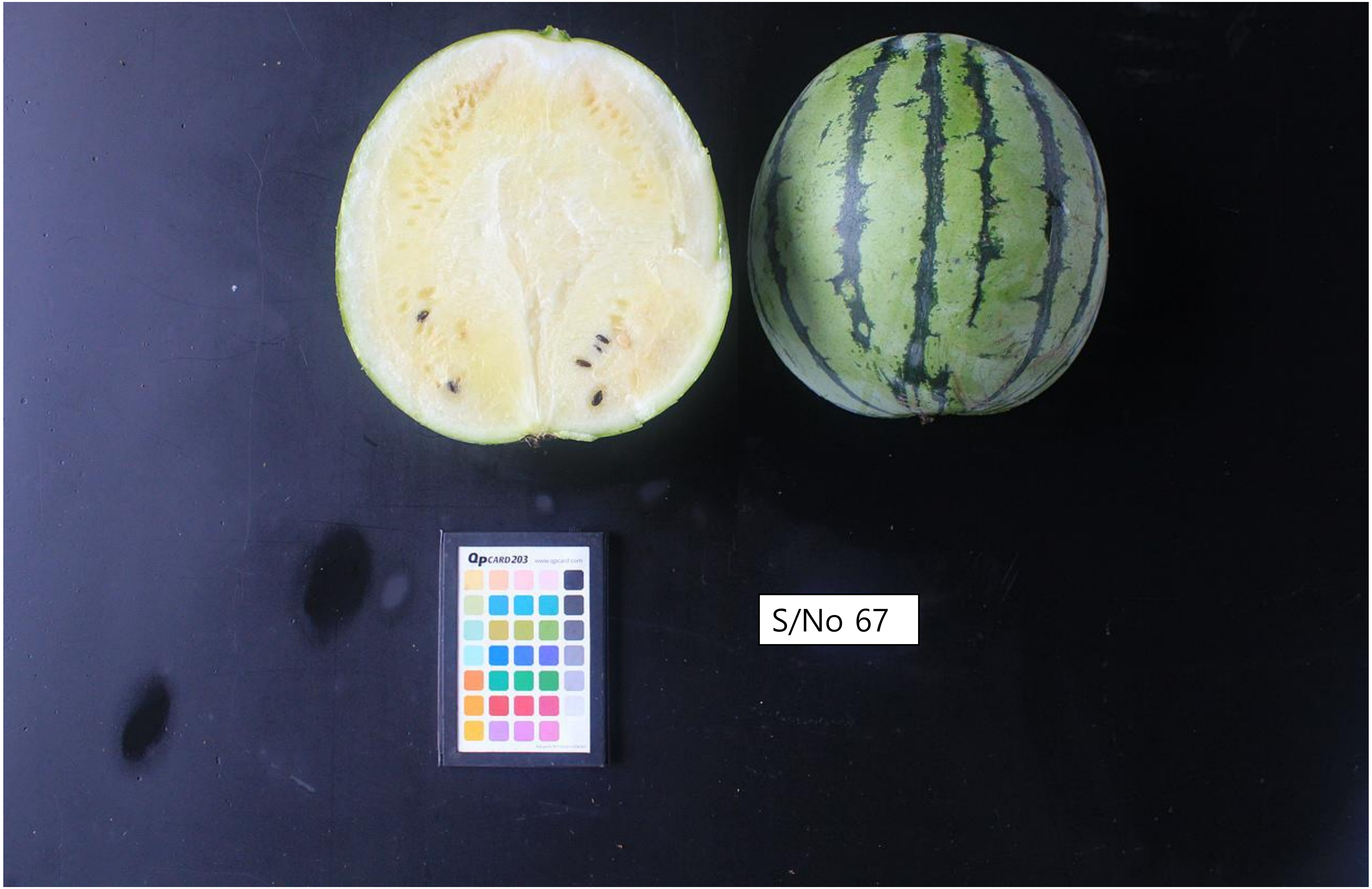
QpCARD 203
S/No 67

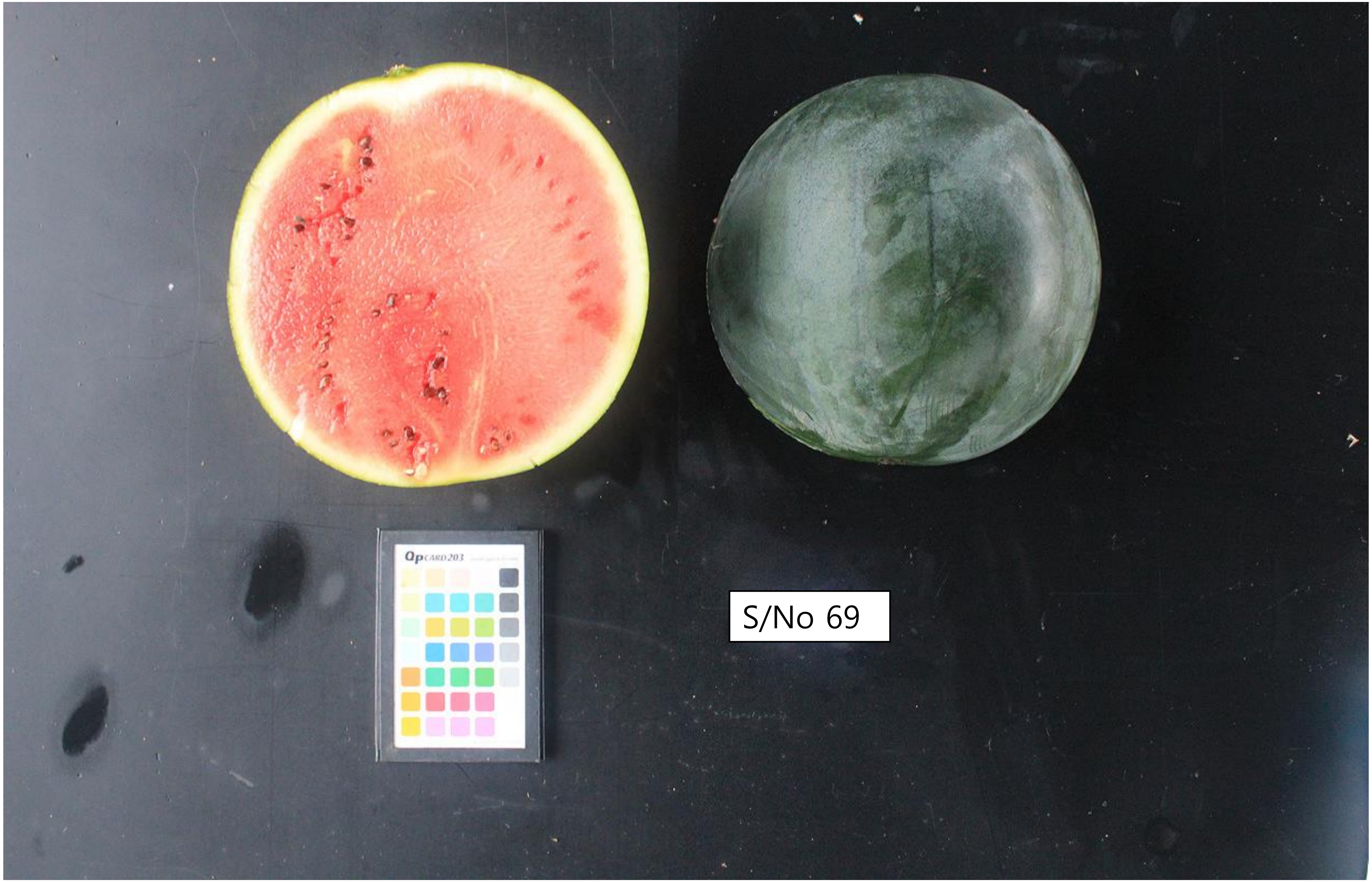
QpCARD 203
S/No 69

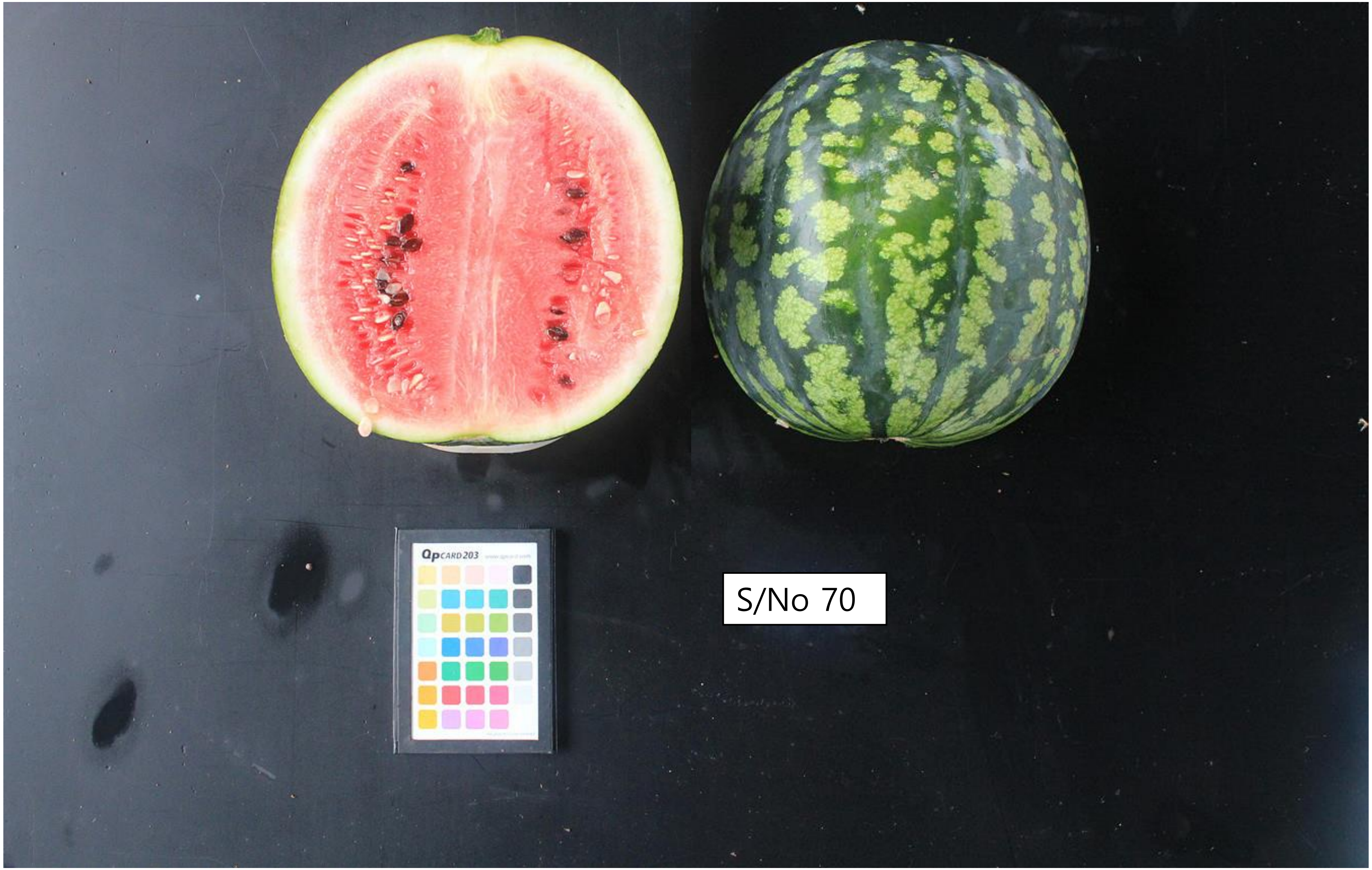

S/No 70

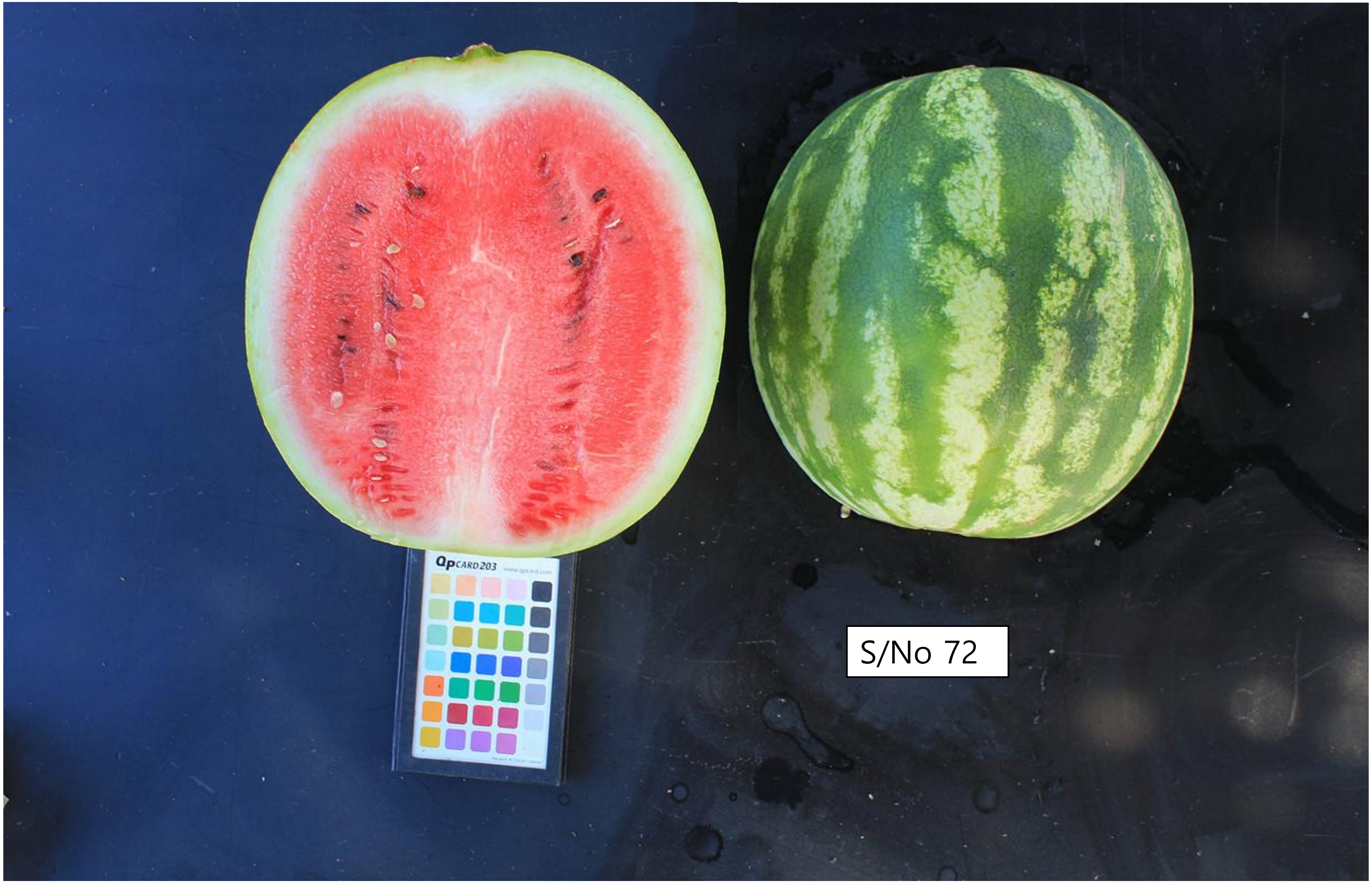
QpCARD 203
S/No 72

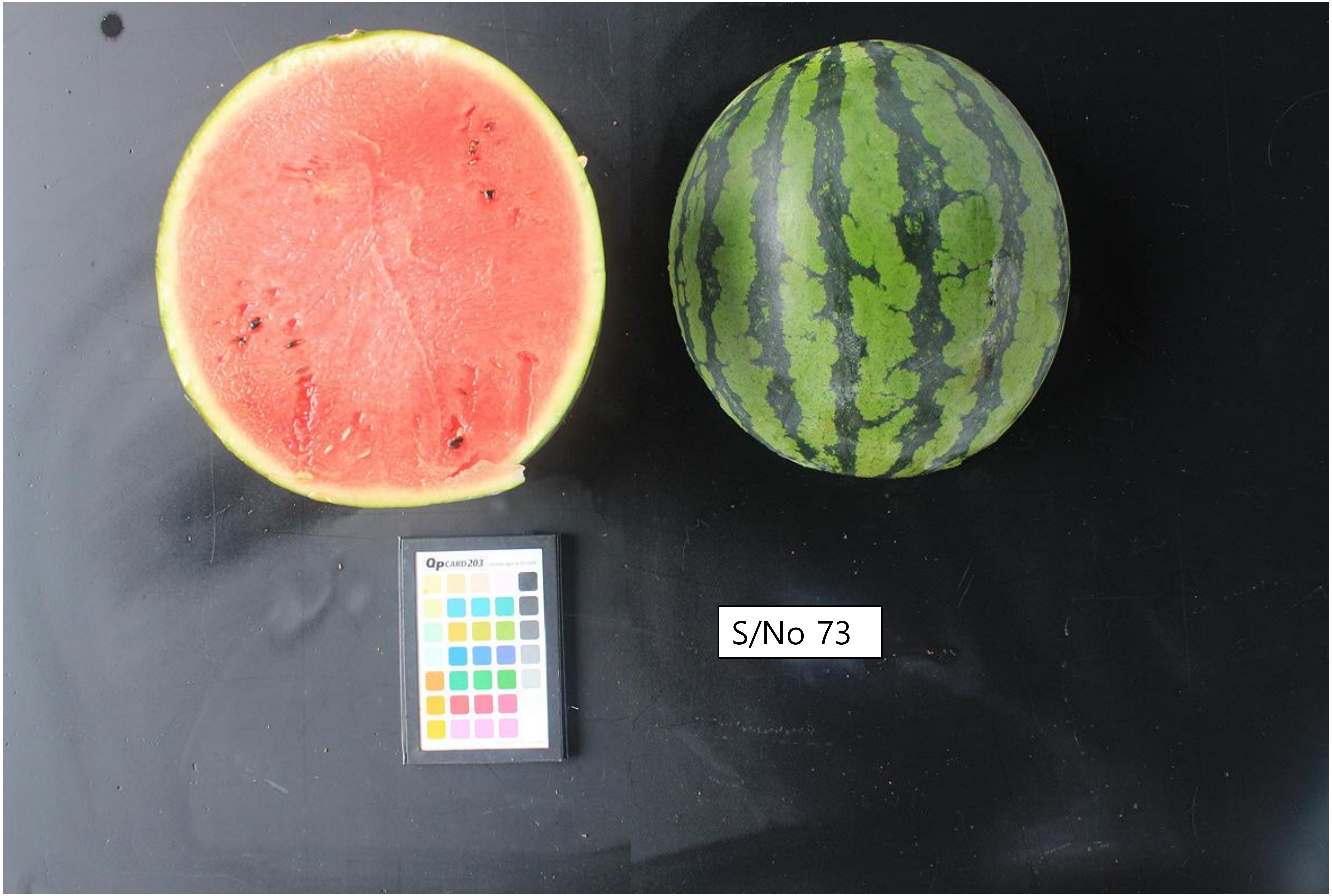
QpCARD 203
S/No 73

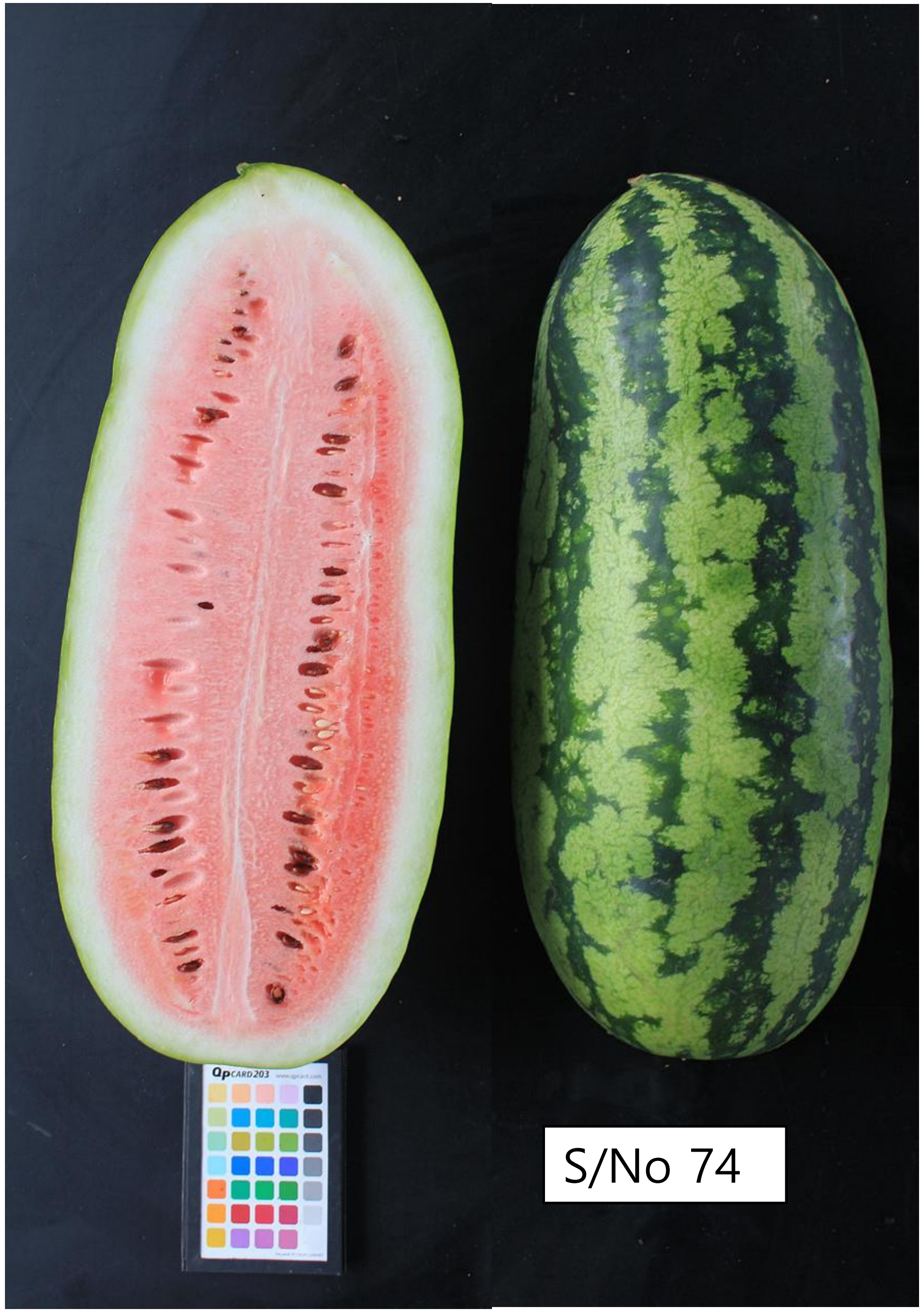
S/No 74

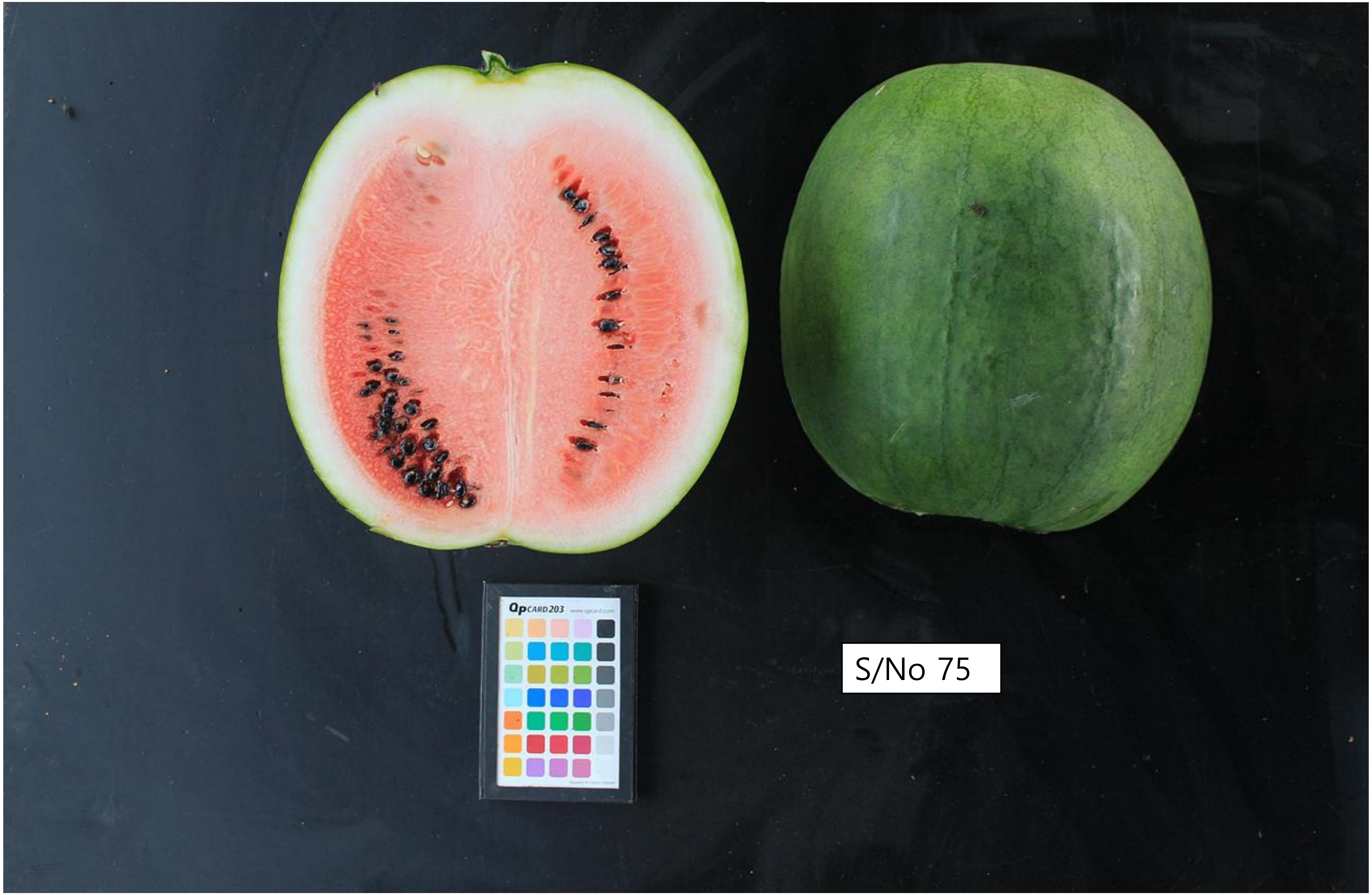
QpCARD 203
S/No 75

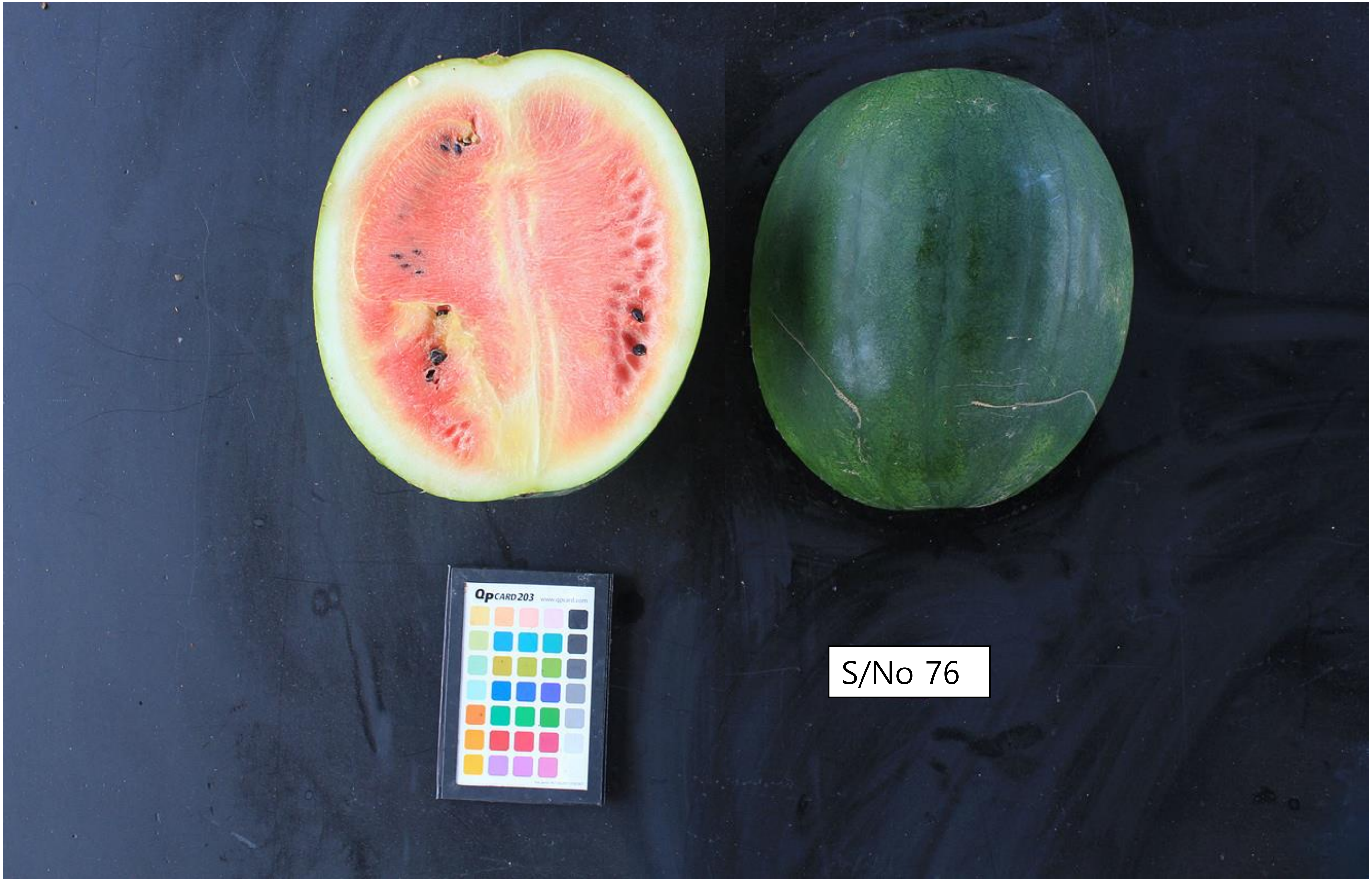
QpCARD203
S/No 76

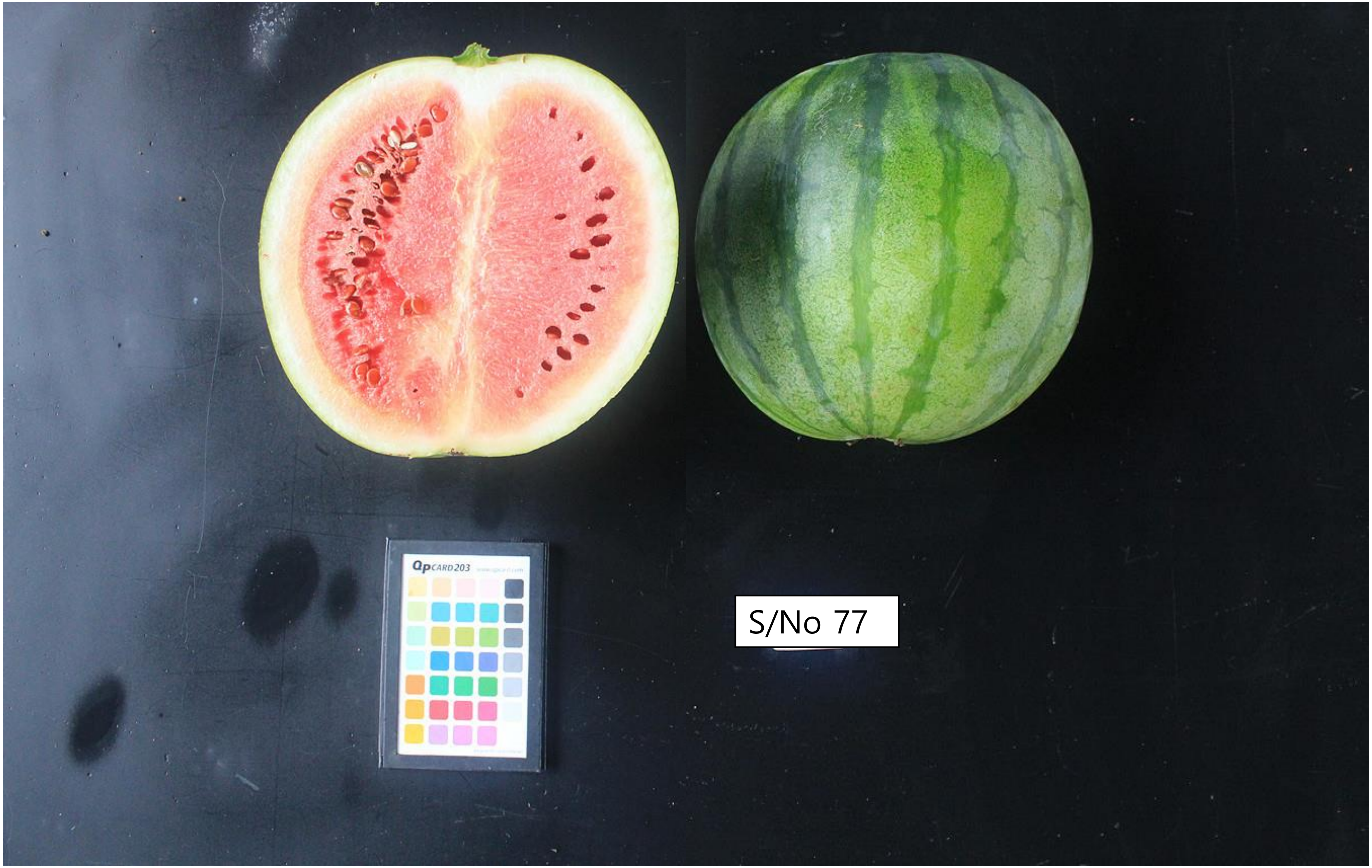
QpCARD203
S/No 77

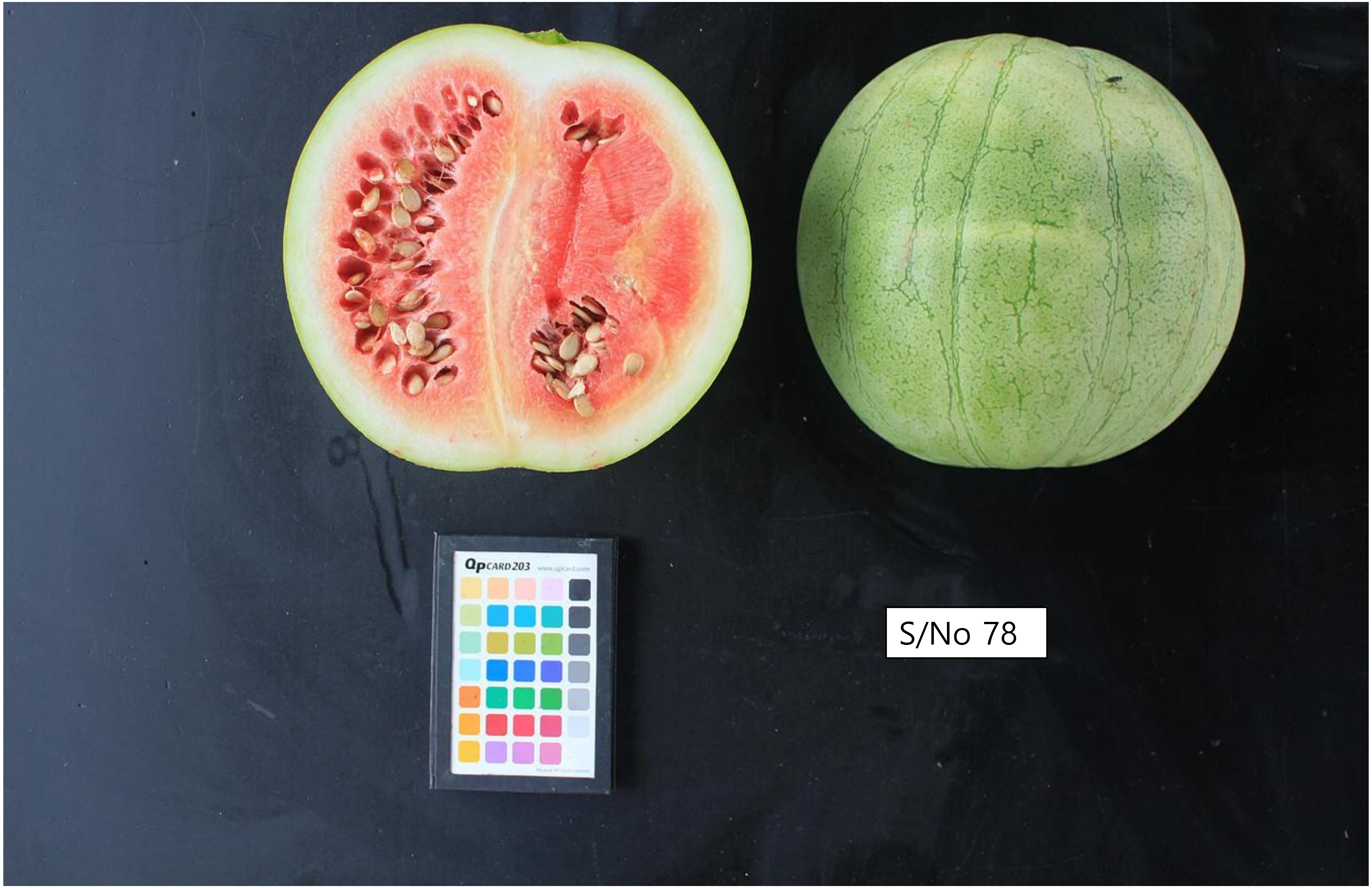
QpCARD 203
www.qpcard.com
S/No 78

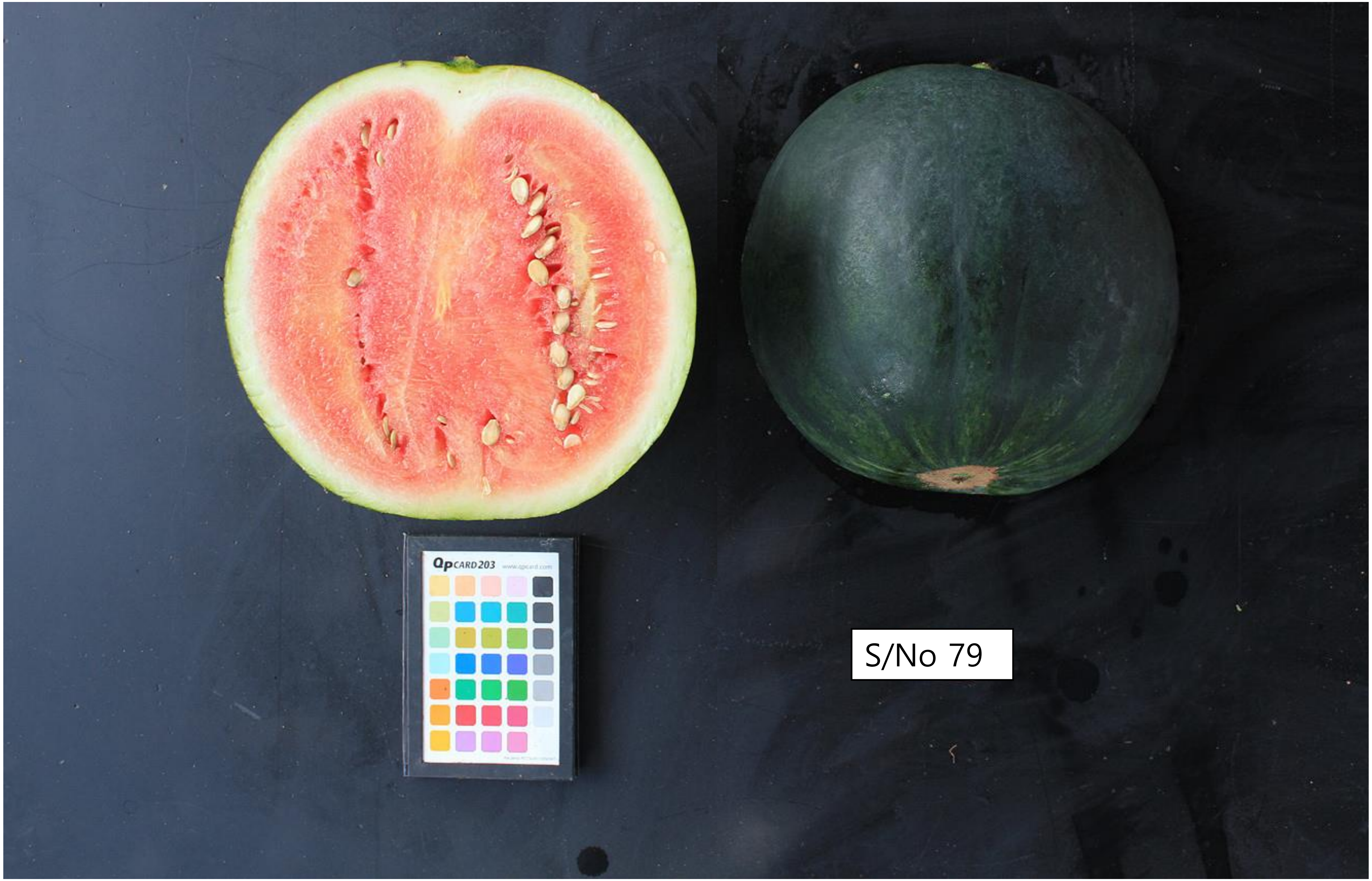
QpCARD 203
S/No 79

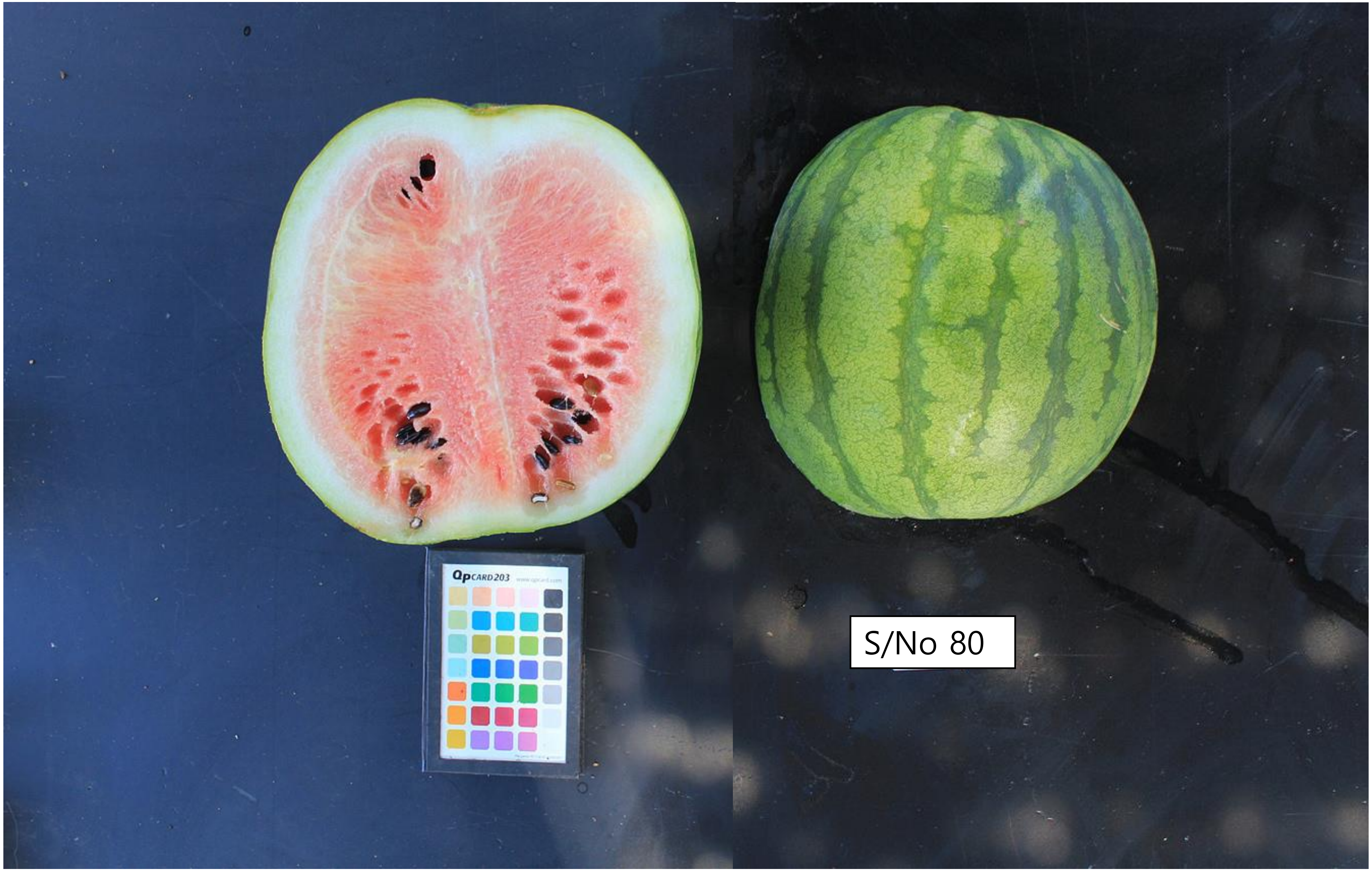
QpCARD203
S/No 80

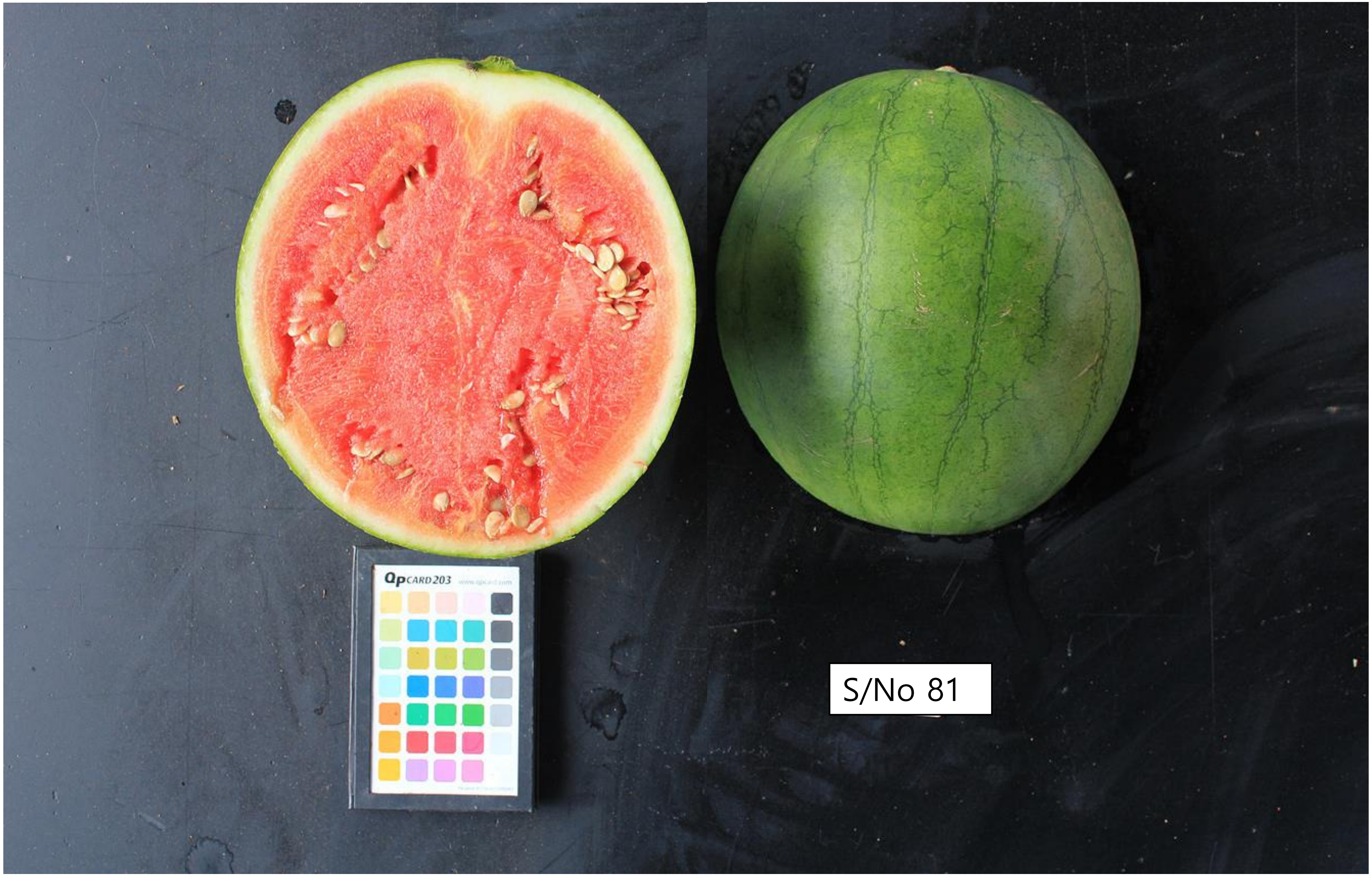
QpCARD203
S/No 81

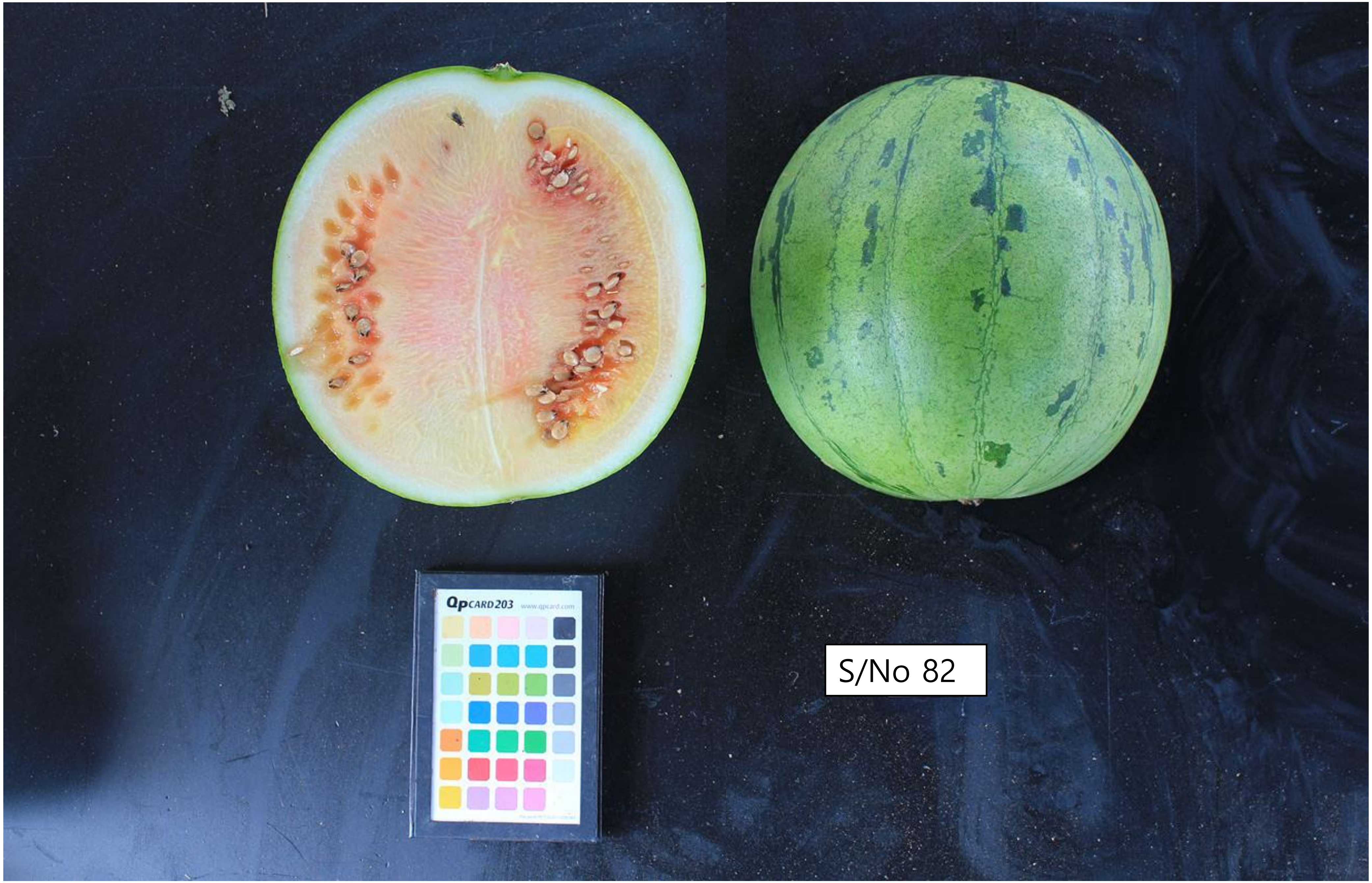
QpCARD203
www.qpcard.com
S/No 82

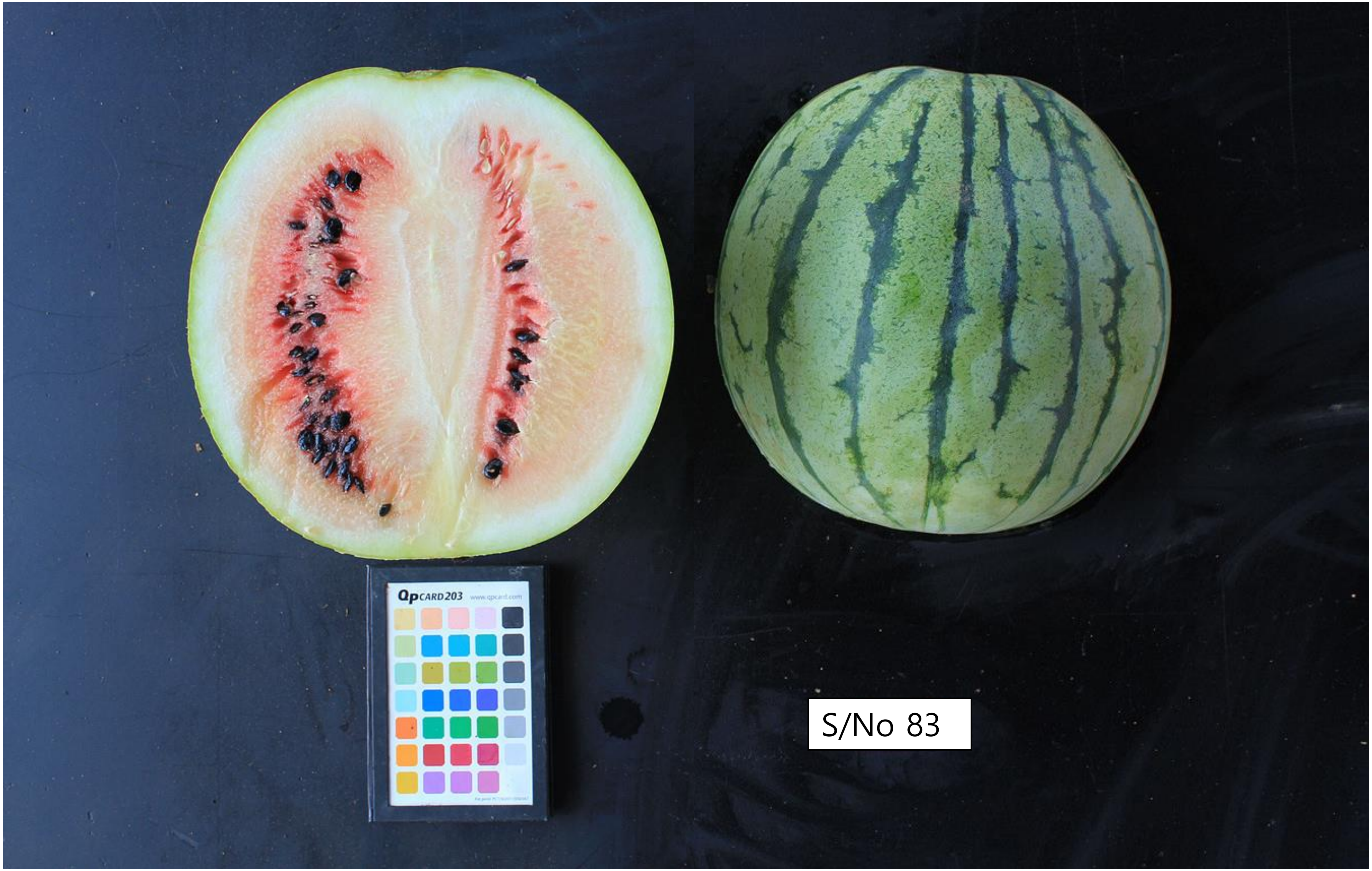

S/No 83

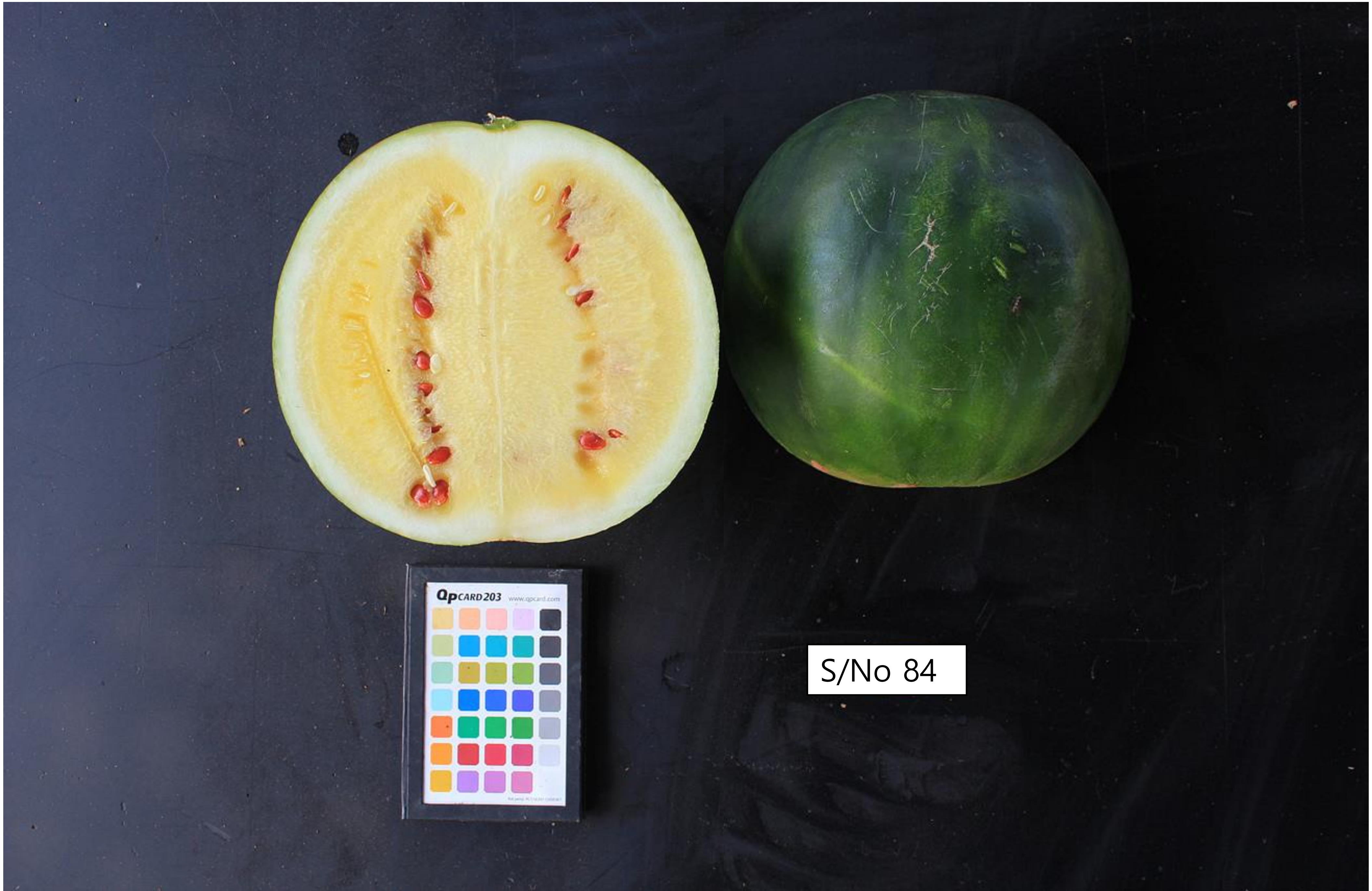
QpCARD 203
www.qpcard.com
S/No 84

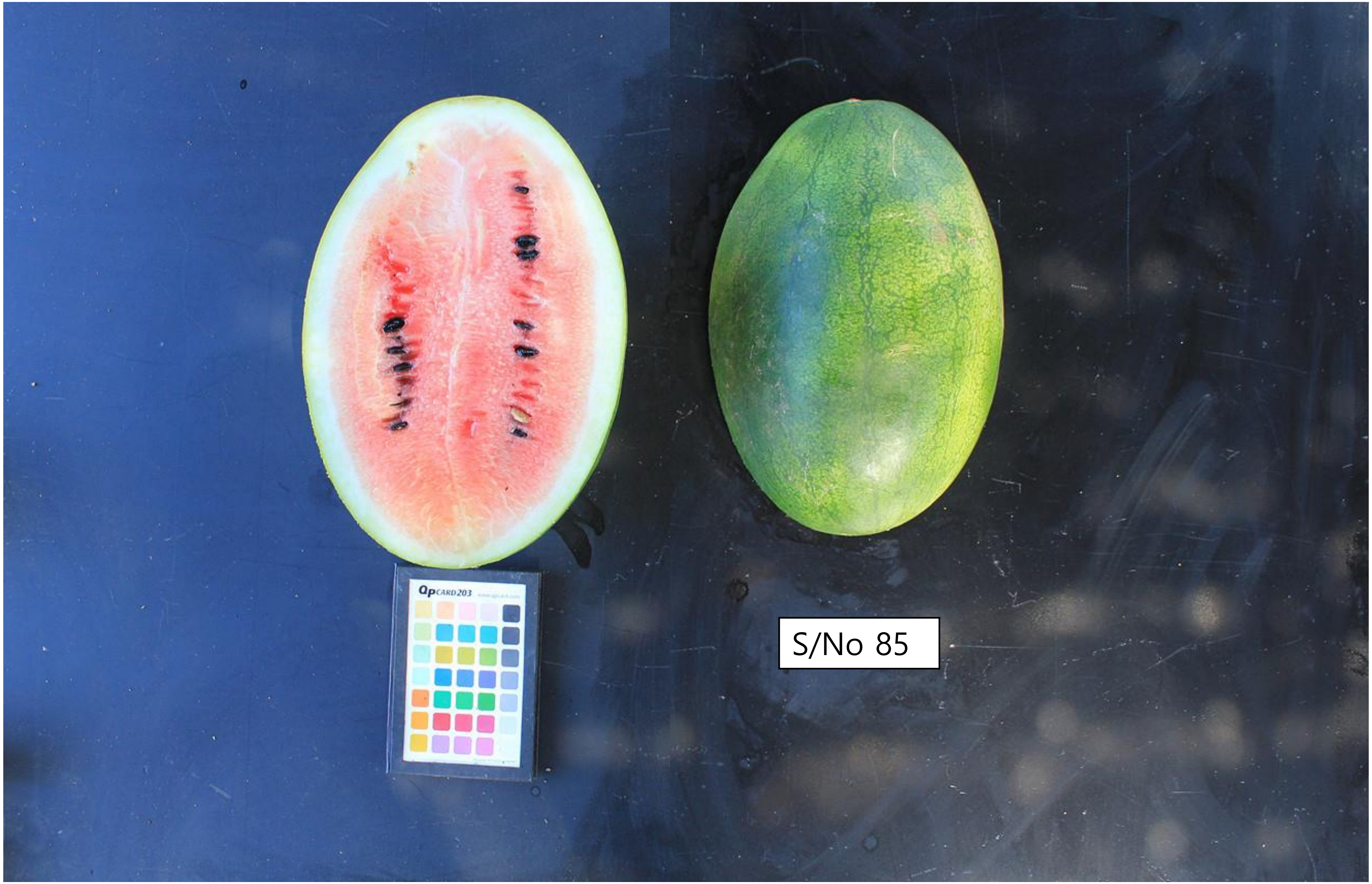
S/No 85

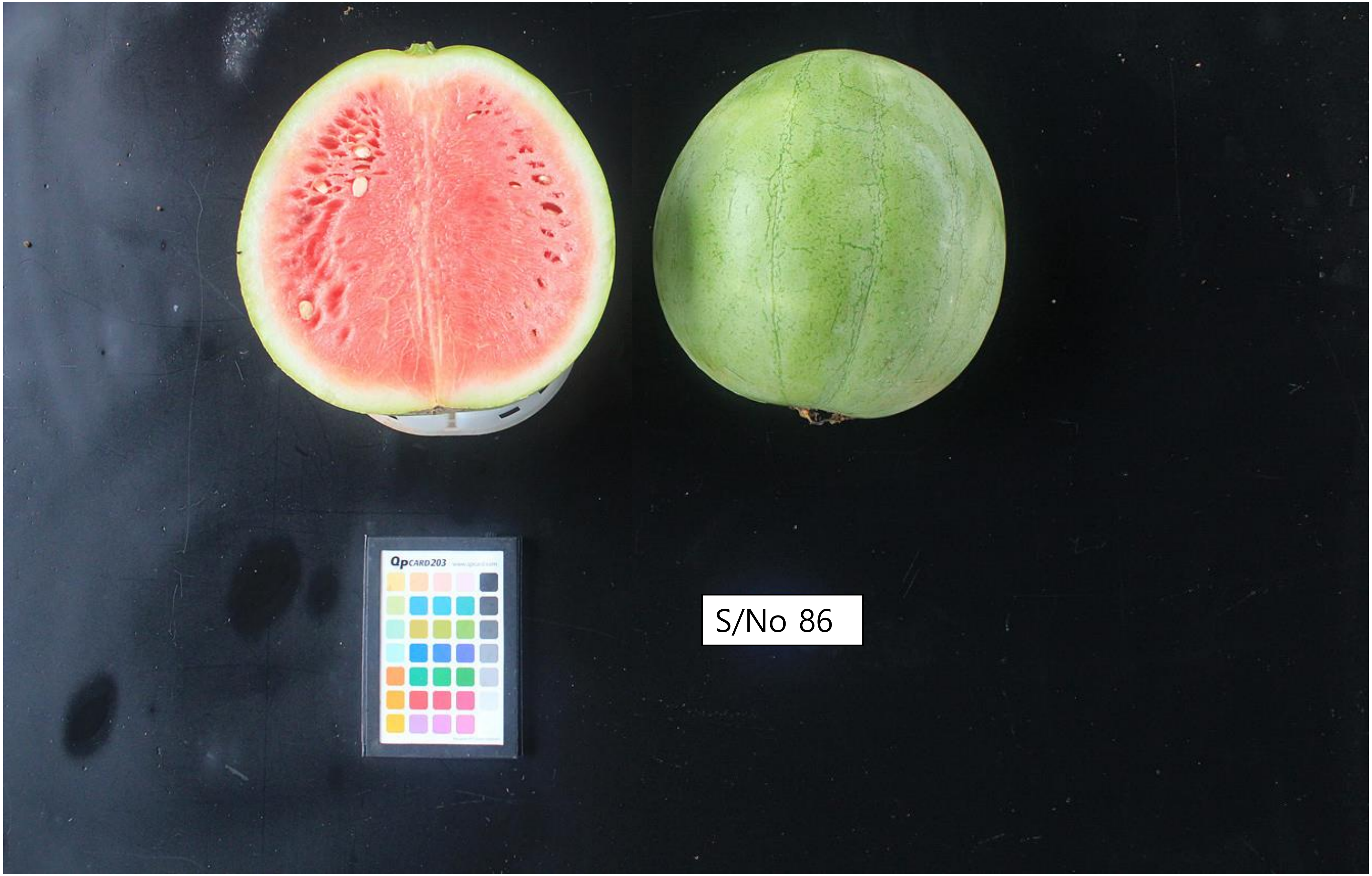
QpCARD203
S/No 86

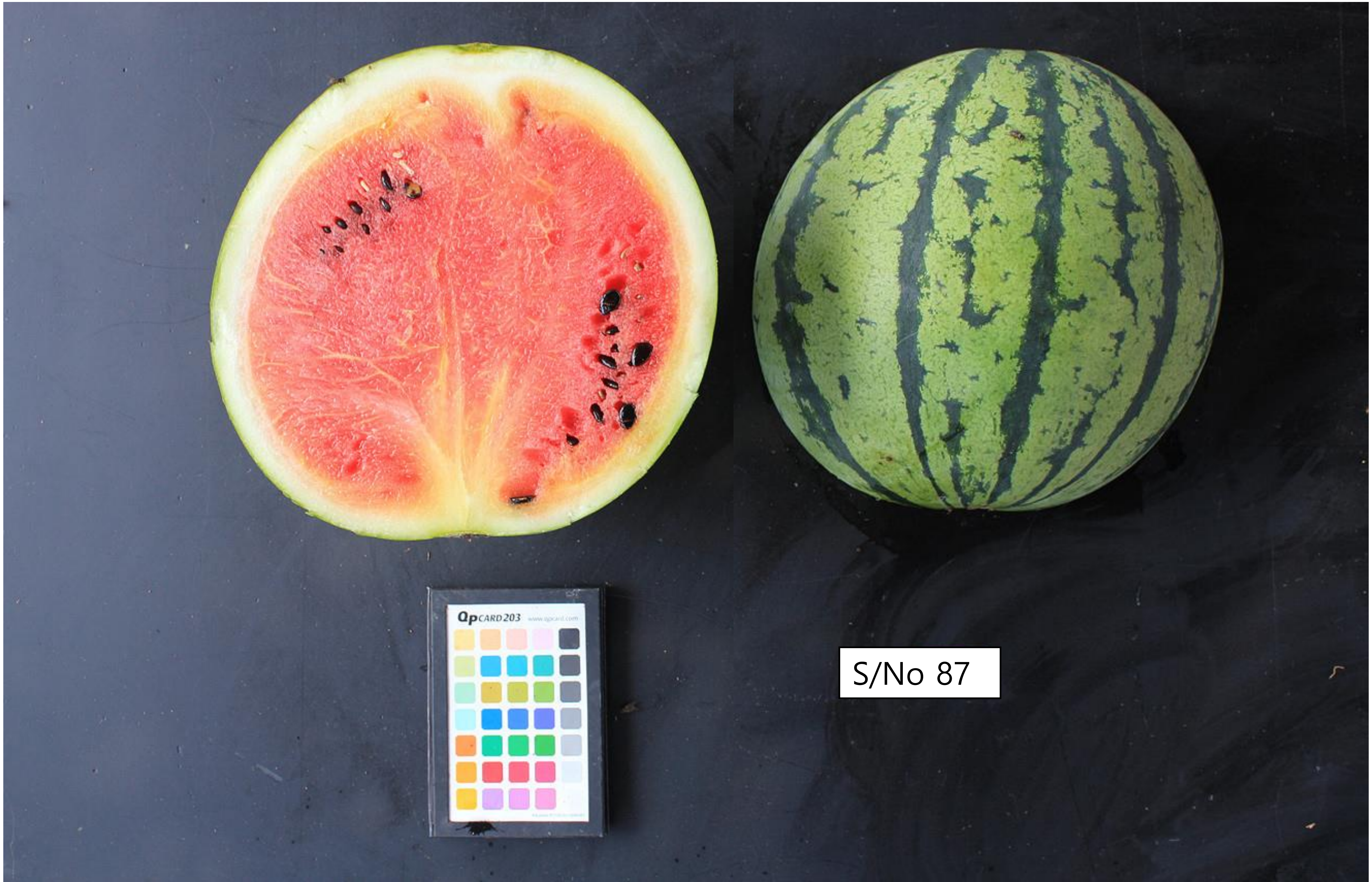
QpCARD 203
S/No 87

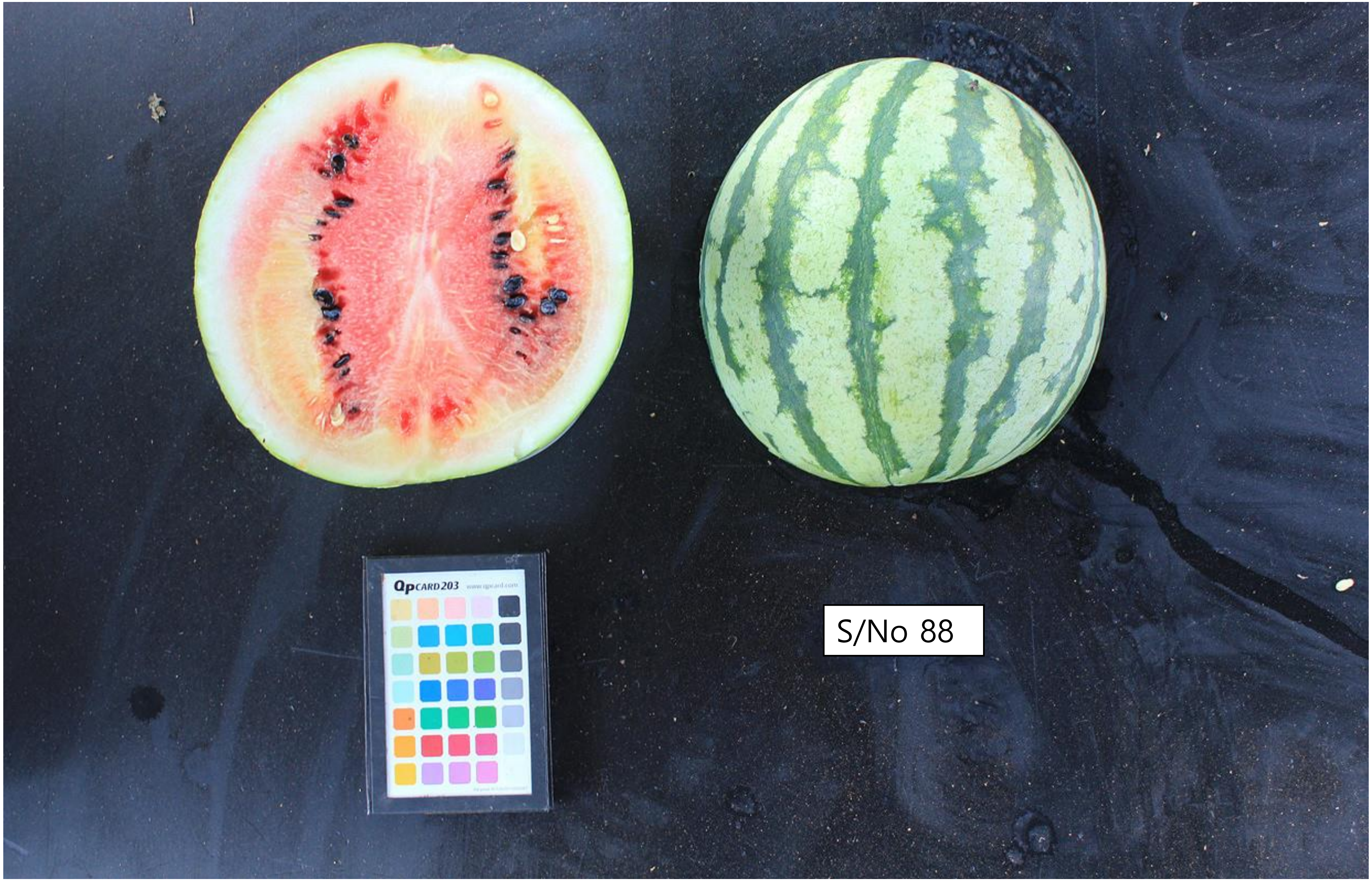
QpCARD 203
S/No 88

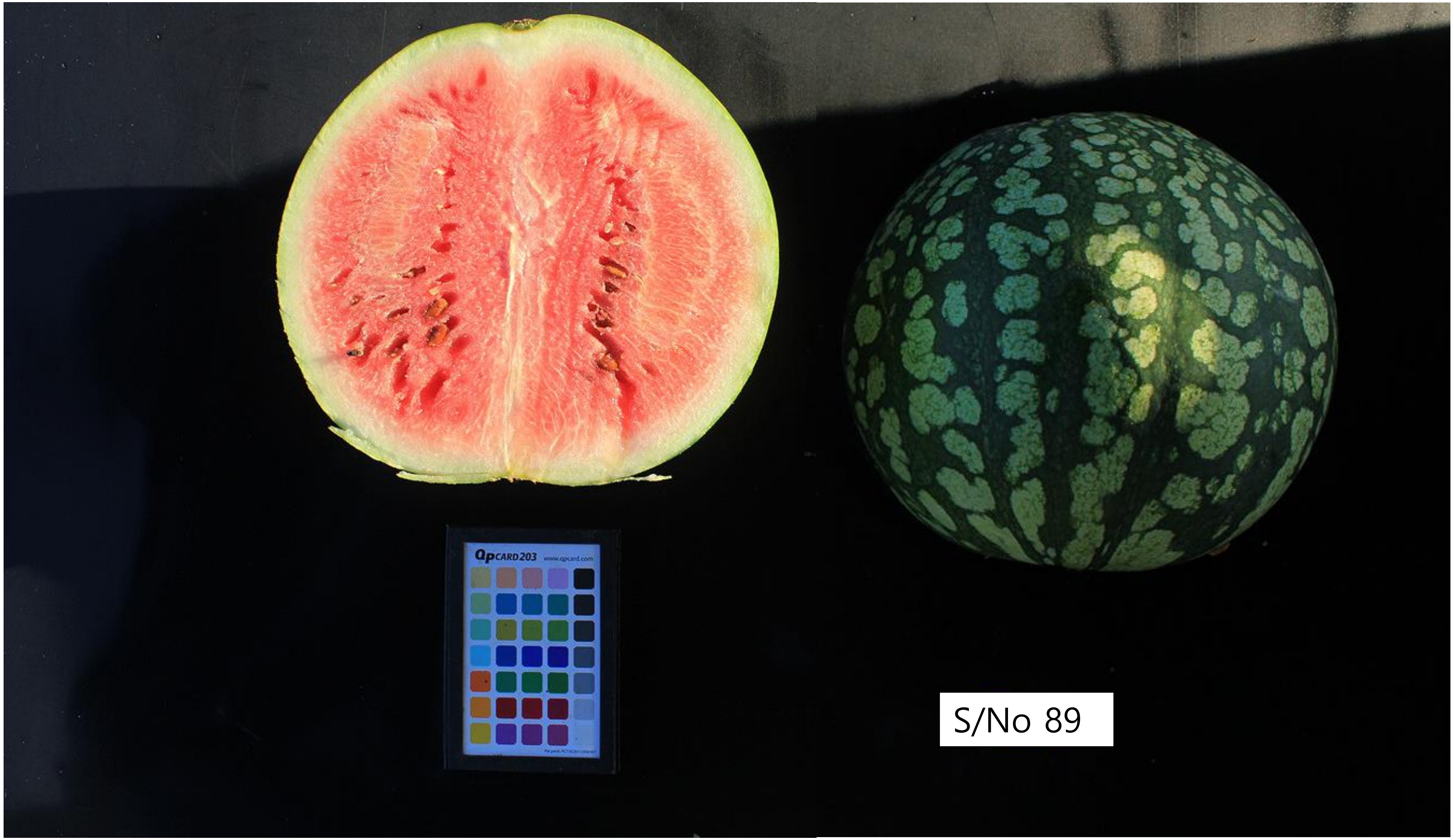
QpCARD 203
www.qpcard.com
S/No 89

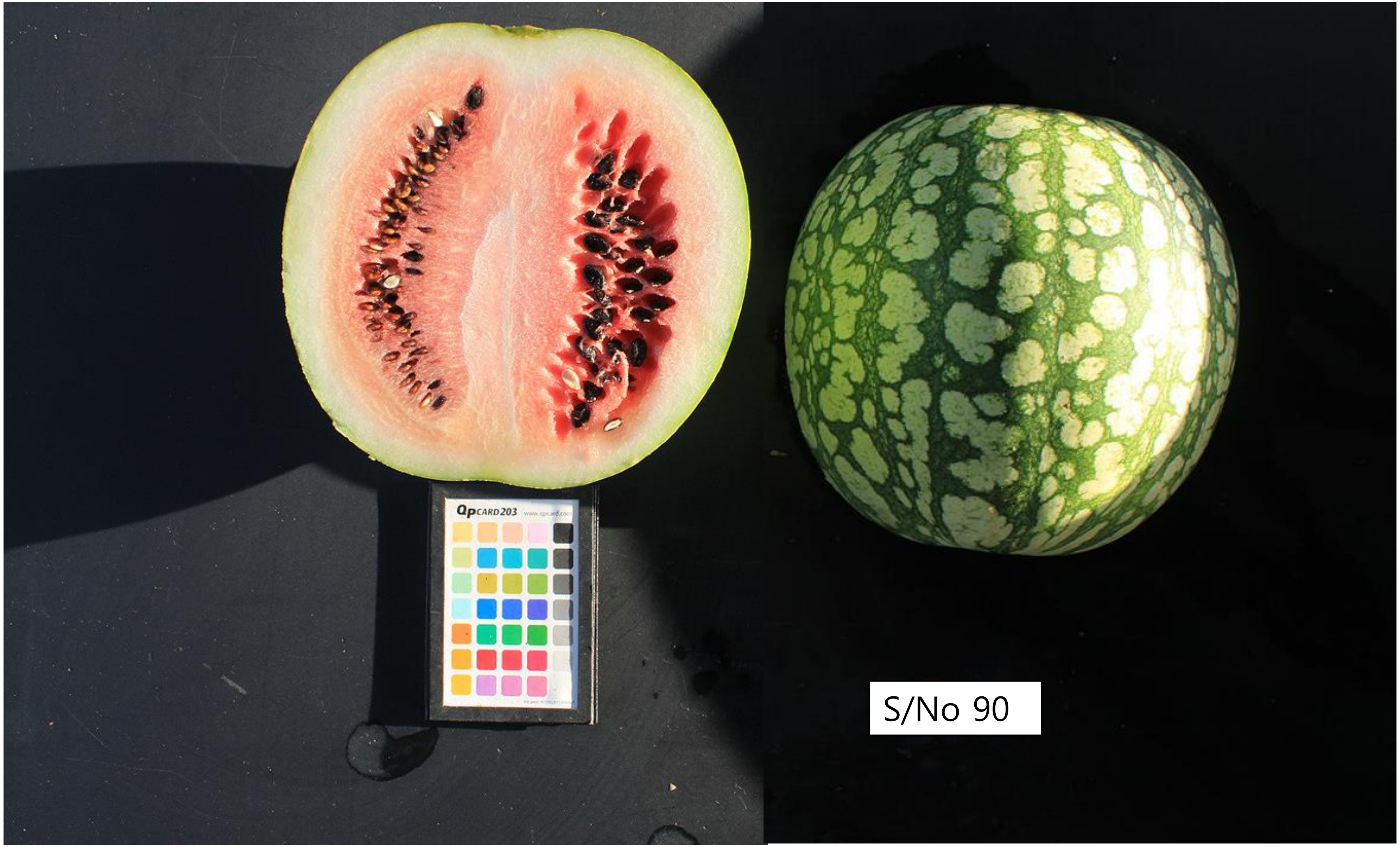
QpCARD203
S/No 90

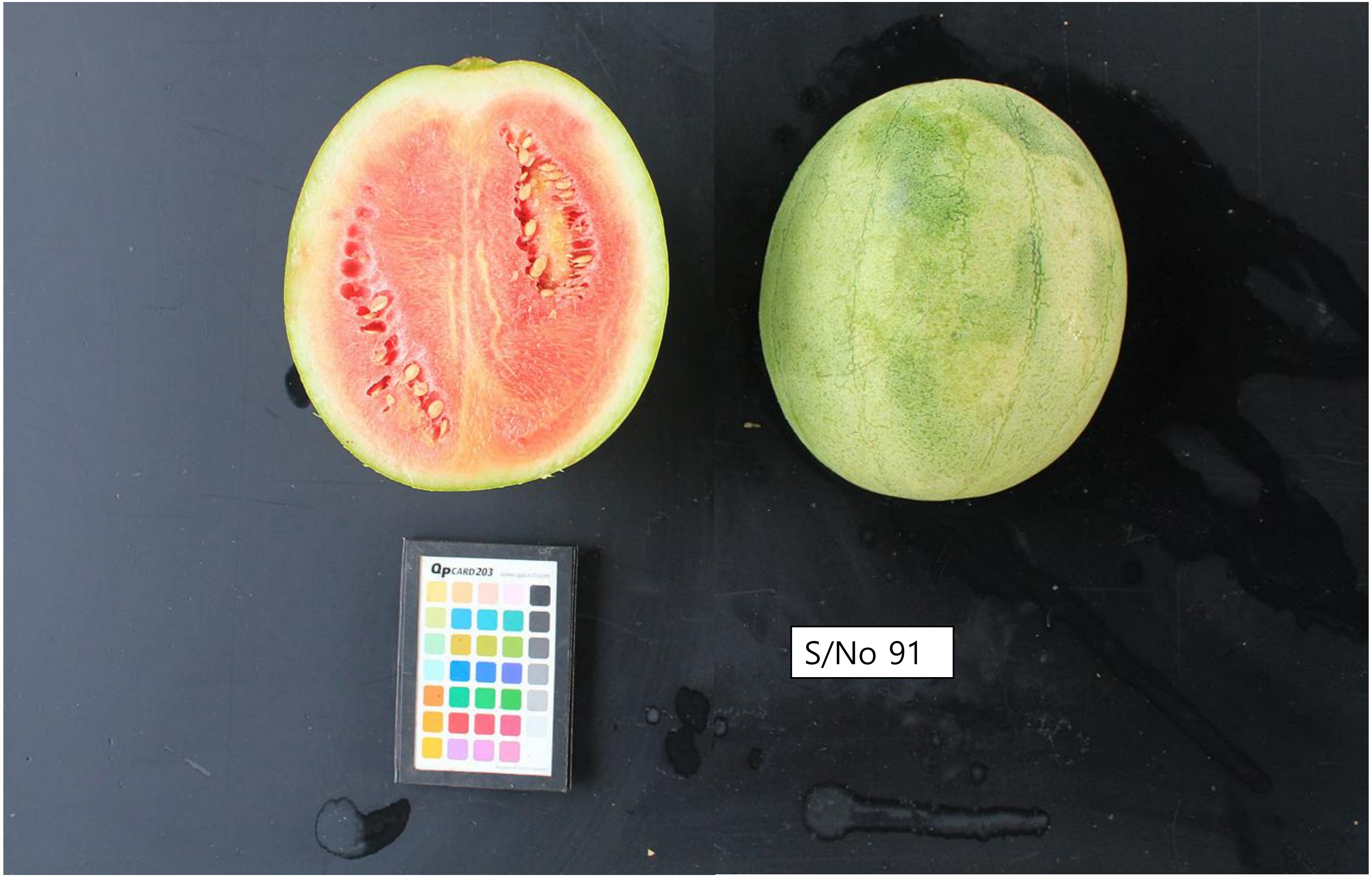
QpCARD203
S/No 91

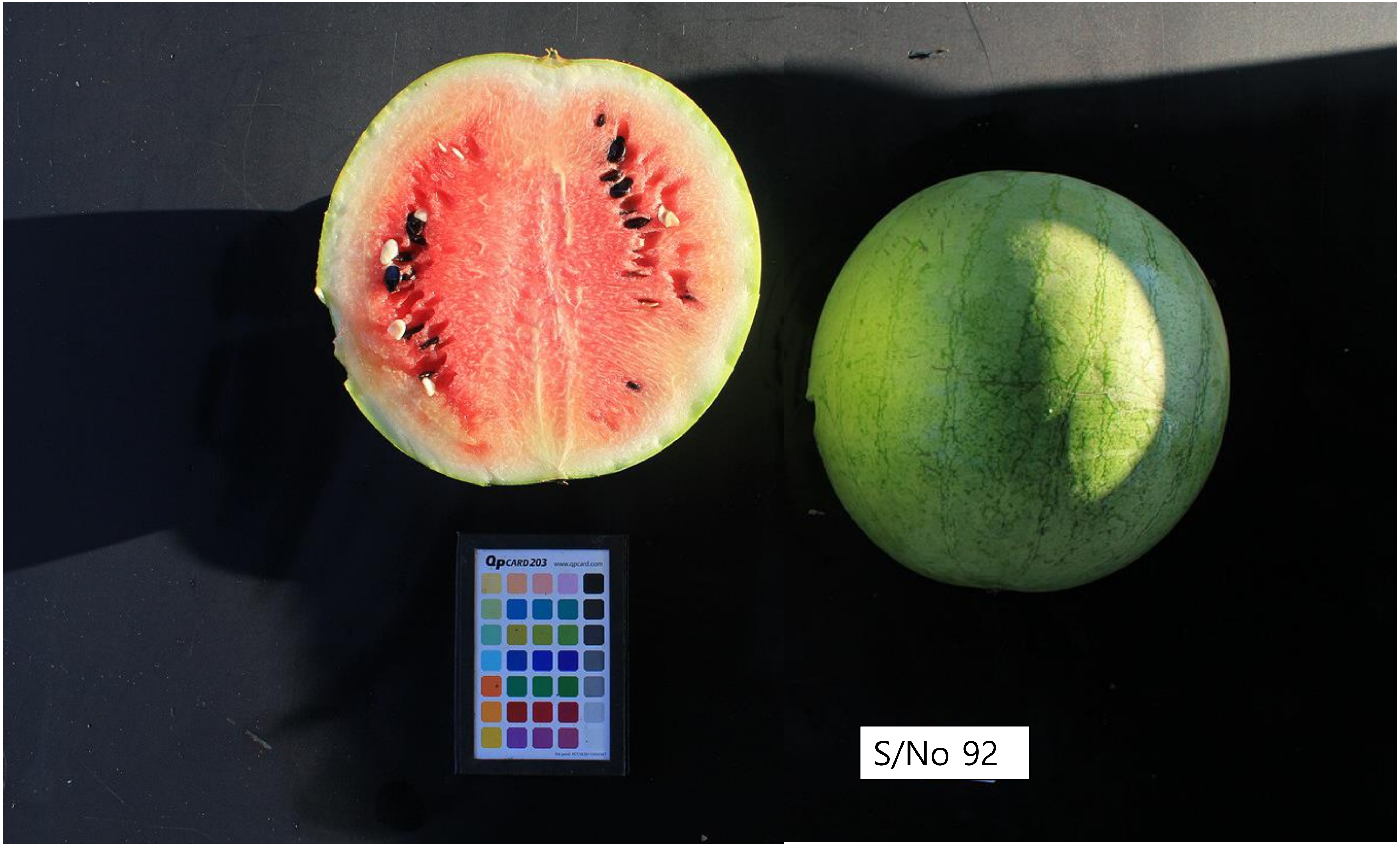
QpCARD203
www.qpcard.com
S/No 92

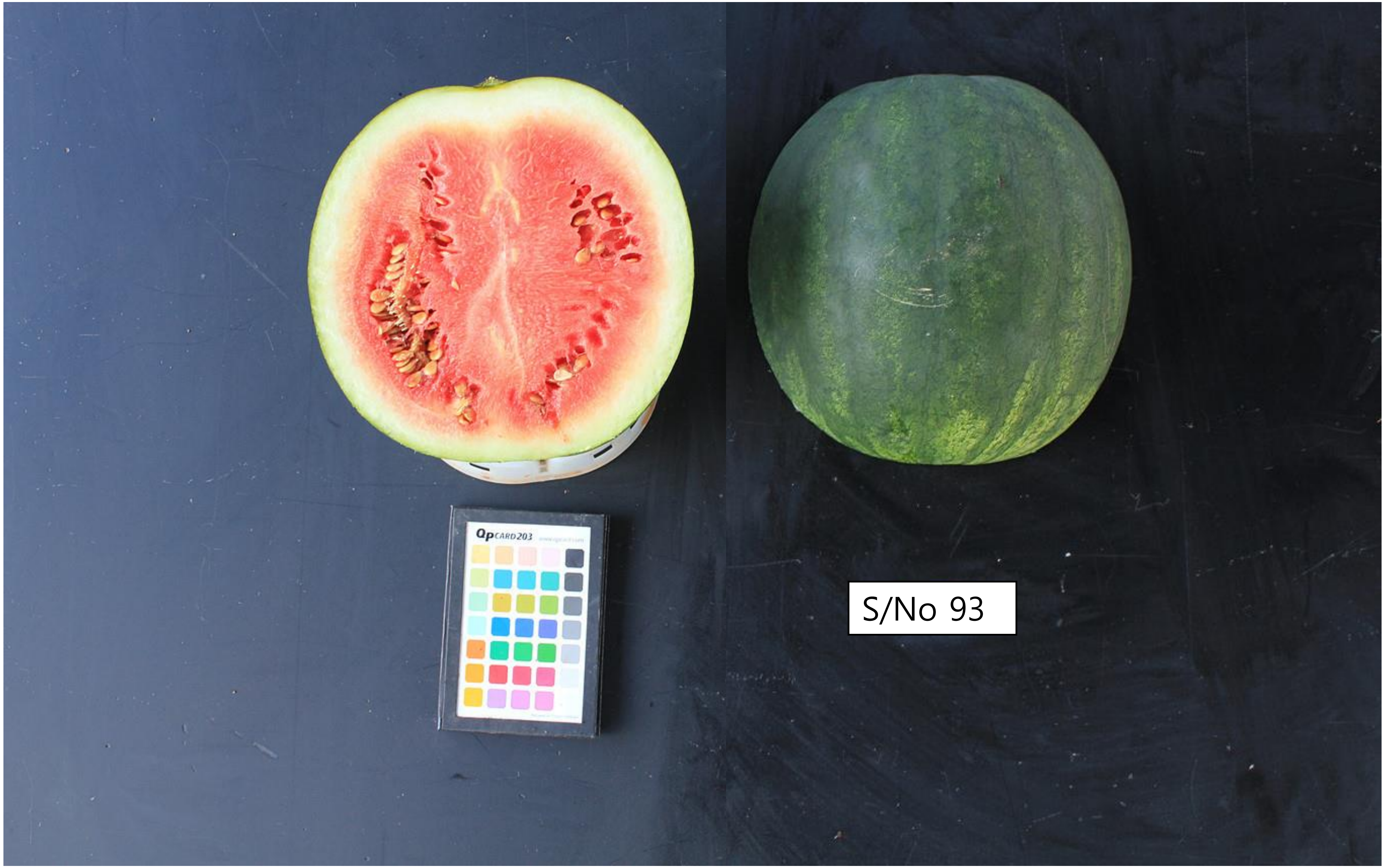
QpCARD203
S/No 93

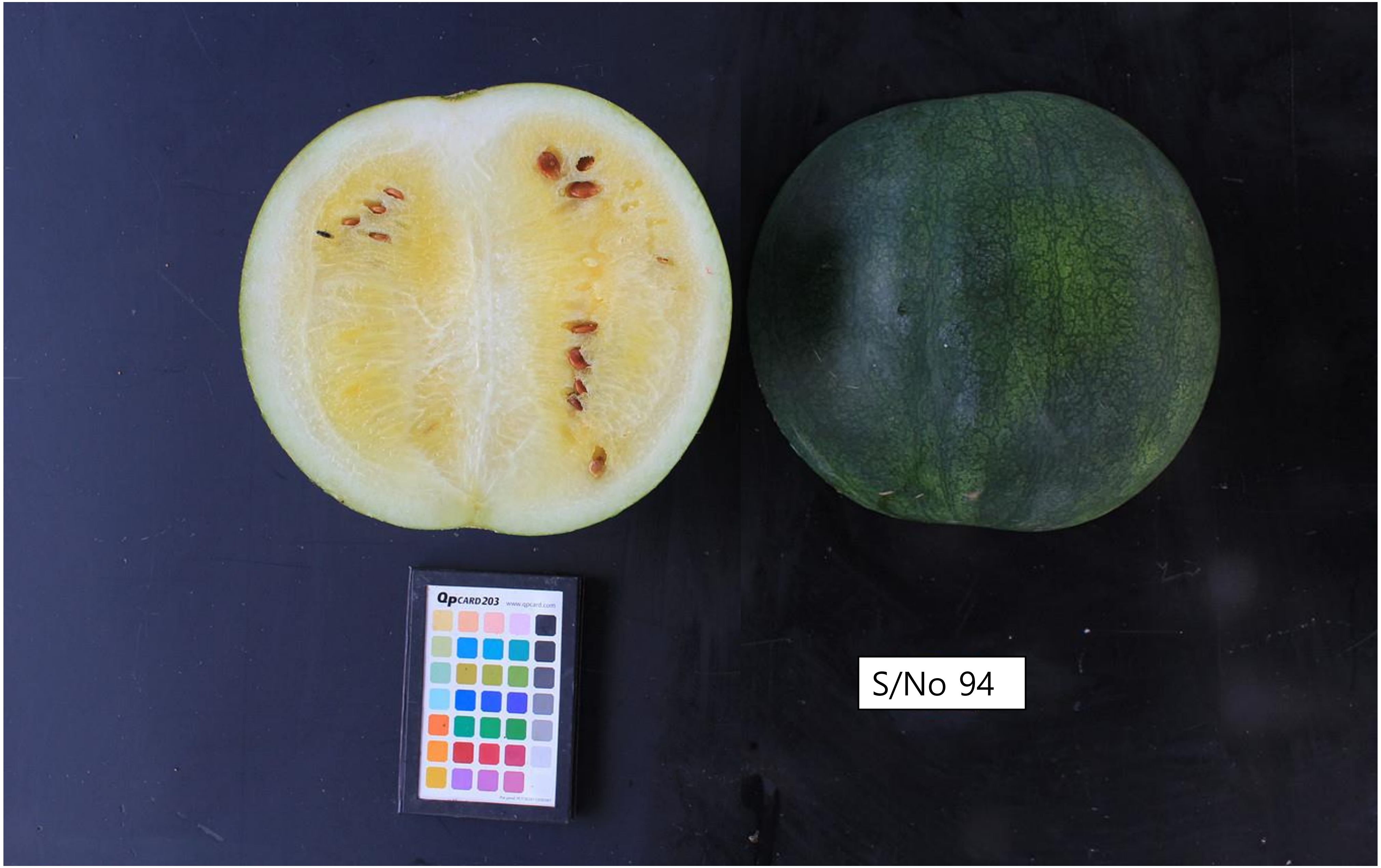
QpCARD 203
www.qpcard.com
S/No 94

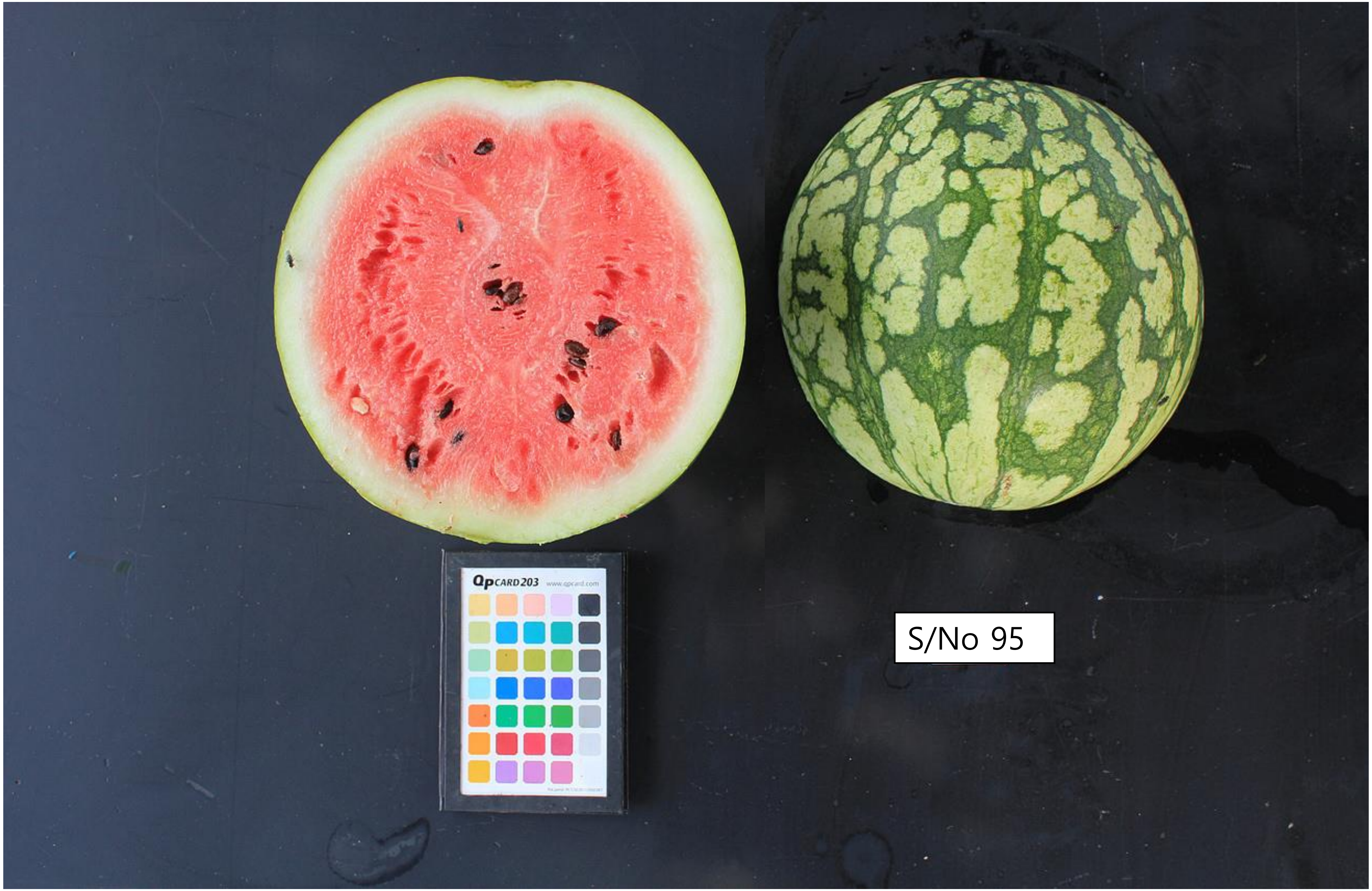
QpCARD 203
www.qpcard.com
S/No 95

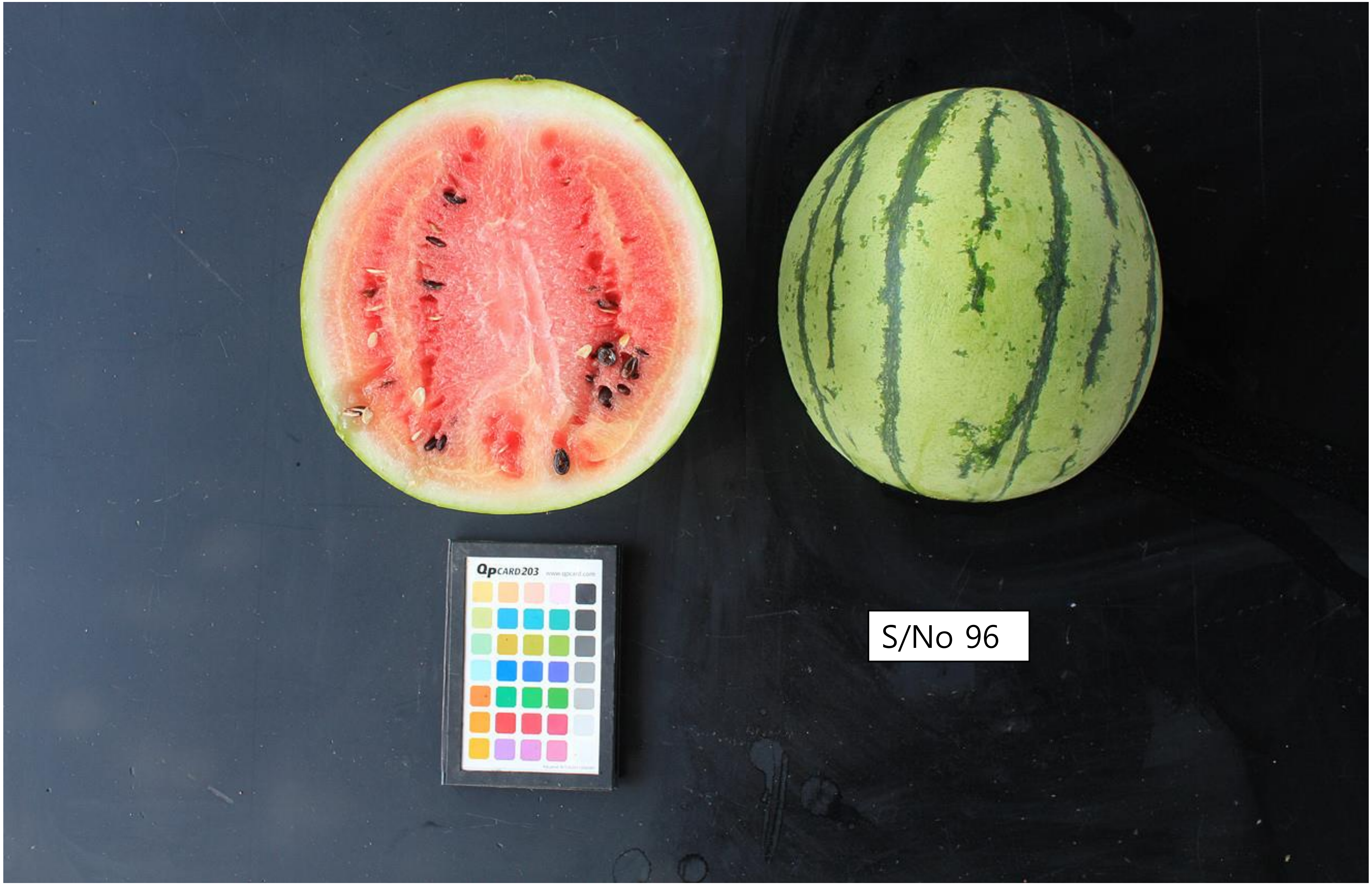
QpCARD 203
S/No 96

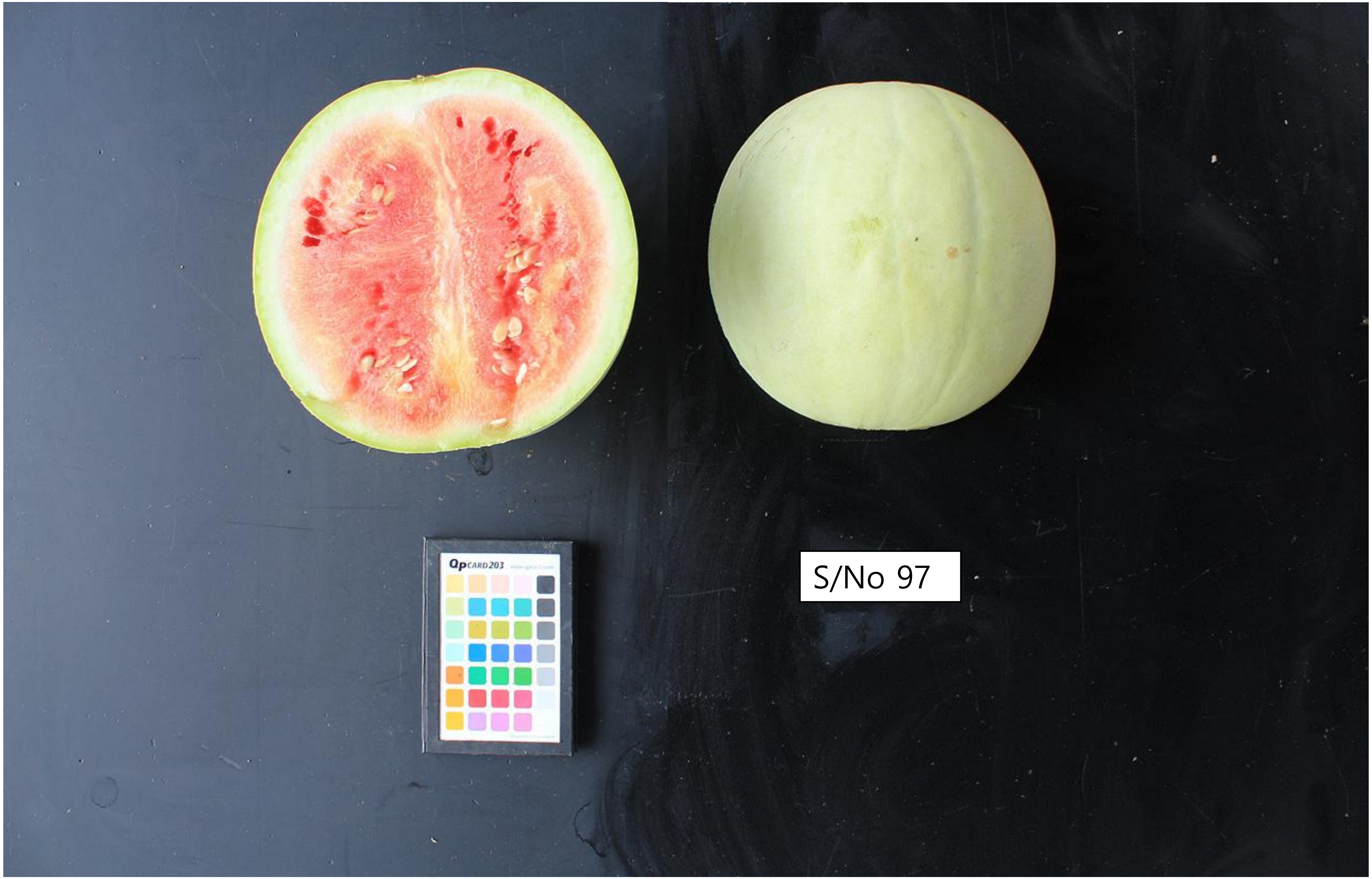
QpCARD 203
S/No 97

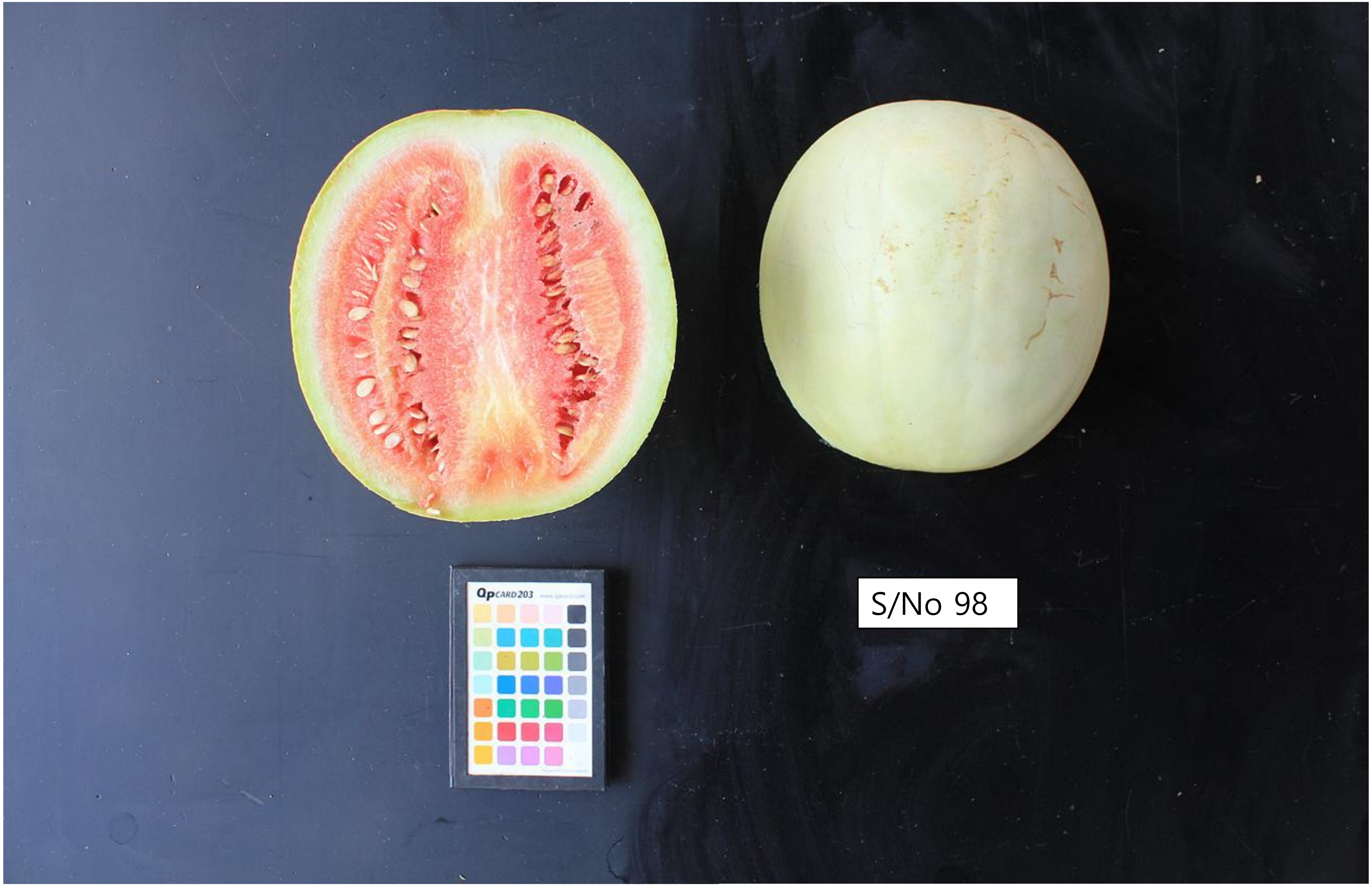
QpCARD203
S/No 98

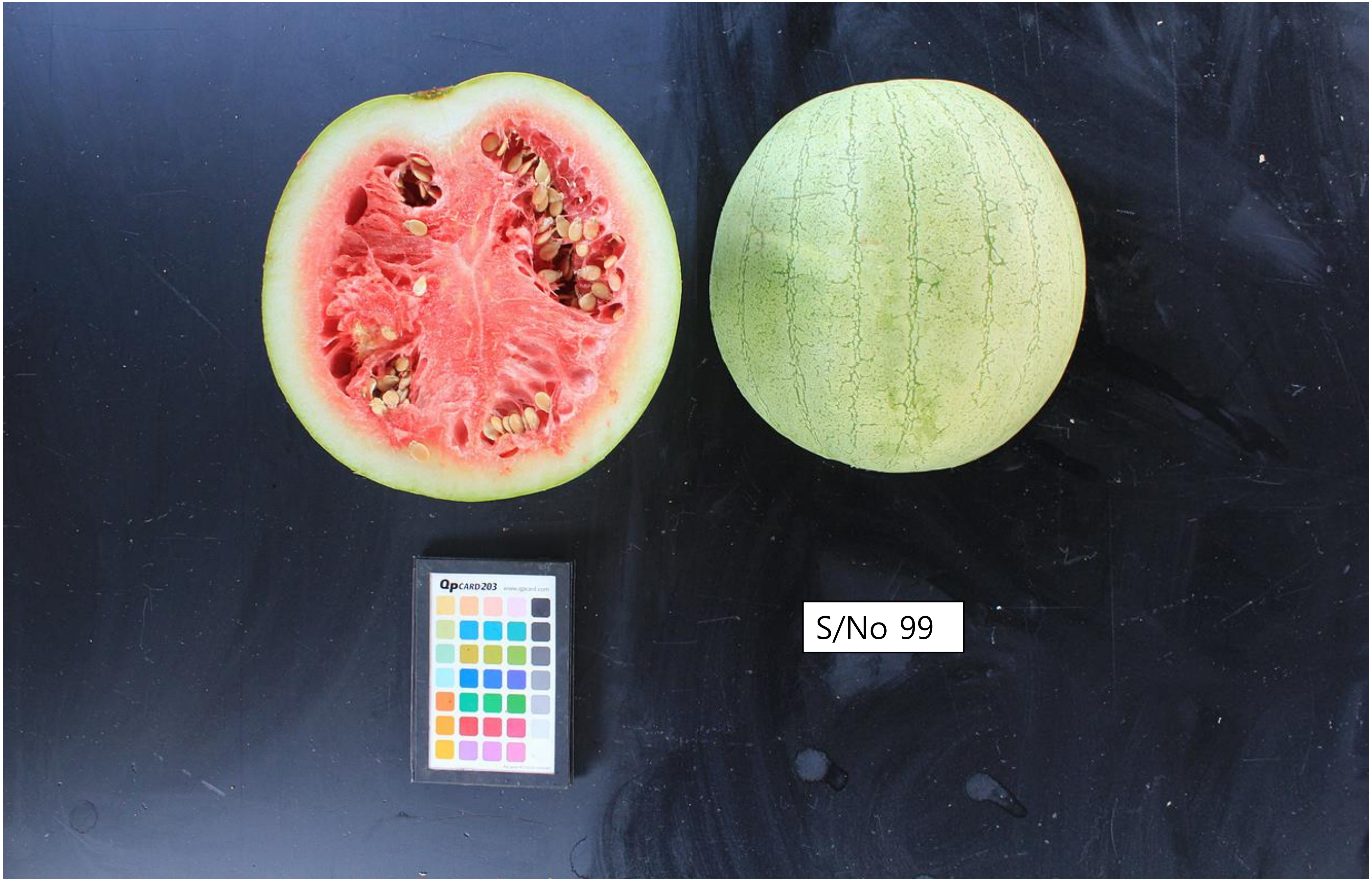
QpCARD203
S/No 99

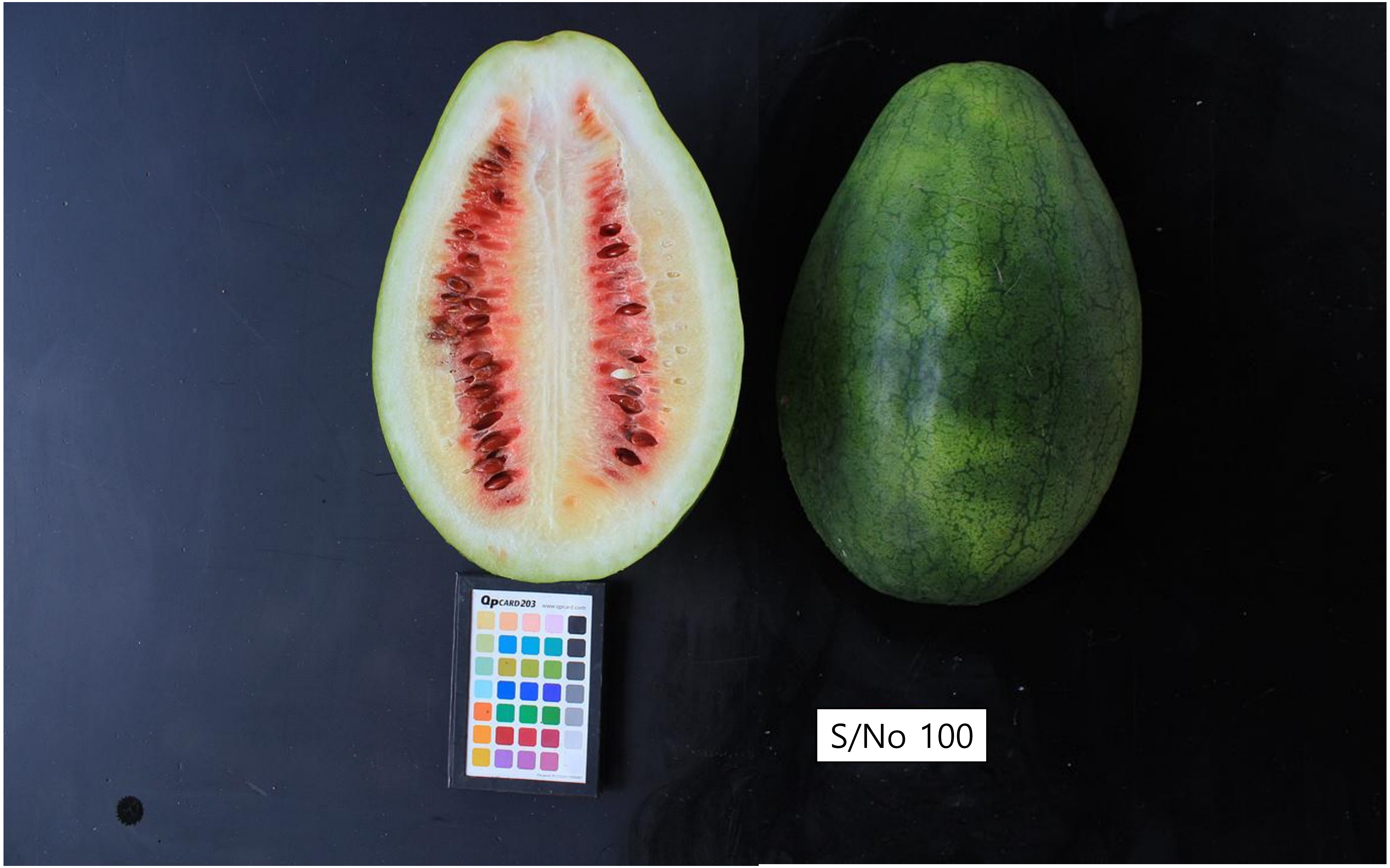
QpCARD 203
S/No 100

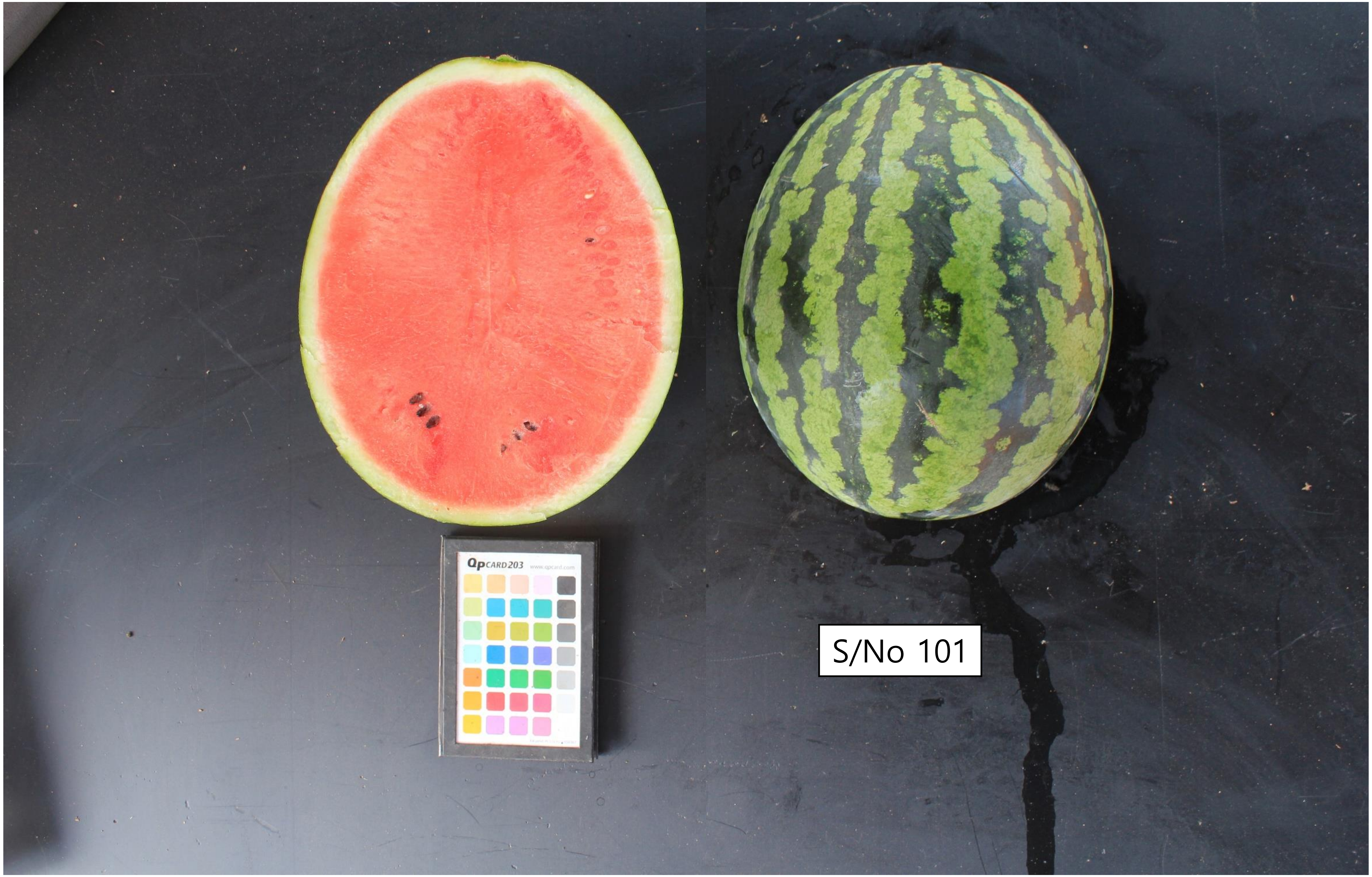
QpCARD 203
www.qpcard.com
S/No 101

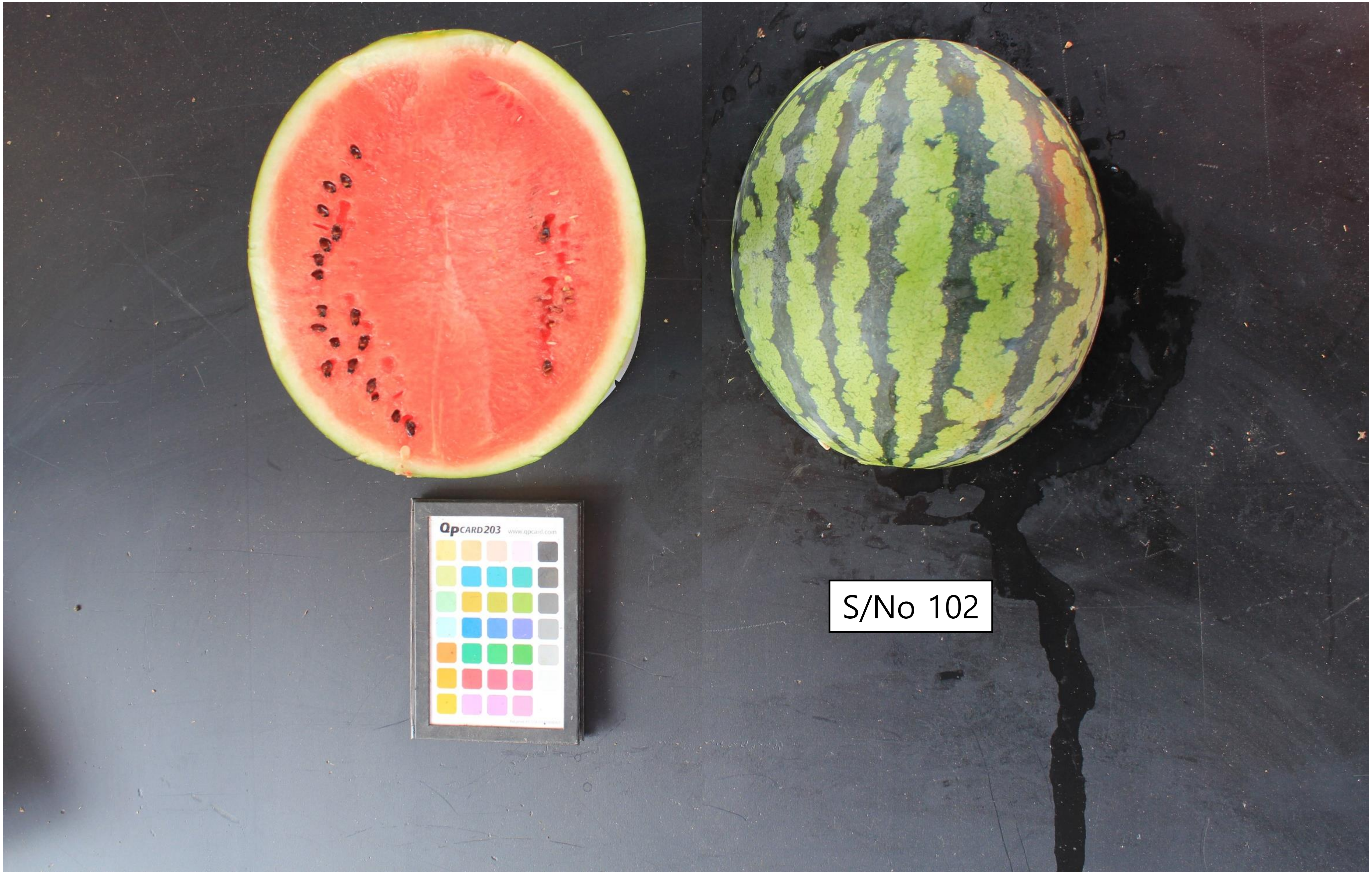
QpCARD203
www.qpcard.com
S/No 102

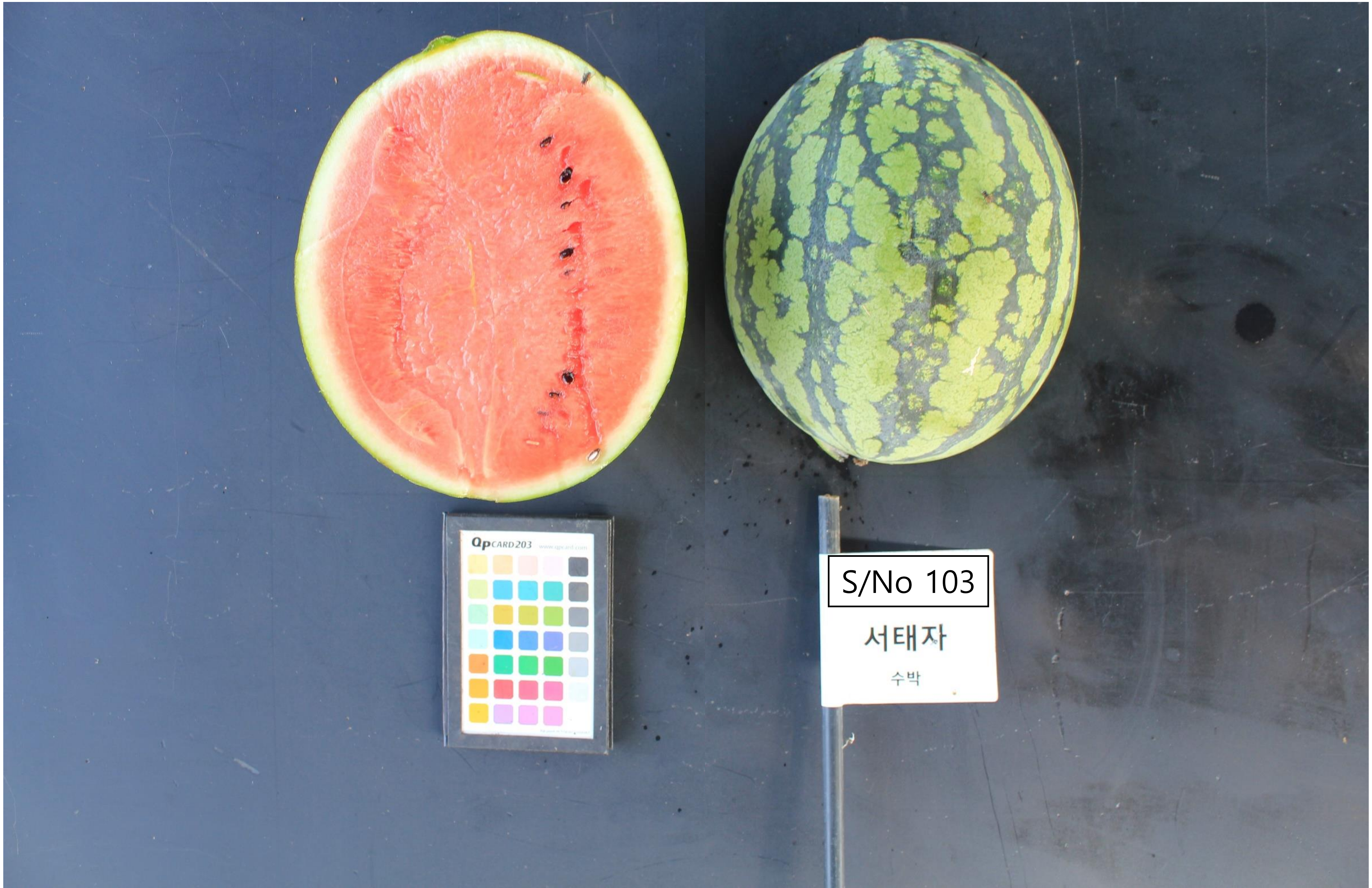
QpCARD 203
S/No 103
서태자
수박

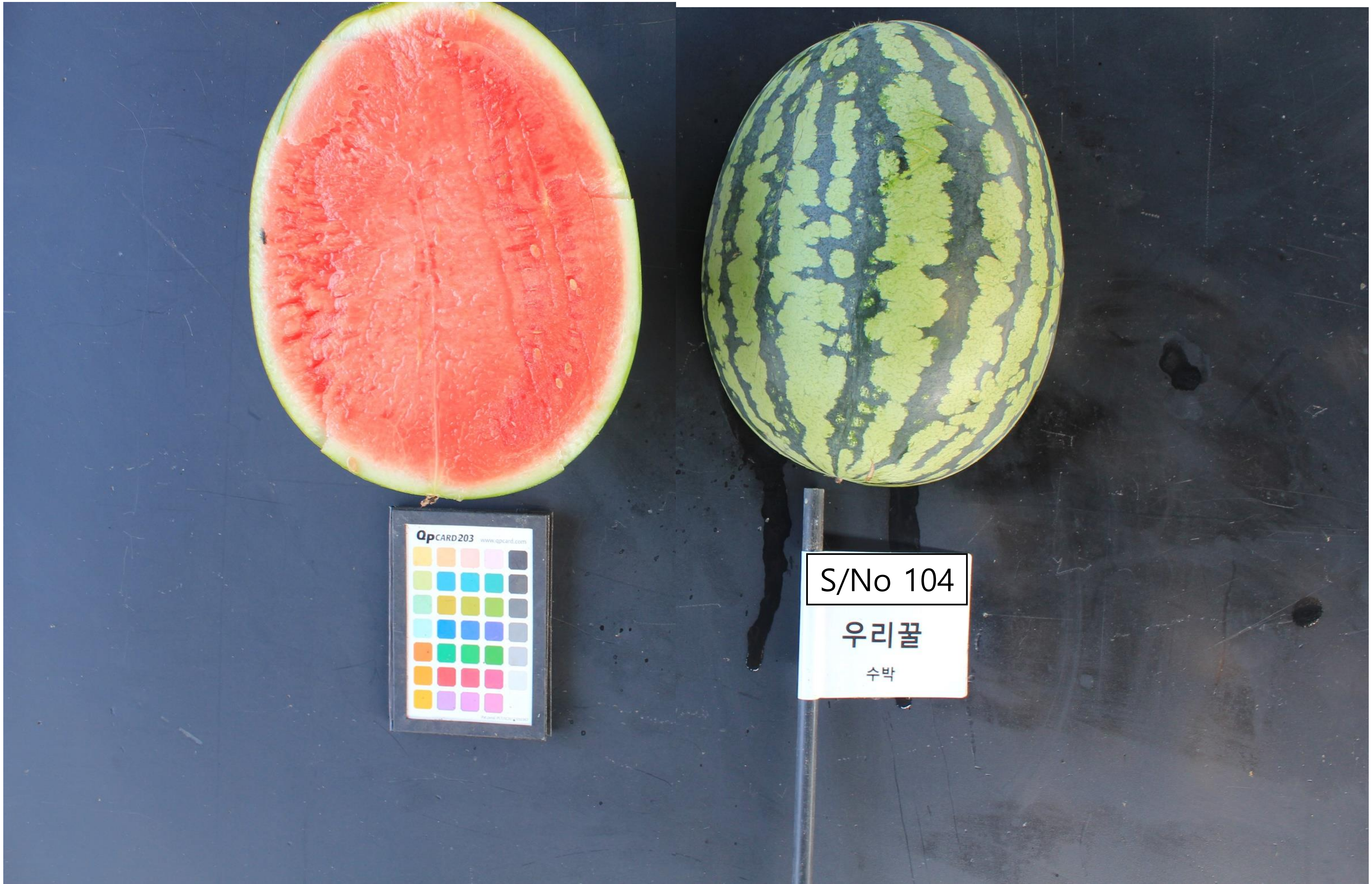
QpCARD 203
S/No 104
우리꿀
수박

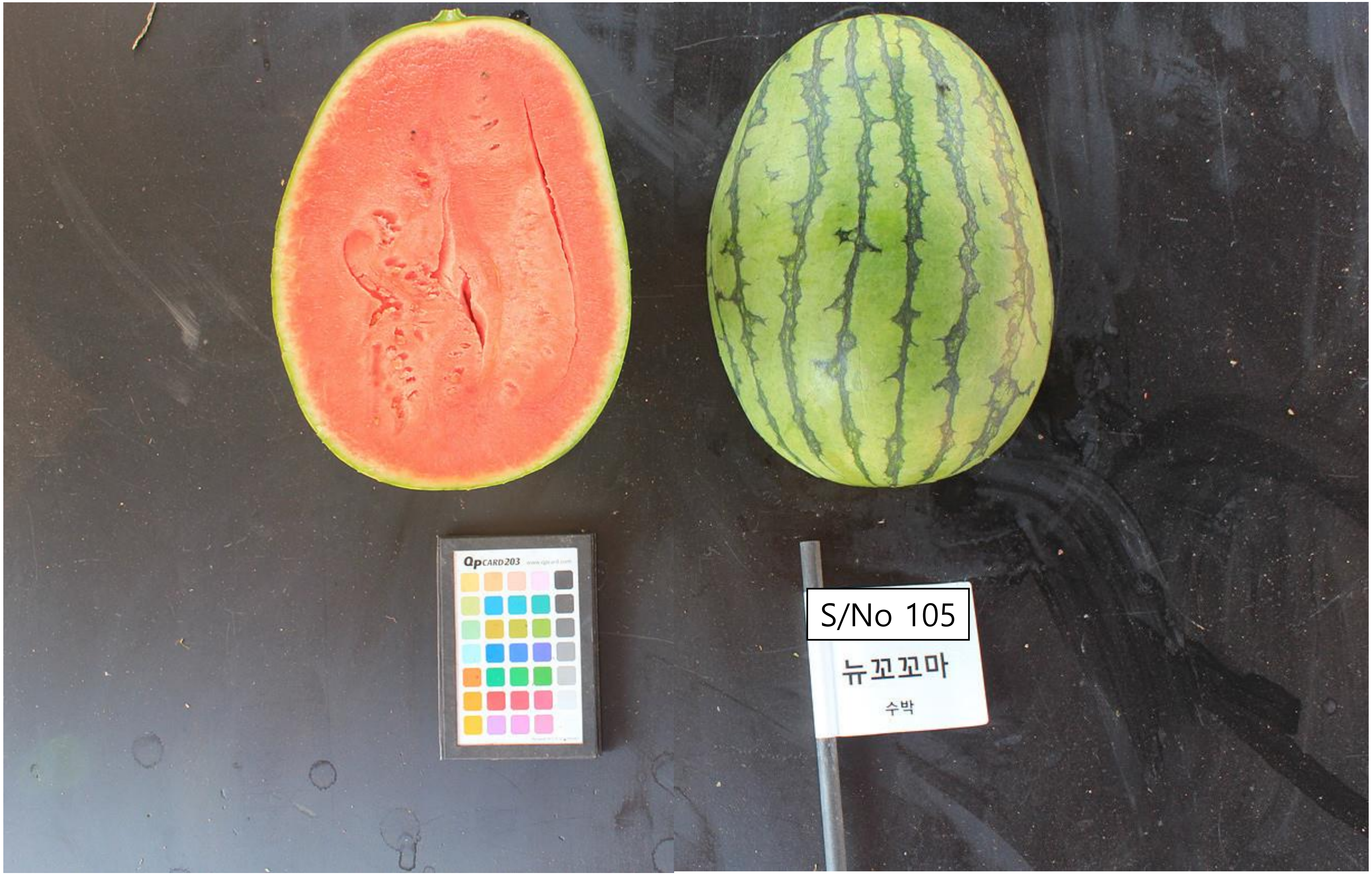
QpCARD 203
S/No 105
뉴꼬꼬마
수박

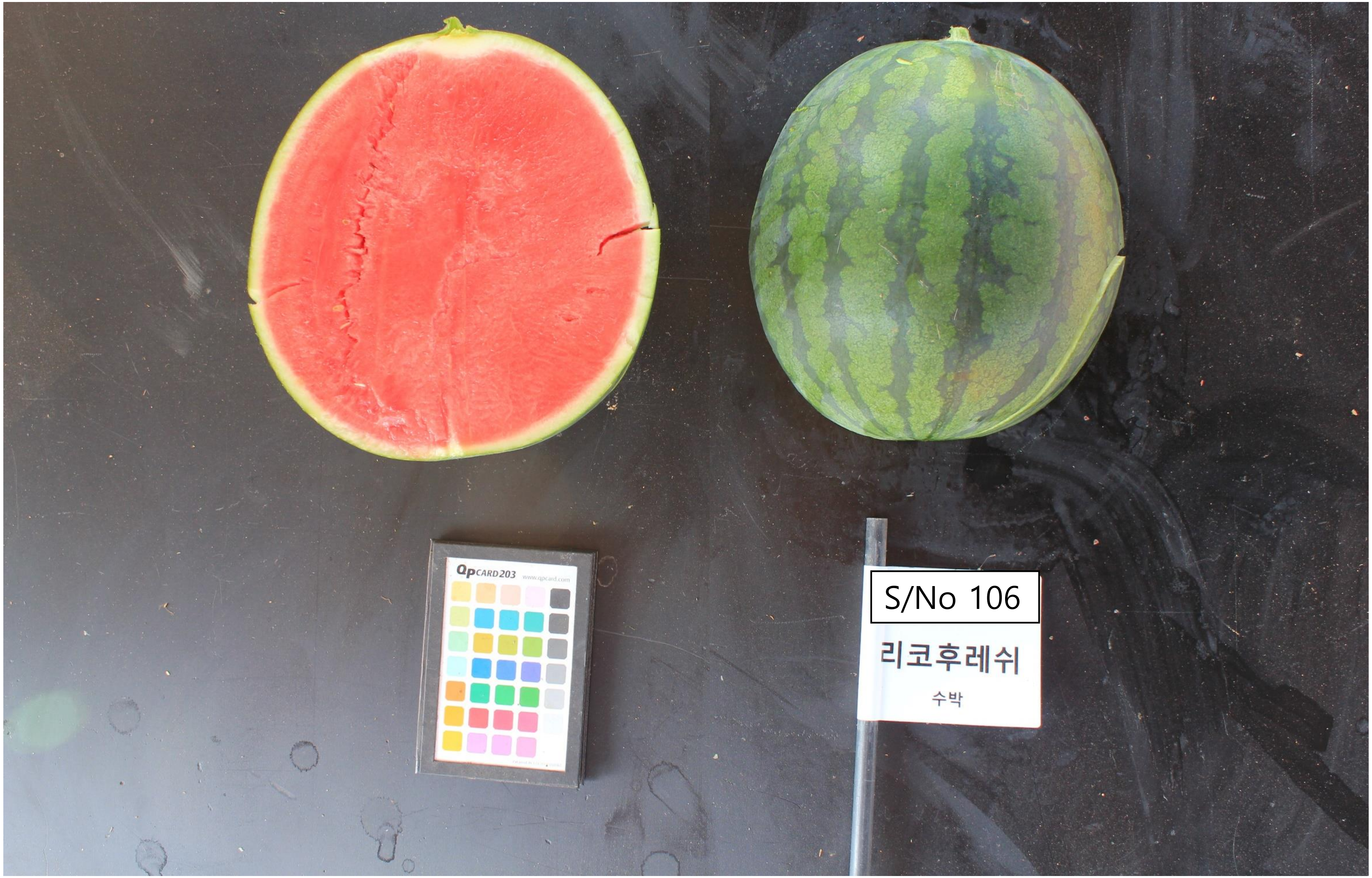
QpCARD 203
www.qpcard.com
S/No 106
리코후레쉬
수박

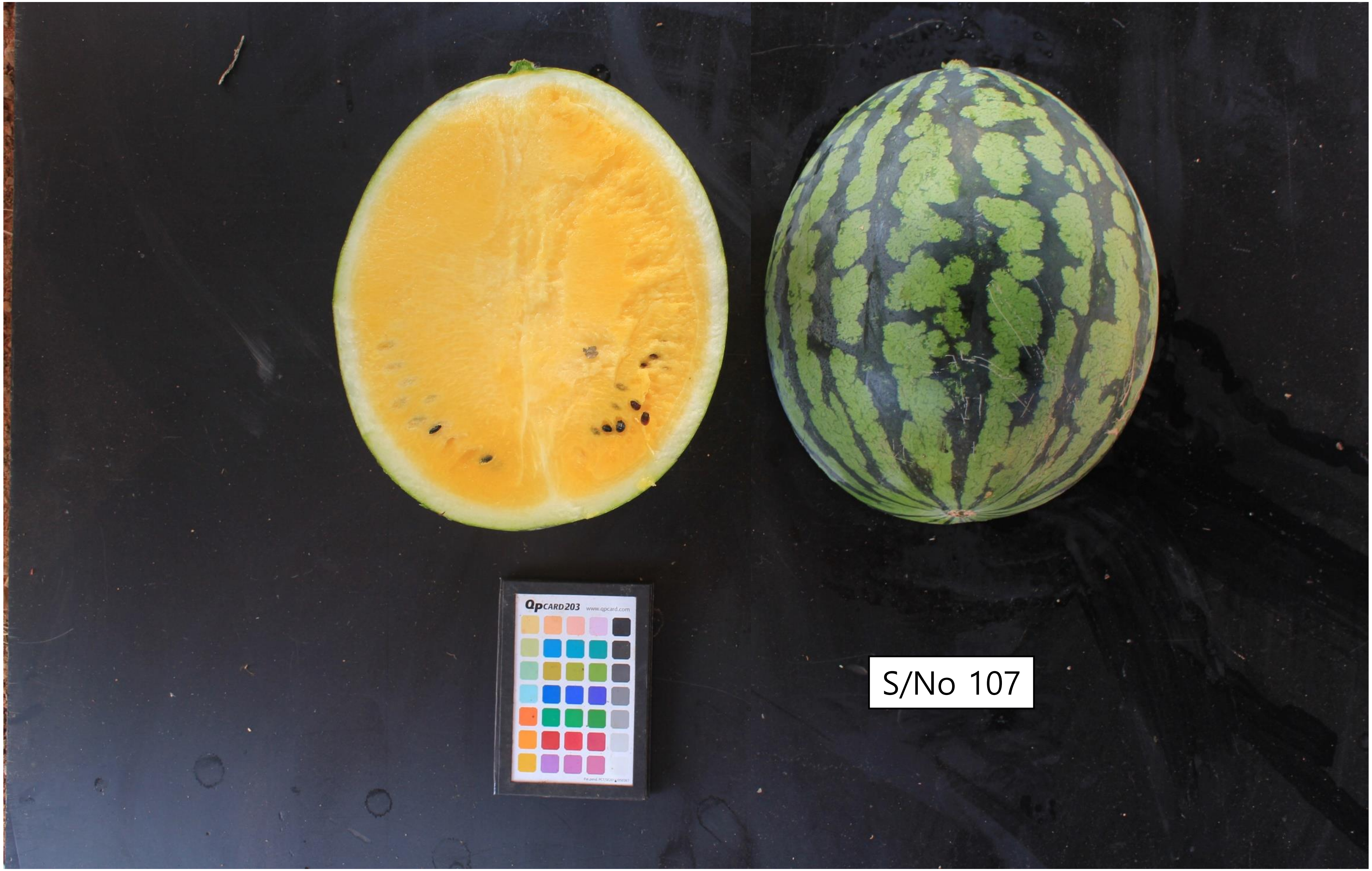
QpCARD203
www.qpcard.com
S/No 107
